# Kinetic Resolution of Allyltriflamides through a Pd-Catalyzed C–H Functionalization with Allenes: Asymmetric Assembly of Tetrahydropyridines

**DOI:** 10.1021/jacs.1c01929

**Published:** 2021-03-02

**Authors:** José Manuel González, Borja Cendón, José Luis Mascareñas, Moisés Gulías

**Affiliations:** Centro Singular de Investigación en Química Biolóxica e Materiais Moleculares (CIQUS) and Departamento de Química Orgánica, Universidade de Santiago de Compostela, 15782 Santiago de Compostela, Spain

## Abstract

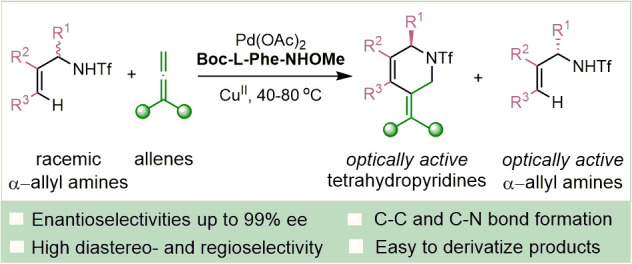

Enantioenriched, six-membered azacycles are essential structural motifs in many products of pharmaceutical or agrochemical interest. Here we report a simple and practical method for enantioselective assembly of tetrahydropyridines, which is paired to a kinetic resolution of α-branched allyltriflamides. The reaction consists of a formal (4+2) cycloaddition between the allylamine derivatives and allenes and is initiated by a palladium(II)-catalyzed C–H activation process. Both the chiral allylamide precursors and the tetrahydropyridine adducts were successfully obtained in high yields, with excellent enantioselectivity (up to 99% *ee*) and selectivity values of up to 127.

The assembly of chiral products through the enantioselective functionalization of C–H bonds represents one of the more relevant challenges in modern organic synthesis.^1,2^ In recent years, a series of brilliant strategies to carry out this type of reactions using transition metal catalysis, and relying on the presence of directing groups, have been described. One of the most relevant approaches to generate asymmetry consists of the desymmetrization of prochiral C–H bonds using palladium catalysts and monoprotected amino acids as metal ligands, a strategy that was pioneered by the group of Yu.^3^ Inspired by this research, we have recently published a palladium-catalyzed desymmetrization of diarylmethanamine triflamides by reaction with allenes to form chiral tetrahydroisoquinolines.^3e^ Although these methodologies are appealing, they require the presence of symmetric groups in the molecule, which represents a significant restriction in terms of the structural variability that can be achieved. Furthermore, the limitation to aromatic substrates reduces possibilities for subsequent synthetic manipulations.

Alternatively, enantioselective reactions can also be performed using kinetic resolutions (KRs). These strategies present the intrinsic limitation of yield, but they enable the recovery of the precursors in an enantioselective manner and are very attractive in terms of scope.^4^ In this context, the group of Yu has recently reported a palladium-catalyzed kinetic resolution of α-branched benzyl amine derivatives via C–H iodination or cross-coupling reactions.^5a,5b^ They also reported the kinetic resolution of racemic alkyltriflamides via cross-coupling reactions with boronates (Scheme 1A).^5c^ Remarkably, related asymmetric reactions using allylamines, or involving the activation of any type of alkenyl C–H bond, have never been described. This scarcity might be associated with the notion that the alkenes could engage in secondary reactions or the perception that attaining effective chiral discrimination might be especially challenging in comparison with reactions involving aromatic Csp^2^–H bonds (Scheme 1C).^6^ However, the catalytic asymmetric synthesis of alkene-containing products is of high interest owing to the elaboration possibilities offered by the presence of double bonds.

**Scheme 1:**
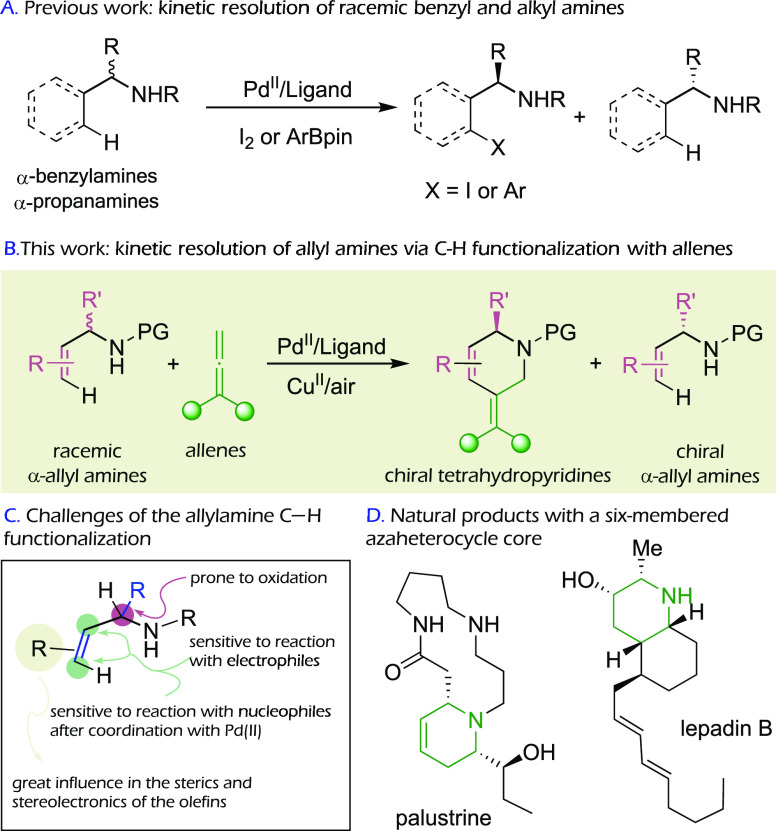
Kinetic Resolution of α-Branched Amines

Herein we report the discovery of a practical and efficient methodology for the kinetic resolution of α-branched allyltriflamides based on a Pd-catalyzed C–H activation process (Scheme 1B). Very importantly, this resolution is performed using highly substituted alkenes, which are traditionally difficult substrates for C–H functionalization reactions. Furthermore, the activation is coupled to a formal cycloaddition rather than to a simple C–H functionalization, therefore allowing a rapid increase in structural complexity, in this case, to give enantioenriched tetrahydropyridine skeletons. Needless to say, tetrahydropyridine and related piperidine scaffolds form the basic heterocyclic core of many chiral natural products and bioactive structures (Scheme 1D). Therefore, the development of practical, direct methods for the enantioselective synthesis of these types of products is of major interest.^7^ Importantly, our reaction also provides a direct access to enantioenriched allylamine derivatives, which are very useful for the construction of a variety of optically active nitrogenated products.

We started our research by surveying conditions that could enable the annulation of racemic allyltriflamide **1a** with the commercially available allene **2a**.^3g,8^ After an extensive screening, we found that heating this mixture in toluene/DMSO (10:1), in the presence of 10 mol% of palladium acetate, 2 equiv of copper acetate, 1.5 equiv of cesium carbonate, and 40 mol% Boc-l-Phe-NHOMe,^5e^ provides the desired cycloadduct **3aa** with an excellent 94% *ee* (49% conversion after 24 h) (Table 1, entry 1). The remaining starting material was isolated with a 90% *ee*. Decreasing the amount of ligand favors the conversion rate but affects the enantiomeric excess of the product. Not surprisingly, when Boc-d-Phe-NHOMe was used as ligand, the opposite enantiomers were obtained with the same enantioselectivity. Changing the ligand to other diprotected (entries 2 and 3) or monoprotected amino acids (entries 4–8) resulted in lower conversions and poorer *ee*’s. It should be noted that, although the use of Boc-phenylalanine as ligand led to a moderate enantioselectivity (58% *ee*), it made possible to recover the starting amide with 99% enantioselectivity (entry 4). Indeed, this ligand gave also good results for some of the other substrates tested (see below, Scheme 3). Significant decreases in conversion and enantioselectivity were observed when the *tert*-butyloxycarbonyl protecting group of the amino acid was replaced by an acetyl, trichloro-*tert*-butyloxycarbonyl, or difluorobenzoyl group (entries 9–11). Moreover, we found that, when using less copper acetate, or replacing it with other oxidants, such as silver acetate or silver carbonate, the conversions and enantioselectivities were poorer (entries 12–14). The use of solvents other than toluene, such as THF, DCE, or isopropanol, clearly gave worse results in both conversion and selectivity (entries 15–17), while with *tert*-amyl alcohol we obtained an *ee* of 80% for the product and an excellent 99% *ee* for the recovered starting material (entry 18). We also found that, without base or DMSO, the reaction hardly progresses (entries 19 and 20), and the enantioselectivity is greatly diminished.

**Table 1:**
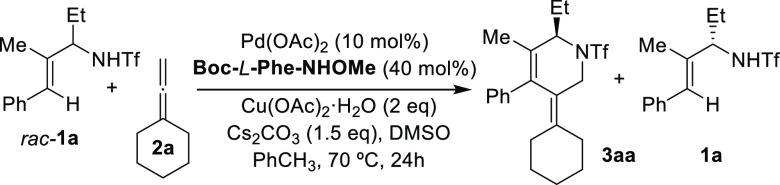
Optimization of Conditions^a^

			*ee* (%)^c^		
entry	deviation from above conditions^a^	*C*^b^ (%)	**3aa**	**1a**	*s*^d^
**1**	**none**	**49**	**94**	**90**	**100**
2	Boc-Val-NHOMe	45	82	66	20
3	Boc-Leu-NHOMe	37	88	52	26
4	Boc-Phe-OH	63	58	99	18
5	Boc-Val-OH	59	58	84	10
6	Boc-Leu-OH	64	54	98	14
7	Boc-*t-*Leu-OH	58	50	70	6
8	Boc-Ile-OH	65	50	92	9
9	TcBoc-Phe-OH	64	20	36	2
10	Ac-Phe-OH	31	48	22	4
11	2,6-F,F-Bz-Phe-OH	47	60	54	7
12	0,5 equiv of Cu(OAc)_2_·H_2_O	36	94	52	54
13	AgOAc as oxidant	9	86	8	14
14	Ag_2_CO_3_ as oxidant	6	92	6	25
15	THF as solvent	25	84	28	15
16	DCE as solvent	39	86	56	23
17	*i*-PrOH as solvent	51	78	80	20
18	*t*-AmylOH as solvent	55	80	99	40
19	no base	4	44	2	3
20	no DMSO	12	72	10	7

a Conditions: *rac*-**1a** (0.1 mmol), **2a** (0.1 mmol), Pd(OAc)_2_ (10 mol%), ligand (40 mol%), Cu(OAc)_2_·H_2_O (2 equiv), Cs_2_CO_3_ (1.5 equiv), DMSO (15 equiv), PhCH_3_ (1.2 mL), air, 70 °C, 24h.

b Calculated conversion, *C* = *ee*_SM_/(*ee*_SM_ + *ee*_PR_).

c Enantiomeric excess (*ee*) was determined by chiral HPLC analysis of the reaction crude.

d Selectivity (*s*) = ln[(1 – C)(1 – *ee*_SM_)]/ln[(1 – C)(1 + *ee*_SM_)].

The scope of the reaction with respect to the allene component was studied using the above optimized conditions, albeit with small adjustments in temperature, and the results are summarized in Scheme 2. Therefore, when the allyltriflamide was tested with a different 1,1-disubstituted allene (5-vinylidenenonane), it resulted in the formation of the cycloadduct **3ba** with a 90% *ee*, while the starting amide was recovered with 82% *ee* (selectivity factor of 48).

**Scheme 2:**
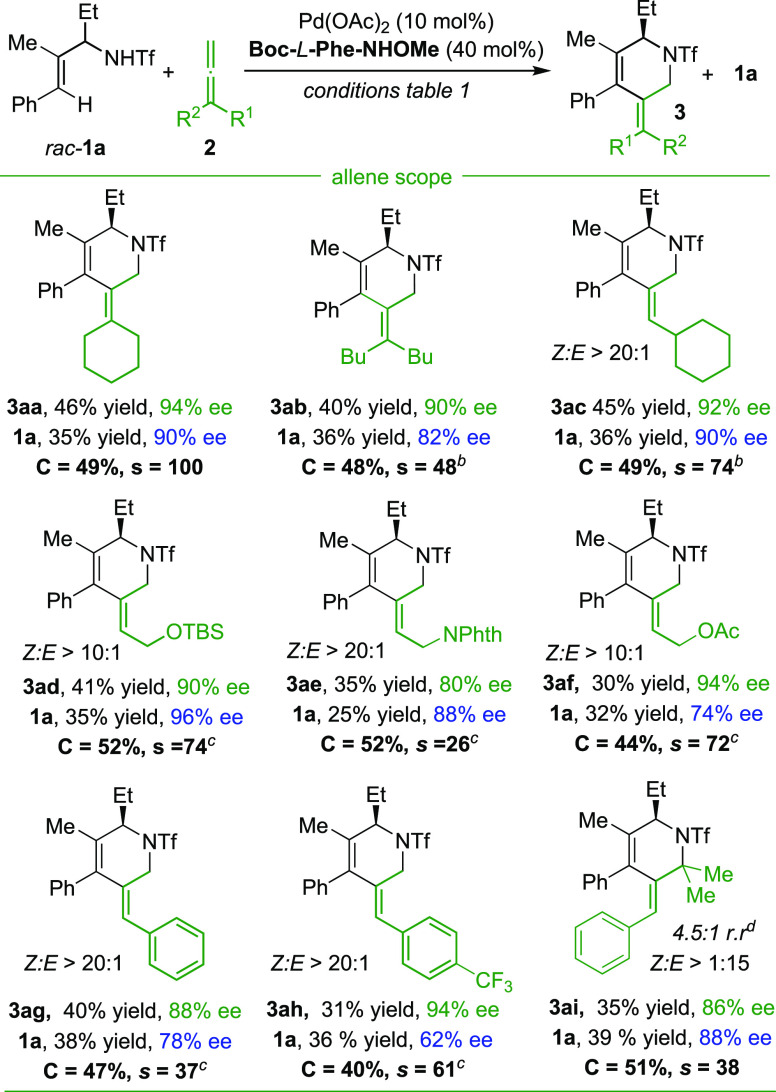
Scope of the Formal (4+2) Cycloaddition of Allyltriflamides and Allenes Conditions: *rac*-**1a** (0.1 mmol), **2** (0.1 mmol), Pd(OAc)_2_ (10 mol%), ligand (40 mol%), Cu(OAc)_2_·H_2_O (2 equiv), Cs_2_CO_3_ (1.5 equiv), DMSO (15 equiv), PhCH_3_ (1.2 mL), air, 70 °C, 24 h. 75 °C. 80 °C. Only the major regioisomer is depicted (see Supporting Information for more details).

The reaction also works very nicely with monosubstituted allenes such as commercially available propa-1,2-dien-1-ylcyclohexane (**2c**), the silyl-protected buta-2,3-dien-1-ol **2d**, or allenes containing acetate (**2f**) and phthalimide (**2e**) functional groups, leading to enantioselectivities up to 94% in the products and over 96% in the corresponding starting materials. In contrast, buta-2,3-dien-1-ol failed to participate in the annulation, and the reaction provided a complex mixture of products. In the case of allenes with aromatic substituents (**2g** and **2h**), we observed very good enantiomeric excess in the products (up to 94% *ee*) but more modest enantioselectivities for the starting materials (62–78% *ee*). Remarkably, trisubstituted allenes (**2i**) can also work with only a slight erosion of the enantioselectivity of the product (86% *ee*). In this reaction we also observed the formation of a small proportion of a different regioisomer.

Gratifyingly, in most of the cases the products were formed with high *E:Z* diastereoselectivity. The preferred formation of the *Z*-isomer with monosubstituted allenes is likely a consequence of steric effects associated to the interaction of the phenyl group of the alkene with the substituent of the allene.

The optimized conditions were also used for further evaluating the scope of the reaction with regard to the allyltriflamide precursors. Pleasingly, we found that allylamine derivatives with other groups at the α-position, including methyl, butyl, and isobutyl, are well tolerated. In all cases the reactions took place with good yields and excellent enantioselectivities (Scheme 3, **1b**–**1d**, up to 94% *ee* for products and starting materials). The crystallization of compounds **3ba** and **1b** allowed determination of the absolute configuration *R* and *S*, respectively, through X-ray crystallographic analysis (Scheme 3, top right). [Their crystal structure data are deposited to the CCDC.]

**Scheme 3:**
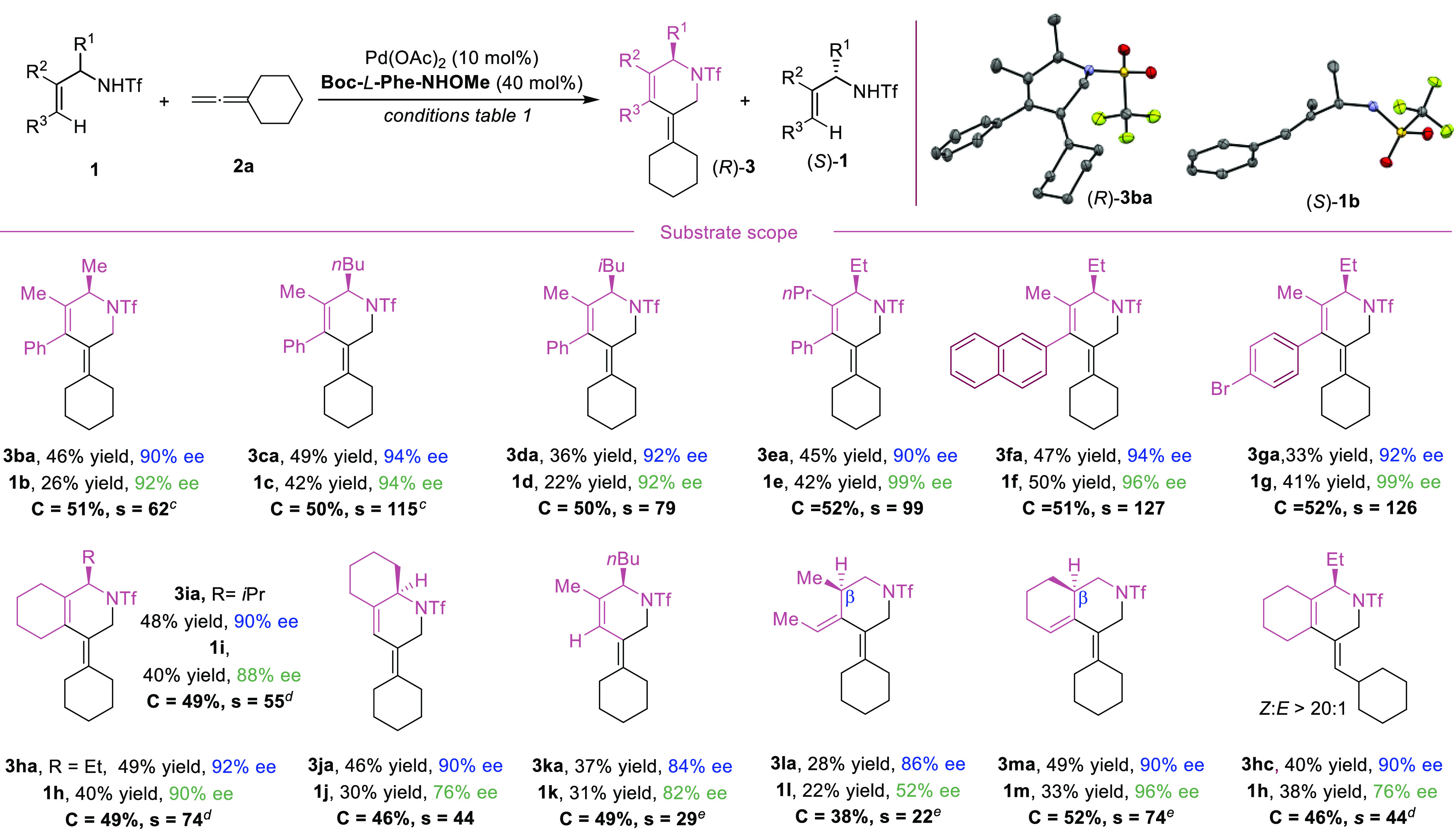
Scope of the Formal (4+2) Cycloaddition of Allyltriflamides and Allenes^,^ Conditions: *rac*-**1** (0.1 mmol), **2a** (0.1 mmol), Pd(OAc)_2_ (10 mol%), ligand (40 mol%), Cu(OAc)_2_·H_2_O (2 equiv), Cs_2_CO_3_ (1.5 equiv), DMSO (15 equiv), PhCH_3_ (1.2 mL), air, 70 °C, 24 h. Hydrogens of crystal structures omitted for clarity. 75 °C. 80 °C. Boc-Phe-OH as ligand, 40 °C.

We also analyzed the reactivity of the precursors with other substituents at the terminal and internal positions of the alkene moiety. As illustrated with the formation of products **3ea**–**3ia** and enantioenriched precursors **1e**–**1i**, enantioselectivities up to 96% and almost enantiopure allylsulfonamides were obtained (selectivities up to 127). Even reactants with the alkene embedded in a cyclohexyl ring, such as **1h** and **1i**, were effective substrates, generating chiral bicyclic structures. Substrates bearing terminal alkenes (**1j** and **1k**) also led to good results, although in this case with slightly lower conversions and enantioselectivities, perhaps because there is less steric encumbrance (**3ja** and **3ka**, up to 90% *ee*). Remarkably, homoallylamide substrates like **1l** and **1m**, in which the chiral center is in the β-position relative to the amine, also participated in the cycloaddition. In this case, as for the formation of **3ka**, *N*-Boc-phenylalanine was a more suitable ligand. Of course, different allylsulfonamides can be combined with different allenes, and therefore a great variety of products can be formed with similar levels of enantioselectivity (see, for instance, **3hc**, with 90% *ee*). As can be deduced from the reaction conditions (Scheme 3), the optimal temperature depends on the type of precursors, likely because of steric factors.

The presence of unsaturations in the cyclic products provides for performing divergent manipulations. For instance, treatment of **3ac** with hydrogen gas in the presence of palladium over carbon led to product **4** in an excellent 91% yield (Scheme 4). Therefore, the hydrogenation is accompanied by an isomerization process which makes it possible to create a new stereocenter in a fully diastereoselective manner. The product **3ac** can also react selectively with ruthenium trichloride to give, in 66% yield, the tetrahydropyridine **5**, exhibiting an α,β-unsaturated motif. Importantly, the triflyl group of the amide can be successfully removed using Red-Al in quantitative yield. In all cases the enantiopurity of the product was intact.

**Scheme 4:**
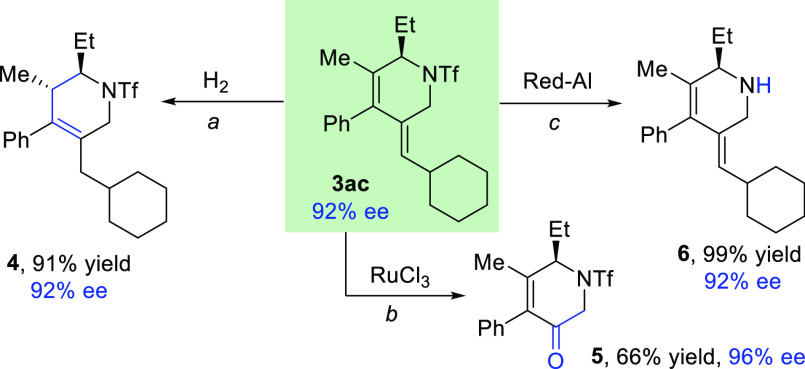
Derivatization of the Chiral Products Conditions: (a) H_2_, Pd/C 10 mol%, AcOEt, rt, 24 h. (b) RuCl_3_ 15 mol%, 3.5 equiv NaIO_4_, H_2_O/CH_3_CN/AcOEt 0.75:1:1, rt, 0.5 h. (c) Red-Al (10 equiv), toluene, 50 °C, 20 h.

As commented before, one of the advantages of this type of kinetic resolution strategies is that the recovered precursor might also be an invaluable platform to produce different types of enantioenriched derivatives. In our case, the chiral allyltriflamides can be easily manipulated owing to the presence of the double bond. For example, compound (*S*)-**1a** (99% *ee*) can be converted into the chiral keto amino product **7** under oxidative conditions (Scheme 5). Amine (*S*)-**1a** can be also alkylated with propargyl bromide, and the resulting enyne can be cyclized to the interesting piperidine **9** using iridium catalysis. This optically active product (99% *ee*), obtained as a single diastereoisomer, exhibits up to four stereocenters.^9^ Finally, we also demonstrated that enantioenriched compounds like (*S*)-**1a** can participate in the (4+2) annulation reaction with allene **2a** under standard conditions, using the d-amino acid ligand derivative, to give the corresponding enantiomer (*S*)-**3aa** (88% yield), which exhibit the same enantiomeric excess as the starting material.

**Scheme 5:**
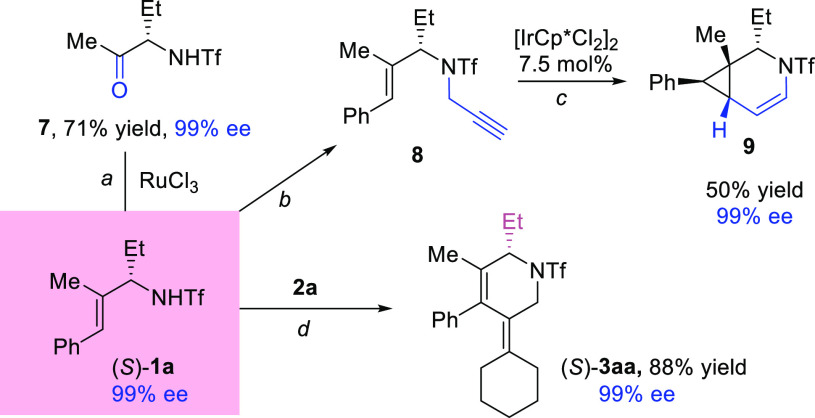
Derivatization of the Chiral Allylamide Starting Materials Conditions: (a) RuCl_3_ 3 mo%, NaIO_4_, CCl_4_/CH_3_CN/H_2_O, 1:1:1.6, rt. (b) K_2_CO_3_, propargyl bromide, 60 °C. (c) 7.5 mol% [IrCp*Cl_2_], dichloroethane, reflux. (d) Optimized conditions are shown in Table 1 using Boc-d-Phe-NHOMe.

Overall, we have discovered a new enantioselective annulation process based on a Pd(II)-catalyzed reaction of allylamine derivatives and allenes and relying on an asymmetric C–H activation step. The reaction allows very efficient kinetic resolutions and provides an unprecedented access to a broad range of enantioenriched piperidines and highly substituted allyl amines. These enantioenriched products can be easily converted into several appealing azacycles and different nitrogenated derivatives. The methodology provides a powerful atom- and step-economical approach to this type of optically active products

## Supporting Information Available

Experimental details and characterization data for all new compounds (PDF)

## Accession Codes

CCDC 2043705 and 2043710 contain the supplementary crystallographic data for this paper. These data can be obtained free of charge via www.ccdc.cam.ac.uk/data_request/cif, or by emailing data_request@ccdc.cam.ac.uk, or by contacting The Cambridge Crystallographic Data Centre, 12 Union Road, Cambridge CB2 1EZ, UK; fax: +44 1223 336033.
