# Supplementary material for: Kinetic Resolution of Allyltriflamides through a Pd-Catalyzed C–H Functionalization with Allenes: Asymmetric Assembly of Tetrahydropyridines
Source: J Am Chem Soc. 2021 Mar 2;143(10):3747–52. doi: 10.1021/jacs.1c01929 (PMC8459456; doi:10.1021/jacs.1c01929)

Supporting Information

Kinetic resolution of allyltriflamides through a Pd-catalyzed C—H functionalization with allenes: asymmetric assembly of tetrahydropyridines

José Manuel González, Borja Cendón, José Luis Mascareñas, Moisés Gulías*

e-mail: moises.gulias@usc.es

Centro Singular de Investigación en Química Biológica y Materiales Moleculares (CIQUS) and Departamento de Química Orgánica, Universidad de Santiago de Compostela, 15782 Santiago de Compostela, Spain.

Table of Contents

1. General Experimental Information 2

2. Synthesis of Starting Materials and Chiral Ligands 3

2.1. Synthesis of Allyltriflamides 3

2.2. Synthesis of Allenes 14

2.3. Synthesis of Chiral Ligands 15

3. Kinetic Resolution via Palladium(II)-Catalyzed Alkenyl C–H Activation 16

4. Synthetic Manipulations of Cycloadducts and Enantioenriched Starting Materials 65

4.1. Cycloadducts 65

4.2. Enantioenriched Starting Materials 72

5. References 78

6. NMR Spectra 79

1. General Experimental Information

Reactions were conducted in dry solvents under Argon unless otherwise stated. Dry solvents were obtained from Across Organics, Extra Dry over Molecular Sieves, and used without further purification. $Pd(OAc)_2$ (98%) [3375-31-1] was obtained from Strem. All other chemicals were purchased from Sigma-Aldrich, Acros Organics, Alfa Aesar, Fluorochem, TCI Chemicals or Abcr and they were used as received. All palladium-catalyzed reactions were carried without precautions to elude moisture or oxygen.

The abbreviation "rt" refers to reactions carried out at a temperature between 21-25°C. Reaction mixtures were stirred using Teflon-coated magnetic stir bars. Thin layer chromatography (TLC) was carried out on pre-coated silica gel F_{254} plates with visualization under UV light or by dipping the plate into solutions of *p*-anisaldehyde, ninhydrin, phosphomolybdic acid or potassium permanganate solutions followed by heating. Column chromatography was performed on silica gel (40-60 µm) unless otherwise stated.

NMR data was collected on Varian Mercury 300 MHz or Bruker AVIII 500 MHz spectrometers. Chemical shifts are given in ppm (δ) and are referenced to the residual $CDCl_3$ solvent peak at 7.26 ppm (^{1}H-NMR) and 77.16 ppm (^{13}C-NMR). Conventional one-dimensional (1D) ^{1}H-NMR, ^{19}F-NMR, ^{13}C{^{1}H}-NMR, Distortionless Enhancement by Polarization Transfer Spectra (DEPT) and two-dimensional (2D) ^{1}H-^{1}H Correlation Spectroscopy (COSY), ^{1}H-^{1}H Nuclear Overhauser Effect Spectroscopy (NOESY), ^{1}H-^{13}C heteronuclear single quantum coherence (HSQC),^{1}H-^{13}C Heteronuclear Multiple-Bond Correlation Spectroscopy (HMBC) experiments were recorded at room temperature under routine conditions. NMR data was analysed using MestReNova NMR data processing software (http://mestrelab.com/). High Resolution Mass Spectra (HRMS) were performed at the CACTUS facility of the University of Santiago de Compostela on a Bruker micrOTOF spectrometer.

Enantiomeric ratios (*er*) were determined on an Agilent HPLC 1100 Series or on a Jasco SFC 4000 series using commercially available chiral columns. All racemic products were prepared under the same procedure than the chiral products although with the employment of a racemic mono-protected amino acid ligand.

X-ray crystallographic analysis of **3ba** and **1b** was performed at the CACTUS facility of the University of Santiago de Compostela and the absolute stereochemistry of all compounds was assigned by analogy to **3ba** and **1b**.

2. Synthesis of Starting Materials and Chiral Ligands

2.1. Synthesis of Allyltriflamides

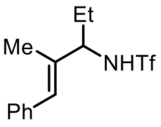

rac-**1a**

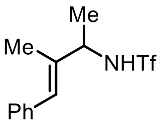

rac-**1b**

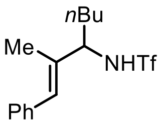

rac-**1c**

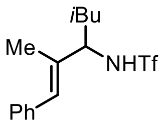

rac-**1d**

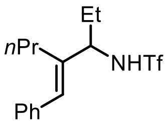

rac-**1e**

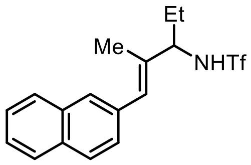

rac-**1f**

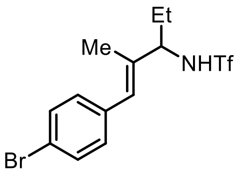

rac-**1g**

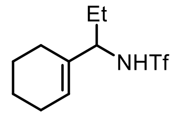

rac-**1h**

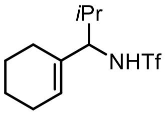

rac-**1i**

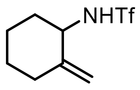

rac-**1j**

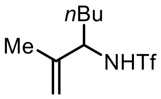

rac-**1k**

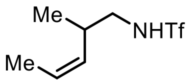

rac-**1l**

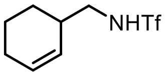

rac-**1m**

General procedure A: preparation of α-substituted allyltriflamides substrates *rac*-1a to *rac*-1g

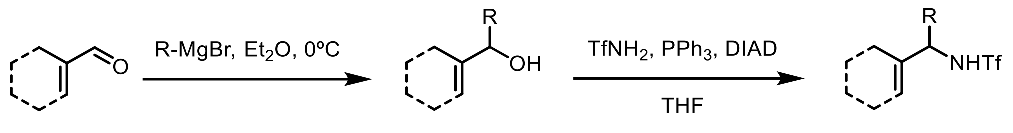

rac-**1a** to *rac*-**1g**

Grignard reagent solution (1.5 eq) was added to an ice cooled solution of α,β-unsaturated aldehyde (1 eq) in dry Et_2O at 0°C and stirred until the starting material had been completely consumed as judged by TLC analysis (around 30 min). The reaction was quenched with saturated NH_4Cl aqueous solution and extracted with Et_2O. The organic layer was washed with brine, dried over Na_2SO_4 and concentrated under reduced pressure. The crude allylic alcohol was used in the next synthetic step without further purification.

The obtained allylic alcohol (1 eq) was dissolved in dry THF (0.1M) and the mixture was cooled at 0°C. Then, PPh_3 (1.1 eq) and trifluoromethanesulfonamide (1.1 eq) were added followed by dropwise addition of DIAD (98%, 1.3 eq). After stirring over night at room temperature, the solvent was removed in vacuum and the crude reaction mixture was purified by flash column chromatography (silica gel, hexane:Et_2O 95:5 to 80:20) to obtain the corresponding allyltriflamides *rac*-**1a** to *rac*-**1g**.

(*E*)-1,1,1-trifluoro-*N*-(2-methyl-1-phenylpent-1-en-3-yl)methanesulfonamide (*rac*-1a)

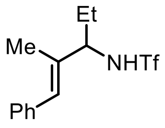

rac-**1a**

rac-**1a** obtained as a yellow pasty solid upon freezing (1.4 g, 40% yield). **^{1}H NMR** (300 MHz, $CDCl_3$) δ, ppm: 7.43 – 7.34 (m, 2H), 7.33 – 7.24 (m, 3H), 6.52 (s, 1H), 5.37 (brs, 1H), 4.08 (t, *J* = 7.4 Hz, 1H), 1.86 (s, 3H), 1.85 – 1.73 (m, 2H), 1.03 (t, *J* = 7.4 Hz, 3H). **^{19}F NMR** (282 MHz, $CDCl_3$) δ, ppm: -77.93. **^{13}C NMR** (75 MHz, $CDCl_3$) δ, ppm: 136.9 (C), 135.0 (C), 129.1 (CH), 128.9 (CH), 128.4 (CH), 127.1 (CH), 119.6 (q, *J* = 321.1 Hz, C), 65.2 (CH), 27.5 (CH_2), 13.2 (CH_3), 10.6 (CH_3). **HRMS** (APCI, $[M+H]^+$) *m/z* calculated for $C_{13}H_{16}F_3NO_2S$: 307.0848; found 307.0847.

(*E*)-1,1,1-trifluoro-*N*-(3-methyl-4-phenylbut-3-en-2-yl)methanesulfonamide (*rac*-1b)

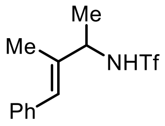

rac-**1b**

rac-**1b** obtained as a white solid upon freezing (1.1 g, 40% yield). **^{1}H NMR** (300 MHz, $CDCl_3$) δ, ppm: 7.43 – 7.35 (m, 2H), 7.32 – 7.24 (m, 3H), 6.55 (s, 1H), 5.36 (d, *J* = 8.2 Hz, 1H), 4.40 – 4.28 (m, 1H), 1.91 (s, 3H), 1.51 (d, *J* = 6.8 Hz, 3H). **^{19}F NMR** (282 MHz, $CDCl_3$) δ, ppm: -78.02 (s). **^{13}C NMR** (75 MHz, $CDCl_3$) δ, ppm: 136.8 (C), 136.6 (C), 129.1 (CH), 128.3 (CH), 127.5 (CH), 127.1 (CH), 119.7 (q, *J* = 321.0 Hz, C), 58.9 (CH), 21.1 (CH_3), 13.9 (CH_3). **HRMS** (APCI, $[M]^+$) *m/z* calculated for $C_{12}H_{14}F_3NO_2S$: 293.0692; found 293.0690.

(*E*)-1,1,1-trifluoro-*N*-(2-methyl-1-phenylhept-1-en-3-yl)methanesulfonamide (*rac*-1c)

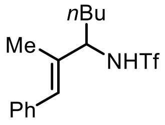

rac-**1c**

rac-**1c** obtained as a white solid (0.91 g, 37% yield). **^{1}H NMR** (300 MHz, $CDCl_3$) δ, ppm: 7.41 – 7.34 (m, 2H), 7.31 – 7.24 (m, 3H), 6.51 (s, 1H), 5.33 (d, *J* = 8.4 Hz, 1H), 4.15 (q, *J* = 7.7 Hz, 1H), 1.85 (d, *J* = 1.3 Hz, 3H), 1.78 – 1.69 (m, 2H), 1.46 – 1.35 (m, 4H), 0.96 (t, *J* = 6.8 Hz, 3H). **^{19}F NMR** (282 MHz, $CDCl_3$) δ, ppm: -77.95 (s). **^{13}C NMR** (75 MHz, $CDCl_3$) δ, ppm: 136.9 (C), 135.3 (C), 129.1 (CH), 128.8 (CH), 128.4 (CH), 127.1 (CH), 119.6 (q, *J* = 321.2 Hz, C), 63.8 (CH), 34.1 (CH_2), 28.1 (CH_2), 22.3 (CH_2), 14.0 (CH_3), 13.1 (CH_3). **HRMS** (APCI, $[M]^+$) *m/z* calculated for $C_{15}H_{20}F_3NO_2S$: 335.1161; found 335.1160.

(*E*)-*N*-(2,5-dimethyl-1-phenylhex-1-en-3-yl)-1,1,1-trifluoromethanesulfonamide (*rac*-1d)

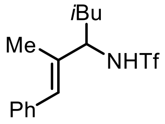

rac-**1d**

rac-**1d** obtained as a yellow solid upon freezing (0.35 g, 36% yield). **^{1}H NMR** (300 MHz, $CDCl_3$) δ, ppm: 7.41 – 7.34 (m, 2H), 7.30 – 7.24 (m, 3H), 6.53 (s, 1H), 5.32 (d, *J* = 8.4 Hz, 1H), 4.24 (q, *J* = 7.7 Hz, 1H), 1.85 (d, *J* = 1.3 Hz, 3H), 1.75 – 1.64 (m, 1H), 1.63 – 1.56 (m, 2H), 1.03 – 0.99 (m, 6H). **^{19}F NMR** (282 MHz, $CDCl_3$) δ, ppm: -77.96 (s). **^{13}C NMR** (75 MHz, $CDCl_3$) δ, ppm: 136.9 (C), 135.4 (C), 129.1 (CH), 128.8 (CH), 128.4 (CH), 127.1 (CH), 119.6 (q, *J* = 321.2, C), 62.2 (CH), 43.6 (CH_2), 24.8 (CH), 22.5 (CH_3), 22.3 (CH_3), 13.0 (CH_3). **HRMS** (APCI, $[M]^+$) *m/z* calculated for $C_{15}H_{20}F_3NO_2S$: 335.1161; found 335.1159.

(*E*)-*N*-(4-benzylideneheptan-3-yl)-1,1,1-trifluoromethanesulfonamide (*rac*-1e)

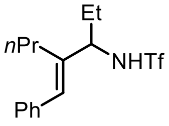

rac-**1e**

rac-**1e** obtained as a white solid upon freezing (0.25 g, 33% yield) from the corresponding aldehyde, synthesized according to the literature.[1] **^{1}H NMR** (300 MHz, $CDCl_3$) δ, ppm: 7.41 – 7.34 (m, 2H), 7.31 – 7.22 (m, 3H), 6.49 (s, 1H), 5.29 (d, *J* = 8.9 Hz, 1H), 4.09 (q, *J* = 7.5 Hz, 1H), 2.38 – 2.25 (m, 1H), 2.22 – 2.10 (m, 1H), 1.95 – 1.82 (m, 1H), 1.82 – 1.69 (m, 1H), 1.60 – 1.44 (m, 2H), 1.04 (t, *J* = 7.4 Hz, 3H), 0.94 (t, *J* = 7.3 Hz, 3H). **^{19}F NMR** (282 MHz, $CDCl_3$) δ, ppm: -77.43 (s). **^{13}C NMR** (75 MHz, $CDCl_3$) δ, ppm: 140.8 (C), 137.0 (C), 128.7 (CH), 128.5 (CH), 127.7 (CH), 127.1 (CH), 119.7 (q, *J* = 321.0 Hz, C), 62.7 (CH), 31.3 (CH_2), 28.5 (CH_2), 22.1 (CH_2), 14.4 (CH_3), 10.6 (CH_3). **HRMS** (APCI, $[M-1]^-$) *m/z* calculated for $C_{15}H_{19}F_3NO_2S$: 334.1094; found 334.1106.

(*E*)-1,1,1-trifluoro-*N*-(2-methyl-1-(naphthalen-2-yl)pent-1-en-3-yl)methanesulfonamide (*rac*-1f)

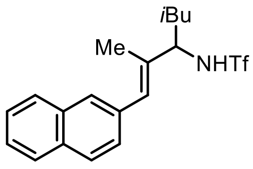

rac-**1f**

rac-**1f** obtained as a white solid (0.34 g, 39% yield) from the corresponding aldehyde, synthesized according to the literature.[1] **^{1}H NMR** (300 MHz, $CDCl_3$) δ, ppm: 7.88 – 7.79 (m, 3H), 7.71 (s, 1H), 7.53 – 7.45 (m, 2H), 7.39 (d, *J* = 8.5 Hz, 1H), 6.65 (s, 1H), 5.42 (d, *J* = 8.5 Hz, 1H), 4.12 (q, *J* = 7.7 Hz, 1H), 1.90 (s, 3H), 1.79 (p, *J* = 7.3 Hz, 2H), 1.04 (t, *J* = 7.4 Hz, 3H). **^{19}F NMR** (282 MHz, $CDCl_3$) δ, ppm: -77.47 (s). **^{13}C NMR** (75 MHz, $CDCl_3$) δ, ppm: 135.5 (C), 134.4 (C), 133.3 (C), 132.4 (C), 128.9 (CH), 128.0 (CH), 127.9 (CH), 127.9 (CH), 127.7 (CH), 127.2 (CH), 126.3 (CH), 126.1 (CH), 119.7 (q, *J* = 321.0 Hz, C), 65.2 (CH), 27.5 (CH_2), 13.3 (CH_3), 10.6 (CH_3). **HRMS** (APPI, $[M-1]^-$) *m/z* calculated for $C_{17}H_{17}F_3NO_2S$: 356.0938; found 356.0942.

(*E*)-*N*-(1-(4-bromophenyl)-2-methylpent-1-en-3-yl)-1,1,1-trifluoromethanesulfonamide (*rac*-1g)

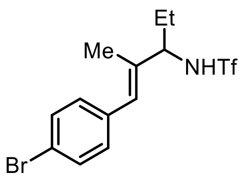

rac-**1g**

rac-**1g** obtained as a yellowish solid (0.39 g, 40% yield) from the corresponding aldehyde, synthesized according to the literature.[1] **^{1}H NMR** (300 MHz, $CDCl_3$) δ, ppm: 7.46 (d, *J* = 8.4 Hz, 2H), 7.10 (d, *J* = 8.3 Hz, 2H), 6.41 (s, 1H), 5.65 (d, *J* = 8.4 Hz, 1H), 4.02 (q, *J* = 7.6 Hz, 1H), 1.81 – 1.69 (m, 5H), 0.98 (t, *J* = 7.4 Hz, 3H). **^{19}F NMR** (282 MHz, $CDCl_3$) δ, ppm: -76.27 (s). **^{13}C NMR** (75 MHz, $CDCl_3$) δ, ppm: 136.1 (C), 135.8 (C), 131.4 (CH), 130.6 (CH), 127.6 (CH), 120.9 (C), 119.6 (q, *J* = 321.0, C), 65.1 (CH), 27.3 (CH_2), 13.1 (CH_3), 10.6 (CH_3). **HRMS** (APCI, $[M-1]^-$) *m/z* calculated for $C_{13}H_{14}BrF_3NO_2S$: 383.9886; found 383.9893.

General procedure B: preparation of α-substituted allyltriflamides substrates *rac*-1h, *rac*-1i and *rac*-1k

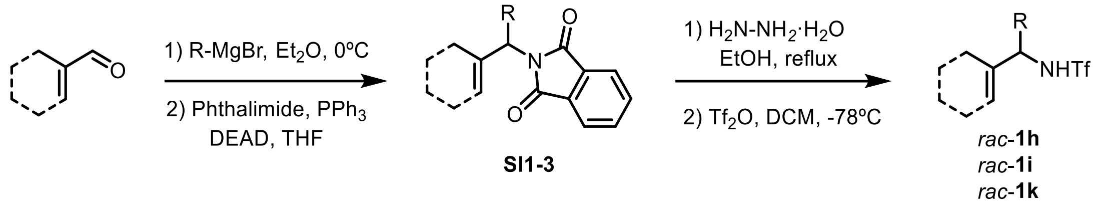

Grignard reagent solution (1.5 eq) was added to an ice cooled solution of α,β-unsaturated aldehyde (1 eq) in dry Et_2O at 0ºC and stirred until the starting material had been completely consumed as judged by TLC analysis (around 30 min). The reaction was quenched with saturated NH_4Cl aqueous solution and extracted with Et_2O. The organic layer was washed with brine, dried over Na_2SO_4 and concentrated under reduced pressure. The crude allylic alcohol was used in the next synthetic step without further purification.

The corresponding allylic alcohol (1 eq) was dissolved in dry THF (0.25M). Then, PPh_3 (1.5 eq), and phthalimide (1 eq) were added. The mixture was cooled at 0°C and DEAD (40% in toluene, 1.5 eq) was added dropwise. After stirring over night at room temperature, the solvent was removed in vacuum. The crude reaction mixture was purified by flash column chromatography (silica gel, hexane:AcOEt 95:5) to obtain the corresponding phthalimide **SI1**, **SI2** and **SI3**. Note: in almost every case a second flash column chromatography was needed to obtain the pure phthalimide.

To a solution of the corresponding phthalimide (1 eq) in EtOH (0.1 M) was added hydrazine hydrate (6 eq) dropwise and the resulting mixture was stirred at reflux until the starting material had been completely consumed as judged by TLC analysis (around 2h). The formed pasty precipitate was then filtered through a plug of celite, washed with abundant Et_2O and the solvent was evaporated under reduced pressure. The desired amine was used in the next synthetic step without further purification.

The amine (1 eq) was dissolved in dry DCM (0.5 M), and the reaction mixture was cooled to -78ºC. Triethylamine (3 eq) was added and the reaction mixture was stirred -78ºC for 5 min. Trifluoromethanesulfonic anhydride (1.05 eq) was then added dropwise and the reaction mixture was stirred at -78ºC for 2 hours, before quenching with ice. The organic layer was separated, and the aqueous layer was extracted twice with DCM. The combined organics were washed with brine, dried over Na_2SO_4, filtered, and then concentrated in vacuo. The products were purified by flash column chromatography (silica gel, hexane:Et_2O 95:5 to 80:20).

2-(1-(cyclohex-1-en-1-yl)propyl)isoindoline-1,3-dione (SI1)

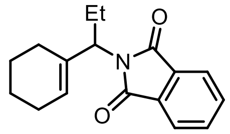

SI1

SI1 obtained as a yellowish oil (4 g, 30% yield). **^{1}H NMR** (300 MHz, $CDCl_3$) δ, ppm: 7.80 (dd, *J* = 5.3, 3.2 Hz, 2H), 7.68 (dd, *J* = 5.5, 3.0 Hz, 2H), 5.80 – 5.71 (m, 1H), 4.55 – 4.44 (m, 1H), 2.33 – 2.14 (m, 1H), 2.10 – 1.90 (m, 5H), 1.64 – 1.44 (m, 4H), 0.86 (t, *J* = 7.4 Hz, 3H). **^{13}C NMR** (75 MHz, $CDCl_3$) δ, ppm: 168.8 (C), 135.1 (C), 133.9 (CH), 132.0 (C), 124.5 (CH), 123.2 (CH), 58.5 (CH), 26.4 (CH_2), 25.2 (CH_2), 22.8 (CH_2), 22.3 (CH_2), 11.5 (CH_3). **HRMS** (APCI, $[M+H]^+$) *m/z* calculated for $C_{17}H_{20}NO_2$: 270.1489; found 270.1488.

2-(1-(cyclohex-1-en-1-yl)-2-methylpropyl)isoindoline-1,3-dione (SI2)

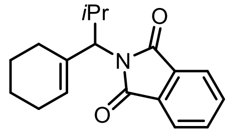

SI2

SI2 obtained as a white solid (700 mg, 13% yield). **^{1}H NMR** (300 MHz, $CDCl_3$) δ, ppm: 7.79 (dd, *J* = 5.5, 3.0 Hz, 2H), 7.67 (dd, *J* = 5.4, 3.1 Hz, 2H), 5.86 (s, 1H), 4.13 (d, *J* = 11.5 Hz, 1H), 2.93 (tp, *J* = 13.1, 6.5 Hz, 1H), 2.18 – 1.90 (m, 4H), 1.64 – 1.41 (m, 4H), 0.92 (d, *J* = 6.6 Hz, 3H), 0.82 (d, *J* = 6.5 Hz, 3H). **^{13}C NMR** (75 MHz, $CDCl_3$) δ, ppm: 169.0 (C), 135.3 (C), 133.9 (CH), 132.0 (C), 127.7 (CH), 123.2 (CH), 65.4 (CH), 26.0 (CH), 25.8 (CH_2), 25.3 (CH_2), 22.9 (CH_2), 22.3 (CH_2). 20.7 (CH_3), 20.2 (CH_3). **HRMS** (APCI, $[M+H]^+$) *m/z* calculated for $C_{18}H_{22}NO_2$: 284.1645; found 284.1644.

2-(2-methylhept-1-en-3-yl)isoindoline-1,3-dione (SI3)

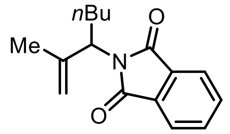

SI3

SI3 obtained as a yellowish oil (950 mg, 50% yield). **^{1}H NMR** (300 MHz, $CDCl_3$) δ, ppm: 7.82 (dd, *J* = 5.1, 3.1 Hz, 2H), 7.70 (dd, *J* = 5.2, 2.8 Hz, 2H), 5.03 (s, 1H), 4.96 (s, 1H), 4.66 (dd, J = 10.2, 5.5 Hz, 1H), 2.38 – 2.20 (m, 1H), 2.06 – 1.91 (m, 1H), 1.75 (s, 3H), 1.41 – 1.15 (m, 4H), 0.85 (t, *J* = 7.0 Hz, 3H). **^{13}C NMR** (75 MHz, $CDCl_3$) δ, ppm: 168.6 (C), 142.7 (C), 134.0 (CH), 131.9 (C), 123.3 (CH), 113.2 (CH_2), 55.9 (CH), 29.0 (CH_2), 22.4 (CH_2), 20.8 (CH_3), 14.1 (CH_3). **HRMS** (APCI, $[M+H]^+$) *m/z* calculated for $C_{16}H_{20}NO_2$: 258.1489; found 258.1480.

***N*-(1-(cyclohex-1-en-1-yl)propyl)-1,1,1-trifluoromethanesulfonamide (*rac*-1h)**

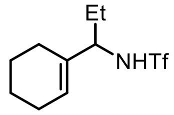

rac-**1h**

rac-**1h** obtained as a yellow oil (390 mg, 67% yield). **^{1}H NMR** (300 MHz, $CDCl_3$) δ, ppm: 5.67 (s, 1H), 3.82 (t, *J* = 7.1 Hz, 1H), 2.10 – 1.99 (m, 2H), 1.96 – 1.86 (m, 2H), 1.72 – 1.52 (m, 6H), 0.90 (t, *J* = 7.3 Hz, 3H). **^{19}F NMR** (282 MHz, $CDCl_3$) δ, ppm: -78.01. **^{13}C NMR** (75 MHz, $CDCl_3$) δ, ppm: 135.3 (C), 126.0 (CH), 119.8 (q, *J* = 321.4 Hz, C), 63.7 (CH), 27.4 (CH_2), 25.1 (CH_2), 24.1 (CH_2), 22.6 (CH_2), 22.4 (CH_2), 10.3 (CH_3). **HRMS** (APCI, $[M+H]^+$) *m/z* calculated for $C_{10}H_{17}F_3NO_2S$: 272.0927; found 272.0925.

N-(1-(cyclohex-1-en-1-yl)-2-methylpropyl)-1,1,1-trifluoromethanesulfonamide (*rac*-1i)

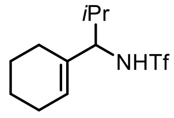

rac-**1i**

rac-**1i** obtained as a yellowish solid (92 mg, 32% yield). **^{1}H NMR** (300 MHz, $CDCl_3$) δ, ppm: 5.61 (s, 1H), 5.37 (brs, 1H), 3.54 (d, *J* = 8.6 Hz, 1H), 2.11 – 1.98 (m, 2H), 1.95 – 1.71 (m, 3H), 1.71 – 1.50 (m, 4H), 0.98 (d, *J* = 6.7 Hz, 3H), 0.88 (d, *J* = 6.7 Hz, 3H). **^{19}F NMR** (282 MHz, $CDCl_3$) δ, ppm: -77.84. **^{13}C NMR** (75 MHz, $CDCl_3$) δ, ppm: 134.9 (C), 126.3 (CH), 119.6 (q, *J* = 321.2 Hz, C), 68.6 (CH), 30.6 (CH), 25.0 (CH_2), 24.1 (CH_2), 22.6 (CH_2), 22.4 (CH_2), 19.9 (CH_3), 18.7 (CH_3). **HRMS** (APCI, $[M+H]^+$) *m/z* calculated for $C_{11}H_{19}F_3NO_2S$: 286.1083; found 286.1086.

1,1,1-trifluoro-N-(2-methylhept-1-en-3-yl)methanesulfonamide (*rac*-1k)

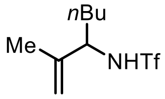

rac-**1k**

rac-**1k** obtained as a yellow oil (150 mg, 74% yield). **^{1}H NMR** (300 MHz, $CDCl_3$) δ, ppm: 5.26 (brs, 1H), 4.93 (s, 2H), 3.98 (t, J = 7.1 Hz, 1H), 1.72 (s, 3H), 1.69 – 1.54 (m, 2H), 1.40 – 1.22 (m, 4H), 0.90 (t, J = 6.8 Hz, 2H). **^{19}F NMR** (282 MHz, $CDCl_3$) δ, ppm: -78.12. **^{13}C NMR** (75 MHz, $CDCl_3$) δ, ppm: 142.8 (C), 119.6 (q, *J* = 320.9 Hz, C), 113.8 (CH_2), 61.5 (CH), 34.0 (CH_2), 27.8 (CH_2), 22.3 (CH_2), 17.9 (CH_3), 13.9 (CH_3). **HRMS** (APCI, $[M+H]^+$) *m/z* calculated for $C_9H_{17}F_3NO_2S$: 260.0927; found 260.0924.

Preparation of allyltriflamide substrate *rac*-1j

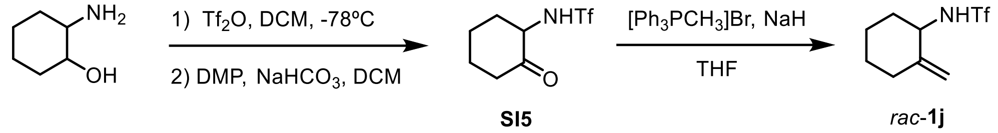

2-Aminocyclohexanol (1 eq) was dissolved in dry DCM (0.2 M), and the reaction mixture was cooled to -78°C. Triethylamine (1.5 eq) was added and the reaction mixture was stirred -78°C for 5 min. Trifluoromethanesulfonic anhydride (1 eq) was then added dropwise and the reaction mixture was stirred at -78°C for 2 hours, before quenching with ice. The organic layer was separated, and the aqueous layer was extracted twice with DCM. The combined organics were washed with brine, dried over Na_2SO_4, filtered, and then concentrated in vacuo. The alcohol product **SI4** was purified by flash column chromatography (silica gel, hexane:AcOEt 80:20 to 50:50).

To a solution of the alcohol previously prepared (1 eq) in DCM (0.1M) at 0°C was added $NaHCO_3$ (2.5 eq) followed by Dess-Martin periodinane (DMP) (1.5 eq) and the reaction was stirred for 3 hours, until the starting material had been completely consumed as judged by TLC analysis. The reaction was quenched with a saturated $NaHCO_3$ aqueous solution, the aqueous layer was extracted with DCM and the organic layers were washed with brine, dried over Na_2SO_4, filtered, and evaporated. The residue was purified by column chromatography (silica gel, hexane:AcOEt 95:5 to 80:20) to obtain the ketone **SI5**.

To a solution of sodium hydride (4 eq) in THF (0.25M) under Ar atmosphere was added methyltriphenylphosphonium bromide (2 eq) at 0°C. The reaction mixture was stirred for 1 hour at that temperature. Then ketone **SI5** (1 eq) was added at 0°C and the reaction was stirred for 16 hours at room temperature and quenched with saturated NH_4Cl aqueous solution. The solvent was removed in vacuo and the resulting mixture was extracted with diethyl ether. The combined organics were washed with brine, dried over Na_2SO_4, filtered, and then concentrated in vacuo. The allyl triflamide *rac*-**1j** was purified by flash column chromatography (silica gel, hexane:AcOEt 95:5 to 80:20).

1,1,1-trifluoro-*N*-(2-hydroxycyclohexyl)methanesulfonamide (SI4)

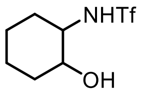

SI4

SI4 obtained as a white solid (1.8 g, 43% yield). **^{1}H NMR** (300 MHz, $CDCl_3$) δ, ppm: 3.42 – 3.31 (m, 1H), 3.30 – 3.19 (m, 1H), 2.21 – 2.02 (m, 2H), 1.82 – 1.66 (m, 2H), 1.44 – 1.20 (m, 4H). **^{19}F NMR** (282 MHz, $CDCl_3$) δ, ppm: -77.96. **^{13}C NMR** (75 MHz, $CDCl_3$) δ, ppm: 119.70 (q, *J* = 320.8 Hz, C), 73.3 (CH), 61.5 (CH), 34.2 (CH_2), 32.6 (CH_2), 24.7 (CH_2), 24.1 (CH_2). **HRMS** (APCI, $[M+H]^+$) *m/z* calculated for $C_7H_{13}F_3NO_3S$: 248.0563; found 248.0565.

1,1,1-trifluoro-*N*-(2-oxocyclohexyl)methanesulfonamide (SI5)

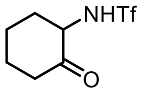

SI5

SI5 obtained as a white solid (410 mg, 83% yield). **^{1}H NMR** (300 MHz, $CDCl_3$) δ, ppm: 4.21 (dd, *J* = 12.3, 6.2 Hz, 1H), 2.69 – 2.53 (m, 2H), 2.41 (td, *J* = 13.5, 6.3 Hz, 1H), 2.22 – 2.10 (m, 1H), 2.00 – 1.89 (m, 1H), 1.87 – 1.54 (m, 3H). **^{19}F NMR** (282 MHz, $CDCl_3$) δ, ppm: -78.05. **^{13}C NMR** (75 MHz, $CDCl_3$) δ, ppm: 205.0 (C), 119.6 (q, *J* = 320.8 Hz, C), 61.9 (CH), 40.9 (CH_2), 37.1 (CH_2), 27.6 (CH_2), 24.0 (CH_2). **HRMS** (APCI, $[M+H]^+$) *m/z* calculated for $C_7H_{11}F_3NO_3S$: 246.0406; found 246.0405.

1,1,1-trifluoro-*N*-(2-methylenecyclohexyl)methanesulfonamide (*rac*-1j)

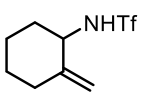

rac-**1j**

rac-**1j** obtained as a white solid (120 mg, 80% yield). **^{1}H NMR** (300 MHz, $CDCl_3$) δ, ppm: 5.09 (brs, 1H), 4.88 (d, *J* = 5.2 Hz, 2H), 4.08 – 3.95 (m, 1H), 2.50 – 2.36 (m, 1H), 2.18 – 2.00 (m, 2H), 1.89 – 1.66 (m, 2H), 1.63 – 1.23 (m, 3H). **^{19}F NMR** (282 MHz, $CDCl_3$) δ, ppm: -78.35. **^{13}C NMR** (75 MHz, $CDCl_3$) δ, ppm: 147.0 (C), 119.67 (q, *J* = 320.5 Hz, C), 107.6 (CH_2), 58.2 (CH), 36.4 (CH_2), 34.5 (CH_2), 27.1 (CH_2), 24.8 (CH_2). **HRMS** (APCI, $[M+H]^+$) *m/z* calculated for $C_8H_{13}F_3NO_2S$: 244.0614; found 244.061.

Preparation of homoallyltriflamide substrate *rac*-1l

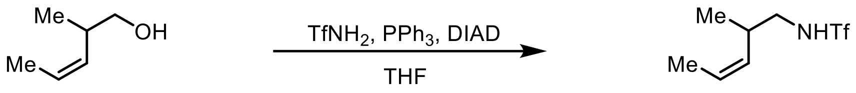

rac-**1l**

The corresponding homoallylic alcohol (1 eq), obtained according to the literature,[2] was dissolved in dry THF (0.1 M) and the mixture was cooled to 0°C. Then, PPh_3 (1.1 eq) and trifluoromethanesulfonamide (1.1 eq) were added followed by dropwise addition of DIAD (98%, 1.3 eq). After stirring overnight at room temperature, the solvent was removed in vacuo and the crude reaction mixture was purified by column chromatography on silica gel (hexane/Et_2O, 98:2 to 95:5) to obtain the corresponding homoallyltriflamide *rac*-**1l**.

(*Z*)-1,1,1-trifluoro-*N*-(2-methylpent-3-en-1-yl)methanesulfonamide (*rac*-1l)

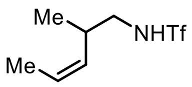

rac-**1l**
Z:E > 10:1

rac-**1l** obtained as a colourless oil (949 mg, 52% yield). **^{1}H NMR** (300 MHz, $CDCl_3$) δ, ppm: 5.64 (dq, *J* = 11.0, 6.9 Hz, 1H), 5.14 – 4.98 (m, 2H), 3.33 – 3.21 (m, 1H), 2.97 (ddd, *J* = 13.0, 9.2, 4.0 Hz, 1H), 2.86 – 2.70 (m, 1H), 1.66 (dd, *J* = 6.9, 1.6 Hz, 3H), 0.99 (d, *J* = 6.7 Hz, 3H). **^{19}F NMR** (282 MHz, $CDCl_3$) δ, ppm: -77.47 (s). **^{13}C NMR** (75 MHz, $CDCl_3$) δ, ppm: 131.6 (CH), 127.6 (CH), 119.8 (q, *J* = 321.0 Hz, C), 49.7 (CH_2), 32.5 (CH), 17.9 (CH_3), 13.2 (CH_3). **HRMS** (APCI, $[M-1]^-$) *m/z* calculated for $C_7H_{11}F_3NO_2S$: 230.0468; found 230.0467.

Preparation of homoallyltriflamide substrate *rac*-1m

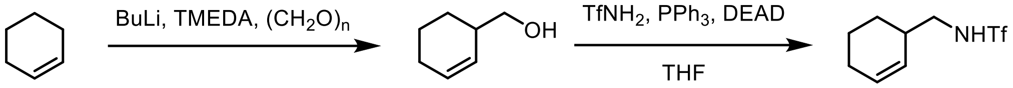

rac-**1m**

The corresponding homoallylic alcohol (1 eq), obtained according to the literature,[3] was dissolved in dry THF (0.25M). Then, trifluoromethanesulfonamide (2 eq) and triphenylphosphine (2 eq) were added. The mixture was cooled at 0°C and DEAD (40% in toluene, 2 eq) was added dropwise. After stirring over night at room temperature, the solvent was removed in vacuum. The crude reaction mixture was purified by flash column chromatography (silica gel, hexane/Et_2O 95:5 to 80:20) to obtain the homoallyltriflamide *rac*-**1m**.

***N*-(cyclohex-2-en-1-ylmethyl)-1,1,1-trifluoromethanesulfonamide (*rac*-1m)**

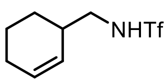

rac-**1m**

rac-**1m** obtained as a yellowish oil (450 mg, 55% yield). **^{1}H NMR** (300 MHz, $CDCl_3$) δ, ppm: 5.93 – 5.83 (m, 1H), 5.49 (d, *J* = 10.1 Hz, 1H), 5.06 (brs, 1H), 3.32 – 3.15 (m, 2H), 2.43 – 2.31 (m, 1H), 2.07 – 1.96 (m, 2H), 1.88 – 1.69 (m, 2H), 1.65 – 1.48 (m, 1H), 1.44 – 1.29 (m, 1H). **^{19}F NMR** (282 MHz, $CDCl_3$) δ, ppm: -77.68. **^{19}F NMR** (282 MHz, $CDCl_3$) δ, ppm: -77.68. **^{13}C NMR** (75 MHz, $CDCl_3$) δ, ppm: 131.5 (CH), 126.3 (CH), 119.8 (q, *J* = 321.2 Hz, C), 49.1 (CH_2), 35.7 (CH), 26.1 (CH_2), 25.1 (CH_2), 20.7 (CH_2). **HRMS** (APCI, $[M+H]^+$) *m/z* calculated for $C_8H_{13}F_3NO_2S$: 244.0614; found 244.0613.

2.2. Synthesis of Allenes

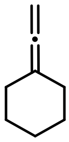

2a

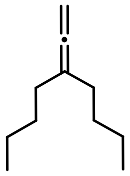

2b

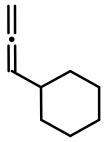

2c

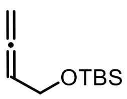

2d

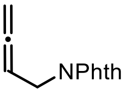

2e

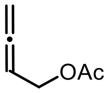

2f

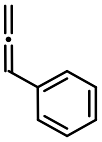

2g

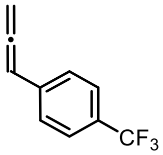

2h

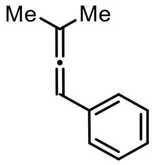

2i

Allene **2a** (vinylidenecyclohexane) was purchased from Sigma-Aldrich or prepared following a procedure reported in the literature.[4a] Allene **2c** (propa-1,2-dien-1-ylcyclohexane) were purchased from Sigma-Aldrich. Allene **2b** (5-vinylidenenonane),[4a] allene **2d** ((buta-2,3-dien-1-yloxy)(tert-butyl)dimethylsilane),[4b] allene **2e** (2-(buta-2,3-dien-1-yl)isoindoline-1,3-dione),[4a] allene **2f** (buta-2,3-dien-1-yl acetate),[4c] allene **2g** (propa-1,2-dien-1-ylbenzene),[4d] allene **2h** (1-(propa-1,2-dien-1-yl)-4-(trifluoromethyl)benzene)[4d] and allene **2i** ((3-methylbuta-1,2-dien-1-yl)benzene)[4e] were prepared according to procedures described in the literature. Spectral data recorded agreed with the previously reported.

2.3. Synthesis of Chiral Ligands

All mono protected amino acids ligands were purchased from commercial sources or synthesized according to literature procedures. Boc-*L*-Phe-NHOMe ligand was synthesized following a procedure reported in the literature with a slight modification.[5]

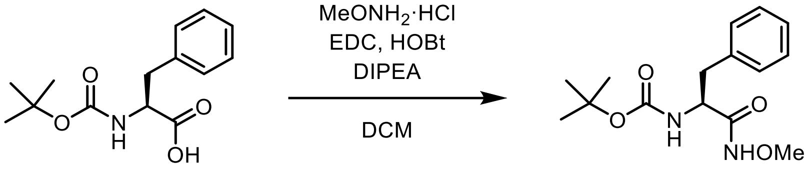

Boc-*L*-Phe-OH **Boc-*L*-Phe-NHOMe**

O-Methylhydroxylamine hydrochloride salt (1.5 eq) and N,N-diisopropylethylamine (DIPEA, 1.5 eq) were added to a cooled (0°C) solution of Boc-*L*-Phe-OH (1 eq), HOBt (1.1 eq) and EDC (1.1 eq) in DCM (0.25 M). After being stirred overnight at room temperature, the reaction mixture was poured into H_2O. The organic layer was separated, dried over anhydrous Na_2SO_4, filtered and concentrated under vacuum. The residue was purified by column chromatography on silica gel (silica gel, DCM/MeOH 99:1 to 95:5) to give the ligand as a pasty solid, which was dissolved in a small amount of DCM and added on hexane in a dropwise manner. Evaporation of the solvents gave Boc-*L*-Phe-NHOMe as a white solid. Spectral data recorded agreed with the previously reported.

3. Kinetic Resolution via Palladium(II)-Catalyzed Alkenyl C–H Activation

General procedure for the kinetic resolution of allyl triflamides through Pd(II)-catalyzed C–H functionalization with allenes

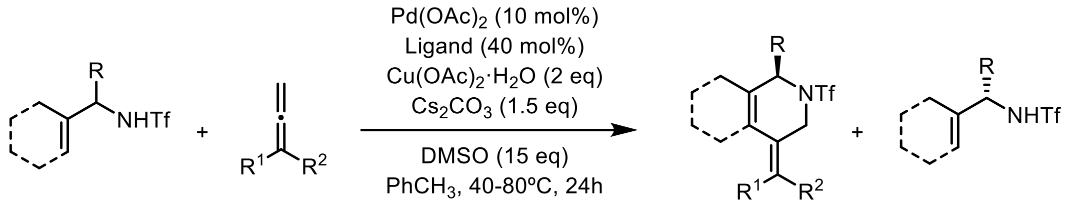

rac-**1a** - *rac*-**1m** **2a - 2i** **3aa - 3hc** **1a - 1m**

$Pd(OAc)_2$ (2.2 mg, 10 mol%), Ligand Boc-Phe-NHOMe or Boc-Phe-OH (40 mol%), $Cu(OAc)_2{\cdot}H_2O$ (40 mg, 2 eq), Cs_2CO_3 (48.9 mg, 1.5 eq) and the corresponding allyltriflamide (0.1 mmol, 1 eq) were weighed in air and placed in a Schlenk tube with a magnetic stir bar. Then, toluene (1.2 mL, 0.08M), DMSO (15 eq) and the corresponding allene (0.1 mmol, 1 eq) were added. The tube was sealed with a rubber septum and the reaction mixture was stirred 24 hours at the indicate temperature (40-80°C). Then, the resulting mixture was cooled to room temperature and filtered through a pad of silica gel and florisil, eluting with Et_2O. Evaporation and column chromatography (silica gel, hexane:Et_2O 99:1 to 80:20) afforded the desired product and the enantioenriched remaining starting material.

IMPORTANT: For measurement of the *ee* value of some of the enantioenriched unreactive starting materials (**1h**, **1i**, **1j**, **1k**, **1l**, **1m**) the following reaction has been applied:

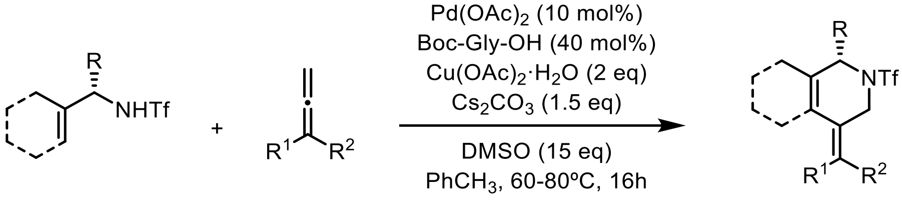

1h - **1m** **2a** or **2c** **3-SM**

$Pd(OAc)_2$ (10 mol%), Boc-Gly-OH (40 mol%), $Cu(OAc)_2{\cdot}H_2O$ (2 eq), Cs_2CO_3 (1.5 eq) and the enantioenriched remaining starting material previously obtained (1 eq) were weighed in air and placed in a Schlenk tube with a magnetic stir bar. Then, toluene (0.08M), DMSO (15 eq) and the same allene to obtain the chiral product (1 eq) were added. The tube was sealed with a rubber septum and the reaction mixture was stirred 16 hours at the indicate temperature (60-80°C). Then, the resulting mixture was cooled to room temperature and filtered through a pad of silica gel and florisil, eluting with Et_2O. Evaporation and column chromatography (silica gel, hexane:Et_2O 99:1) afforded the desired product.

(*R*)-3-cyclohexylidene-6-ethyl-5-methyl-4-phenyl-1-((trifluoromethyl)sulfonyl)-1,2,3,6-tetrahydropyridine (3aa)

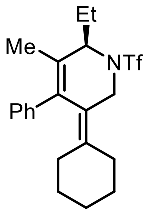

3aa

Using Boc-Phe-NHOMe as ligand at 70°C, **3aa** was obtained as a yellow oil (18.7 mg, 46%). **^{1}H NMR** (300 MHz, $CDCl_3$) δ, ppm: 7.37 – 7.22 (m, 3H), 7.14 (d, *J* = 7.6 Hz, 2H), 4.45 – 4.29 (m, 2H), 4.13 (t, *J* = 6.9 Hz, 1H), 2.27 – 2.10 (m, 2H), 1.90 – 1.74 (m, 5H), 1.67 – 1.50 (m, 5H), 1.48 – 1.37 (m, 2H), 1.08 (m, 5H). **^{19}F NMR** (282 MHz, $CDCl_3$) δ, ppm: -75.36 (s). **^{13}C NMR** (75 MHz, $CDCl_3$) δ, ppm: 141.5 (C), 141.0 (C), 135.0 (C), 134.8 (C), 129.3 (CH), 128.1 (CH), 126.5 (CH), 121.4 (C), 120.3 (q, *J* = 324.8 Hz, C), 62.7 (CH), 45.8 (CH_2), 32.5 (CH_2), 31.3 (CH_2), 27.9 (CH_2), 27.0 (CH_2), 26.3 (CH_2), 19.9 (CH_3), 11.0 (CH_3). **HRMS** (APCI, $[M+H]^+$) *m/z* calculated for $C_{21}H_{27}F_3NO_2S$: 414.1709; found 414.1710.

Enantioselectivity of the cycloadduct product was determined by chiral SFC analysis on Phenomenex Cellulose-1 at 40°C (CO_2 : MeOH = 99:1, 1 mL/min, λ=254 nm).

Racemic sample (*rac*-**3aa**)

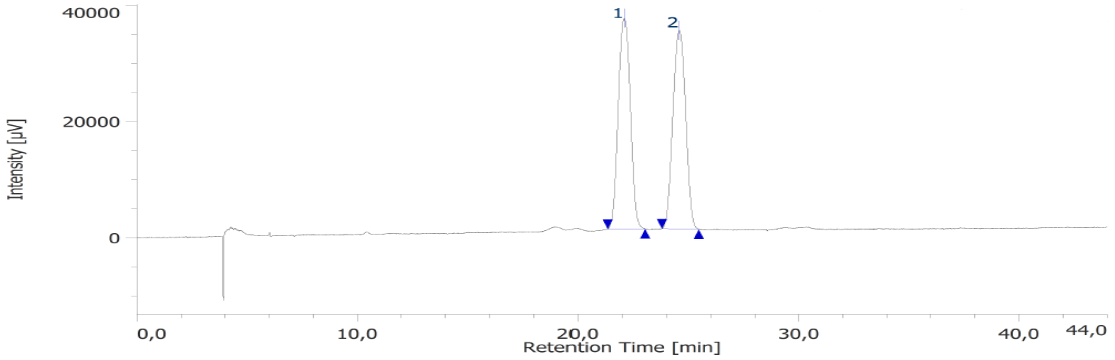

#	Peak Name	CH	tR [min]	Area [μV·sec]	Height [μV]	Area%	Height%	Quantity	NTP	Resolution	Symmetry Factor	Warning
1	Unknown	11	22,073	1386301	36215	50,178	51,475	N/A	7102	2,316	1,023	
2	Unknown	11	24,560	1376477	34139	49,822	48,525	N/A	7897	N/A	1,024	

Asymmetric sample (**3aa**, 97:3 *e.r.*, 94% *ee*)

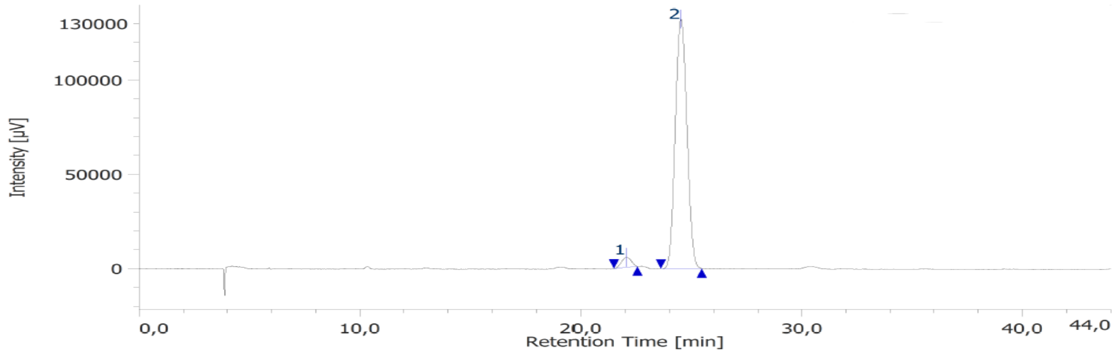

#	Peak Name	CH	tR [min]	Area [μV·sec]	Height [μV]	Area%	Height%	Quantity	NTP	Resolution	Symmetry Factor	Warning
1	Unknown	11	22,040	165768	5322	3,293	3,866	N/A	10250	2,655	0,954	
2	Unknown	11	24,510	4868700	132335	96,707	96,134	N/A	9716	N/A	1,057	

Enantioselectivity of the remaining starting material was determined by chiral HPLC analysis on Chiralpak IB at rt (Hexane : *i*PrOH = 98:2, 0.5 mL/min, λ=254 nm).

Racemic sample (*rac*-**1a**)

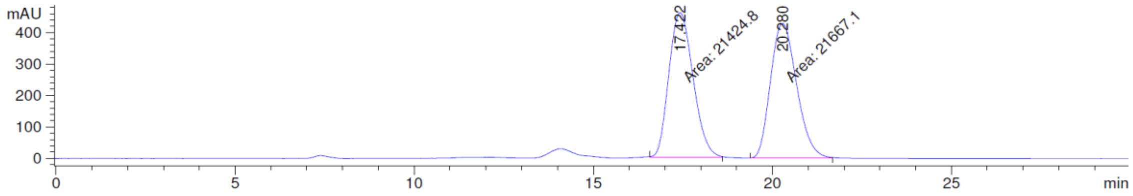

Peak #	RetTime [min]	Type	Width [min]	Area [mAU*s]	Height [mAU]	Area %
1	17.422	MM	0.7789	2.14248e4	458.45050	49.7188
2	20.280	MM	0.8431	2.16671e4	428.30322	50.2812

Asymmetric sample (**1a**, 95:5 *e.r.*, 90% *ee*)

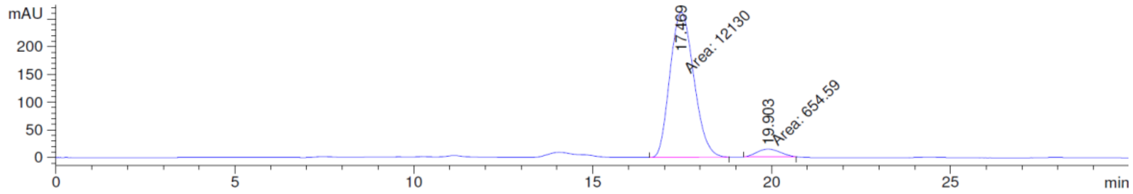

Peak #	RetTime [min]	Type	Width [min]	Area [mAU*s]	Height [mAU]	Area %
1	17.469	MM	0.7727	1.21300e4	261.63016	94.8799
2	19.903	MM	0.7629	654.58960	14.30130	5.1201

(*R*)-6-ethyl-5-methyl-3-(nonan-5-ylidene)-4-phenyl-1-((trifluoromethyl)sulfonyl)-1,2,3,6-tetrahydropyridine (3ab)

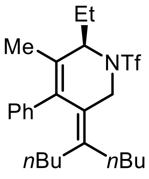

3ab

Using Boc-Phe-NHOMe as ligand at 75°C, **3ab** was obtained as a yellow oil (18.3 mg, 40%). **^{1}H NMR** (300 MHz, $CDCl_3$) δ, ppm: 7.40 – 7.23 (m, 3H), 7.18 – 7.08 (m, 2H), 4.40 (d, *J* = 13.7 Hz, 1H), 4.27 (d, *J* = 13.8 Hz, 1H), 4.10 (t, *J* = 7.5 Hz, 1H), 2.12 – 1.97 (m, 2H), 1.92 – 1.71 (m, 5H), 1.63 – 1.47 (m, 2H), 1.42 – 1.24 (m, 5H), 1.11 – 0.99 (m, 4H), 0.99 – 0.90 (m, 3H), 0.90 – 0.74 (m, 2H), 0.67 (t, *J* = 7.0 Hz, 3H). **^{19}F NMR** (282 MHz, $CDCl_3$) δ, ppm: -75.27 (s). **^{13}C NMR** (75 MHz, $CDCl_3$) δ, ppm: 142.3 (C), 141.1 (C), 135.5 (C), 135.0 (C), 129.4 (CH), 128.2 (CH), 126.8 (CH), 124.5 (C), 120.51 (q, *J* = 324.9 Hz), 62.8 (CH), 46.4 (CH_2), 33.9 (CH_2), 33.2 (CH_2), 30.9 (CH_2), 30.1 (CH_2), 26.7 (CH_2), 23.1 (CH_2), 23.0 (CH_2), 20.0 (CH_3), 14.10 (CH_3), 13.92 (CH_3), 11.18 (CH_3). **HRMS** (APCI, $[M+H]^+$) *m/z* calculated for $C_{24}H_{35}F_3NO_2S$: 458.2335; found 458.2333.

Enantioselectivity of the cycloadduct product was determined by chiral SFC analysis on Phenomenex Cellulose-1 at 40°C (CO_2 : MeOH = 99:1, 1 mL/min, λ=254 nm).

Racemic sample (*rac*-**3ab**)

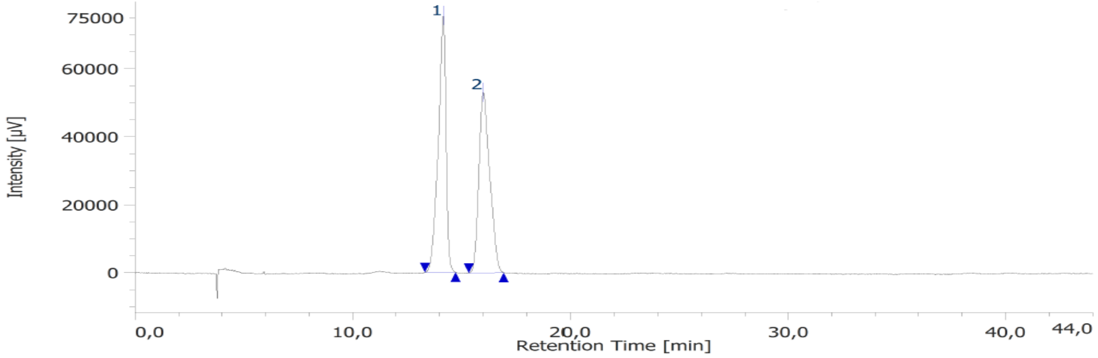

#	Peak Name	CH	tR [min]	Area [µV·sec]	Height [µV]	Area%	Height%	Quantity	NTP	Resolution	Symmetry Factor	Warning
1	Unknown	7	14,170	1873935	75440	50,390	58,717	N/A	8626	2,404	0,764	
2	Unknown	7	16,013	1844936	53042	49,610	41,283	N/A	4772	N/A	1,269	

Asymmetric sample (**3ab**, 95:5 *e.r.*, 90% *ee*)

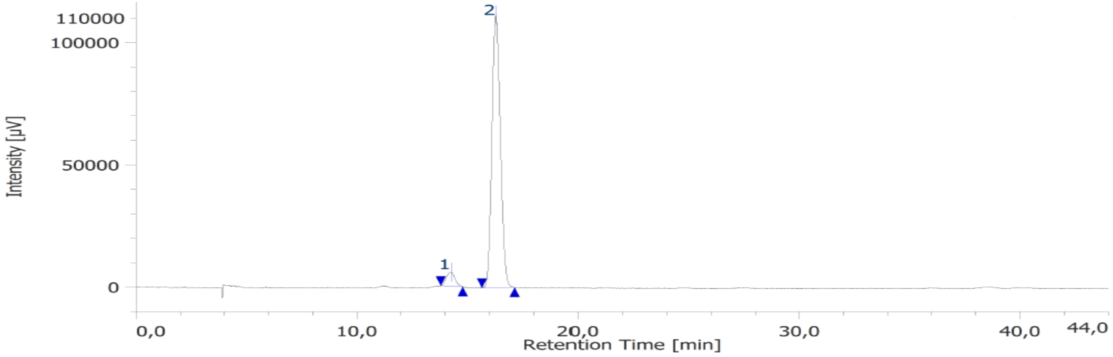

#	Peak Name	CH	tR [min]	Area [µV·sec]	Height [µV]	Area%	Height%	Quantity	NTP	Resolution	Symmetry Factor	Warning
1	Unknown	7	14,260	158177	5740	5,053	4,904	N/A	5826	2,796	1,057	
2	Unknown	7	16,293	2972464	111307	94,947	95,096	N/A	8398	N/A	1,121	

Enantioselectivity of the remaining starting material was determined by chiral HPLC analysis on Chiralpak IB at rt (Hexane : *i*PrOH = 98:2, 0.5 mL/min, λ=254 nm).

Racemic sample (*rac*-**1a**)

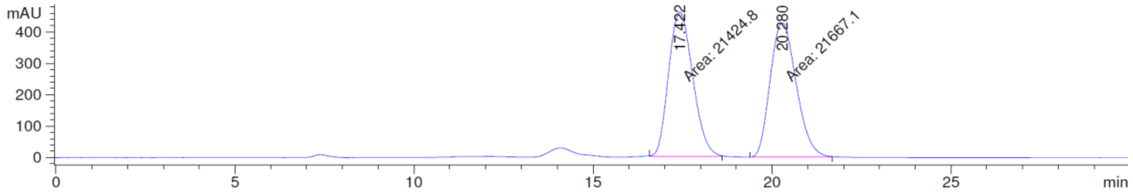

Peak #	RetTime [min]	Type	Width [min]	Area [mAU*s]	Height [mAU]	Area %
1	17.422	MM	0.7789	2.14248e4	458.45050	49.7188
2	20.280	MM	0.8431	2.16671e4	428.30322	50.2812

Asymmetric sample (**1a**, 91:9 *e.r.*, 82% *ee*)

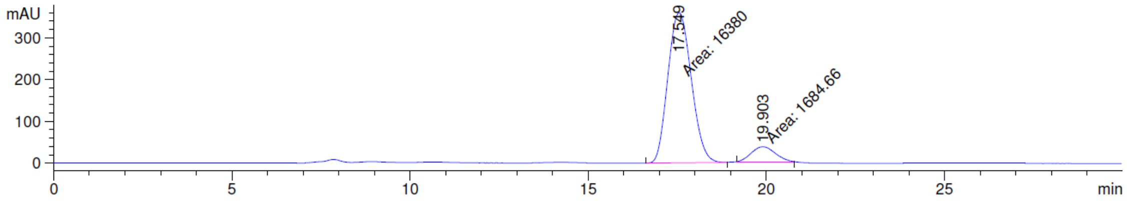

Peak #	RetTime [min]	Type	Width [min]	Area [mAU*s]	Height [mAU]	Area %
1	17.549	MM	0.7585	1.63800e4	359.91064	90.6743
2	19.903	MM	0.7776	1684.66138	36.10990	9.3257

(*R*,*Z*)-3-(cyclohexylmethylene)-6-ethyl-5-methyl-4-phenyl-1-((trifluoromethyl)sulfonyl)-1,2,3,6-tetrahydropyridine (3ac)

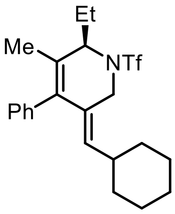

3ac

Using Boc-Phe-NHOMe as ligand at 75°C, **3ac** was obtained as a white solid (19.2 mg, 45%) and as a single diastereoisomer (*Z*:*E* > 20:1). ^{1}**H NMR** (300 MHz, $CDCl_3$) δ, ppm: 7.41 – 7.27 (m, 3H), 7.02 – 6.93 (m, 2H), 4.76 (d, *J* = 16.9 Hz, 1H), 4.67 (d, *J* = 9.5 Hz, 1H), 4.13 (dd, *J* = 10.5, 3.3 Hz, 1H), 4.03 (d, *J* = 16.9 Hz, 1H), 2.18 – 2.03 (m, 1H), 1.97 – 1.69 (m, 3H), 1.68 – 1.54 (m, 5H), 1.51 (s, 3H), 1.33 – 1.14 (m, 2H), 1.10 (t, *J* = 7.4 Hz, 3H), 0.96 – 0.79 (m, 2H). ^{19}**F NMR** (282 MHz, $CDCl_3$) δ, ppm: -76.56 (s). ^{13}**C NMR** (75 MHz, $CDCl_3$) δ, ppm: 138.4 (C), 135.5 (CH), 134.8 (C), 131.3 (C), 129.8 (CH), 128.4 (CH), 128.1 (C), 127.0 (CH), 120.1 (q, *J* = 324.0 Hz), 61.4 (CH), 41.3 (CH_2), 37.1 (CH), 32.8 (CH_2), 32.5 (CH_2), 25.9 (CH_2), 25.8 (CH_2), 25.8 (CH_2), 24.5 (CH_2), 19.2 (CH_3), 11.12 (CH_3). **HRMS** (APCI, $[M+H]^+$) *m/z* calculated for $C_{22}H_{29}F_3NO_2S$, 428.1866; found 428.1861.

The stereochemistry of the exocyclic double bond was assigned based on the observed nOe between H_a (6.90 ppm) and H_b (4.67 ppm) and the absence of a nOe between H_a (6.90 ppm) and H_c or H_d (4.76 ppm, 4.03 ppm).

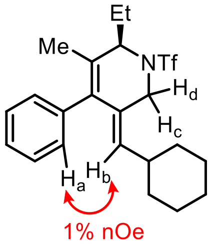

Enantioselectivity of the cycloadduct product was determined by chiral SFC analysis on Phenomenex Cellulose-1 at 40^oC (CO_2 : MeOH = 99:1, 1 mL/min, λ=254 nm).

Racemic sample (*rac*-**3ac**)

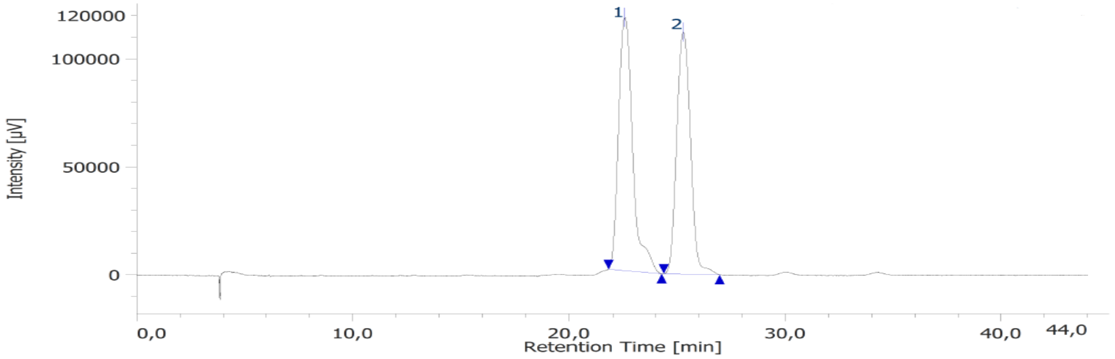

#	Peak Name	CH	tR [min]	Area [µV·sec]	Height [µV]	Area%	Height%	Quantity	NTP	Resolution	Symmetry Factor	Warning
1	Unknown	7	22,553	5370122	117142	51,850	51,135	N/A	6113	2,323	1,554	
2	Unknown	7	25,267	4986945	111941	48,150	48,865	N/A	7232	N/A	1,081	

Asymmetric sample (**3ac**, 96:4 *e.r.*, 92% *ee*)

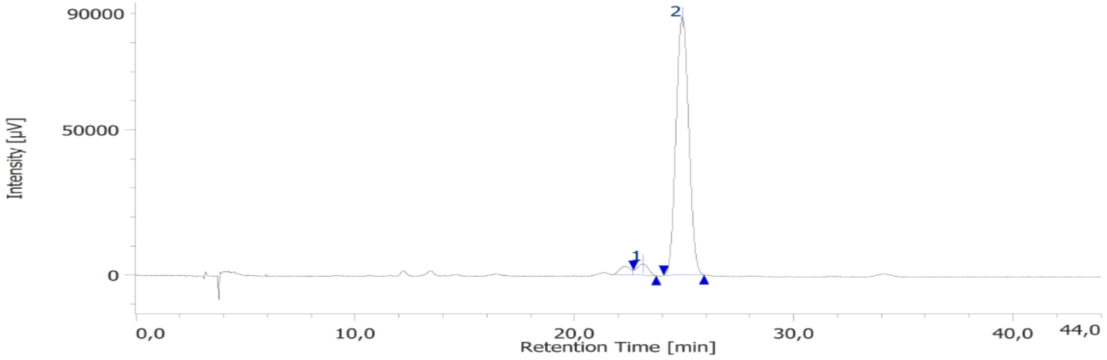

#	Peak Name	CH	tR [min]	Area [µV·sec]	Height [µV]	Area%	Height%	Quantity	NTP	Resolution	Symmetry Factor	Warning
1	Unknown	7	23,107	141678	3943	3,807	4,253	N/A	7665	1,688	N/A	
2	Unknown	7	24,913	3580084	88784	96,193	95,747	N/A	8356	N/A	1,049	

Enantioselectivity of the remaining starting material was determined by chiral HPLC analysis on Chiralpak IB at rt (Hexane : *i*PrOH = 98:2, 0.5 mL/min, λ=254 nm).

Racemic sample (*rac*-**1a**)

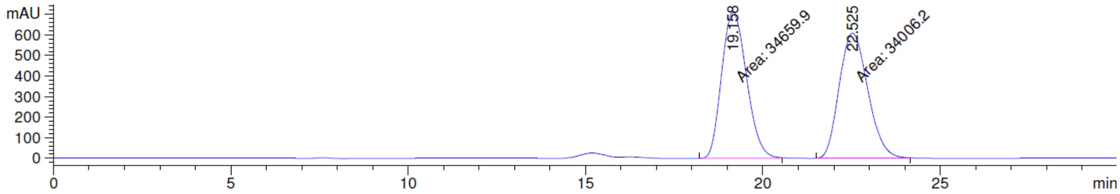

Peak #	RetTime [min]	Type	Width [min]	Area [mAU*s]	Height [mAU]	Area %
1	19.158	MM	0.8138	3.46599e4	709.87299	50.4760
2	22.525	MM	0.9333	3.40062e4	607.26520	49.5240

Asymmetric sample (**1a**, 95:5 *e.r.*, 90% *ee*)

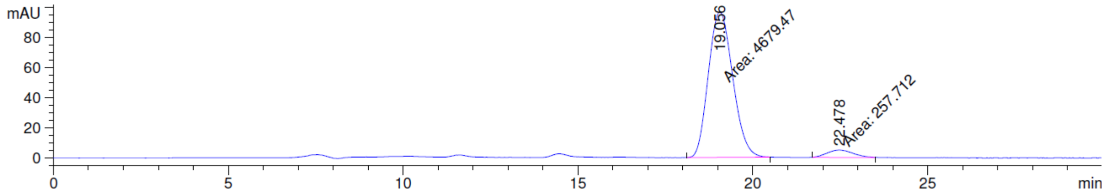

Peak #	RetTime [min]	Type	Width [min]	Area [mAU*s]	Height [mAU]	Area %
1	19.056	MM	0.8064	4679.47266	96.71806	94.7802
2	22.478	MM	0.8612	257.71240	4.98731	5.2198

(*R*,*Z*)-3-(2-((tert-butyldimethylsilyl)oxy)ethylidene)-6-ethyl-5-methyl-4-phenyl-1-((trifluoromethyl)sulfonyl)-1,2,3,6-tetrahydropyridine (3ad)

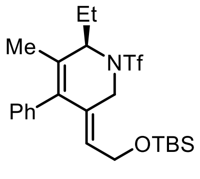

3ad

Using Boc-Phe-NHOMe as ligand at 80°C, **3ad** was obtained as a yellow oil (20.2 mg, 41%) and with a *Z*:*E* ratio > 10:1. ^{1}**H NMR** (500 MHz, $CDCl_3$) δ, ppm: 7.38 – 7.34 (m, 2H), 7.32 – 7.29 (m, 1H), 7.02 – 6.98 (m, 2H), 4.96 (t, *J* = 5.9 Hz, 1H), 4.78 (d, *J* = 17.2 Hz, 1H), 4.20 – 4.13 (m, 3H), 4.03 (d, *J* = 17.3 Hz, 1H), 1.92 – 1.83 (m, 1H), 1.78 – 1.69 (m, 1H), 1.55 (s, 3H), 1.10 (t, *J* = 7.4 Hz, 3H), 0.83 (s, 9H), -0.00 (s, 3H), -0.02 (s, 3H). ^{19}**F NMR** (282 MHz, $CDCl_3$) δ, ppm: -76.09 (s). ^{13}**C NMR** (126 MHz, $CDCl_3$) δ, ppm: 137.9 (C), 134.3 (C), 133.2 (C), 130.9 (C), 129.7 (CH), 128.5 (CH), 128.3 (CH), 127.2 (CH), 120.15 (q, *J* = 323.8 Hz), 61.2 (CH), 59.8 (CH_2), 41.3 (CH_2), 25.6 (CH_3), 24.5 (CH_2), 19.3 (CH_3), 18.4 (C), 11.1 (CH_3), -5.2 (CH_3), -5.2 (CH_3). **HRMS** (APCI, $[M+H]^+$) *m/z* calculated for $C_{23}H_{33}F_3NO_3SSi$: 488.1897; found 488.1898.

The stereochemistry of the exocyclic double bond was assigned based on the observed nOe between H_a (4.96 ppm) and H_b (7 ppm, 1.3%) and the absence of a nOe between H_a (4.96 ppm) and H_c or H_d (4.78 ppm, 4.03 ppm).

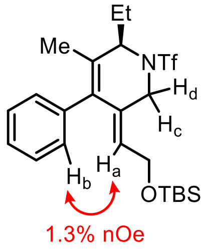

Enantioselectivity of the cycloadduct product was determined by chiral SFC analysis on Phenomenex Cellulose-1 at 40⁰C (CO_2 : MeOH = 99:1, 1 mL/min, λ=254 nm).

Racemic sample (*rac*-**3ad**)

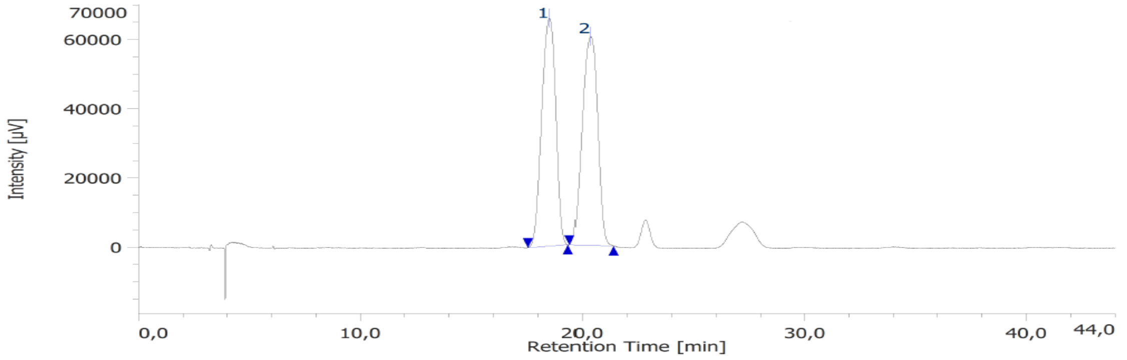

#	Peak Name	CH	tR [min]	Area [μV·sec]	Height [μV]	Area%	Height%	Quantity	NTP	Resolution	Symmetry Factor	Warning
1	Unknown	11	18,533	2899952	65856	50,102	52,153	N/A	3703	1,429	0,917	
2	Unknown	11	20,347	2888196	60418	49,898	47,847	N/A	3770	N/A	0,938	

Asymmetric sample (**3ad**, 95:5 *e.r.*, 90% *ee*)

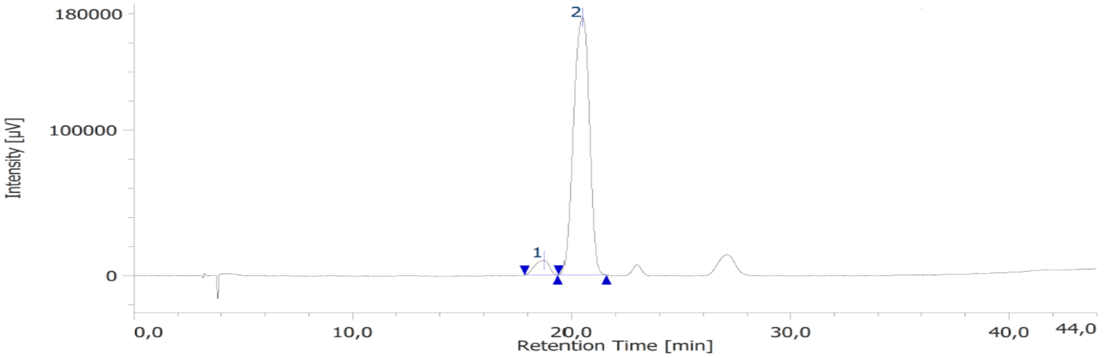

#	Peak Name	CH	tR [min]	Area [μV·sec]	Height [μV]	Area%	Height%	Quantity	NTP	Resolution	Symmetry Factor	Warning
1	Unknown	11	18,743	467811	10002	4,993	5,347	N/A	3198	1,275	0,828	
2	Unknown	11	20,473	8901280	177060	95,007	94,653	N/A	3448	N/A	0,926	

Enantioselectivity of the remaining starting material was determined by chiral HPLC analysis on Chiralpak IB at rt (Hexane : *i*PrOH = 98:2, 0.5 mL/min, λ=254 nm).

Racemic sample (*rac*-**1a**)

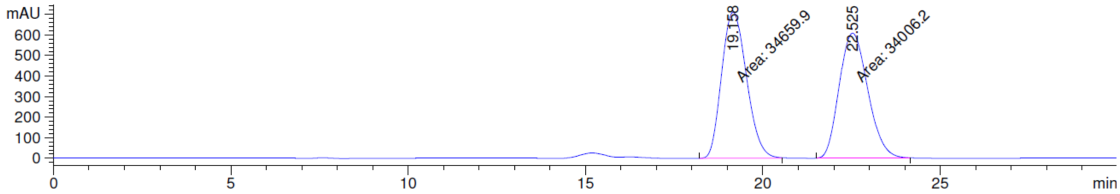

Peak #	RetTime [min]	Type	Width [min]	Area [mAU*s]	Height [mAU]	Area %
1	19.158	MM	0.8138	3.46599e4	709.87299	50.4760
2	22.525	MM	0.9333	3.40062e4	607.26520	49.5240

Asymmetric sample (**1a**, 98:2 *e.r.*, 96% *ee*)

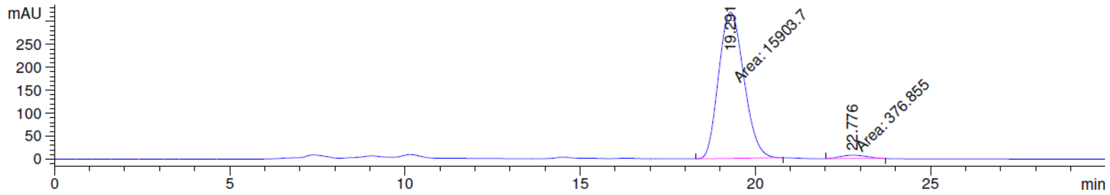

Peak #	RetTime [min]	Type	Width [min]	Area [mAU*s]	Height [mAU]	Area %
1	19.291	MM	0.8316	1.59037e4	318.73874	97.6853
2	22.776	MM	0.8586	376.85471	7.31548	2.3147

(*R*,*Z*)-2-(2-(6-ethyl-5-methyl-4-phenyl-1-((trifluoromethyl)sulfonyl)-1,6-dihydropyridin-3(2H)-ylidene)ethyl)-1H-indene-1,3(2H)-dione (3ae)

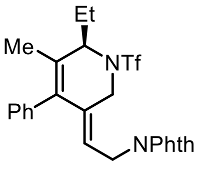

3ae

Using Boc-Phe-NHOMe as ligand at 80°C, **3ae** was obtained as a white solid (17.4 mg, 35%) and as a single diastereoisomer (*Z*:*E* > 20:1). ^{1}**H NMR** (500 MHz, $CDCl_3$) δ, ppm: 7.84 – 7.77 (m, 2H), 7.71 – 7.68 (m, 2H), 7.34 – 7.28 (m, 3H), 6.95 (d, *J* = 7.0 Hz, 2H), 5.08 (d, *J* = 17.3 Hz, 1H), 4.87 (t, *J* = 7.6 Hz, 1H), 4.33 – 4.25 (m, 2H), 4.24 – 4.15 (m, 2H), 1.92 – 1.83 (m, 1H), 1.83 – 1.73 (m, 1H), 1.54 (s, 3H), 1.13 (t, *J* = 7.4 Hz, 3H). ^{19}**F NMR** (282 MHz, $CDCl_3$) δ, ppm: -75.77 (s). ^{13}**C NMR** (126 MHz, $CDCl_3$) δ, ppm: 167.8 (C), 137.5 (C), 134.1 (CH), 134.1 (C), 133.8 (C), 132.2 (C), 129.7 (CH), 128.7 (CH), 127.4 (CH), 123.5 (CH), 121.4 (CH), 120.1 (q, *J* = 324.0 Hz, C), 61.4 (CH), 41.3 (CH_2), 35.0 (CH_2), 24.4 (CH_2), 19.4 (CH_3), 11.23 (CH_3). **HRMS** (APCI, $[M+H]^+$) *m/z* calculated for $C_{25}H_{24}F_3N_2O_4S$: 505.1403; found 505.1415.

The stereochemistry of the exocyclic double bond was assigned based on the observed nOe between H_a (4.87 ppm) and H_b (6.95 ppm, 2%).

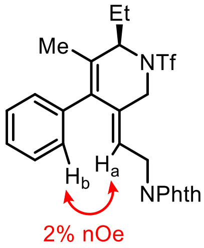

Enantioselectivity of the cycloadduct product was determined by chiral SFC analysis on Phenomenex Cellulose-1 at 40°C (CO_2 : MeOH = 95:5, 1 mL/min, λ=254 nm).

Racemic sample (*rac*-**3ae**)

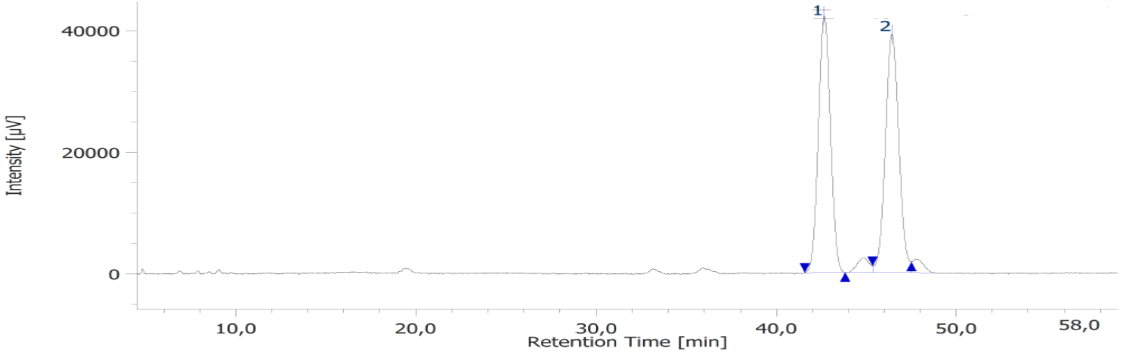

#	Peak Name	CH	tR [min]	Area [μV·sec]	Height [μV]	Area%	Height%	Quantity	NTP	Resolution	Symmetry Factor	Warning
1	Unknown	11	42,653	1969555	42231	49,035	51,859	N/A	18881	2,886	1,035	
2	Unknown	11	46,410	2047092	39203	50,965	48,141	N/A	18393	N/A	1,084	

Asymmetric sample (**3ae**, 90:10 *e.r.*, 80% *ee*)

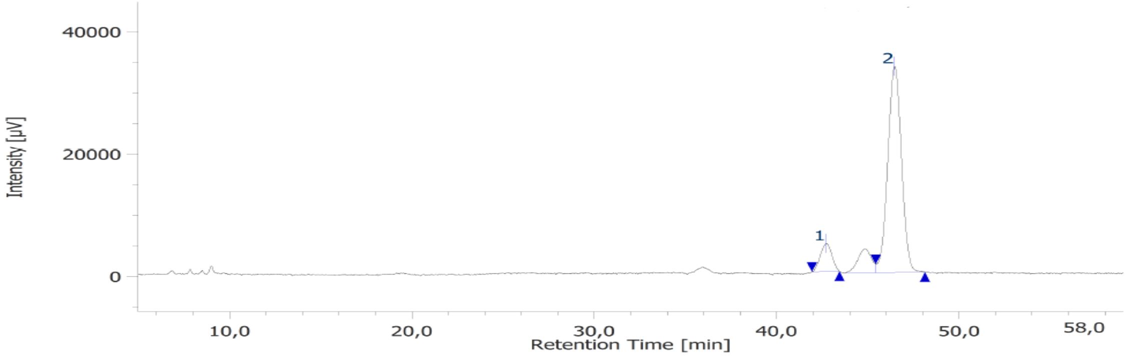

#	Peak Name	CH	tR [min]	Area [μV·sec]	Height [μV]	Area%	Height%	Quantity	NTP	Resolution	Symmetry Factor	Warning
1	Unknown	11	42,703	201311	4592	10,203	11,985	N/A	20008	2,898	0,992	
2	Unknown	11	46,447	1771828	33722	89,797	88,015	N/A	18059	N/A	0,986	

Enantioselectivity of the remaining starting material was determined by chiral HPLC analysis on Chiralpak IB at rt (Hexane : *i*PrOH = 98:2, 0.5 mL/min, λ=254 nm).

Racemic sample (*rac*-**1a**)

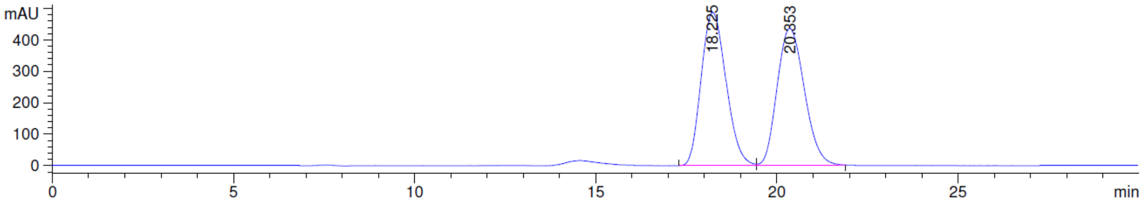

Peak #	RetTime [min]	Type	Width [min]	Area [mAU*s]	Height [mAU]	Area %
1	18.225	BV	0.7467	2.32308e4	486.86664	50.4846
2	20.353	VB	0.8061	2.27848e4	435.72626	49.5154

Asymmetric sample (**1a**, 94:6 *e.r.*, 88% *ee*)

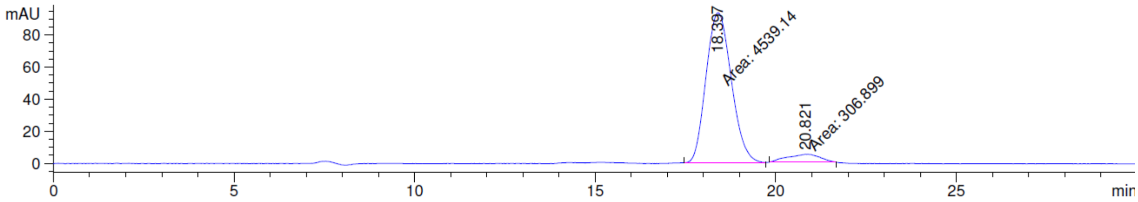

Peak #	RetTime [min]	Type	Width [min]	Area [mAU*s]	Height [mAU]	Area %
1	18.397	MM	0.8091	4539.14014	93.49895	93.6670
2	20.821	MM	1.0545	306.89944	4.85072	6.3330

(*R*,*Z*)-2-(6-ethyl-5-methyl-4-phenyl-1-((trifluoromethyl)sulfonyl)-1,6-dihydropyridin-3(2*H*)-ylidene)ethyl acetate (3af)

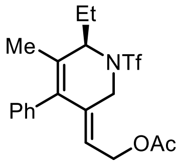

3af

Using Boc-Phe-NHOMe as ligand at 80ºC, **3af** was obtained as a yellowish oil (12.7 mg, 30% yield) and with a *Z:E* ratio > 10:1. **^{1}H NMR** (500 MHz, $CDCl_3$) δ, ppm: 7.39 – 7.36 (m, 2H), 7.34 – 7.31 (m, 1H), 7.02 – 6.99 (m, 2H), 4.97 (t, *J* = 7.0 Hz, 1H), 4.82 (d, *J* = 17.2 Hz, 1H), 4.61 – 4.50 (m, 2H), 4.18 (dd, *J* = 10.7, 3.1 Hz, 1H), 4.09 (d, *J* = 17.2 Hz, 1H), 2.01 (s, 3H), 1.91 – 1.85 (m, 1H), 1.78 – 1.72 (m, 1H), 1.56 (s, 3H), 1.11 (t, *J* = 7.4 Hz, 3H). **^{19}F NMR** (282 MHz, $CDCl_3$) δ, ppm: -76.07 (s). **^{13}C NMR** (126 MHz, $CDCl_3$) δ, ppm: 170.8 (C), 137.5 (C), 135.2 (C), 134.4 (C), 134.1 (C), 129.7 (CH), 128.7 (CH), 127.4 (CH), 121.7 (CH), 120.9 (q, *J* = 323.6 Hz, C), 61.3 (CH), 60.1 (CH_2), 41.1 (CH_2), 24.4 (CH_2), 20.9 (CH_3), 19.4 (CH_3), 11.2 (CH_3). **HRMS** (APCI, $[M]^+$) *m/z* calculated for $C_{19}H_{22}F_3NO_4S$: 417.1216; found 417.1217.

The stereochemistry of the exocyclic double bond was assigned based on the observed nOe between H_a (4.82 ppm) and H_b (4.61 – 4.50 ppm) and the absence of a nOe between H_a (4.82 ppm) and H_c (4.97 ppm).

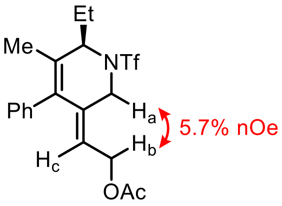

Enantioselectivity of the cycloadduct product was determined by chiral SFC analysis on Phenomenex Cellulose-1 at 40 ºC (CO_2:MeOH = 99:1, 1 mL/min, λ= 254 nm).

Racemic Sample (*rac*-**3af**)

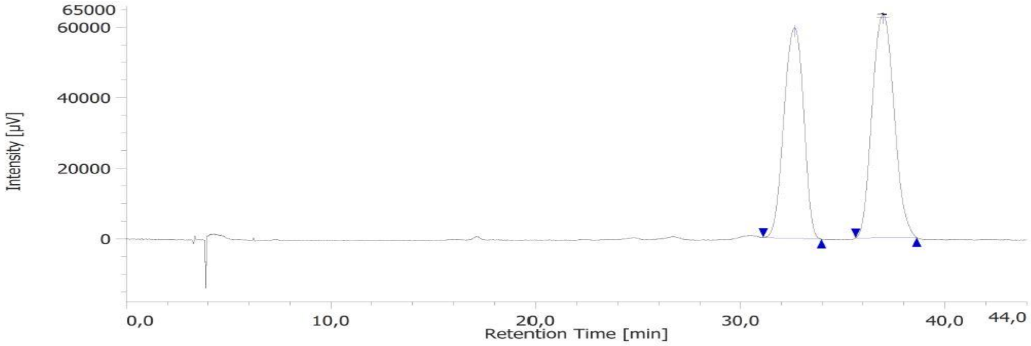

#	Peak Name	CH	tR [min]	Area [µV·sec]	Height [µV]	Area%	Height%	Quantity	NTP	Resolution	Symmetry Factor	Warning
1	Unknown	7	32,623	3999883	59635	45,871	48,547	N/A	4993	2,244	0,943	
2	Unknown	7	36,957	4719945	63204	54,129	51,453	N/A	5328	N/A	1,111	

Asymmetric sample (**3af**, 97:3 *e.r.*, 94% *ee*)

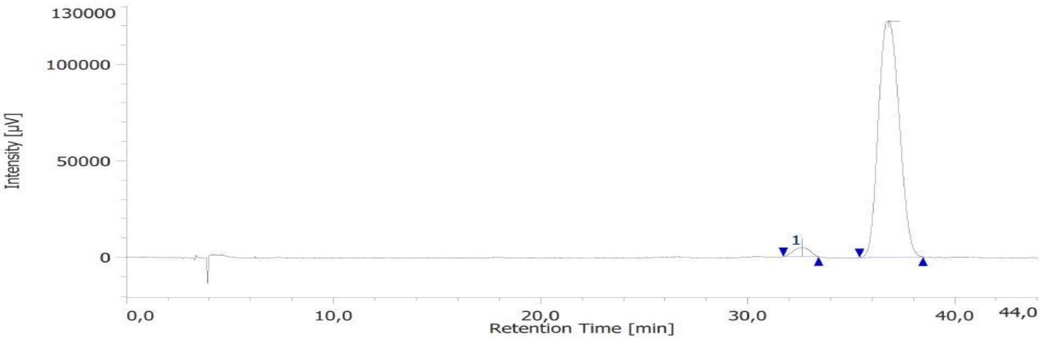

#	Peak Name	CH	tR [min]	Area [µV·sec]	Height [µV]	Area%	Height%	Quantity	NTP	Resolution	Symmetry Factor	Warning
1	Unknown	7	32,630	268607	4774	3,032	3,711	N/A	6660	2,372	0,932	
2	Unknown	7	36,773	8590316	123865	96,968	96,289	N/A	5971	N/A	1,125	

Enantioselectivity of the remaining starting material was determined by chiral HPLC analysis on Chiralpak IB at rt (Hexane:^{i}PrOH = 98:2, 0.5 mL/min, λ=254 nm)

Racemic Sample (*rac*-**1a**)

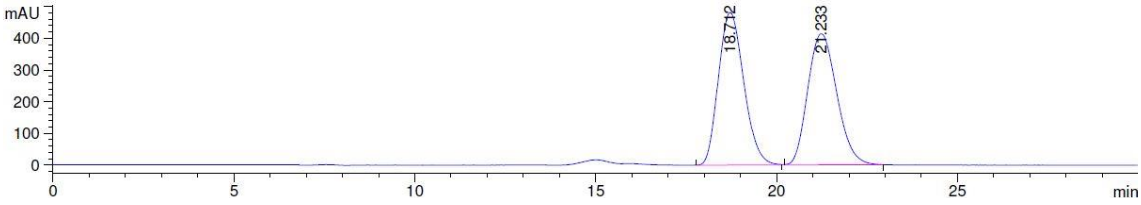

Peak #	RetTime [min]	Type	Width [min]	Area [mAU*s]	Height [mAU]	Area %
1	18.712	BB	0.7629	2.34683e4	481.28320	50.4478
2	21.233	BB	0.8762	2.30516e4	413.64481	49.5522

Asymmetric sample (**1a**, 87:13 *e.r.*, 74% ee_{SM})

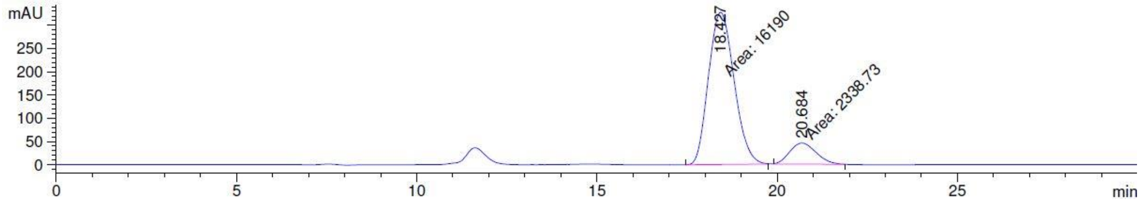

Peak #	RetTime [min]	Type	Width [min]	Area [mAU*s]	Height [mAU]	Area %
1	18.427	MM	0.8242	1.61900e4	327.39706	87.3778
2	20.684	MM	0.8564	2338.73169	45.51228	12.6222

(*R*,*Z*)-3-benzylidene-6-ethyl-5-methyl-4-phenyl-1-((trifluoromethyl)sulfonyl)-1,2,3,6-tetrahydropyridine (3ag)

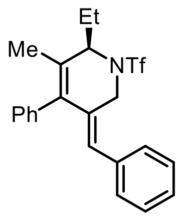

3ag

Using Boc-Phe-NHOMe as ligand at 80°C, **3ag** was obtained as a white solid (16.9 mg, 40%) and as a single diastereoisomer (*Z*:*E* > 20:1). ^{1}**H NMR** (300 MHz, $CDCl_3$) δ, ppm: 7.49 – 7.18 (m, 6H), 7.17 – 7.02 (m, 4H), 5.93 (s, 1H), 4.98 (d, *J* = 17.0 Hz, 1H), 4.31 (d, *J* = 17.1 Hz, 1H), 4.22 (dd, *J* = 10.5, 3.1 Hz, 1H), 2.02 – 1.77 (m, 2H), 1.62 (s, 3H), 1.15 (t, *J* = 7.4 Hz, 3H). ^{19}**F NMR** (282 MHz, $CDCl_3$) δ, ppm: -76.58 (s). ^{13}**C NMR** (75 MHz, $CDCl_3$) δ, ppm: 138.2 (C), 136.4 (C), 135.1 (C), 134.1 (C), 131.6 (C), 129.9 (CH), 128.9 (CH), 128.7 (CH), 128.6 (CH), 127.4 (CH), 127.4 (CH), 120.1 (q, *J* = 323.8 Hz, C), 61.4 (CH), 42.0 (CH_2), 24.5 (CH_2), 19.5 (CH_3), 11.2 (CH_3). **HRMS** (APCI, $[M+H]^+$) *m/z* calculated for $C_{22}H_{23}F_3NO_2S$, 422.1396; found 422.1395.

The stereochemistry of the exocyclic double bond was assigned based on the observed nOe between H_a (4.98 ppm) and H_b (7.07 ppm) and the absence of a nOe between H_a (4.98 ppm) and H_c (5.93 ppm).

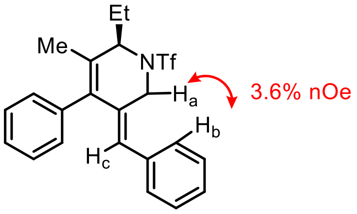

Enantioselectivity of the cycloadduct product was determined by chiral SFC analysis on Phenomenex i-Cellulose-5 at 40°C (CO_2 : MeOH = 99:1, 1 mL/min, λ=254 nm).

Racemic sample (*rac*-**3ag**)

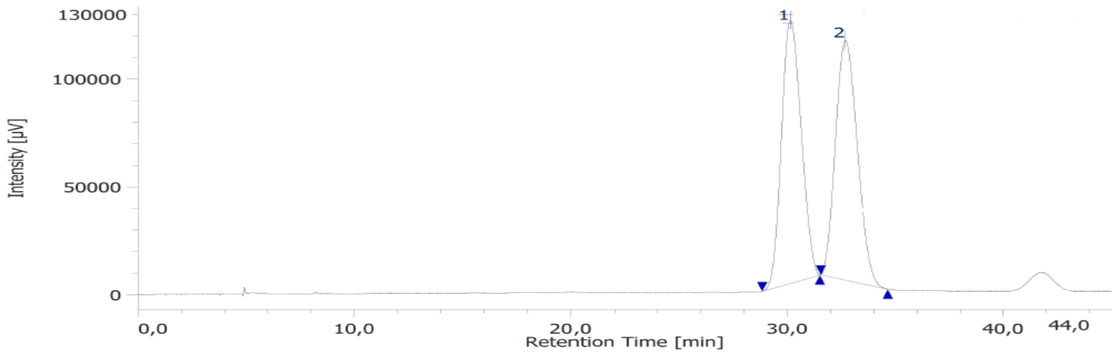

#	Peak Name	CH	tR [min]	Area [µV·sec]	Height [µV]	Area%	Height%	Quantity	NTP	Resolution	Symmetry Factor	Warning
1	Unknown	6	30,133	7774323	122014	49,848	52,355	N/A	4861	1,403	1,128	
2	Unknown	6	32,680	7821825	111039	50,152	47,645	N/A	4685	N/A	1,168	

Asymmetric sample (**3ag**, 94:6 *e.r.*, 88% *ee*)

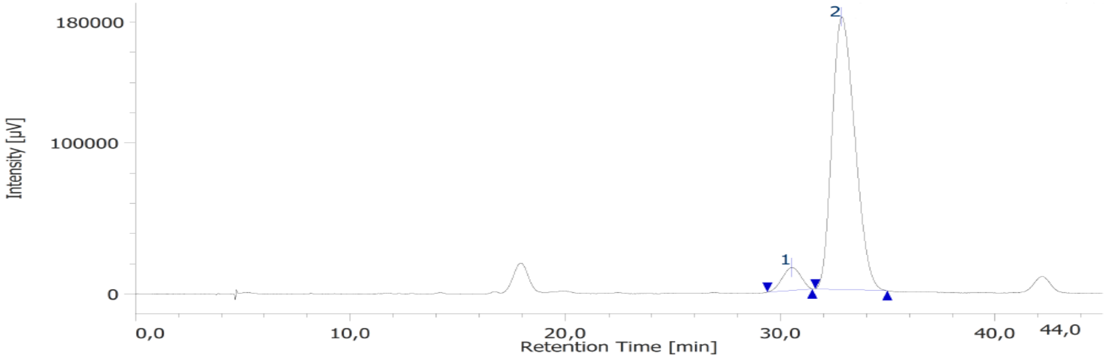

#	Peak Name	CH	tR [min]	Area [µV·sec]	Height [µV]	Area%	Height%	Quantity	NTP	Resolution	Symmetry Factor	Warning
1	Unknown	6	30,517	907455	15192	6,355	7,766	N/A	5447	1,277	0,948	
2	Unknown	6	32,843	13371345	180443	93,645	92,234	N/A	4319	N/A	1,254	

Enantioselectivity of the remaining starting material was determined by chiral HPLC analysis on Chiralpak IB at rt (Hexane : *i*PrOH = 98:2, 0.5 mL/min, λ=254 nm).

Racemic sample (*rac*-**1a**)

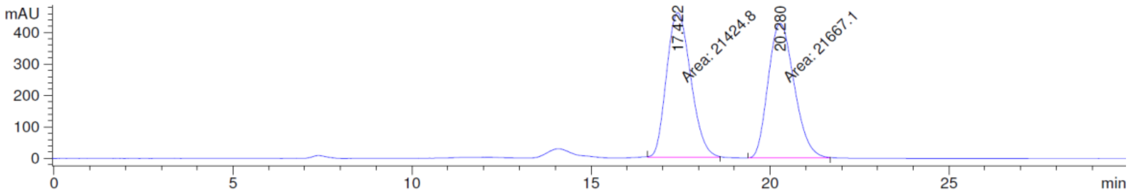

Peak #	RetTime [min]	Type	Width [min]	Area [mAU*s]	Height [mAU]	Area %
1	17.422	MM	0.7789	2.14248e4	458.45050	49.7188
2	20.280	MM	0.8431	2.16671e4	428.30322	50.2812

Asymmetric sample (**1a**, 89:11 *e.r.*, 78% *ee*)

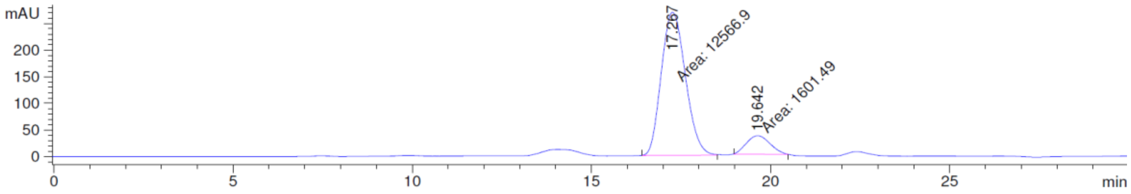

Peak #	RetTime [min]	Type	Width [min]	Area [mAU*s]	Height [mAU]	Area %
1	17.267	MM	0.7787	1.25669e4	268.98740	88.6967
2	19.642	MM	0.7648	1601.49280	34.89842	11.3033

(*R*,*Z*)-6-ethyl-5-methyl-4-phenyl-3-(4-(trifluoromethyl)benzylidene)-1-((trifluoromethyl)sulfonyl)-1,2,3,6-tetrahydropyridine (3ah)

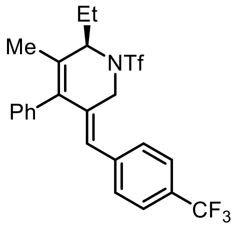

3ah

Using Boc-Phe-NHOMe as ligand at 80°C, **3ah** was obtained as a white solid (15.1 mg, 31%) and as a single diastereoisomer (*Z*:*E* > 20:1). **^{1}H NMR** (300 MHz, $CDCl_3$) δ, ppm: 7.55 (d, *J* = 7.8 Hz, 2H), 7.48 – 7.32 (m, 3H), 7.17 (d, *J* = 7.6 Hz, 2H), 7.10 (d, *J* = 7.3 Hz, 2H), 5.91 (s, 1H), 4.89 (d, *J* = 17.5 Hz, 1H), 4.32 – 4.16 (m, 2H), 2.05 – 1.73 (m, 2H), 1.61 (s, 3H), 1.13 (t, *J* = 7.0 Hz, 3H). **^{19}F NMR** (282 MHz, $CDCl_3$) δ, ppm: -62.72 (s), -76.19 (s). **^{13}C NMR** (75 MHz, $CDCl_3$) δ, ppm: 140.0 (C), 137.8 (C), 135.7 (C), 134.9 (C), 133.5 (C), 129.8 (CH), 129,5 (C), 129.1 (CH), 128.8 (CH), 127.6 (CH), 125.6 (CH), 125.5 (CH), 124.17 (q, *J* = 272.0 Hz, C), 120.07 (d, *J* = 324.0 Hz, C), 61.5 (CH), 41.8 (CH_2), 24.4 (CH_2), 19.6 (CH_3), 11.2 (CH_3). **HRMS** (APCI, $[M+H]^+$) *m/z* calculated for $C_{23}H_{22}F_6NO_2S$, 490.1270; found 490.1257.

The stereochemistry of the exocyclic double bond was assigned based on the observed nOe between H_a (4.89 ppm) and H_b (7.17 ppm, 3.2%) and the absence of a nOe between H_a (4.89 ppm) and H_c (5.91 ppm).

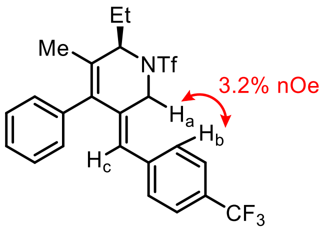

Enantioselectivity of the cycloadduct product was determined by chiral SFC analysis on Phenomenex Cellulose-1 at 40°C (CO_2 : MeOH = 99:1, 1 mL/min, λ=254 nm).

Racemic sample (*rac*-**3ah**)

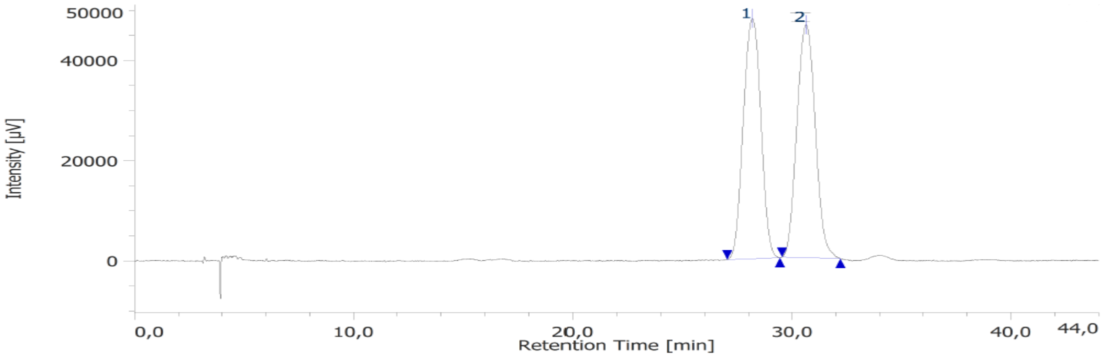

#	Peak Name	CH	tR [min]	Area [μV·sec]	Height [μV]	Area%	Height%	Quantity	NTP	Resolution	Symmetry Factor	Warning
1	Unknown	11	28,180	2675658	47921	49,594	50,781	N/A	5419	1,578	0,997	
2	Unknown	11	30,627	2719459	46447	50,406	49,219	N/A	6028	N/A	1,077	

Asymmetric sample (**3ah**, 97:3 *e.r.*, 94% *ee*)

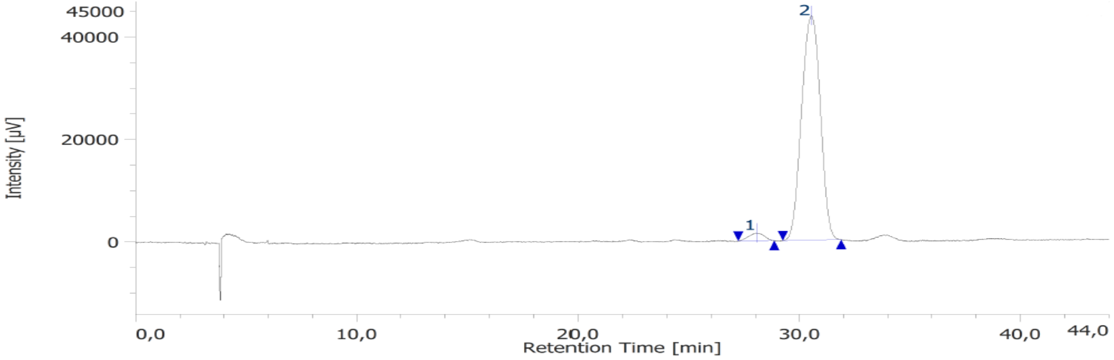

#	Peak Name	CH	tR [min]	Area [μV·sec]	Height [μV]	Area%	Height%	Quantity	NTP	Resolution	Symmetry Factor	Warning
1	Unknown	11	28,053	73953	1528	2,783	3,375	N/A	6462	1,644	0,924	
2	Unknown	11	30,527	2583805	43749	97,217	96,625	N/A	5673	N/A	0,982	

Enantioselectivity of the remaining starting material was determined by chiral HPLC analysis on Chiralpak IB at rt (Hexane : *i*PrOH = 98:2, 0.5 mL/min, λ=254 nm).

Racemic sample (*rac*-**1a**)

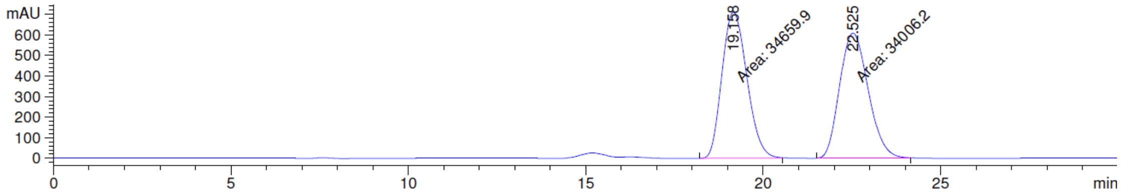

Peak #	RetTime [min]	Type	Width [min]	Area [mAU*s]	Height [mAU]	Area %
1	19.158	MM	0.8138	3.46599e4	709.87299	50.4760
2	22.525	MM	0.9333	3.40062e4	607.26520	49.5240

Asymmetric sample (**1a**, 81:19 *e.r.*, 62% *ee*)

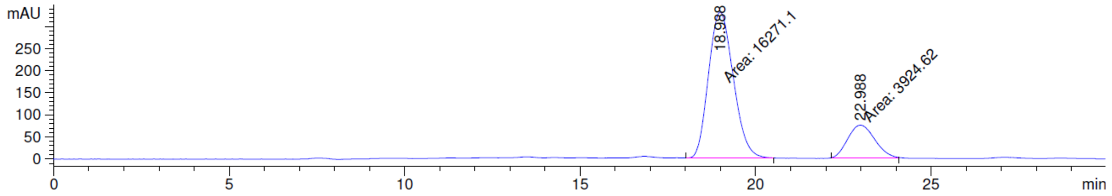

Peak #	RetTime [min]	Type	Width [min]	Area [mAU*s]	Height [mAU]	Area %
1	18.988	MM	0.8206	1.62711e4	330.48367	80.5670
2	22.988	MM	0.8844	3924.62134	73.96192	19.4330

(*R*,*E*)-3-benzylidene-6-ethyl-2,2,5-trimethyl-4-phenyl-1-((trifluoromethyl)sulfonyl)-1,2,3,6-tetrahydropyridine (3ai) + (6*R*)-6-ethyl-5-methyl-2,4-diphenyl-3-(propan-2-ylidene)-1-((trifluoromethyl)sulfonyl)-1,2,3,6-tetrahydropyridine (3ai')

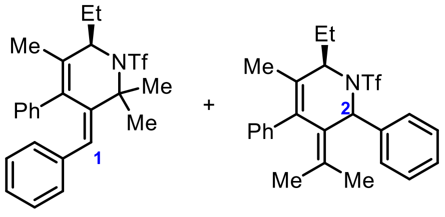

Using Boc-Phe-NHOMe as ligand at 70°C, **3ai** (*E*:*Z* > 15:1) and **3ai'** were obtained as a colourless oil (15.6 mg, 35%) and as an inseparable mixture of regioisomers (*r*:*r* = 4.5:1). **^{1}H NMR** (500 MHz, $CDCl_3$) δ, ppm: 7.24 – 7.10 (m, 2H), 7.07 – 7.02 (m, 1H), 6.94 – 6.81 (m, 8H), 6.78 (d, *J* = 7.3 Hz, 2H), 6.60 (s, 1H), 6.00 (s, 0.22H), 4.14 – 4.02 (m, 1.22H), 2.12 – 2.01 (m, 2H), 1.98 – 1.90 (m, 9H), 1.80 (s, 3H), 1.04 (t, *J* = 7.4 Hz, 3H), 0.89 (t, *J* = 7.3 Hz, 0.75H). **^{19}F NMR** (282 MHz, $CDCl_3$) δ, ppm: -74.40 (s), -75.01 (s). **^{13}C NMR** (189 MHz, $CDCl_3$) δ, ppm:, 145.0 (C), 140.4 (C), 140.3 (C), 137.7 (C), 137.5 (C), 136.9 (C), 136.0 (C), 134.5 (C), 133.2 (C), 131.0 (C), 130.1 (CH), 129.9 (CH), 129.0 (CH), 128.9 (CH), 128.4 (CH), 128.0 (CH), 127.5 (CH), 127.2 (CH), 126.8 (CH), 126.7 (CH), 126.5 (CH), 126.0 (CH), 125.7 (CH), 119.99 (q, *J* = 326.3 Hz, C), 66.3 (C), 65.0 (CHx2), 60.8 (CH), 30.5 (CH_3), 29.8 (CH_2), 29.8 (CH_3), 27.5 (CH_2), 21.4 (CH_3), 20.6 (CH_3), 11.5 (CH_3). **HRMS** (APCI, $[M+H]^+$) *m/z* calculated for $C_{24}H_{27}F_3NO_2S$, 450.1709; found 450.1708.

The stereochemistry of the exocyclic double bond in **3ai** was assigned based on the observed nOe between H_a (6.60 ppm) and *blue* methyl signals (2 ppm, 9%).

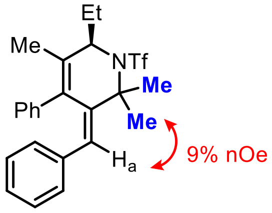

Enantioselectivity of the mixture of regioisomers was determined by chiral SFC analysis on Phenomenex Cellulose-1 at 40°C (CO_2 : MeOH = 98:2, 1 mL/min, λ=254 nm).

Racemic sample

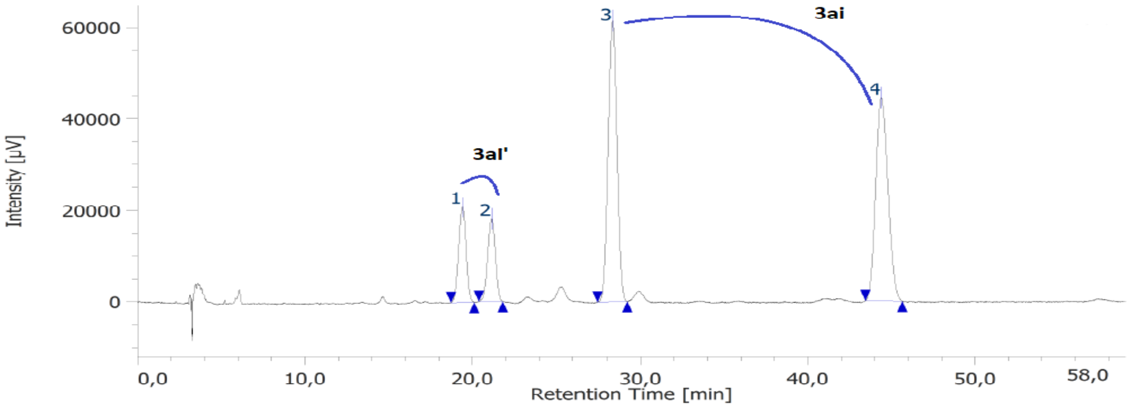

#	Peak Name	CH	tR [min]	Area [µV·sec]	Height [µV]	Area%	Height%	Quantity	NTP	Resolution	Symmetry Factor	Warning
1	Unknown	9	19,400	652975	20841	11,335	14,421	N/A	8498	2,069	0,995	
2	Unknown	9	21,160	578445	18022	10,041	12,470	N/A	9596	7,746	0,965	
3	Unknown	9	28,317	2262498	61351	39,275	42,452	N/A	13121	13,644	1,015	
4	Unknown	9	44,380	2266786	44303	39,349	30,656	N/A	16738	N/A	1,140	

Asymmetric sample (**3ai**, 93:7 *e.r.*, 86% *ee*; **3ai'**, 95:5 *e.r.*, 90% *ee*)

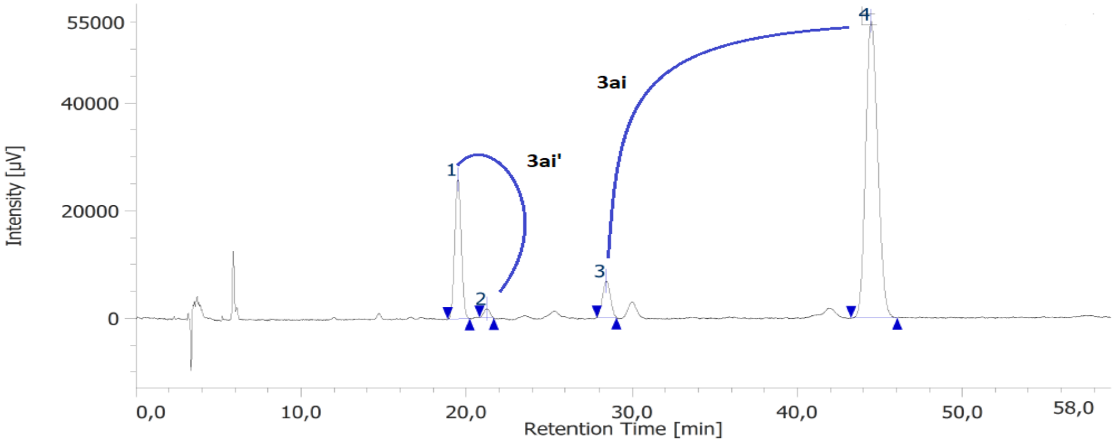

#	Peak Name	CH	tR [min]	Area [µV·sec]	Height [µV]	Area%	Height%	Quantity	NTP	Resolution	Symmetry Factor	Warning
1	Unknown	9	19,497	717527	25843	18,905	28,900	N/A	11040	2,459	1,038	
2	Unknown	9	21,277	42782	1690	1,127	1,890	N/A	14395	8,836	0,923	
3	Unknown	9	28,423	224746	6771	5,921	7,571	N/A	15520	14,168	1,001	
4	Unknown	9	44,457	2810378	55120	74,046	61,639	N/A	17178	N/A	1,156	

Enantioselectivity of the remaining starting material was determined by chiral HPLC analysis on Chiralpak IB at rt (Hexane : *i*PrOH = 98:2, 0.5 mL/min, λ=254 nm).

Racemic sample (*rac*-**1a**)

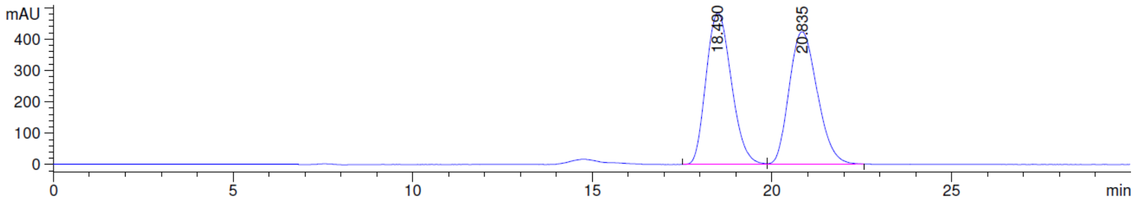

Peak #	RetTime [min]	Type	Width [min]	Area [mAU*s]	Height [mAU]	Area %
1	18.490	BB	0.7643	2.35569e4	483.60040	50.5531
2	20.835	BB	0.8477	2.30414e4	422.87766	49.4469

Asymmetric sample (**1a**, 94:6 *e.r.*, 88% *ee*)

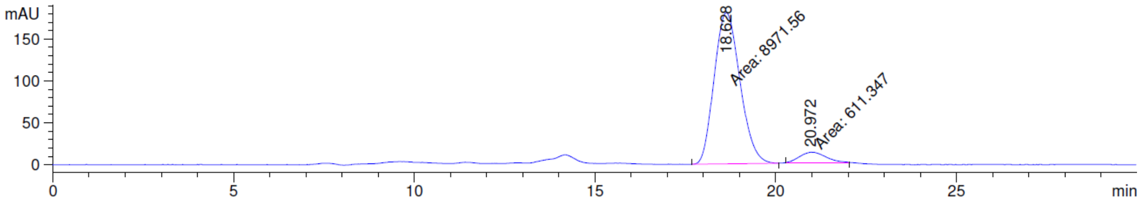

Peak #	RetTime [min]	Type	Width [min]	Area [mAU*s]	Height [mAU]	Area %
1	18.628	MM	0.8276	8971.56348	180.66393	93.6204
2	20.972	MM	0.8346	611.34741	12.20787	6.3796

(*R*)-3-cyclohexylidene-5,6-dimethyl-4-phenyl-1-((trifluoromethyl)sulfonyl)-1,2,3,6-tetrahydropyridine (3ba)

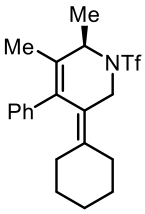

3ba

Using Boc-Phe-NHOMe as ligand at 75°C, **3ba** was obtained as a white solid (18.5 mg, 46% yield). **^{1}H NMR** (300 MHz, $CDCl_3$) δ, ppm: 7.37 – 7.30 (m, 2H), 7.28 – 7.21 (m, 1H), 7.16 – 7.12 (m, 2H), 4.43 – 4.34 (m, 2H), 4.32 – 4.24 (m, 1H), 2.28 – 2.19 (m, 2H), 1.79 (s, 3H), 1.65 – 1.54 (m, 4H), 1.48 (d, *J* = 6.7 Hz, 3H), 1.46 – 1.38 (m, 2H), 1.17 – 1.07 (m, 2H). **^{19}F NMR** (282 MHz, $CDCl_3$) δ, ppm: -75.66 (s). **^{13}C NMR** (75 MHz, $CDCl_3$) δ, ppm: 141.8 (C), 141.7 (C), 135.1 (C), 134.4 (C), 129.5 (CH), 128.2 (CH), 126.7 (CH), 121.8 (C), 120.3 (q, *J* = 324.3 Hz, C), 56.9 (CH), 45.0 (CH_2), 32.6 (CH_2), 31.9 (CH_2), 28.2 (CH_2), 27.2 (CH_2), 26.4 (CH_2), 20.3 (CH_3), 18.6 (CH_3). **HRMS** (APCI, $[M+H]^+$) *m/z* calculated for $C_{20}H_{25}F_3NO_2S$: 400.1553; found 400.1553.

Enantioselectivity of the cycloadduct product was determined by chiral SFC analysis on Phenomenex Cellulose-1 at 40°C (CO_2 : MeOH = 99:1, 1 mL/min, λ= 254 nm).

Racemic sample (*rac*-**3ba**)

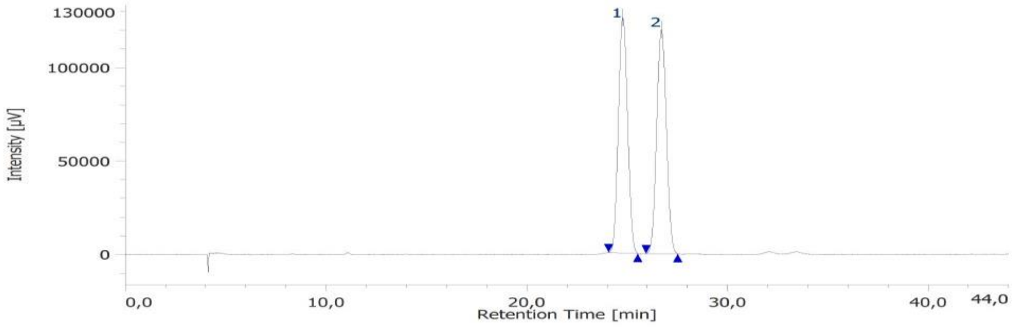

#	Peak Name	CH	tR [min]	Area [µV·sec]	Height [µV]	Area%	Height%	Quantity	NTP	Resolution	Symmetry Factor	Warning
1	Unknown	7	24.777	4023809	125860	50.008	51.207	N/A	13248	2.192	1.051	
2	Unknown	7	26.700	4022493	119927	49.992	48.793	N/A	14125	N/A	1.044	

Asymmetric sample (**3ba**, 95:5 *e.r.*, 90% *ee*)

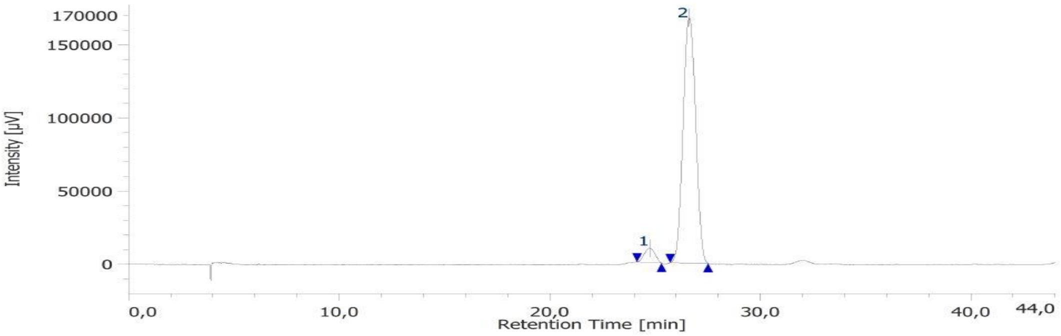

#	Peak Name	CH	tR [min]	Area [µV·sec]	Height [µV]	Area%	Height%	Quantity	NTP	Resolution	Symmetry Factor	Warning
1	Unknown	7	24.740	343527	9713	4.690	5.473	N/A	9956	1.769	0.972	
2	Unknown	7	26.617	6981508	167768	95.310	94.527	N/A	8791	N/A	1.029	

Enantioselectivity of the remaining starting material was determined by chiral HPLC analysis on Chiralpak IA3 at rt (Hexane : *i*PrOH = 98:2, 0.5 mL/min, λ=254 nm).

Racemic sample (*rac*-**1b**)

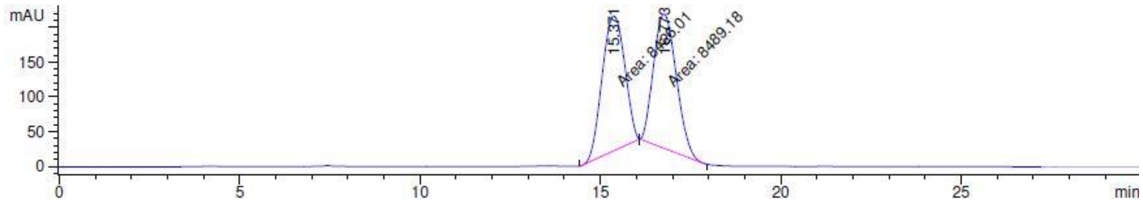

Peak #	RetTime [min]	Type	Width [min]	Area [mAU*s]	Height [mAU]	Area %
1	15.371	MM	0.7249	8426.00586	193.71617	49.8133
2	16.773	MM	0.7344	8489.17871	192.64542	50.1867

Asymmetric sample (**1b**, 96:4 *e.r.*, 92% *ee*)

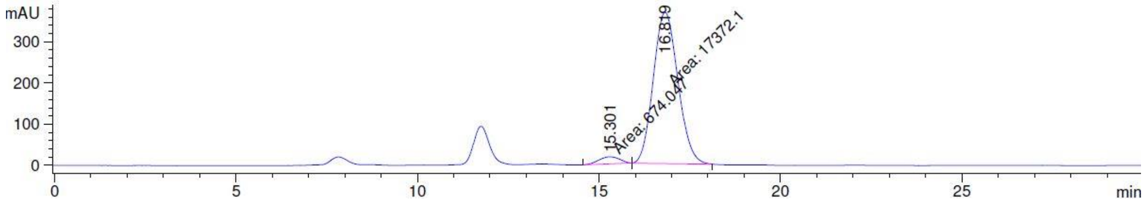

Peak #	RetTime [min]	Type	Width [min]	Area [mAU*s]	Height [mAU]	Area %
1	15.301	MM	0.6695	674.04718	16.78071	3.7351
2	16.819	MM	0.7885	1.73721e4	367.18243	96.2649

Absolute configuration of the cycloadduct **3ba** was determined as ***R*** by X-ray crystallography. The structure was deposited in the Cambridge Structural Database: 2043705.

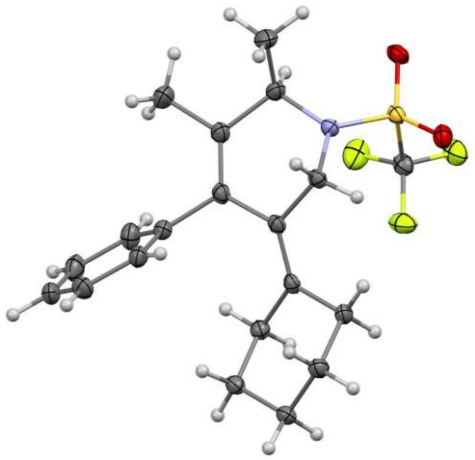

Absolute configuration of the remaining starting material **1b** was determined as ***S*** by X-ray crystallography. The structure was deposited in the Cambridge Structural Database: 2043710.

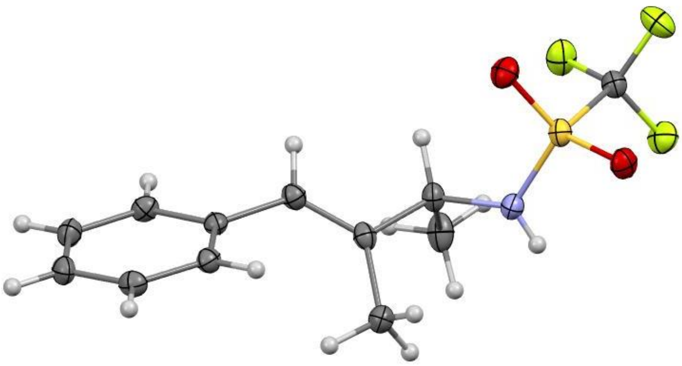

(*R*)-6-butyl-3-cyclohexylidene-5-methyl-4-phenyl-1-((trifluoromethyl)sulfonyl)-1,2,3,6-tetrahydropyridine (3ca)

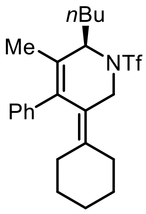

3ca

Using Boc-Phe-NHOMe as ligand at 75°C, **3ca** was obtained as a white solid (21.8 mg, 49% yield). **^{1}H NMR** (300 MHz, $CDCl_3$) δ, ppm: 7.36 – 7.28 (m, 2H), 7.28 – 7.18 (m, 1H), 7.12 (d, *J* = 7.5 Hz, 2H), 4.44 – 4.27 (m, 2H), 4.17 (t, *J* = 7.1 Hz, 1H), 2.28 – 2.06 (m, 2H), 1.83 (s, 3H), 1.80 – 1.72 (m, 2H), 1.71 – 1.60 (m, 1H), 1.59 – 1.48 (m, 3H), 1.48 – 1.30 (m, 6H), 1.17 – 1.05 (m, 2H), 0.93 (t, J = 6.7 Hz, 3H). **^{19}F NMR** (282 MHz, $CDCl_3$) δ, ppm: -75.17 (s). **^{13}C NMR** (75 MHz, $CDCl_3$) δ, ppm: 141.6 (C), 141.0 (C), 135.2 (C), 135.0 (C), 129.4 (CH), 128.2 (CH), 126.6 (CH), 121.6 (C), 120.4 (q, *J* = 324.7 Hz, C), 61.6 (CH), 45.9 (CH_2), 33.0 (CH_2), 32.6 (CH_2), 31.4 (CH_2), 28.6 (CH_2), 28.0 (CH_2), 27.2 (CH_2), 26.4 (CH_2), 22.6 (CH_2), 20.0 (CH_3), 14.1 (CH_3). **HRMS** (APCI, $[M+H]^+$) *m/z* calculated for $C_{23}H_{31}F_3NO_2S$: 442.2022; found 442.2021.

Enantioselectivity of the cycloadduct product was determined by chiral HPLC analysis on Chiralpak IB at rt (Hexane, 0.2 mL/min, λ=254 nm).

Racemic sample (*rac*-**3ca**)

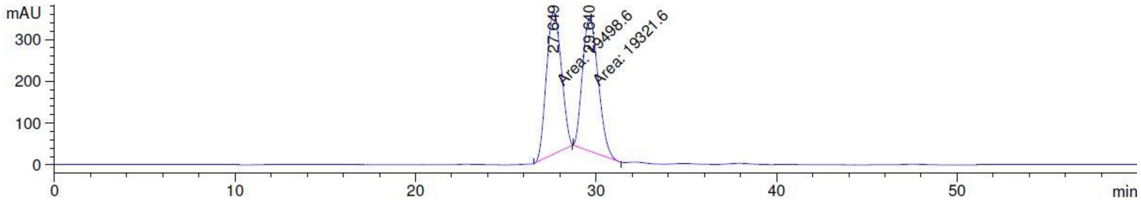

Peak #	RetTime [min]	Type	Width [min]	Area [mAU*s]	Height [mAU]	Area %
1	27.649	MM	0.9570	1.94986e4	339.57526	50.2281
2	29.640	MM	1.0125	1.93216e4	318.04230	49.7719

Asymmetric sample (**3ca**, 97:3 *e.r.*, 94% *ee*)

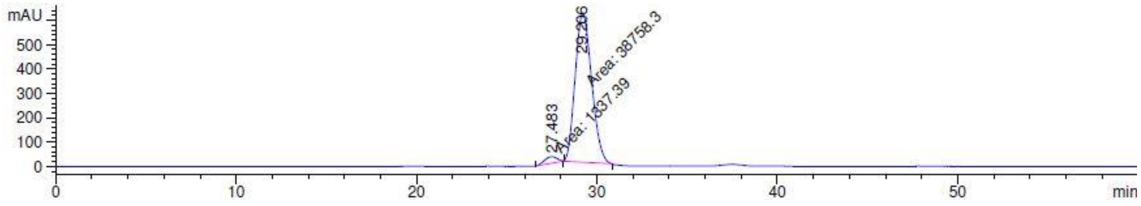

Peak #	RetTime [min]	Type	Width [min]	Area [mAU*s]	Height [mAU]	Area %
1	27.483	MM	0.8163	1337.38599	27.30531	3.3355
2	29.206	MM	1.0604	3.87583e4	609.15936	96.6645

Enantioselectivity of the remaining starting material was determined by chiral HPLC analysis on Chiralpak IA3 at rt (Hexane : *i*PrOH = 98:2, 0.5 mL/min, λ=254 nm).

Racemic sample (*rac*-**1c**)

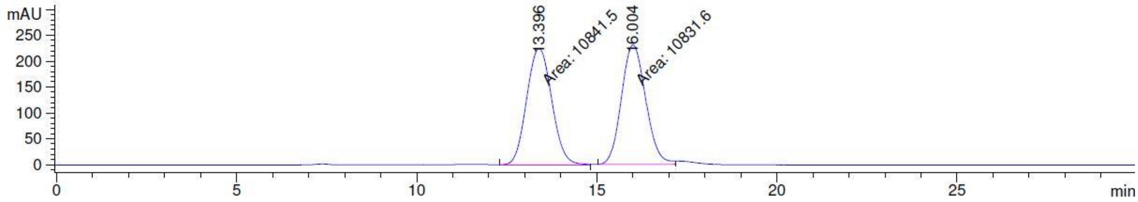

Peak #	RetTime [min]	Type	Width [min]	Area [mAU*s]	Height [mAU]	Area %
1	13.396	MM	0.8045	1.08415e4	224.60303	50.0230
2	16.004	MM	0.7824	1.08316e4	230.72429	49.9770

Asymmetric sample (**1c**, 97:3 *e.r.*, 94% *ee*)

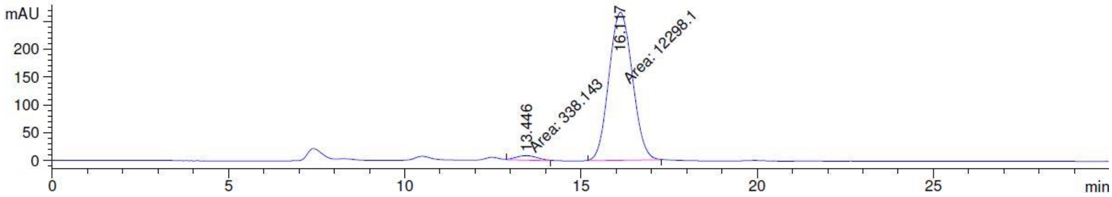

Peak #	RetTime [min]	Type	Width [min]	Area [mAU*s]	Height [mAU]	Area %
1	13.446	MM	0.6984	338.14297	8.06964	2.6760
2	16.117	MM	0.7722	1.22981e4	265.45099	97.3240

(*R*)-3-cyclohexylidene-6-isobutyl-5-methyl-4-phenyl-1-((trifluoromethyl)sulfonyl)-1,2,3,6-tetrahydropyridine (3da)

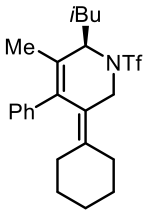

3da

Using Boc-Phe-NHOMe as ligand at 70°C, **3da** was obtained as a white solid (15.9 mg, 36% yield). **^{1}H NMR** (300 MHz, $CDCl_3$) δ, ppm: 7.34 – 7.28 (m, 2H), 7.25 – 7.19 (m, 1H), 7.13 – 7.08 (m, 2H), 4.43 – 4.32 (m, 2H), 4.27 – 4.20 (m, 1H), 2.27 – 2.17 (m, 1H), 2.15 – 2.05 (m, 1H), 1.82 (s, 3H), 1.77 – 1.60 (m, 3H), 1.60 – 1.49 (m, 4H), 1.48 – 1.35 (m, 2H), 1.16 – 1.06 (m, 2H), 1.03 – 0.95 (m, 6H). **^{19}F NMR** (282 MHz, $CDCl_3$) δ, ppm: -75.71 (s). **^{13}C NMR** (75 MHz, $CDCl_3$) δ, ppm: 141.6 (C), 140.8 (C), 135.5 (C), 134.8 (C), 129.4 (CH), 128.2 (CH), 126.6 (CH), 121.4 (C), 120.4 (q, *J* = 324.9 Hz, C), 60.0 (CH), 45.8 (CH_2), 42.0 (CH_2), 32.6 (CH_2), 31.4 (CH_2), 28.0 (CH_2), 27.3 (CH_2), 26.4 (CH_2), 25.2 (CH), 23.1 (CH_3), 22.4 (CH_3), 19.9 (CH_3). **HRMS** (APCI, $[M+H]^+$) *m/z* calculated for $C_{23}H_{31}F_3NO_2S$: 442.2022; found 442.2024.

Enantioselectivity of the cycloadduct product was determined by chiral HPLC analysis on Chiralpak IF3 at rt (Hexane, 0.5 mL/min, λ=254 nm).

Racemic sample (*rac*-**3da**)

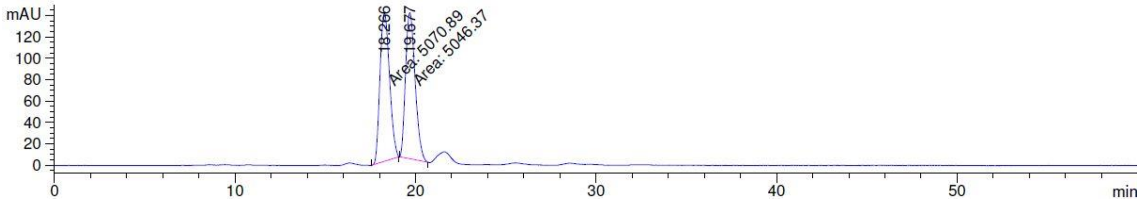

Peak #	RetTime [min]	Type	Width [min]	Area [mAU*s]	Height [mAU]	Area %
1	18.266	MM	0.6095	5070.88916	138.66040	50.1212
2	19.677	MM	0.6157	5046.37451	136.61263	49.8788

Asymmetric sample (**3da**, 96:4 *e.r.*, 92% *ee*)

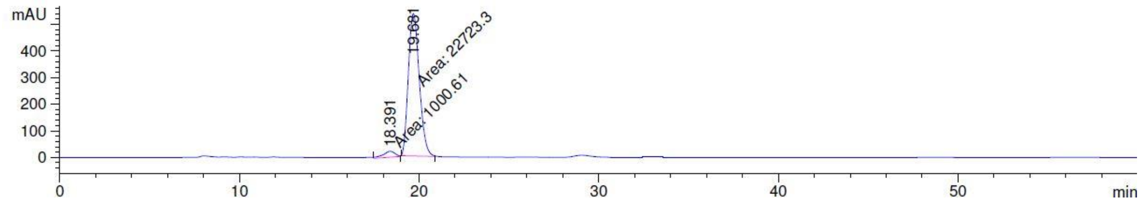

Peak #	RetTime [min]	Type	Width [min]	Area [mAU*s]	Height [mAU]	Area %
1	18.391	MM	0.7598	1000.60773	21.94888	4.2177
2	19.681	MM	0.7076	2.27233e4	535.20099	95.7823

Enantioselectivity of the remaining starting material was determined by chiral HPLC analysis on Chiralpak IA3 at rt (Hexane : *i*PrOH = 98:2, 0.5 mL/min, λ=220 nm).

Racemic sample (*rac*-**1d**)

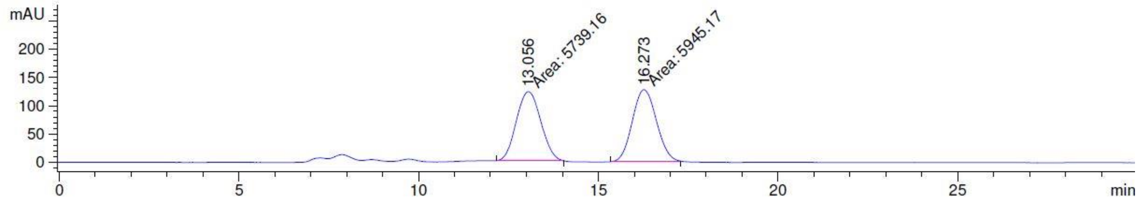

Peak #	RetTime [min]	Type	Width [min]	Area [mAU*s]	Height [mAU]	Area %
1	13.056	MM	0.7864	5739.15967	121.63890	49.1184
2	16.273	MM	0.7791	5945.17139	127.18729	50.8816

Asymmetric sample (**1d**, 96:4 *e.r.*, 92% *ee*)

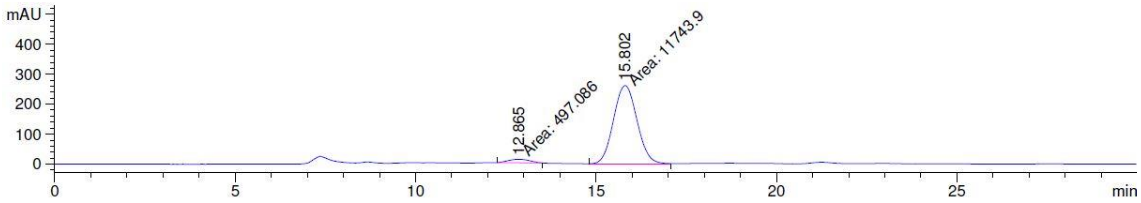

Peak #	RetTime [min]	Type	Width [min]	Area [mAU*s]	Height [mAU]	Area %
1	12.865	MM	0.6886	497.08633	12.03178	4.0608
2	15.802	MM	0.7505	1.17439e4	260.80704	95.9392

(*R*)-3-cyclohexylidene-6-ethyl-4-phenyl-5-propyl-1-((trifluoromethyl)sulfonyl)-1,2,3,6-tetrahydropyridine (3ea)

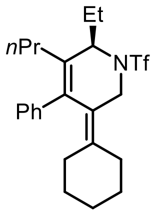

3ea

Using Boc-Phe-NHOMe as ligand at 70°C, **3ea** was obtained as a white solid (19.7 mg, 45% yield). ^{1}**H NMR** (300 MHz, $CDCl_3$) δ, ppm: 7.38 – 7.25 (m, 3H), 7.15 (d, *J* = 7.6 Hz, 2H), 4.46 – 4.30 (m, 2H), 4.27 – 4.20 (m, 1H), 2.27 – 2.09 (m, 4H), 1.92 – 1.75 (m, 2H), 1.71 – 1.53 (m, 4H), 1.51 – 1.39 (m, 4H), 1.17 – 1.03 (m, 5H), 0.86 (t, *J* = 7.2 Hz, 3H). ^{19}**F NMR** (282 MHz, $CDCl_3$) δ, ppm: -74.89 (s). ^{13}**C NMR** (75 MHz, $CDCl_3$) δ, ppm: 141.7 (C), 141.4 (C), 139.8 (C), 135.5 (C), 129.2 (CH), 128.2 (CH), 126.6 (CH), 122.0 (C), 120.4 (q, *J* = 324.5 Hz, C), 61.2 (CH), 45.9 (CH_2), 35.1 (CH_2), 32.7 (CH_2), 31.4 (CH_2), 28.0 (CH_2), 27.1 (CH_2), 26.9 (CH_2), 26.4 (CH_2), 22.1 (CH_2), 14.3 (CH_3), 11.4 (CH_3). **HRMS** (APCI, $[M+H]^+$) *m/z* calculated for $C_{23}H_{31}F_3NO_2S$: 442.2022; found 442.2015.

Enantioselectivity of the cycloadduct product was determined by chiral HPLC analysis on Chiralpak IB at rt (Hexane : *i*PrOH = 98:2, 0.5 mL/min, λ=220 nm), by performing the oxidative cleavage of the exocyclic double bond to obtain the corresponding unsaturated cyclic ketone following the procedure on page S67.

Racemic sample (*rac*-**3ea**)

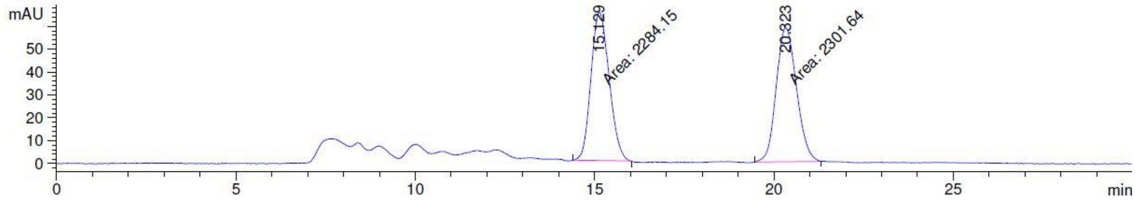

Peak #	RetTime [min]	Type	Width [min]	Area [mAU*s]	Height [mAU]	Area %
1	15.129	MM	0.5876	2284.14917	64.78648	49.8093
2	20.323	MM	0.6552	2301.63892	58.55118	50.1907

Asymmetric sample (**3ea**, 95:5 *e.r.*, 90% *ee*)

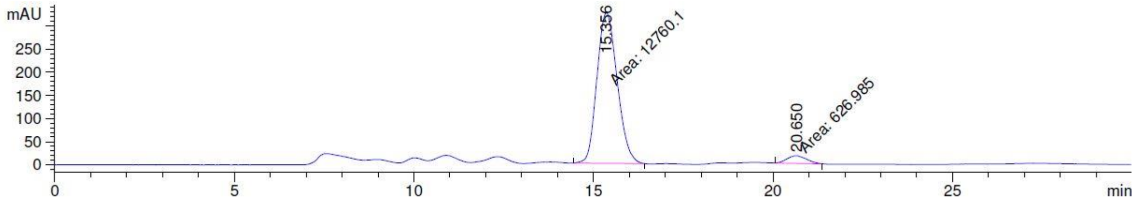

Peak #	RetTime [min]	Type	Width [min]	Area [mAU*s]	Height [mAU]	Area %
1	15.356	MM	0.6542	1.27601e4	325.09180	95.3165
2	20.650	MM	0.6399	626.98486	16.33078	4.6835

Enantioselectivity of the remaining starting material was determined by chiral HPLC analysis on Chiralpak IB at rt (Hexane : *i*PrOH = 98:2, 0.5 mL/min, λ=254 nm).

Racemic sample (*rac*-**1e**)

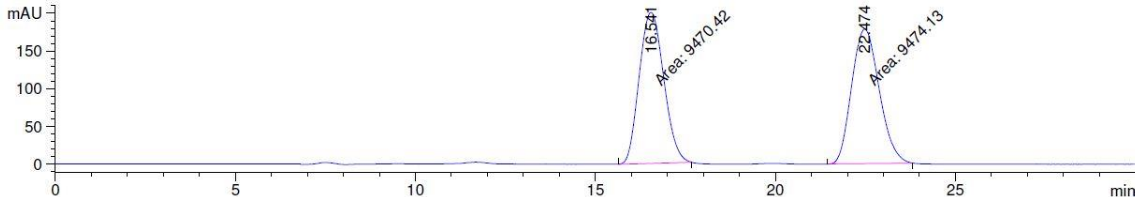

Peak #	RetTime [min]	Type	Width [min]	Area [mAU*s]	Height [mAU]	Area %
1	16.541	MM	0.7894	9470.41504	199.94191	49.9902
2	22.474	MM	0.8867	9474.12988	178.07813	50.0098

Asymmetric sample (**1e**, 100:0 *e.r.*, 99% *ee*)

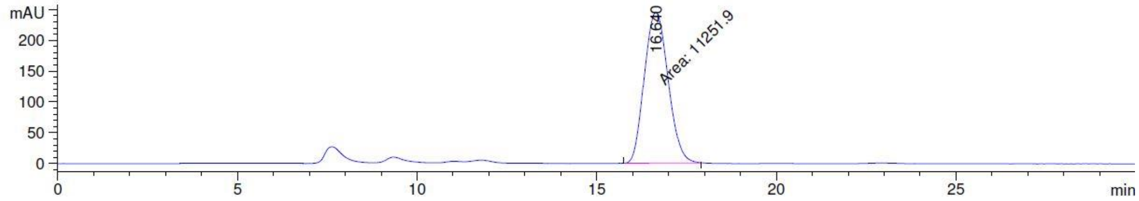

Peak #	RetTime [min]	Type	Width [min]	Area [mAU*s]	Height [mAU]	Area %
1	16.640	MM	0.7654	1.12519e4	245.02095	100.0000

***(R)*-3-cyclohexylidene-6-ethyl-5-methyl-4-(naphthalen-2-yl)-1-((trifluoromethyl)sulfonyl)-1,2,3,6-tetrahydropyridine (3fa)**

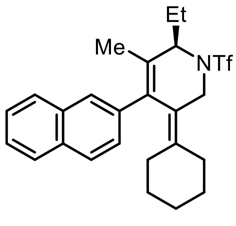

3fa

Using Boc-Phe-NHOMe as ligand at 70°C, **3fa** was obtained as a white solid (21.8 mg, 47% yield). **^{1}H NMR** (300 MHz, $CDCl_3$) δ, ppm: 7.78 – 7.68 (m, 3H), 7.49 (s, 1H), 7.44 – 7.35 (m, 2H), 7.21 – 7.15 (m, 1H), 4.40 – 4.27 (m, 2H), 4.08 (t, *J* = 7.1 Hz, 1H), 2.22 – 2.12 (m, 1H), 2.11 – 2.00 (m, 1H), 1.88 – 1.71 (m, 5H), 1.65 – 1.54 (m, 1H), 1.53 – 1.41 (m, 3H), 1.37 – 1.18 (m, 2H), 1.04 – 0.91 (m, 5H). **^{19}F NMR** (282 MHz, $CDCl_3$) δ, ppm: -74.93 (s). **^{13}C NMR** (75 MHz, $CDCl_3$) δ, ppm: 141.5 (C), 139.2 (C), 135.7 (C), 135.2 (C), 133.4 (C), 132.3 (C), 128.0 (2 x CH), 127.9 (3 x CH), 126.4 (CH), 126.0 (CH), 121.6 (C), 120.6 (q, *J* = 325.6 Hz, C), 62.9 (CH), 45.9 (CH_2), 32.7 (CH_2), 31.4 (CH_2), 27.8 (CH_2), 27.1 (CH_2), 26.5 (CH_2), 26.3 (CH_2), 20.1 (CH_3), 11.1 (CH_3). **HRMS** (APCI, $[M+H]^+$) *m/z* calculated for $C_{25}H_{29}F_3NO_2S$: 464.1866; found 464.1868.

Enantioselectivity of the cycloadduct product was determined by chiral HPLC analysis on Chiralpak IF3 at rt (Hexane, 0.2 mL/min, λ=254 nm).

Racemic sample (*rac*-**3fa**)

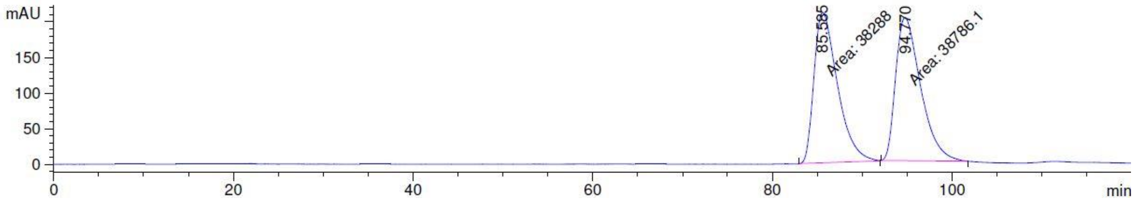

Peak #	RetTime [min]	Type	Width [min]	Area [mAU*s]	Height [mAU]	Area %
1	85.585	MM	3.0265	3.82880e4	210.84778	49.6769
2	94.770	MM	3.2156	3.87861e4	201.02774	50.3231

Asymmetric sample (**3fa**, 97:3 *e.r.*, 94% *ee*)

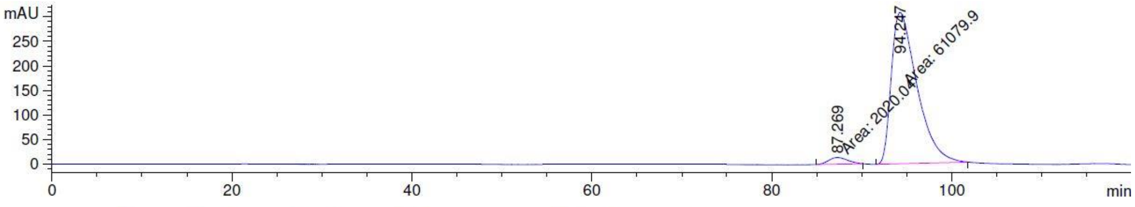

Peak #	RetTime [min]	Type	Width [min]	Area [mAU*s]	Height [mAU]	Area %
1	87.269	MM	2.5000	2020.04321	13.46700	3.2013
2	94.247	MM	3.3002	6.10799e4	308.46527	96.7987

Enantioselectivity of the remaining starting material was determined by chiral HPLC analysis on Chiralpak IA3 at rt (Hexane : *i*PrOH = 98:2, 0.5 mL/min, λ=254 nm).

Racemic sample (*rac*-**1f**)

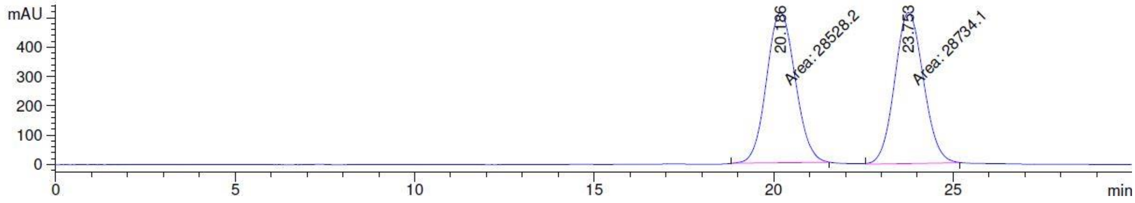

Peak #	RetTime [min]	Type	Width [min]	Area [mAU*s]	Height [mAU]	Area %
1	20.186	MM	0.9282	2.85282e4	512.24359	49.8202
2	23.753	MM	0.9299	2.87341e4	515.01093	50.1798

Asymmetric sample (**1f**, 98:2 *e.r.*, 96% *ee*

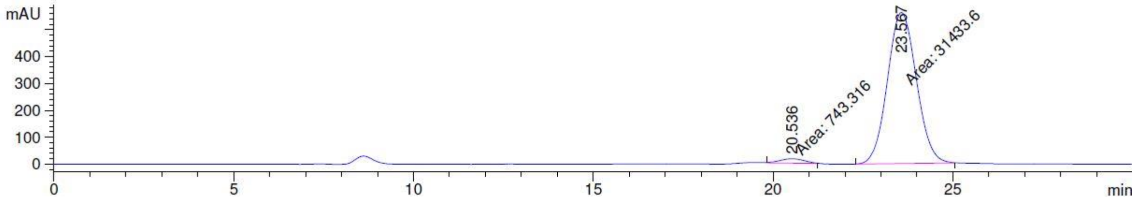

Peak #	RetTime [min]	Type	Width [min]	Area [mAU*s]	Height [mAU]	Area %
1	20.536	MM	0.7666	743.31555	16.16107	2.3101
2	23.567	MM	0.9332	3.14336e4	561.38086	97.6899

(*R*)-4-(4-bromophenyl)-3-cyclohexylidene-6-ethyl-5-methyl-1-((trifluoromethyl)sulfonyl)-1,2,3,6-tetrahydropyridine (3ga)

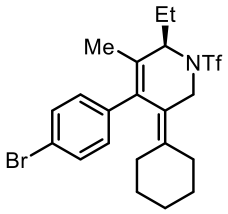

3ga

Using Boc-Phe-NHOMe as ligand at 70ºC, **3ga** was obtained as a yellowish oil (16.1 mg, 33% yield). **^{1}H NMR** (300 MHz, $CDCl_3$) δ, ppm: 7.45 (d, *J* = 8.4 Hz, 2H), 7.00 (d, *J* = 8.4 Hz, 2H), 4.40 – 4.27 (m, 2H), 4.10 (t, *J* = 7.1 Hz, 1H), 2.26 – 2.05 (m, 2H), 1.89 – 1.72 (m, 5H), 1.71 – 1.59 (m, 2H), 1.57 – 1.49 (m, 2H), 1.48 – 1.37 (m, 2H), 1.19 – 1.08 (m, 2H), 1.02 (t, *J* = 7.4 Hz, 3H). **^{19}F NMR** (282 MHz, $CDCl_3$) δ, ppm: -75.00 (s). **^{13}C NMR** (75 MHz, $CDCl_3$) δ, ppm: 141.5 (C), 140.6 (C), 135.8 (C), 134.2 (C), 131.5 (CH), 131.1 (CH), 121.3 (C), 120.6 (C), 120.5 (q, *J* = 325.0 Hz, C), 62.8 (CH), 45.7 (CH_2), 32.7 (CH_2), 31.3 (CH_2), 27.8 (CH_2), 27.1 (CH_2), 26.2 (2 x CH_2), 19.9 (CH_3), 11.0 (CH_3). **HRMS** (APCI, $[M+H]^+$) *m/z* calculated for $C_{21}H_{26}BrF_3NO_2S$: 492.0814; found 492.0816.

Enantioselectivity of the cycloadduct product was determined by chiral SFC analysis on Phenomenex Cellulose-1 at 40 ºC (CO_2 : MeOH = 99:1, 1 mL/min, λ= 254 nm).

Racemic sample (*rac*-**3ga**)

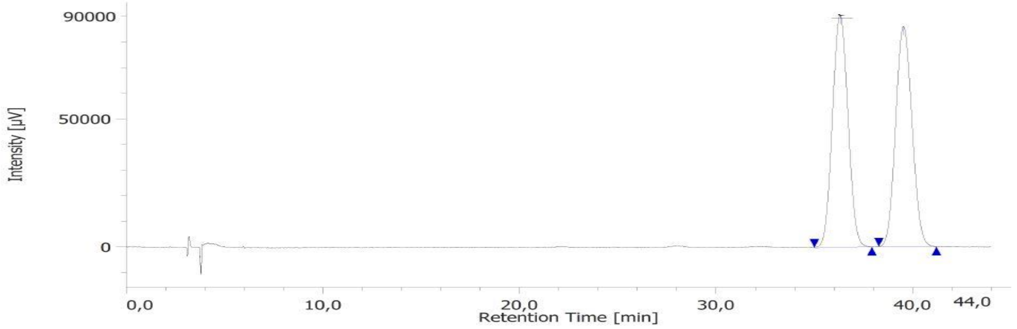

#	Peak Name	CH	tR [min]	Area [µV·sec]	Height [µV]	Area%	Height%	Quantity	NTP	Resolution	Symmetry Factor	Warning
1	Unknown	7	36.303	4991143	90558	50.055	51.322	N/A	9689	2.134	1.039	
2	Unknown	7	39.523	4980106	85892	49.945	48.678	N/A	10396	N/A	1.078	

Asymmetric sample (**3ga**, 96:4 *e.r.*, 92% *ee*)

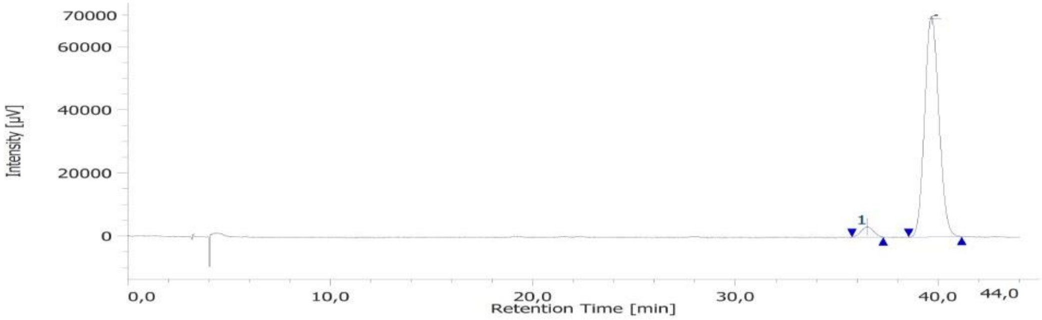

#	Peak Name	CH	tR [min]	Area [µV·sec]	Height [µV]	Area%	Height%	Quantity	NTP	Resolution	Symmetry Factor	Warning
1	Unknown	7	36.520	138553	3244	3.885	4.421	N/A	16151	2.576	0.949	
2	Unknown	7	39.663	3427723	70142	96.115	95.579	N/A	14944	N/A	1.097	

Enantioselectivity of the remaining starting material was determined by chiral HPLC analysis on Chiralpak IA3 at rt (Hexane : *i*PrOH = 98:2, 0.5 mL/min, λ=254 nm).

Racemic sample (*rac*-**1g**)

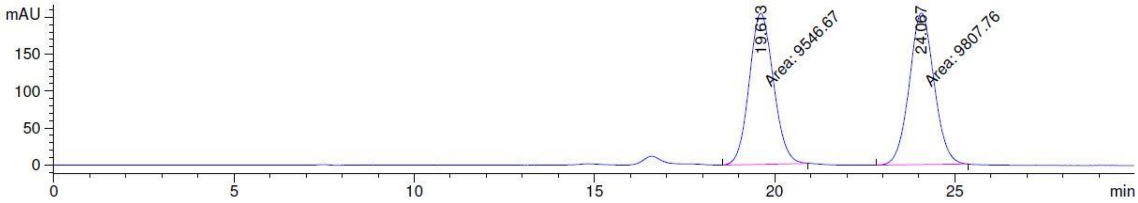

Peak #	RetTime [min]	Type	Width [min]	Area [mAU*s]	Height [mAU]	Area %
1	19.613	MM	0.7780	9546.66992	204.51718	49.3255
2	24.067	MM	0.7975	9807.75977	204.97623	50.6745

Asymmetric sample (**1g**, 100:0 *e.r.*, 99% *ee*)

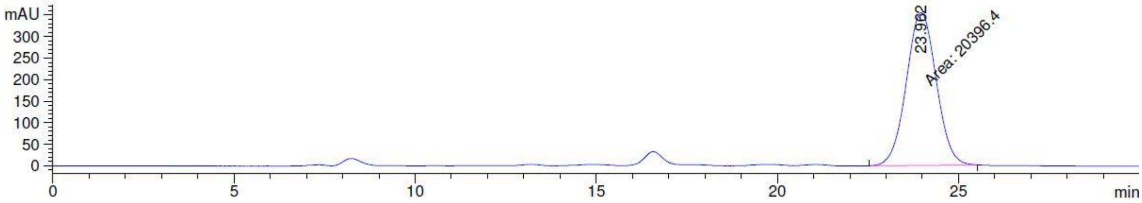

Peak #	RetTime [min]	Type	Width [min]	Area [mAU*s]	Height [mAU]	Area %
1	23.962	MM	0.9585	2.03964e4	354.67468	100.0000

(*R*)-4-cyclohexylidene-1-ethyl-2-((trifluoromethyl)sulfonyl)-1,2,3,4,5,6,7,8-octahydroisoquinoline (3ha)

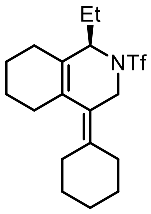

3ha

Using Boc-Phe-NHOMe as ligand at 80°C, **3ha** was obtained as a yellow oil (18.6 mg, 49%). **^{1}H NMR** (300 MHz, $CDCl_3$) δ, ppm: 4.21 (d, *J* = 13.7 Hz, 1H), 4.07 (d, *J* = 13.4 Hz, 1H), 3.87 (t, *J* = 6.3 Hz, 1H), 2.52 – 2.35 (m, 1H), 2.32 – 2.00 (m, 7H), 1.78 – 1.63 (m, 5H), 1.63 – 1.40 (m, 7H), 0.93 (t, *J* = 7.3 Hz, 3H). **^{19}F NMR** (282 MHz, $CDCl_3$) δ, ppm: -75.11 (s). **^{13}C NMR** (75 MHz, $CDCl_3$) δ, ppm: 138.2 (C), 136.0 (C), 131.8 (C), 121.9 (C), 120.5 (q, *J* = 324.8 Hz, C), 61.7 (CH), 45.9 (CH_2), 33.2 (CH_2), 31.2 (CH_2), 30.5 (CH_2), 29.5 (CH_2), 28.2 (CH_2), 28.0 (CH_2), 26.8 (CH_2), 26.6 (CH_2), 23.3 (CH_2), 22.7 (CH_2), 11.1 (CH_3). **HRMS** (APCI, $[M+H]^+$) *m/z* calculated for $C_{18}H_{27}F_3NO_2S$: 378.1709; found 378.1707.

Enantioselectivities of the cycloadduct product and the modified remaining starting material were determined by chiral HPLC analysis on Chiralpak IF3 at rt (Hexane, 0.3 mL/min, λ=254 nm).

Racemic sample (*rac*-**3ha**)

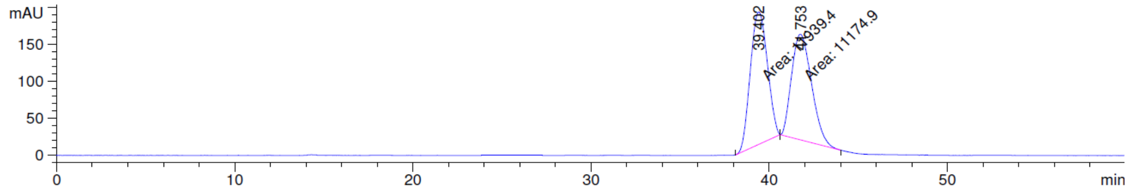

Peak #	RetTime [min]	Type	Width [min]	Area [mAU*s]	Height [mAU]	Area %
1	39.402	MM	1.1101	1.19394e4	179.25676	51.6537
2	41.753	MM	1.3018	1.11749e4	143.06543	48.3463

Asymmetric sample of the product (**3ha**, 96:4 *e.r.*, 92% *ee*)

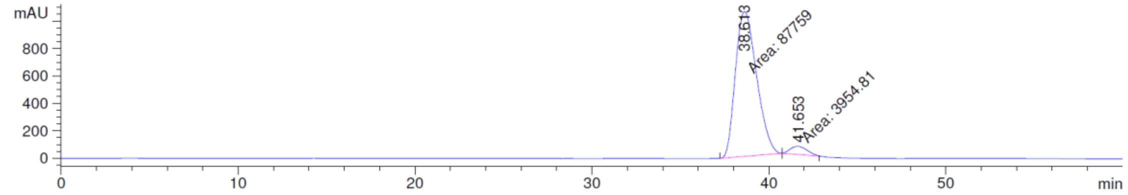

Peak #	RetTime [min]	Type	Width [min]	Area [mAU*s]	Height [mAU]	Area %
1	38.613	MM	1.3862	8.77590e4	1055.14014	95.6879
2	41.653	MM	1.1059	3954.80762	59.60394	4.3121

Asymmetric sample of the **1h** transformed in **3ha-SM**, (95:5 *e.r.*, 90% *ee*) at 80°C

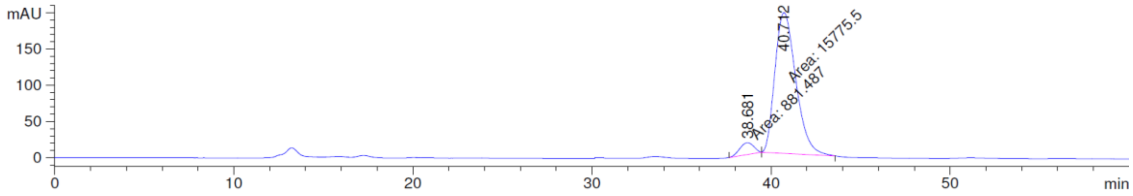

Peak #	RetTime [min]	Type	Width [min]	Area [mAU*s]	Height [mAU]	Area %
1	38.681	MM	0.9066	881.48663	16.20429	5.2920
2	40.712	MM	1.3537	1.57755e4	194.22549	94.7080

(*R*)-4-cyclohexylidene-1-isopropyl-2-((trifluoromethyl)sulfonyl)-1,2,3,4,5,6,7,8-octahydroisoquinoline (3ia)

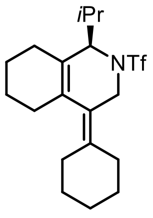

3ia

Using Boc-Phe-NHOMe as ligand at 80°C, **3ia** was obtained as a white solid (18.8 mg, 48%). ^{1}**H NMR** (300 MHz, $CDCl_3$) δ, ppm: 4.29 (d, *J* = 14.6 Hz, 1H), 4.08 (d, *J* = 14.2 Hz, 1H), 3.53 (d, *J* = 10.2 Hz, 1H), 2.60 – 2.42 (m, 1H), 2.35 – 2.13 (m, 5H), 2.13 – 1.93 (m, 3H), 1.84 – 1.70 (m, 2H), 1.69 – 1.37 (m, 9H), 1.03 (d, *J* = 6.7 Hz, 3H), 0.89 (d, *J* = 6.7 Hz, 3H). ^{19}**F NMR** (282 MHz, $CDCl_3$) δ, ppm: -75.02 (s). ^{13}**C NMR** (75 MHz, $CDCl_3$) δ, ppm: 137.6 (C), 131.3 (C), 122.0 (C), 120.5 (q, *J* = 325.5 Hz), 119,6 (C), 66.9 (CH), 46.1 (CH_2), 33.2 (CH_2), 31.5 (CH), 31.1 (CH_2), 30.8 (CH_2), 30.3 (CH_2), 28.3 (CH_2), 27.8 (CH_2), 26.6 (CH_2), 23.2 (CH_2), 22.7 (CH_2), 21.1 (CH_3), 19.8 (CH_3). **HRMS** (APCI, $[M+H]^+$) *m/z* calculated for $C_{19}H_{29}F_3NO_2S$, 392.1866; found 392.1867.

Enantioselectivities of the cycloadduct product and the modified remaining starting material were determined by chiral HPLC analysis on Chiralpak IF3 at rt (Hexane, 0.5 mL/min, λ=254 nm).

Racemic sample (*rac*-**3ia**)

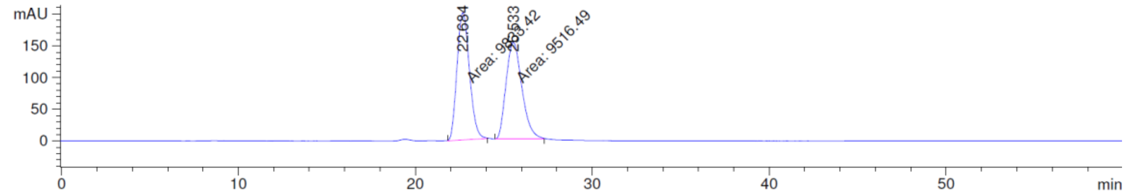

Peak #	RetTime [min]	Type	Width [min]	Area [mAU*s]	Height [mAU]	Area %
1	22.684	MM	0.8163	9833.41992	200.78348	50.8190
2	25.533	MM	1.0402	9516.48535	152.47478	49.1810

Asymmetric sample of the product (**3ia**, 95:5 *e.r.*, 90% *ee*)

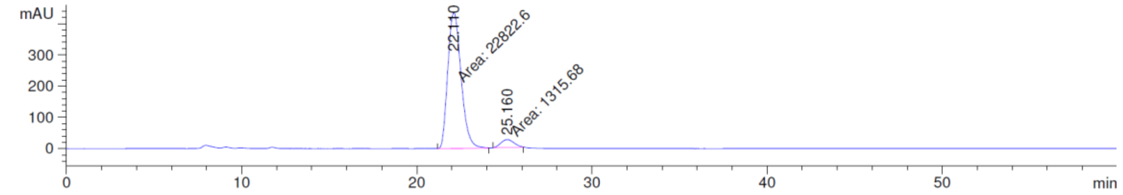

Peak #	RetTime [min]	Type	Width [min]	Area [mAU*s]	Height [mAU]	Area %
1	22.110	MM	0.8731	2.28226e4	435.65637	94.5494
2	25.160	MM	0.8708	1315.68201	25.18203	5.4506

Asymmetric sample of **1i** transformed in **3ia-SM** (94:6 *e.r.*, 88% *ee*) at 80°C

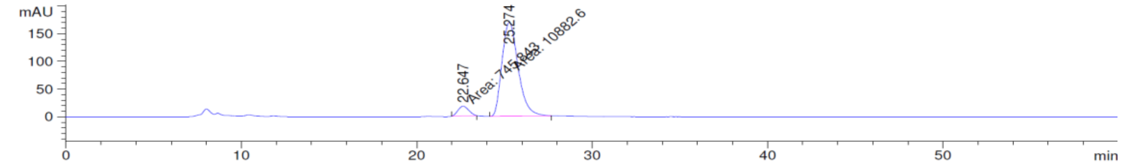

Peak #	RetTime [min]	Type	Width [min]	Area [mAU*s]	Height [mAU]	Area %
1	22.647	MM	0.7104	745.84253	17.49790	6.4139
2	25.274	MM	1.0692	1.08826e4	169.64622	93.5861

(*R*)-3-cyclohexylidene-1-((trifluoromethyl)sulfonyl)-1,2,3,5,6,7,8,8a-octahydroquinoline (3ja)

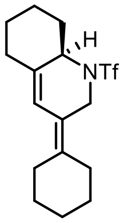

3ja

Using Boc-Phe-NHOMe as ligand at 70°C, **3ja** was obtained as a white solid (16.1 mg, 46%). **^{1}H NMR** (300 MHz, $CDCl_3$) δ, ppm: 4.69 (d, *J* = 15.2 Hz, 1H), 4.20 – 4.09 (m, 1H), 3.64 (d, *J* = 15.2 Hz, 1H), 2.45 – 2.27 (m, 2H), 2.26 – 2.03 (m, 4H), 1.95 – 1.82 (m, 2H), 1.79 – 1.47 (m, 8H), 1.39 – 1.19 (m, 2H). **^{19}F NMR** (282 MHz, $CDCl_3$) δ, ppm: -77.05 (s). **^{13}C NMR** (75 MHz, $CDCl_3$) δ, ppm: 137.5 (C), 137.3 (C), 119.9 (q, *J* = 323.7 Hz), 117.7 (C), 118.8 (CH), 58.2 (CH), 41.5 (CH_2), 35.7 (CH_2), 30.4 (CH_2), 29.6 (CH_2), 27.9 (CH_2), 27.9 (CH_2), 27.8 (CH_2), 26.7 (CH_2), 25.2(CH_2). **HRMS** (APCI, $[M+H]^+$) *m/z* calculated for $C_{16}H_{23}F_3NO_2S$, 350.1396; found 350.1399.

Enantioselectivities of the cycloadduct product and the modified remaining starting material were determined by chiral SFC analysis on Phenomenex i-Cellulose-1 at 40°C (CO_2 : MeOH = 99:1, 1 mL/min, λ=254 nm).

Racemic sample (*rac*-**3ja**)

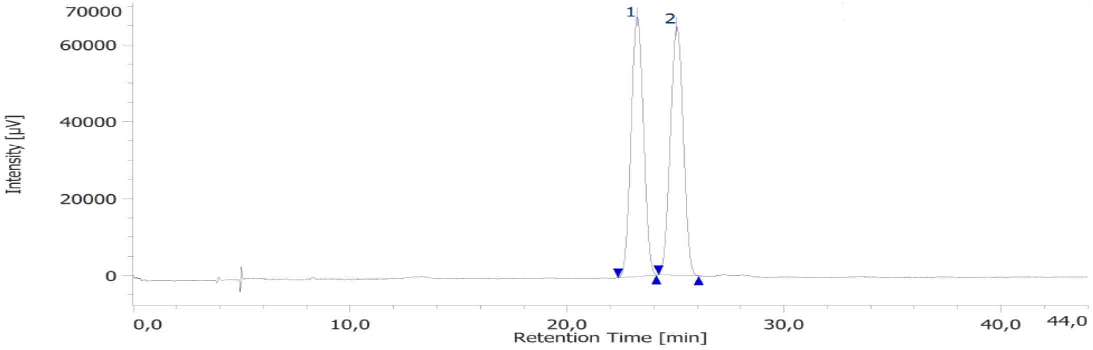

#	Peak Name	CH	tR [min]	Area [µV·sec]	Height [µV]	Area%	Height%	Quantity	NTP	Resolution	Symmetry Factor	Warning
1	Unknown	6	23,227	2694465	67413	50,164	51,048	N/A	7388	1,665	1,027	
2	Unknown	6	25,053	2676801	64645	49,836	48,952	N/A	8021	N/A	1,006	

Asymmetric sample (**3ja**, 95:5 *e.r.*, 90% *ee*)

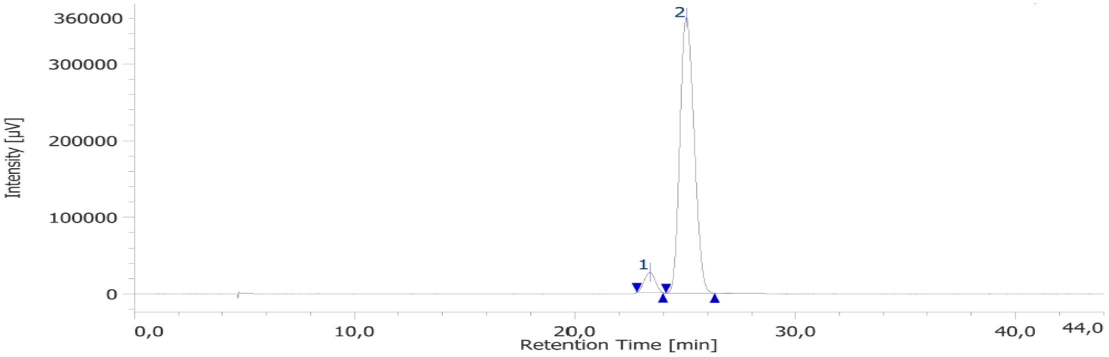

#	Peak Name	CH	tR [min]	Area [µV·sec]	Height [µV]	Area%	Height%	Quantity	NTP	Resolution	Symmetry Factor	Warning
1	Unknown	6	23,370	938200	26651	5,473	6,899	N/A	9194	1,518	1,002	
2	Unknown	6	25,030	16204820	359635	94,527	93,101	N/A	6752	N/A	1,161	

Asymmetric sample of **1j** transformed in **3ja-SM** (88:12 *e.r.*, 76% *ee*) at 70°C

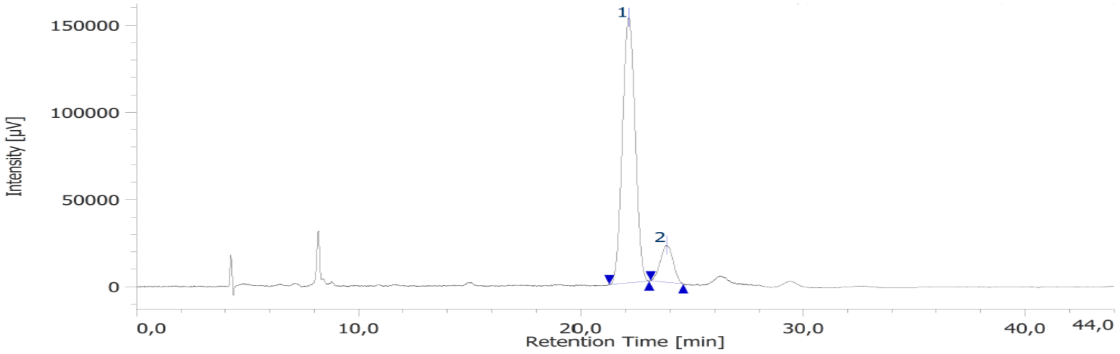

#	Peak Name	CH	tR [min]	Area [µV·sec]	Height [µV]	Area%	Height%	Quantity	NTP	Resolution	Symmetry Factor	Warning
1	Unknown	6	22,130	6066900	152167	87,731	87,725	N/A	6739	1,564	1,015	
2	Unknown	6	23,830	848432	21291	12,269	12,275	N/A	7484	N/A	1,024	

(*R*)-6-butyl-3-cyclohexylidene-5-methyl-1-((trifluoromethyl)sulfonyl)-1,2,3,6-tetrahydropyridine (3ka)

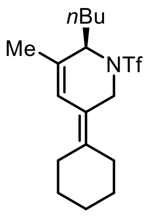

3ka

Using Boc-OH as ligand at 40°C, **3ka** was obtained as a yellow oil (13.4 mg, 37%). ^{1}**H NMR** (300 MHz, $CDCl_3$) δ, ppm: 6.32 (s, 1H), 4.70 (d, *J* = 16.4 Hz, 1H), 4.11 (dd, *J* = 9.9, 3.3 Hz, 1H), 3.80 (d, *J* = 16.4 Hz, 1H), 2.45 – 2.34 (m, 1H), 2.34 – 2.15 (m, 2H), 2.15 – 2.04 (m, 1H), 1.84 (s, 3H), 1.79 – 1.28 (m, 12H), 0.95 (t, *J* = 7.0 Hz, 3H). ^{19}**F NMR** (282 MHz, $CDCl_3$) δ, ppm: -76.85 (s). ^{13}**C NMR** (75 MHz, $CDCl_3$) δ, ppm: 136.6 (C), 133.6 (C), 119.8 (q, *J* = 323.9 Hz, C), 119.7 (CH), 118.3 (C), 117.7 (C), 59.2 (CH), 41.2 (CH_2), 31.3 (CH_2), 30.5 (CH_2), 29.4 (CH_2), 28.2 (CH_2), 28.0 (CH_2), 27.8 (CH_2), 26.7 (CH_2), 22.4 (CH_2), 21.4 (CH_3), 13.9 (CH_3). **HRMS** (APCI, $[M+H]^+$) *m/z* calculated for $C_{17}H_{27}F_3NO_2S$: 366.1709; found 366.1706.

Enantioselectivities of the cycloadduct product and the modified remaining starting material were determined by chiral SFC analysis on Phenomenex Cellulose-1 at 40°C (CO_2, 1 mL/min, λ=254 nm).

Racemic sample (*rac*-**3ka**)

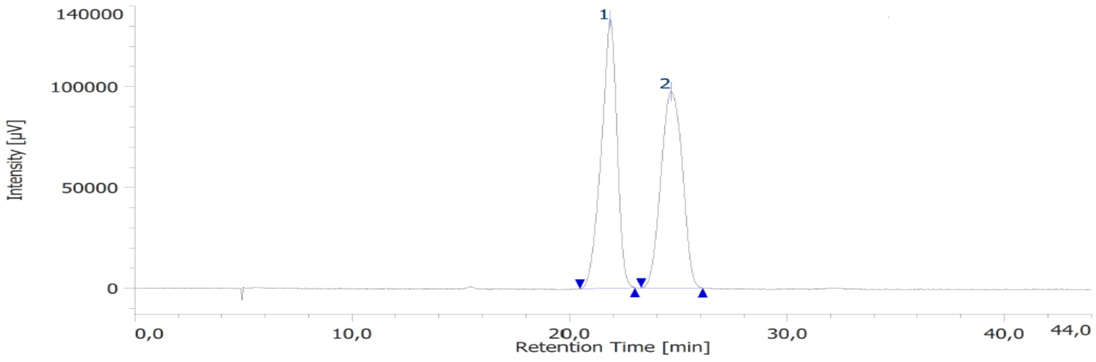

#	Peak Name	CH	tR [min]	Area [µV·sec]	Height [µV]	Area%	Height%	Quantity	NTP	Resolution	Symmetry Factor	Warning
1	Unknown	10	21,837	6713537	133220	50,076	57,770	N/A	4450	1,758	0,863	
2	Unknown	10	24,657	6693095	97382	49,924	42,230	N/A	2675	N/A	1,001	

Asymmetric sample (**3ka**, 92:8 *e.r.*, 84% *ee*)

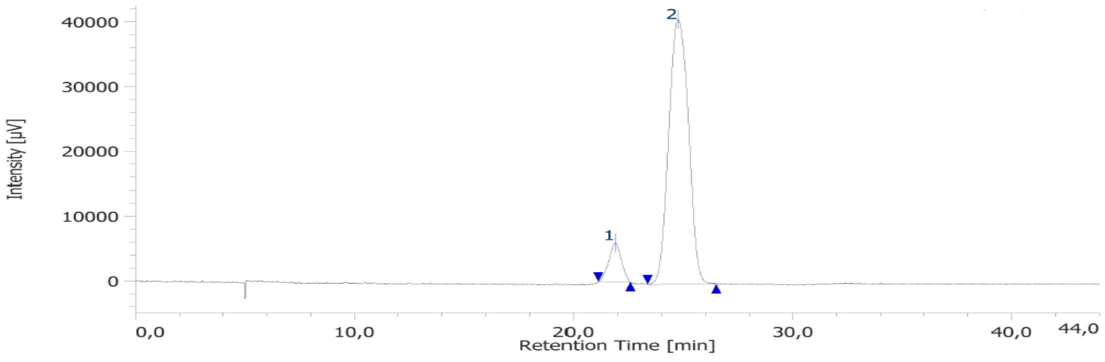

#	Peak Name	CH	tR [min]	Area [µV·sec]	Height [µV]	Area%	Height%	Quantity	NTP	Resolution	Symmetry Factor	Warning
1	Unknown	10	21,887	237661	5984	8,476	12,764	N/A	6978	2,065	0,926	
2	Unknown	10	24,747	2566310	40894	91,524	87,236	N/A	3275	N/A	1,041	

Asymmetric sample of **1k** transformed in **3ka-SM** (91:9 *e.r.*, 82% *ee*) at 60°C

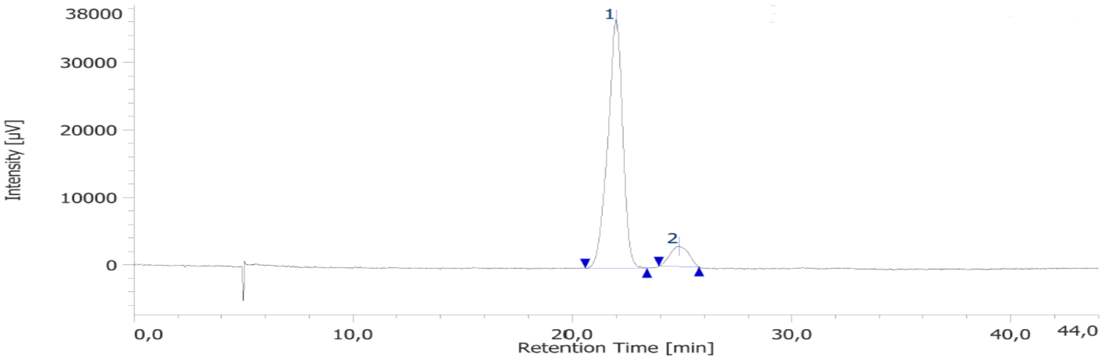

#	Peak Name	CH	tR [min]	Area [µV·sec]	Height [µV]	Area%	Height%	Quantity	NTP	Resolution	Symmetry Factor	Warning
1	Unknown	10	21,963	1755542	36859	90,622	92,416	N/A	5159	1,971	0,888	
2	Unknown	10	24,847	181669	3025	9,378	7,584	N/A	3379	N/A	0,978	

(*R*,*E*)-3-cyclohexylidene-4-ethylidene-5-methyl-1-((trifluoromethyl)sulfonyl)piperidine (3la)

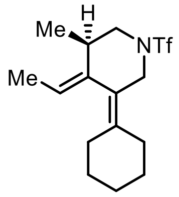

3la

Using Boc-Phe-OH as ligand at 40ºC, **3la** was obtained as a colourless oil (9.4 mg, 28%). **^{1}H NMR** (300 MHz, $CDCl_3$) δ, ppm: 5.25 (q, *J* = 6.9 Hz, 1H), 4.67 – 4.56 (m, 1H), 3.63 – 3.37 (m, 3H), 3.08 – 2.96 (m, 1H), 2.33 – 2.20 (m, 3H), 2.18 – 2.08 (m, 1H), 1.69 (d, *J* = 6.9 Hz, 3H), 1.62 – 1.46 (m, 6H), 1.00 (d, *J* = 6.8 Hz, 3H). **^{19}F NMR** (282 MHz, $CDCl_3$) δ, ppm: -75.72 (s). **^{13}C NMR** (75 MHz, $CDCl_3$) δ, ppm: 139.4 (C), 138.2 (C), 123.4 (C), 122.8 (CH), 120.3 (q, *J* = 323.5 Hz, C), 52.7 (CH_2), 49.6 (CH_2), 32.1 (CH), 31.7 (CH_2), 30.5 (CH_2), 28.9 (CH_2), 28.3 (CH_2), 26.9 (CH_2), 16.4 (CH_3), 12.9 (CH_3). **HRMS** (APCI, $[M+H]^+$) *m/z* calculated for $C_{15}H_{23}F_3NO_2S$: 338.1396; found 338.1394.

Enantioselectivity of the cycloadduct product and the modified remaining starting material were determined by chiral HPLC analysis on Chiralpak IF3 at rt (Hexane, 0.5 mL/min, λ=220 nm).

Racemic Sample (*rac*-**3la**)

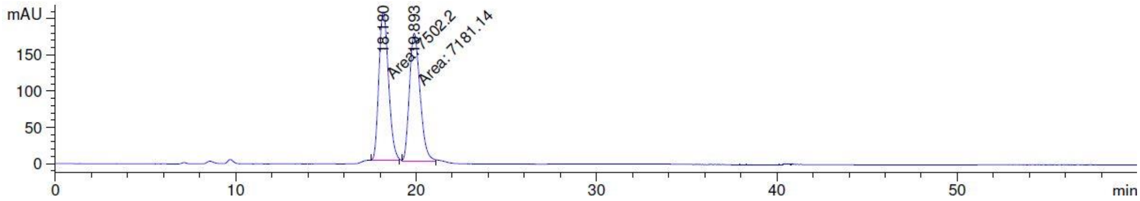

Peak #	RetTime [min]	Type	Width [min]	Area [mAU*s]	Height [mAU]	Area %
1	18.180	MM	0.6142	7502.19873	203.56342	51.0933
2	19.893	MM	0.6807	7181.13574	175.81841	48.9067

Asymmetric sample (**3la**, 93:7 *e.r.*, 86% *ee*)

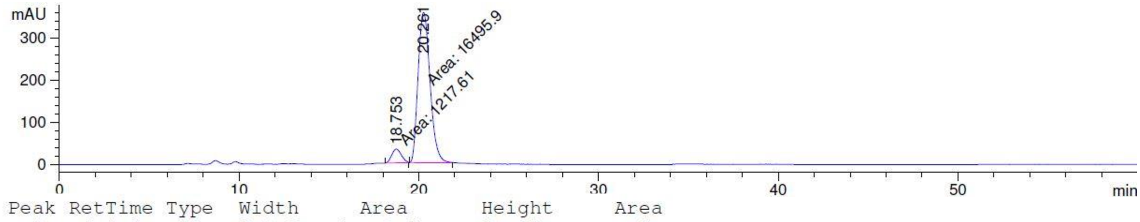

Peak #	RetTime [min]	Type	Width [min]	Area [mAU*s]	Height [mAU]	Area %
1	18.753	MM	0.6193	1217.61194	32.76723	6.8739
2	20.261	MM	0.7706	1.64959e4	356.78619	93.1261

Asymmetric sample of **1l** transformed in **3la-SM** (76:24 *e.r.*, 52% *ee*) at 60ºC.

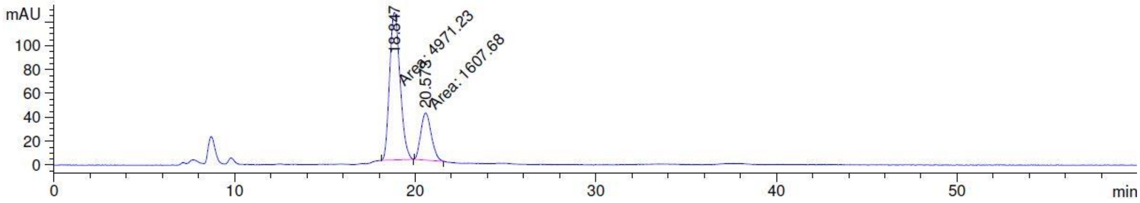

Peak #	RetTime [min]	Type	Width [min]	Area [mAU*s]	Height [mAU]	Area %
1	18.847	MM	0.6694	4971.22559	123.76408	75.5631
2	20.573	MM	0.6783	1607.68335	39.50513	24.4369

(*R*)-4-cyclohexylidene-2-((trifluoromethyl)sulfonyl)-1,2,3,4,6,7,8,8a-octahydroisoquinoline (3ma)

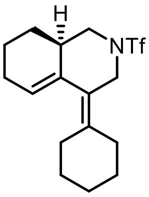

3ma

Using Boc-Phe-OH as ligand at 40°C, **3ma** was obtained as a white solid (17.1 mg, 49%). **^{1}H NMR** (300 MHz, $CDCl_3$) δ, ppm: 5.62 – 5.48 (m, 1H), 4.77 (d, *J* = 13.6 Hz, 1H), 3.92 (dd, *J* = 12.8, 5.6 Hz, 1H), 3.40 (d, *J* = 13.5 Hz, 1H), 3.40 (d, *J* = 13.5 Hz, 1H), 2.40 – 2.30 (m, 2H), 2.30 – 2.18 (m, 3H), 2.16 – 2.03 (m, 2H), 1.92 – 1.80 (m, 1H), 1.78 – 1.65 (m, 1H), 1.64 – 1.44 (m, 7H), 1.30 – 1.06 (m, 1H). **^{19}F NMR** (282 MHz, $CDCl_3$) δ, ppm: -76.52 (s). **^{13}C NMR** (75 MHz, $CDCl_3$) δ, ppm: 138.2 (C), 134.9 (C), 127.2 (CH), 124.6 (C), 120.2 (q, *J* = 323.4 Hz, C), 53.4 (CH_2), 48.9 (CH_2), 37.7 (CH), 31.6 (CH_2), 30.7 (CH_2), 28.8 (CH_2), 28.2 (CH_2), 27.2 (CH_2), 26.9 (CH_2), 25.6 (CH_2), 20.8 (CH_2). **HRMS** (APCI, $[M+H]^+$) *m/z* calculated for $C_{16}H_{23}F_3NO_2S$: 350.1396; found 350.1393.

Enantioselectivities of the cycloadduct product and the modified remaining starting material were determined by chiral HPLC analysis on Chiralpak IF3 at rt (Hexane, 0.3 mL/min, λ=220 nm).

Racemic sample (*rac*-**3ma**)

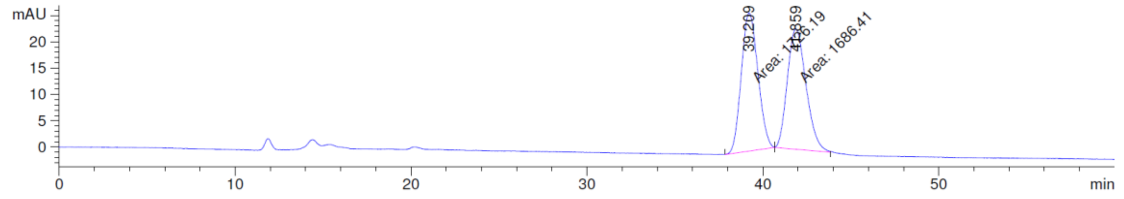

Peak #	RetTime [min]	Type	Width [min]	Area [mAU*s]	Height [mAU]	Area %
1	39.209	MM	1.0965	1726.19202	26.23886	50.5828
2	41.859	MM	1.2304	1686.41492	22.84294	49.4172

Asymmetric sample of the product (**3ma**, 95:5 *e.r.*, 90% *ee*)

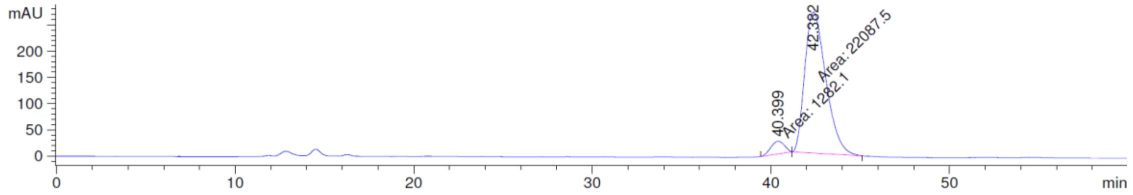

Peak #	RetTime [min]	Type	Width [min]	Area [mAU*s]	Height [mAU]	Area %
1	40.399	MM	0.8880	1282.10352	24.06380	5.4862
2	42.382	MM	1.3708	2.20875e4	268.55194	94.5138

Asymmetric sample of **1m** transformed in **3ma-SM**, 98:2 *e.r.*, 96% *ee* at 60°C

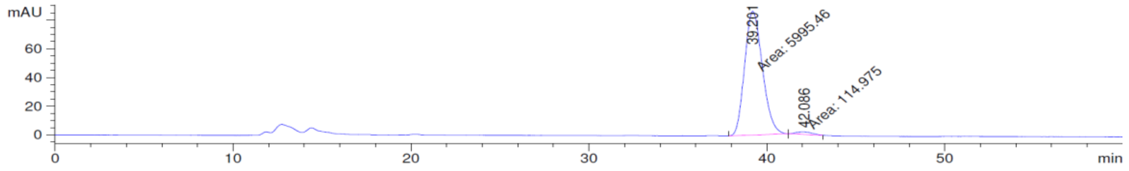

Peak #	RetTime [min]	Type	Width [min]	Area [mAU*s]	Height [mAU]	Area %
1	39.201	MM	1.1580	5995.45703	86.28731	98.1184
2	42.086	MM	1.0274	114.97482	1.86509	1.8816

(*R*,*Z*)-4-(cyclohexylmethylene)-1-ethyl-2-((trifluoromethyl)sulfonyl)-1,2,3,4,5,6,7,8-octahydroisoquinoline (3hc)

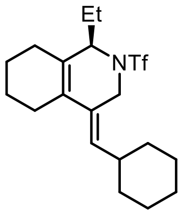

3hc

Using Boc-Phe-NHOMe as ligand at 80°C, **3hc** was obtained as a yellow oil (15.5 mg, 40%) and as a single diastereoisomer. **^{1}H NMR** (300 MHz, $CDCl_3$) δ, ppm: 5.31 (d, *J* = 9.2 Hz, 1H), 4.67 (d, *J* = 16.9 Hz, 1H), 3.99 – 3.87 (m, 2H), 2.27 – 2.09 (m, 3H), 2.09 – 1.92 (m, 1H), 1.83 – 1.50 (m, 11H), 1.43 – 1.10 (m, 5H), 1.10 – 1.00 (m, 4H). **^{19}F NMR** (282 MHz, $CDCl_3$) δ, ppm: -76.83. **^{13}C NMR** (75 MHz, $CDCl_3$) δ, ppm: 132.5 (C), 129.4 (CH), 126.9 (C), 126.9 (C) 119.87 (q, *J* = 323.9 Hz, C), 60.9 (CH), 41.4 (CH_2), 36.7 (CH), 33.0 (CH_2), 32.8 (CH_2), 28.8 (CH_2), 25.9 (CH_2), 25.8 (CH_2), 25.7 (CH_2), 24.3 (CH_2), 22.7(CH_2), 22.3 (CH_2), 11.0 (CH_3). **HRMS** (APCI, $[M+H]^+$) *m/z* calculated for $C_{19}H_{29}F_3NO_2S$: 392.1866; found 392.1863.

The stereochemistry of the exocyclic double bond was assigned based on the observed nOe between H_a (5.31 ppm) and H_b (2.2 ppm, 5.8%) and the absence of nOe between H_a (5.31 ppm) and H_c (4.67 ppm).

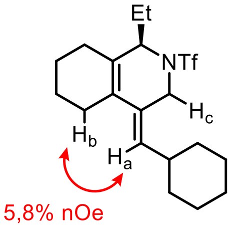

Enantioselectivities of the cycloadduct product and the modified remaining starting material were determined by chiral HPLC analysis on Chiralpak OZ-H at rt (Hexane, 0.5 mL/min, λ=254 nm).

Racemic sample (*rac*-**3hc**)

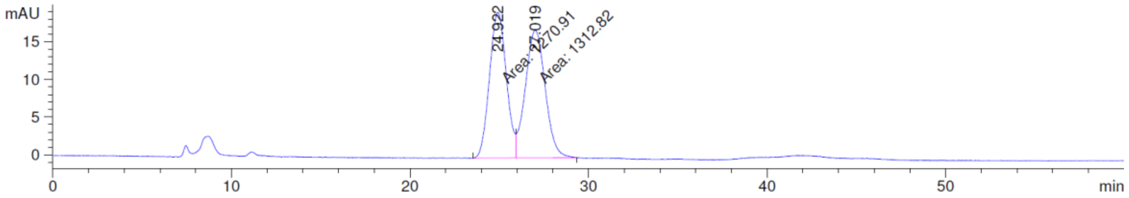

Peak #	RetTime [min]	Type	Width [min]	Area [mAU*s]	Height [mAU]	Area %
1	24.922	MF	1.0953	1270.91138	19.33822	49.1890
2	27.019	FM	1.2899	1312.82178	16.96325	50.8110

Asymmetric sample of the product (**3hc**, 95:5 *e.r.*, 90% *ee*)

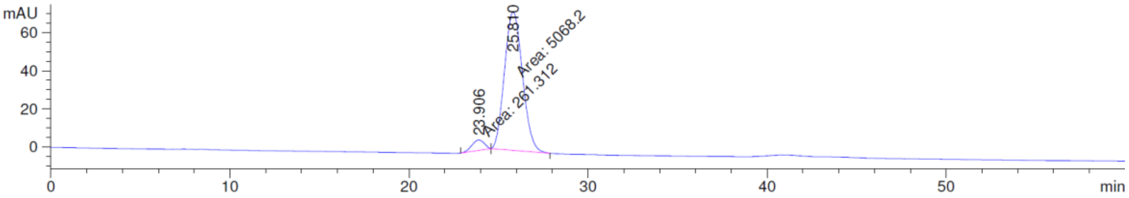

Peak #	RetTime [min]	Type	Width [min]	Area [mAU*s]	Height [mAU]	Area %
1	23.906	MM	0.8134	261.31241	5.35462	4.9031
2	25.810	MM	1.1650	5068.19629	72.50867	95.0969

Asymmetric sample of **1h** transformed in **3hc-SM** (88:12 *e.r.*, 76% *ee)* at 80°C

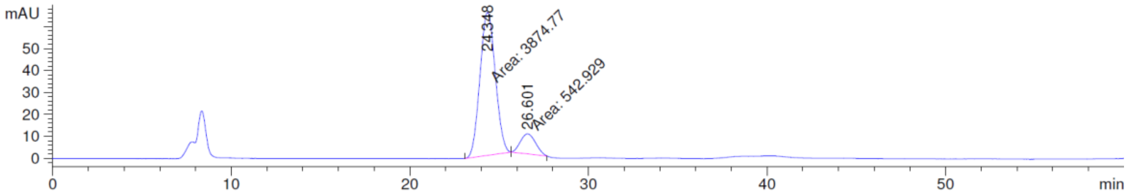

Peak #	RetTime [min]	Type	Width [min]	Area [mAU*s]	Height [mAU]	Area %
1	24.348	MM	0.9928	3874.76538	65.04765	87.7101
2	26.601	MM	0.9941	542.92853	9.10289	12.2899

4. Synthetic Manipulations of Cycloadducts and Enantioenriched Starting Materials

4.1. Cycloadducts

Hydrogenation of the exocyclic double bond

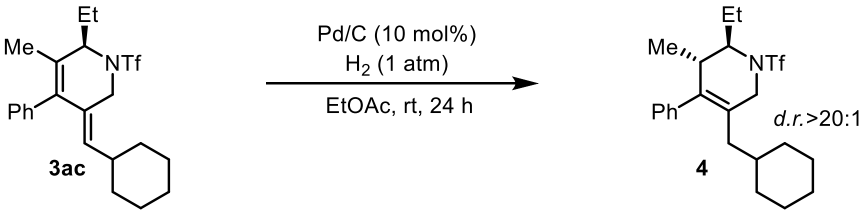

According to the literature,[6] a nitrogen flushed Schlenk was charged with compound **3ac** (42.8 mg, 0.10 mmol), Pd/C (21.3 mg, 0.010 mmol) and ethyl acetate (0.9 mL, 0.12 M). A H_2 balloon was bubbled and the resulting suspension was stirred at rt for 24h, until TLC indicates full conversion of starting material, under H_2 atmosphere. The mixture was filtered over celite, the filtrate was concentrated, and the obtained residue was purified by column chromatography on silica gel (0.5% Et_2O/hexanes) to afford **4** as a colourless oil (39.3 mg, 91% yield).

(2*R*)-5-(cyclohexylmethyl)-2-ethyl-3-methyl-4-phenyl-1-((trifluoromethyl)sulfonyl)-1,2,3,6-tetrahydropyridine (4)

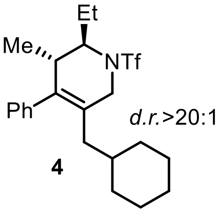

4 was obtained as a colourless oil (39.3 mg, 91% yield). **^{1}H NMR** (300 MHz, $CDCl_3$) δ, ppm: 7.40 – 7.25 (m, 3H), 7.13 – 6.96 (m, 2H), 4.12 (d, *J* = 17.3 Hz, 1H), 3.81 (t, *J* = 7.5 Hz, 1H), 3.74 (d, *J* = 17.4 Hz, 1H), 2.46 – 2.37 (m, 1H), 1.97 – 1.52 (m, 10H), 1.34 – 1.14 (m, 3H), 1.13 – 1.02 (m, 6H), 0.80 – 0.53 (m, 2H). **^{19}F NMR** (282 MHz, $CDCl_3$) δ, ppm: -76.19 (s). **^{13}C NMR** (75 MHz, $CDCl_3$) δ, ppm: 141.5 (C), 136.9 (C), 129.1 (CH), 128.4 (CH), 126.9 (CH), 125.8 (C), 120.3 (q, *J* = 322.6 Hz, C), 61.3 (CH), 44.6 (CH_2), 39.4 (CH), 39.0 (CH_2), 36.2 (CH), 33.6 (CH_2), 33.0 (CH_2), 26.4 (CH_2), 26.3 (CH_2), 26.3 (CH_2), 25.3 (CH_2), 18.8 (CH_3), 11.3 (CH_3). **HRMS** (APCI, $[M+H]^+$) *m/z* calculated for $C_{22}H_{31}F_3NO_2S$: 430.2022; found 430.2021.

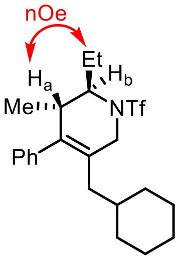

The stereochemistry of the product **4** was assigned based on the nOe (NOESY experiment) of the CH_2 of the ehyl group and H_a as well as the J = 0 Hz between H_a and H_b.

The enantiomeric excess of compound **4** was determined by chiral HPLC analysis on Chiralpak IF3 at rt (Hexane, 0.2 mL/min, λ=210 nm).

Racemic Sample (*rac*-**4**)

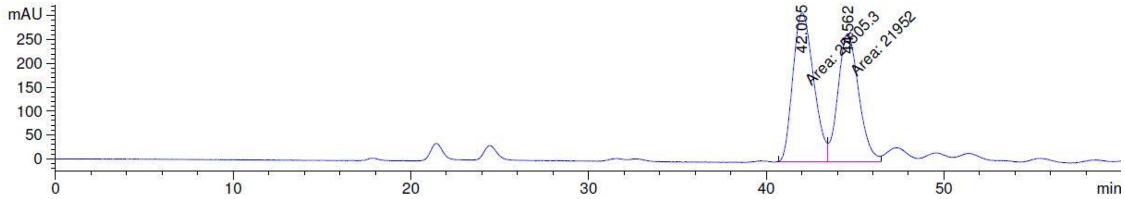

Peak #	RetTime [min]	Type	Width [min]	Area [mAU*s]	Height [mAU]	Area %
1	42.005	MF	1.3587	2.55053e4	312.87000	53.7437
2	44.562	FM	1.3596	2.19520e4	269.10062	46.2563

Asymmetric sample (**4**, 96:4 *e.r.*, 92% *ee*)

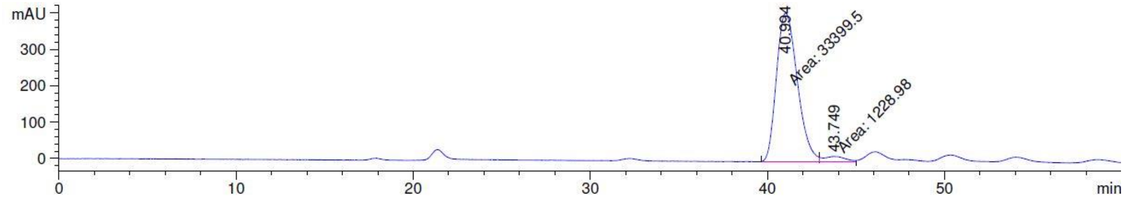

Peak #	RetTime [min]	Type	Width [min]	Area [mAU*s]	Height [mAU]	Area %
1	40.994	MF	1.3642	3.33995e4	408.05023	96.4510
2	43.749	FM	1.4027	1228.97522	14.60252	3.5490

Oxidative cleavage of the exocyclic double bond

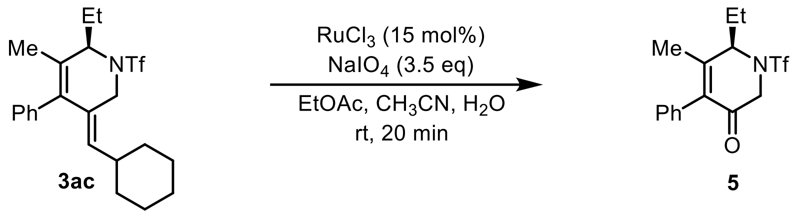

According to the literature,[7] a solution of $NaIO_4$ (74.9 mg, 0.35 mmol) in water (0.6 mL, 0.17 M) was added to a solution of $RuCl_3$ (3.1 mg, 0.015 mmol) in MeCN (0.8 mL, 0.13 M). This mixture was stirred 2 minutes and then a solution of the compound **3ac** (42.8 mg, 0.1 mmol) in EtOAc (0.8 mL, 0.13 M) was added. The mixture was stirred for 20 minutes until TLC indicates complete consumption of the starting material. $MgSO_4$ was then added and the resulting heterogeneous mixture was washed with EtOAc. The resulting organic phase was concentrated in vacuo and the crude residue was purified by column chromatography on silica gel (5 to 10% Et_2O/hexanes) to yield the ketone **5** as a yellow oil (22.9 mg, 66% yield).

(*R*)-6-ethyl-5-methyl-4-phenyl-1-((trifluoromethyl)sulfonyl)-1,6-dihydropyridin-3(2*H*)-one (5)

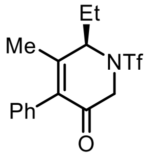

5

5 was obtained as a yellow oil (22.9 mg, 66% yield). ^{1}**H NMR** (300 MHz, $CDCl_3$) δ, ppm: 7.44 – 7.34 (m, 3H), 7.06 – 7.01 (m, 2H), 4.50 (m, 1H), 4.39 (dd, *J* = 10.6, 3.6 Hz, 1H), 4.14 – 4.05 (m, 1H), 2.04 – 1.77 (m, 5H), 1.19 (t, *J* = 7.4 Hz, 3H). ^{19}**F NMR** (282 MHz, $CDCl_3$) δ, ppm: -76.10 (s). ^{13}**C NMR** (75 MHz, $CDCl_3$) δ, ppm: 189.0 (C), 156.7 (C), 136.1 (C), 132.9 (C), 129.6 (CH), 128.6 (CH), 128.3 (CH), 120.2 (q, *J* = 323.3 Hz, C), 61.6 (CH), 49.2 (CH_2), 24.4 (CH_2), 20.2 (CH_3), 11.6 (CH_3). **HRMS** (APCI, $[M+H]^+$) *m/z* calculated for $C_{15}H_{17}F_3NO_3S$: 348.0876; found 348.0873.

The enantiomeric excess of ketone **5** was determined by chiral HPLC analysis on Chiralpak OZ-H at rt (Hexane:^{i}PrOH = 98:2, 0.5 mL/min, λ=254 nm).

Racemic Sample (*rac*-**5**)

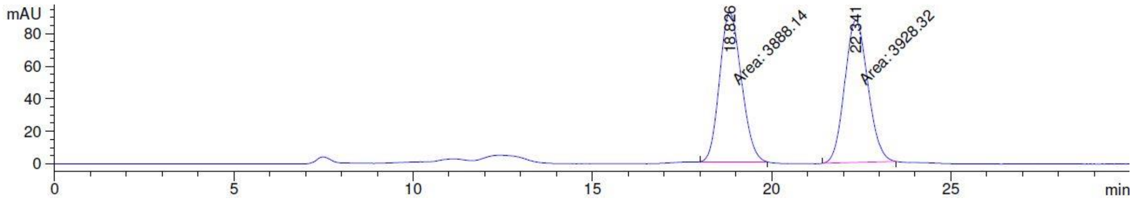

Peak #	RetTime [min]	Type	Width [min]	Area [mAU*s]	Height [mAU]	Area %
1	18.826	MM	0.7043	3888.13989	92.00770	49.7430
2	22.341	MM	0.7475	3928.31860	87.59303	50.2570

Asymmetric sample (**5**, 98:2 *e.r.*, 96% *ee*)

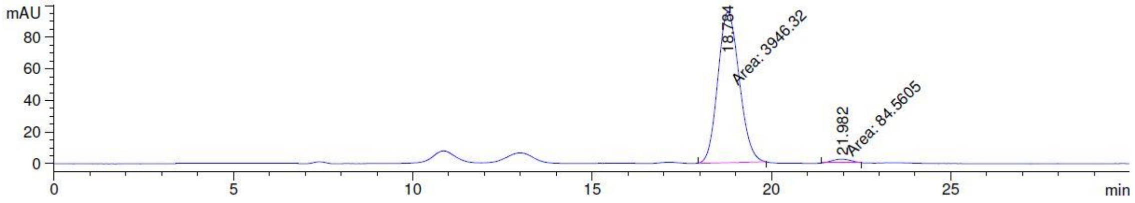

Peak #	RetTime [min]	Type	Width [min]	Area [mAU*s]	Height [mAU]	Area %
1	18.784	MM	0.6913	3946.31909	95.14167	97.9022
2	21.982	MM	0.5942	84.56052	2.37199	2.0978

Deprotection of the *N*-triflyl moiety

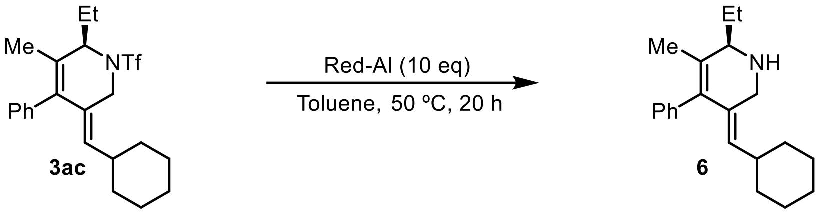

According to the literature,[8] to a stirred solution of **3ac** (42.8 mg, 0.1 mmol) in freshly distilled toluene (1.0 mL, 0.1 M) at 0°C and under argon was added sodium bis(2-methoxyethoxy)-aluminum hydride (60 wt. % in toluene, 0. 34 mL, 1.0 mmol). The resulting mixture was stirred for 20h at 50 °C and, after completion, it was cooled to 0°C and quenched by addition of 5% aq. NH_4Cl (5 mL). This mixture was extracted with dichloromethane (3 x 5 mL), dried over Na_2SO_4 and concentrated under vacuum. The obtained residue was purified by column chromatography on silica gel (DCM/MeOH/Et_3N 98:1:1 to 96:2:2) to afford the amine **6** as a yellowish oil (29.5 mg, 99% yield).

(*R*,*Z*)-3-(cyclohexylmethylene)-6-ethyl-5-methyl-4-phenyl-1,2,3,6-tetrahydropyridine (6)

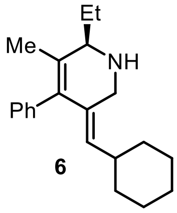

6

6 was obtained as a yellowish oil (29.5 mg, 99% yield). **^{1}H NMR** (300 MHz, $CDCl_3$) δ, ppm: 7.36 – 7.20 (m, 3H), 7.09 – 7.00 (m, 2H), 4.57 (d, *J* = 9.3 Hz, 1H), 3.79 (d, *J* = 14.7 Hz, 1H), 3.54 (d, *J* = 14.7 Hz, 1H), 3.32 (dd, *J* = 8.6, 3.1 Hz, 1H), 2.90 – 2.78 (m, 1H), 2.21 – 2.08 (m, 1H), 1.83 – 1.70 (m, 2H), 1.68 – 1.50 (m, 6H), 1.45 (s, 3H), 1.33 – 1.13 (m, 4H), 1.02 (t, *J* = 7.4 Hz, 3H). **^{13}C NMR** (75 MHz, $CDCl_3$) δ, ppm: 140.1 (C), 135.8 (C), 134.0 (C), 132.7 (CH), 132.6 (C), 130.3 (CH), 128.0 (CH), 126.4 (CH), 59.4 (CH), 42.0 (CH_2), 36.9 (CH), 33.3 (CH_2), 33.1 (CH_2), 26.1 (CH_2), 26.0 (CH_2), 26.0 (CH_2), 24.9 (CH_2), 18.6 (CH_3), 10.6 (CH_3). **HRMS** (APCI, $[M+H]^+$) *m/z* calculated for $C_{21}H_{30}N$: 296.2373; found 296.2377.

For measurement of the enantiomeric excess of the free amine **6** the following transformation has been applied:

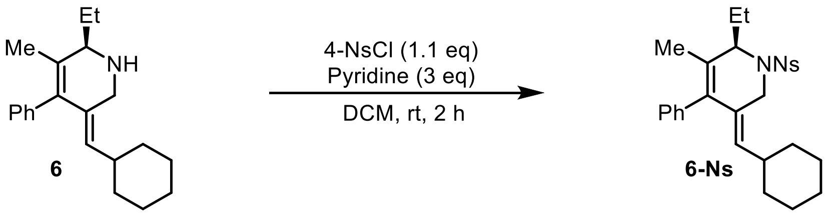

According to the literature,[9] to a solution of the deprotected tetrahydropyridine **6** (32.0 mg, 0.11 mmol) and 4-nitrobenzenesulfonyl chloride (26.4 mg, 0.12 mmol) in dichloromethane (0.4 mL, 0.3 M) under Ar atmosphere was added pyridine (26 µl, 0.33 mmol). The reaction was stirred at room temperature until consumption of the starting material and, after completion, the reaction was quenched with aqueous HCl (10%). The resulting mixture was extracted three times with dichloromethane and the combined organic layers were washed with NaCl (sat.) and dried over anhydrous Na_2SO_4. Evaporation of the solvent followed by purification by flash column chromatography on silica gel (20% Et_2O/hexanes) afforded the *N*-nosyl tetrahydropyridine **6-Ns** as a white solid (23.4 mg, 45% yield).

(*R*,*Z*)-3-(cyclohexylmethylene)-6-ethyl-5-methyl-1-((4-nitrophenyl)sulfonyl)-4-phenyl-1,2,3,6-tetrahydropyridine (6-Ns)

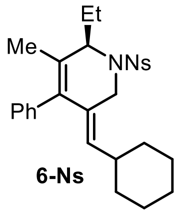

6-Ns

6-Ns was obtained as a white solid (23.4 mg, 45% yield). **^{1}H NMR** (300 MHz, $CDCl_3$) δ, ppm: 8.27 (d, *J* = 8.8 Hz, 2H), 7.95 (d, *J* = 8.8 Hz, 2H), 7.20 – 7.12 (m, 3H), 6.38 – 6.14 (m, 2H), 4.85 (d, *J* = 17.3 Hz, 1H), 4.44 (d, *J* = 9.4 Hz, 1H), 4.10 (dd, *J* = 10.6, 2.9 Hz, 1H), 4.01 – 3.91 (m, 1H), 2.31 – 2.14 (m, 1H), 1.91 – 1.76 (m, 1H), 1.75 – 1.54 (m, 6H), 1.48 – 1.38 (m, 1H), 1.32 (s, 3H), 1.30 – 1.22 (m, 1H), 1.15 (t, *J* = 7.3 Hz, 3H), 1.03 – 0.75 (m, 3H). **^{13}C NMR** (75 MHz, $CDCl_3$) δ, ppm: 150.1 (C), 146.5 (C), 137.7 (C), 135.7 (CH), 134.7 (C), 130.9 (C), 129.2 (CH), 128.7 (CH), 128.3 (CH), 127.3 (C), 126.9 (CH), 123.9 (CH), 60.6 (CH), 40.4 (CH_2), 37.0 (CH), 32.9 (CH_2), 32.9 (CH_2), 25.9 (CH_2), 25.8 (CH_2), 25.8 (CH_2), 24.8 (CH_2), 19.4 (CH_3), 11.4 (CH_3). **HRMS** (APCI, $[M+H]^+$) *m/z* calculated for $C_{27}H_{33}N_2O_4S$: 481.2156; found 481.2160.

The enantiomeric excess of compound **6-Ns** was determined by chiral HPLC analysis on Chiralpak IA3 at rt (Hexane:^{i}PrOH = 99:1, 0.5 mL/min, λ=220 nm).

Racemic Sample (*rac*-**6-Ns**)

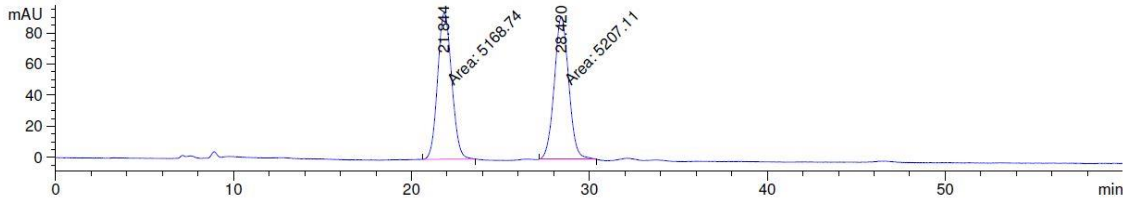

Peak #	RetTime [min]	Type	Width [min]	Area [mAU*s]	Height [mAU]	Area %
1	21.844	MM	0.9163	5168.74268	94.01632	49.8151
2	28.420	MM	0.9453	5207.11377	91.80250	50.1849

Asymmetric sample (**6-Ns**, 96:4 *e.r.*, 92% *ee*)

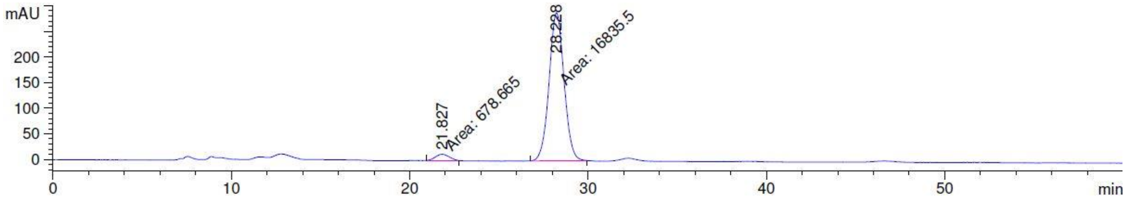

Peak #	RetTime [min]	Type	Width [min]	Area [mAU*s]	Height [mAU]	Area %
1	21.827	MM	0.9090	678.66504	12.44317	3.8749
2	28.228	MM	0.9714	1.68355e4	288.83960	96.1251

4.2. Enantioenriched Starting Materials

Oxidative cleavage of the double bond of the allyltriflamide

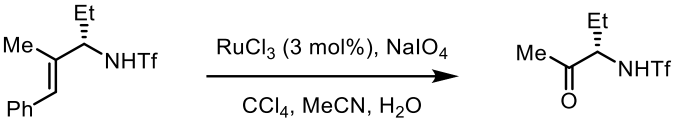

1a **7**

Following a procedure reported in the literature,[10] to a solution of **1a** (92 mg, 0.3 mmol, 1 eq) in a mixed solvent $CCl_4/CH_3CN/H_2O$ (1:1:1.6, v/v/v, 1.8 mL), $NaIO_4$ (257 mg, 1.2 mmol, 4 eq) was added and stirred for 5 min before the addition of $RuCl_3{\cdot}H_2O$ (2.1 mg, 0.01 mmol, 3 mol%). The resulting brown mixture was stirred for 5 min (monitored by TLC) and quenched with 1N HCl. The mixture was extracted with CH_2Cl_2 and the extract was concentrated. The residue was purified by column chromatography (silica gel, hexane:AcOEt 95:5 to 80:20) to afford the ketone **7** as a white solid (50 mg, 71%).

(*S*)-1,1,1-trifluoro-*N*-(2-oxopentan-3-yl)methanesulfonamide (7)

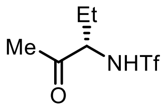

7

7 was obtained as a white solid (50 mg, 71%). **^{1}H NMR** (300 MHz, $CDCl_3$) δ, ppm: 6.08 (d, *J* = 7.1 Hz, 1H), 4.31 (q, *J* = 6.7 Hz, 1H), 2.26 (s, 3H), 2.09 – 1.91 (m, 1H), 1.86 – 1.61 (m, 1H), 0.95 (t, *J* = 7.4 Hz, 3H). **^{19}F NMR** (282 MHz, $CDCl_3$) δ, ppm: -77.58 (s). **^{13}C NMR** (75 MHz, $CDCl_3$) δ, ppm: 204.2 (C), 119.7 (q, *J* = 322.1 Hz, C), 64.3 (CH), 26.7 (CH_3), 25.5 (CH_2), 8.6 (CH_3). **HRMS** (APCI, $[M-H]^+$) *m/z* calculated for $C_6H_9F_3NO_3S$: 232.0261; found 232.0261.

The enantiomeric excess of ketone **7** was determined by chiral HPLC analysis on Chiralpak IB at rt (Hexane : *i*PrOH = 98:2, 0.5 mL/min, λ=254 nm).

Racemic sample (*rac*-**7**)

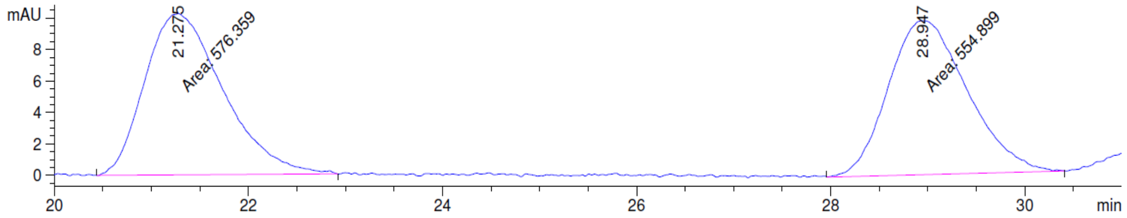

Peak #	RetTime [min]	Type	Width [min]	Area [mAU*s]	Height [mAU]	Area %
1	21.275	MM	0.9349	576.35944	10.27450	50.9485
2	28.947	MM	0.9432	554.89911	9.80476	49.0515

Asymmetric sample (**7**, 100:0 *e.r.*, 99% *ee*)

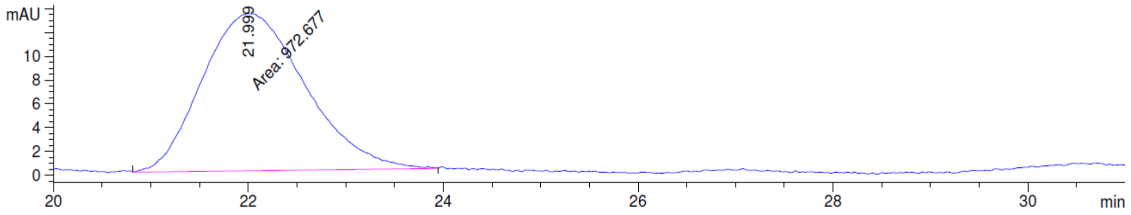

Peak #	RetTime [min]	Type	Width [min]	Area [mAU*s]	Height [mAU]	Area %
1	21.999	MM	1.2242	972.67670	13.24242	100.0000

_N_-propargylation followed by Ir-catalyzed cycloisomerization

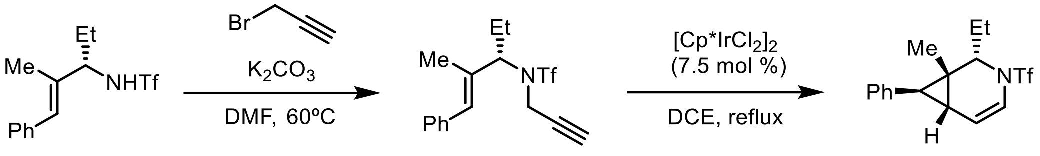

Allyltriflamide **1a** (154 mg, 0.5 mmol, 1eq) was added to a suspension of K_2CO_3 (113 mg, 0.8 mmol, 1.6 eq) in DMF (0.6 mL, 0.9M) and the solution was stirred for 1 h at room temperature. Propargyl bromide (52 µL, 0.55 mmol, 1.1 eq, 80% in toluene) was added and the mixture was stirred overnight at 60°C. After dilution with ether (3 mL), the solution was washed with water (3 x 10 mL) and the aqueous phase was further extracted with ether (3 x 10 mL). The combined organic layers were dried over anhydrous Na_2SO_4 and concentrated in vacuo. Then, column chromatography (silica gel, hexane:Et_2O 95:5) afforded the desired *N*-tethered enyne **8** as a brown oil (126 mg, 76%).

Then, following a procedure reported in the literature,[11] $[IrCp^*Cl_2]_2$ (13.8 mg, 0.017 mmol, 7.5 mol%) was dissolved in dry DCE (1.1 mL) at room temperature under an argon atmosphere. A solution of the obtained *N*-tethered enyne **8** (80 mg 0.23 mmol) in dry DCE (1.2 mL) was added. The mixture was stirred at reflux for 16 h. After cooling to room temperature, the solution was filtered through a short pad of silica gel. The solvent was removed under vacuum and the residue was purified by flash chromatography (silica gel, hexane:Et_2O 98:2) affording the product **9** in 50% yield as a yellowish oil.

(*S*,*E*)-1,1,1-trifluoro-*N*-(2-methyl-1-phenylpent-1-en-3-yl)-*N*-(prop-2-yn-1-yl)methanesulfonamide (8)

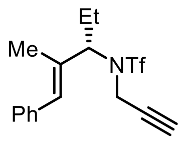

8

8 was obtained as a brown oil (126 mg, 76%). **^{1}H NMR** (300 MHz, $CDCl_3$) δ, ppm: 7.43 – 7.33 (m, 2H), 7.30 (d, *J* = 7.2 Hz, 3H), 6.64 (s, 1H), 4.43 – 4.32 (m, 1H), 4.26 – 3.98 (m, 2H), 2.37 – 2.16 (m, 2H), 2.09 – 1.89 (m, 4H), 1.07 (t, *J* = 7.3 Hz, 3H). **^{19}F NMR** (282 MHz, $CDCl_3$) δ, ppm: -75.41 (s). **^{13}C NMR** (75 MHz, $CDCl_3$) δ, ppm: 136.9 (C), 132.5 (C), 130.5 (CH), 129.1 (CH), 128.4 (CH), 127.3 (CH), 119.87 (q, *J* = 322.7 Hz, C), 78.4 (C), 73.2 (CH), 68.5 (CH), 34.1 (CH_2), 24.2 (CH_2), 17.4 (CH_3), 11.6 (CH_3). **HRMS** (APCI, $[M+H]^+$) *m/z* calculated for $C_{16}H_{19}F_3NO_2S$: 346.1083; found 346.1079.

(1*R*,2*S*,6*S*,7*S*)-2-ethyl-1-methyl-7-phenyl-3-((trifluoromethyl)sulfonyl)-3-azabicyclo[4.1.0]hept-4-ene (9)

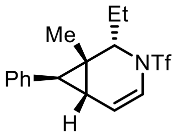

9

9 was obtained as a yellowish oil (40 mg, 50%) and as a single diastereoisomer. **^{1}H NMR** (300 MHz, $CDCl_3$) δ, ppm: 7.37 – 7.18 (m, 3H), 7.13 (d, *J* = 7.3 Hz, 2H), 6.13 (d, *J* = 7.5 Hz, 1H), 5.98 – 5.87 (m, 1H), 4.20 (dd, *J* = 9.1, 3.4 Hz, 1H), 2.57 (d, *J* = 4.3 Hz, 1H), 1.97 – 1.81 (m, 1H), 1.74 – 1.60 (m, 2H), 1.01 (t, *J* = 7.4 Hz, 3H), 0.94 (s, 3H). **^{19}F NMR** (282 MHz, $CDCl_3$) δ, ppm: -74.23 (s). **^{13}C NMR** (75 MHz, $CDCl_3$) δ, ppm: 137.1 (C), 128.8 (CH), 128.4 (CH), 126.7 (CH), 119.6 (CH), 117.4 (CH), 59.0 (CH), 37.1 (C), 35.8 (CH), 24.4 (CH_2), 20.1 (CH), 16.7 (CH_3), 10.7 (CH_3). **HRMS** (APCI, $[M+H]^+$) *m/z* calculated for $C_{16}H_{19}F_3NO_2S$: 346.1083; found 346.1081.

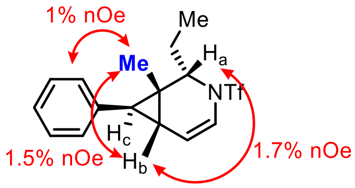

The stereochemistry of **9** was assigned based on several nOe experiments. The observation of nOes between the *blue* methyl with the aromatic protons and with H_b (1.85 ppm, 1H) allows to confirm the *syn* configuration of these 3 groups. Additionally, nOe between H_b and H_a allows to stablish all relative stereochemistry.

The enantiomeric excess of compound **9** was determined by chiral HPLC analysis on Chiralpak IA3 at rt (Hexane, 0.5 mL/min, λ=220 nm).

Racemic sample (*rac*-**9**)

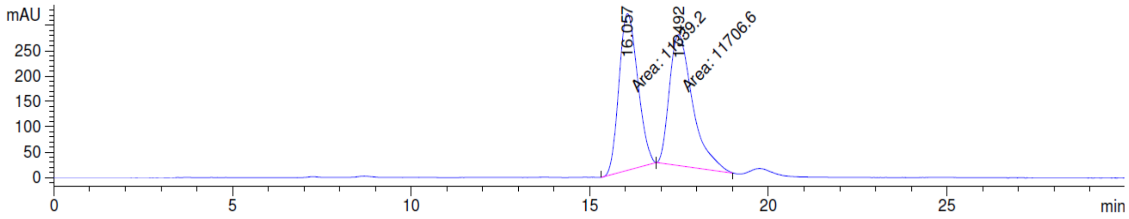

Peak #	RetTime [min]	Type	Width [min]	Area [mAU*s]	Height [mAU]	Area %
1	16.057	MM	0.6115	1.13392e4	309.07544	49.2028
2	17.492	MM	0.7548	1.17066e4	258.48047	50.7972

Asymmetric sample (**9**, 100:0 *e.r.*, 99% *ee*)

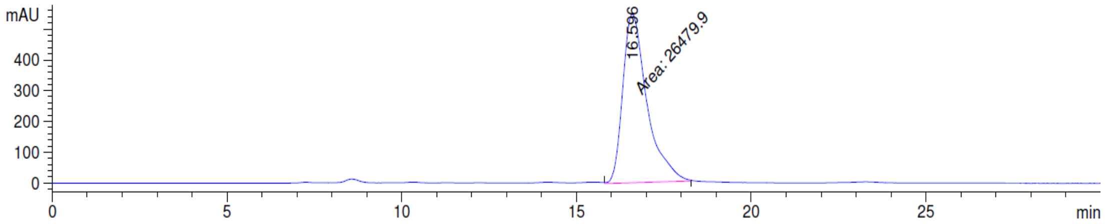

Peak #	RetTime [min]	Type	Width [min]	Area [mAU*s]	Height [mAU]	Area %
1	16.596	MM	0.8050	2.64799e4	548.26031	100.0000

Preparation of the cycloadduct with (*S*)-configuration

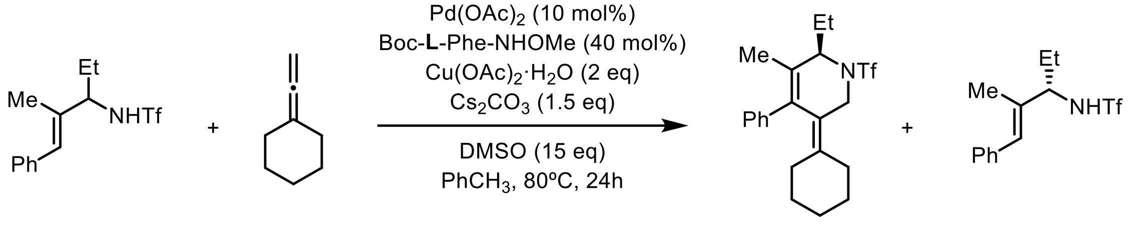

rac-**1a** | **2a** | (*R*)-**3aa** | **1a** 30% yield 99% *ee*

Following the standard procedure for the kinetic resolution at 80°C, **1a** was recovered in 30% yield and 99% *ee*.

Enantioselectivity of the remaining starting material **1a** was determined by chiral HPLC analysis on Chiralpak IB at rt (Hexane : *i*PrOH = 98:2, 0.5 mL/min, λ=254 nm).

Racemic sample (*rac*-**1a**)

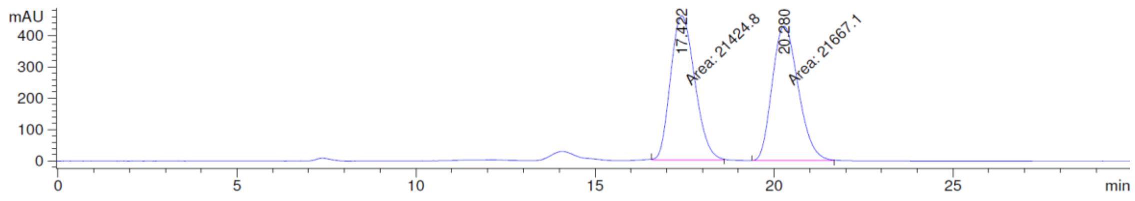

Peak #	RetTime [min]	Type	Width [min]	Area [mAU*s]	Height [mAU]	Area %
1	17.422	MM	0.7789	2.14248e4	458.45050	49.7188
2	20.280	MM	0.8431	2.16671e4	428.30322	50.2812

Asymmetric sample (**1a**, 100:0 *e.r.*, 99% ee_{SM})

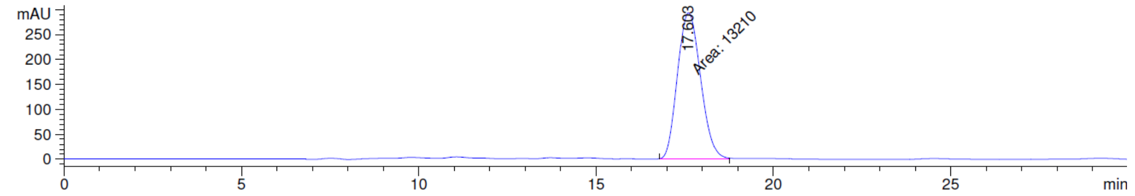

Peak #	RetTime [min]	Type	Width [min]	Area [mAU*s]	Height [mAU]	Area %
1	17.603	MM	0.7520	1.32100e4	292.78214	100.0000

Then, the recovered **1a** (99% *ee*) was subjected to the standard conditions at 70°C, using Boc-D-Phe-NHOMe as chiral ligand, to afford (*S*)-**3aa** in 88% yield and 99% *ee*.

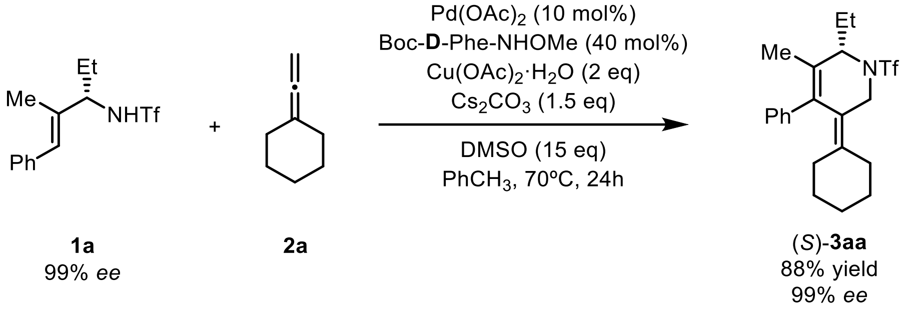

Enantioselectivity of the cycloadduct product (*S*)-**3aa** was determined by chiral SFC analysis on Phenomenex Cellulose-1 at 40°C (CO_2 : MeOH = 99:1, 1 mL/min, λ=254 nm).

Racemic sample (*rac*-**3aa**)

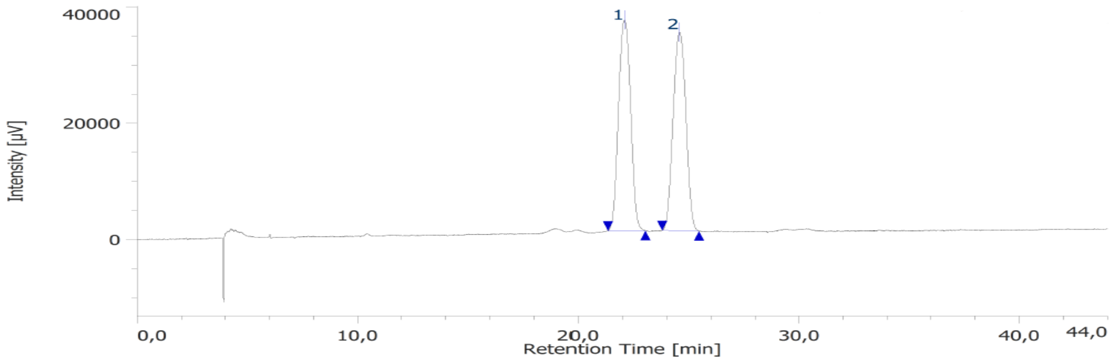

#	Peak Name	CH	tR [min]	Area [µV·sec]	Height [µV]	Area%	Height%	Quantity	NTP	Resolution	Symmetry Factor	Warning
1	Unknown	11	22,073	1386301	36215	50,178	51,475	N/A	7102	2,316	1,023	
2	Unknown	11	24,560	1376477	34139	49,822	48,525	N/A	7897	N/A	1,024	

Asymmetric sample ((*S*)-**3aa**, 100:0 *e.r.*, 99% *ee*)

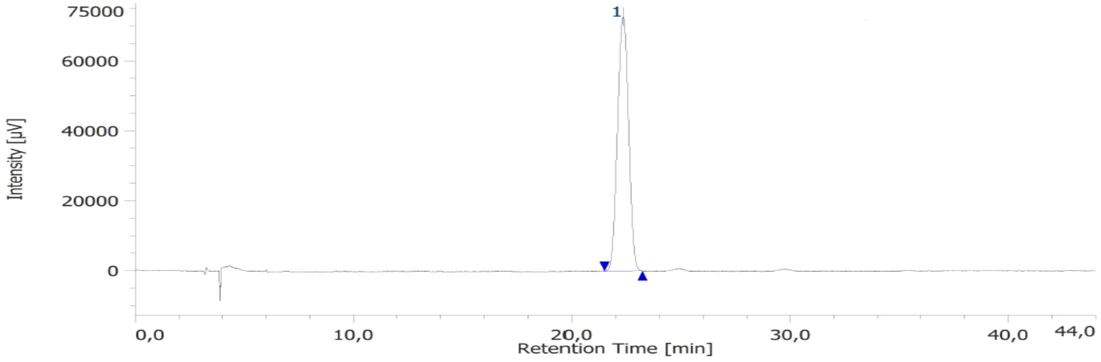

#	Peak Name	CH	tR [min]	Area [µV·sec]	Height [µV]	Area%	Height%	Quantity	NTP	Resolution	Symmetry Factor	Warning
1	Unknown	11	22,330	2581237	72683	100,000	100,000	N/A	8618	N/A	0,990	

5. References

(1) Ji, E.; Meng, H.; Zheng, Y.; Ramadoss, V.; Wang, Y. *Eur. J. Org. Chem.* **2019**, *44*, 7367-7371.

(2) Murphy, S. K.; Petrone, D. A.; Coulter, M. M.; Dong, V. M. *Org. Lett*. **2011**, *13*, 6216-6219.

(3) (a) Zhong, C.; Tung, P.; Chao, T.; Yeh, M. *J. Org. Chem.* **2017**, *82*, 481-501.

(4) a) Kippo, T.; Fukuyama, T.; Ryu, I. *Org. Lett*. **2011**, *13*, 3864–3867. b) Clavier, H.; Le Jeune, K.; de Riggi, I.; Tenaglia, A.; Buono, G. *Org. Lett.* **2010**, *13*, 308–311. c) Shukla, R. K.; Nair, A. M.; Khan, S.; Volla, C. M. R. *Angew. Chemie Int. Ed.* **2020**, *59*, 17042–17048. d) Gevorgyan, A.; Obst, M. F.; Guttormsen, Y.; Maseras, F.; Hopmann, K. H.; Bayer, A. *Chem. Sci.* **2019**, *10*, 10072–10078. e) Boreux, A.; Indukuri, K.; Gagosz, F.; Riant, O. *ACS Catal*. **2017**, *7*, 8200–8204.

(5) Xiao, K.-J.; Lin, D. W.; Miura, M.; Zhu, R.-Y.; Gong, W.; Wasa, M.; Yu, J.-Q. *J. Am. Chem. Soc.* **2014**, *136*, 8138–8142.

(6) Casanova, N.; Del Río, K. P.; García-Fandiño, R.; Mascareñas, J. L.; Gulías, M. *ACS Catal.* **2016**, *6*, 3349-3353.

(7) Fernández, D. F.; Gulías, M.; Mascareñas, J. L.; López, F. *Angew. Chem. Int. Ed.* **2017**, *56*, 9541-9545.

(8) Shao, Q.; Wu, Q.-F.; He, J.; Yu, J.-Q. *J. Am. Chem. Soc.* **2018**, *140*, 5322-5325.

(9) Font, M.; Cendón, B.; Seoane, A.; Mascareñas, J. L.; Gulías, M. *Angew. Chem. Int. Ed.* **2018**, *57*, 8255-8259.

(10) Zhou, C. Y.; Zhu, S. F.; Wang, L. X.; Zhou, Q. L *J. Am. Chem. Soc.* **2010**, *132*, 10955–10957.

(11) Benedetti, E.; Simonneau, A.; Hours, A.; Amouri, H.; Penoni, A.; Palmisano, G.; Malacria, M.; Goddard, J.-P.; Fensterbank, L. *Adv. Synth. Catal.* **2011**, *353*, 1908–1912.

6. NMR Spectra

^{1}H NMR (300 MHz, $CDCl_3$)

SI1

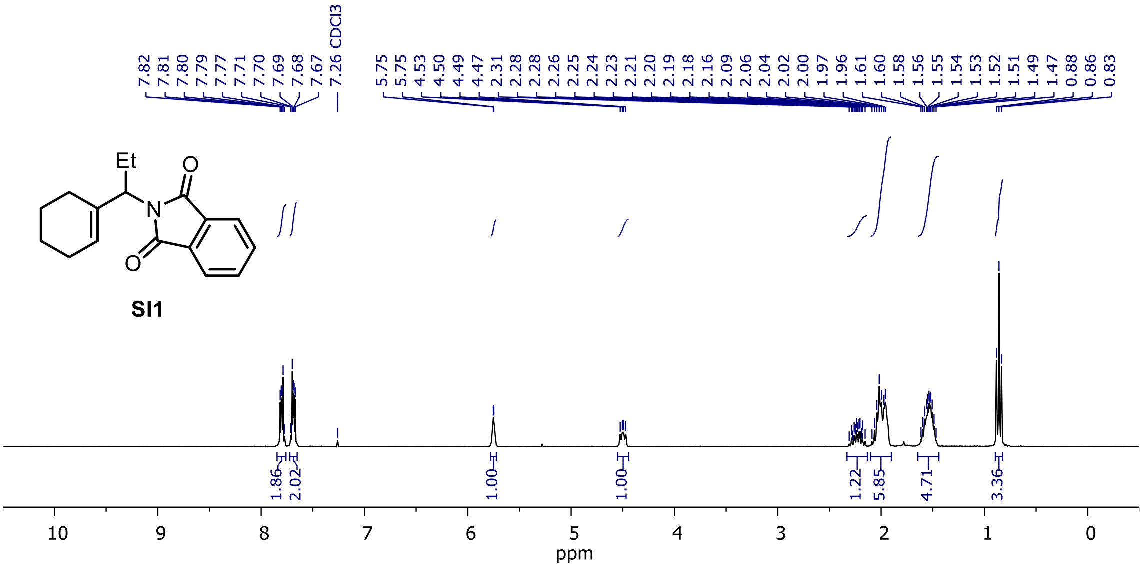

DEPT-135

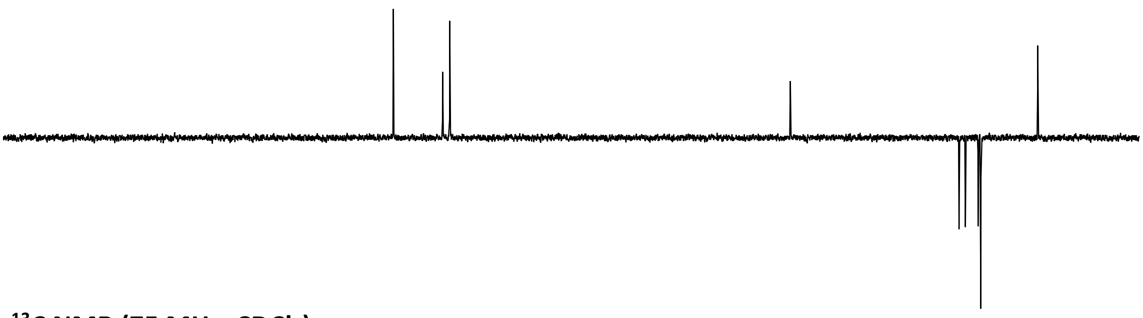

^{13}C NMR (75 MHz, $CDCl_3$)

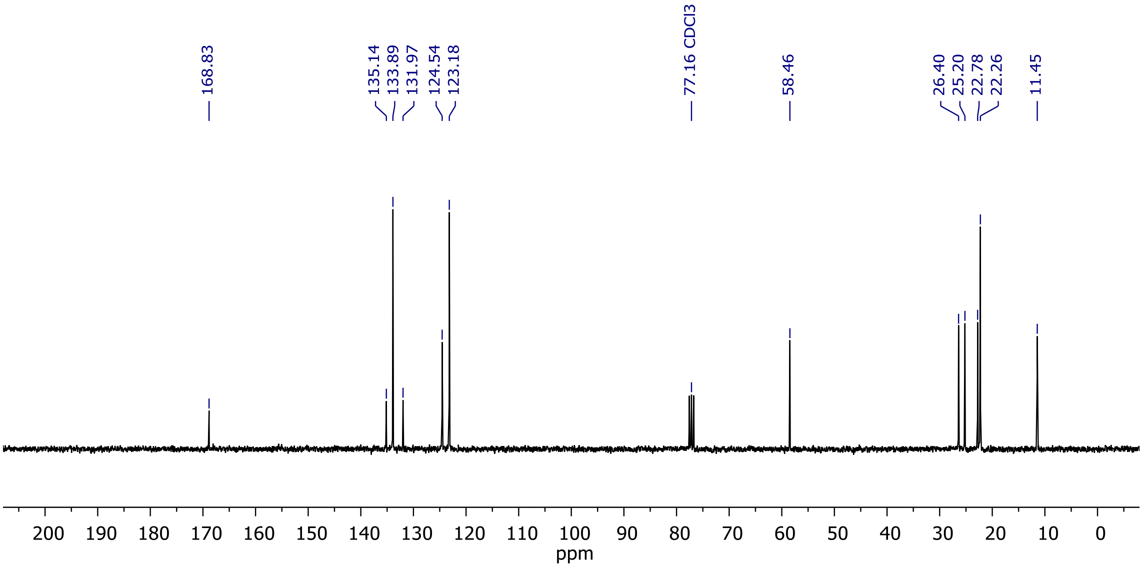

^{1}H NMR (300 MHz, CDCl$_3$)

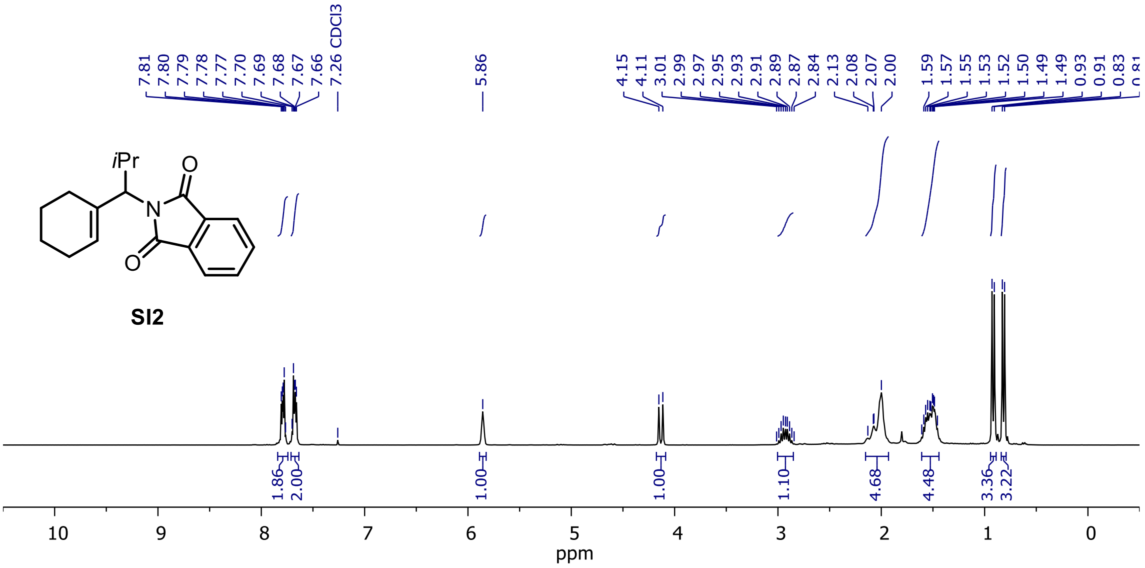

DEPT-135

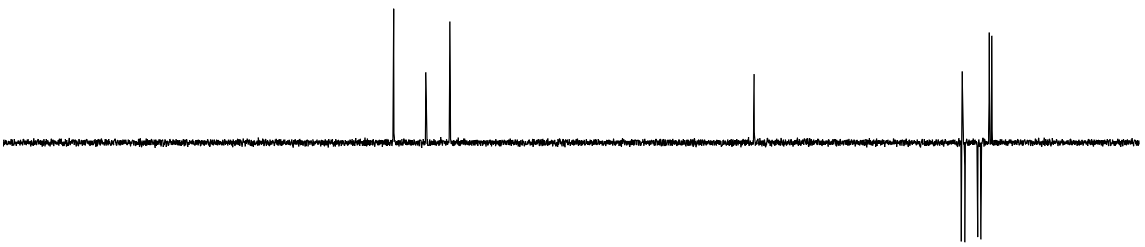

^{13}C NMR (75 MHz, CDCl$_3$)

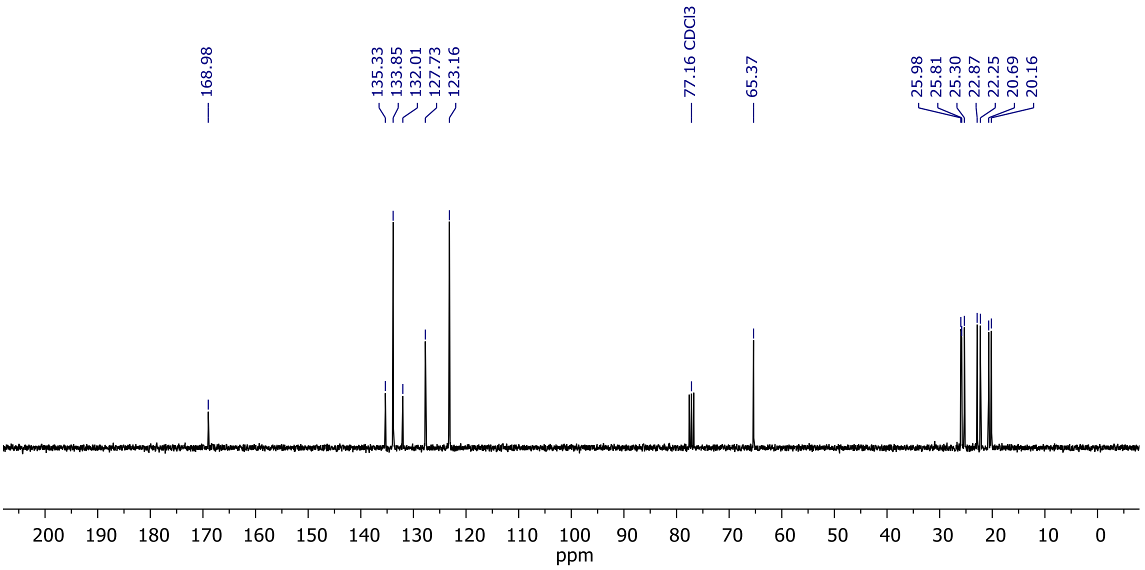

^{1}H NMR (300 MHz, $CDCl_3$)

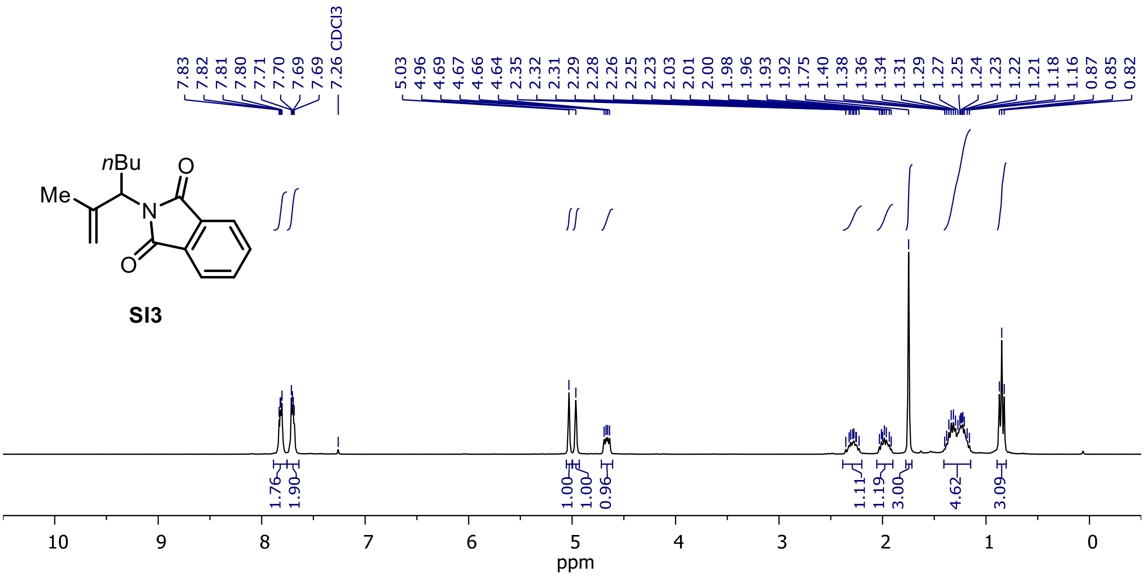

DEPT-135

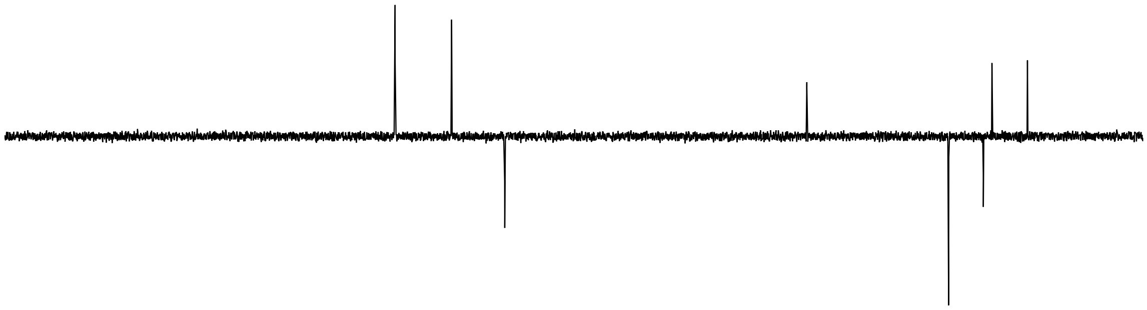

^{13}C NMR (75 MHz, $CDCl_3$)

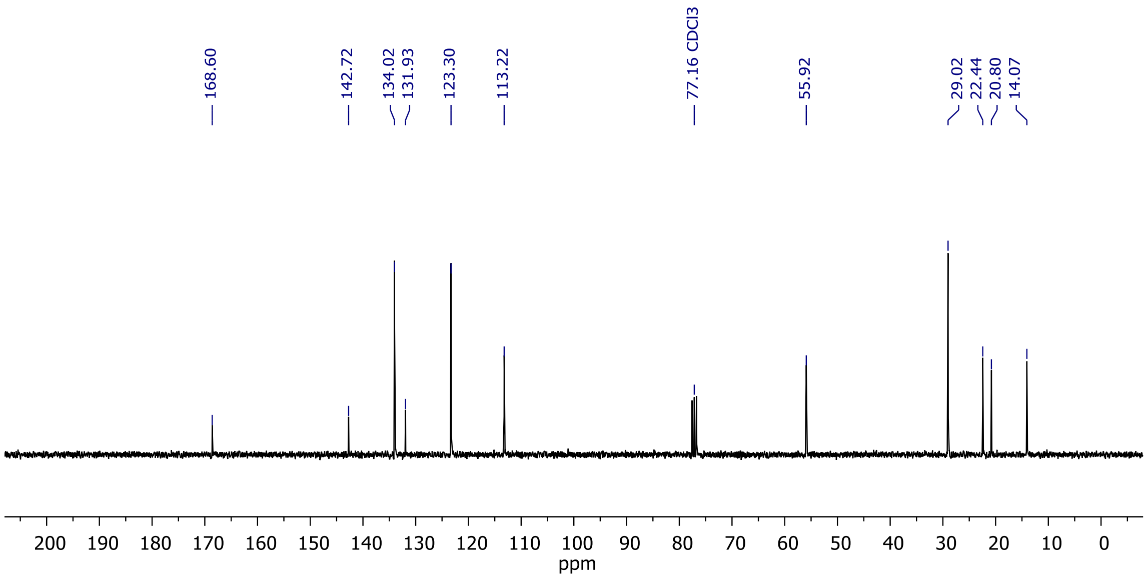

^{1}H NMR (300 MHz, CDCl$_3$)

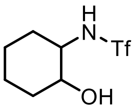

SI4

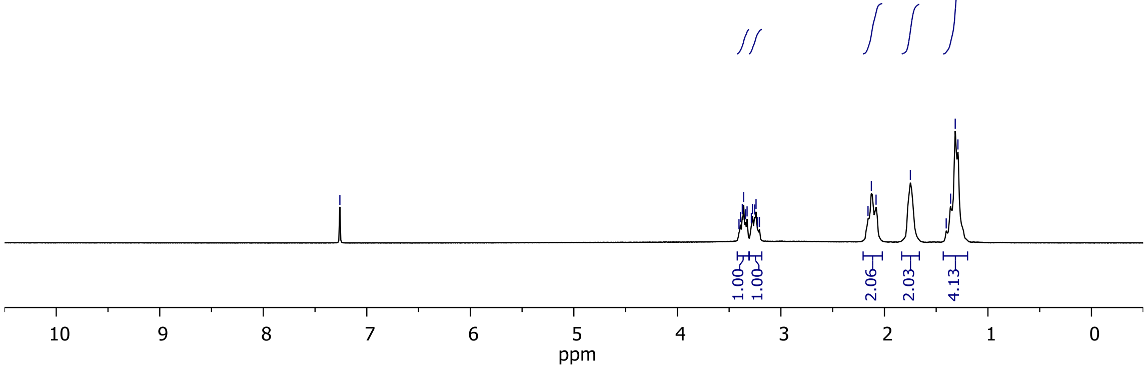

DEPT-135

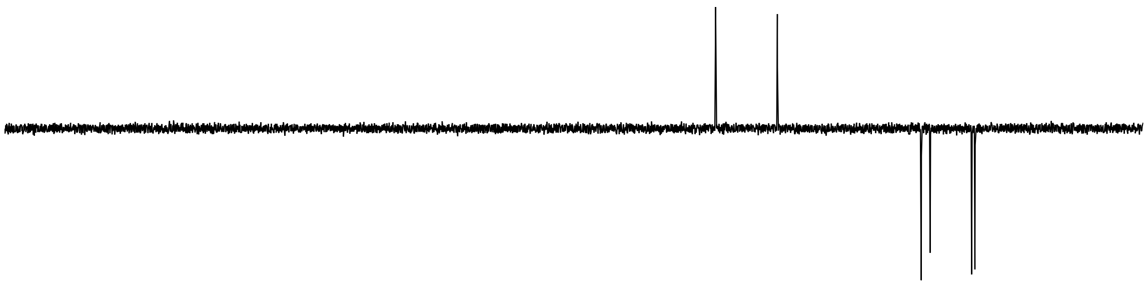

^{13}C NMR (75 MHz, CDCl$_3$)

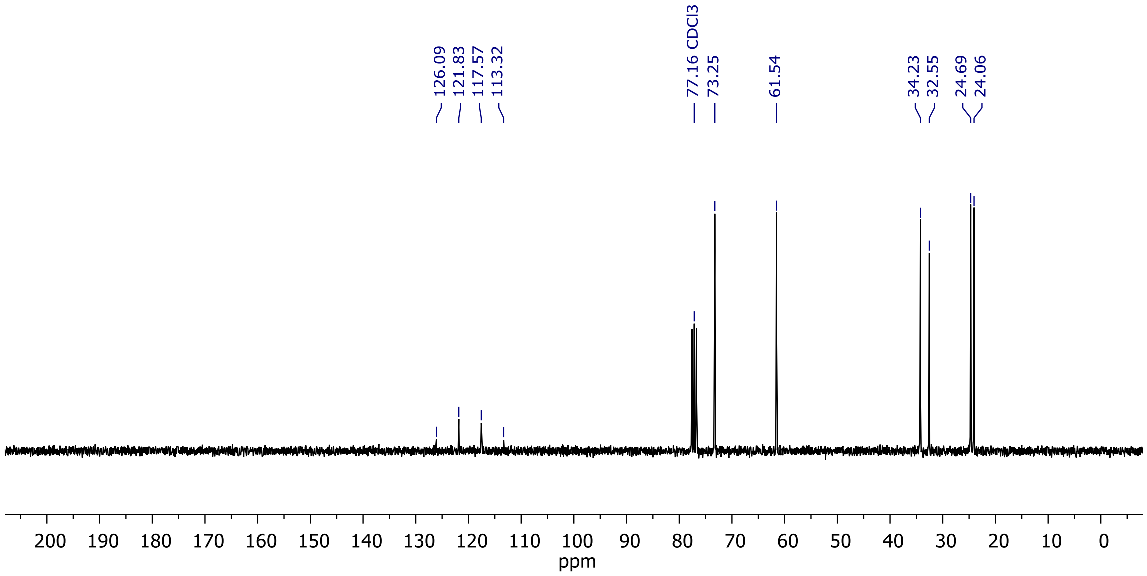

^{1}H NMR (300 MHz, $CDCl_3$)

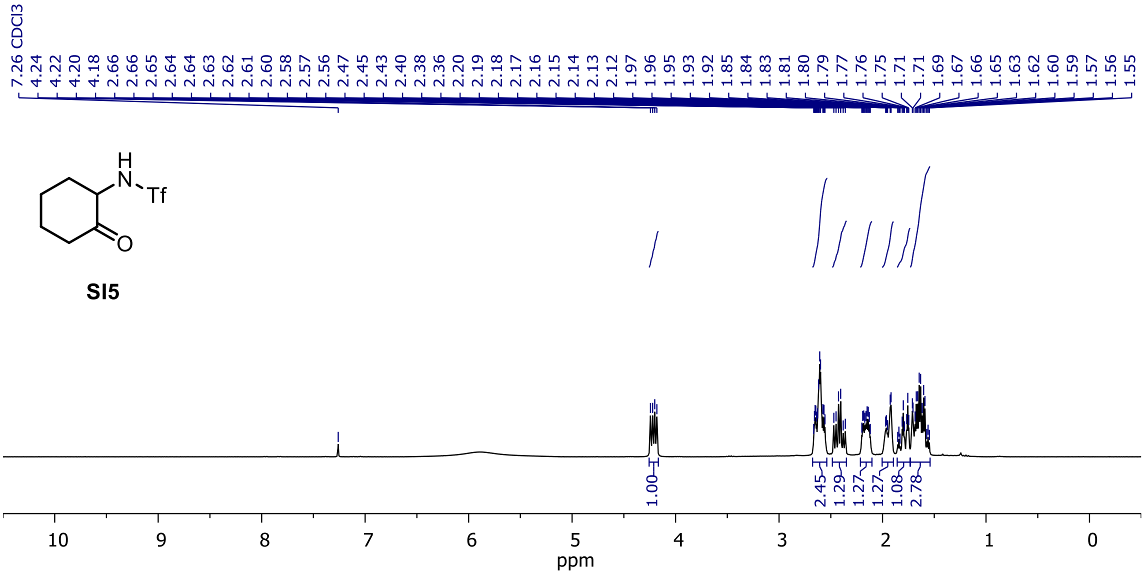

DEPT-135

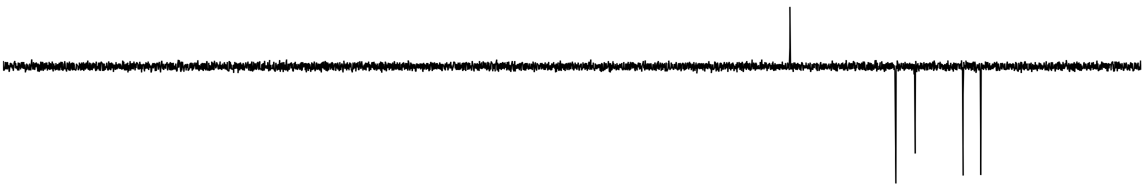

^{13}C NMR (75 MHz, $CDCl_3$)

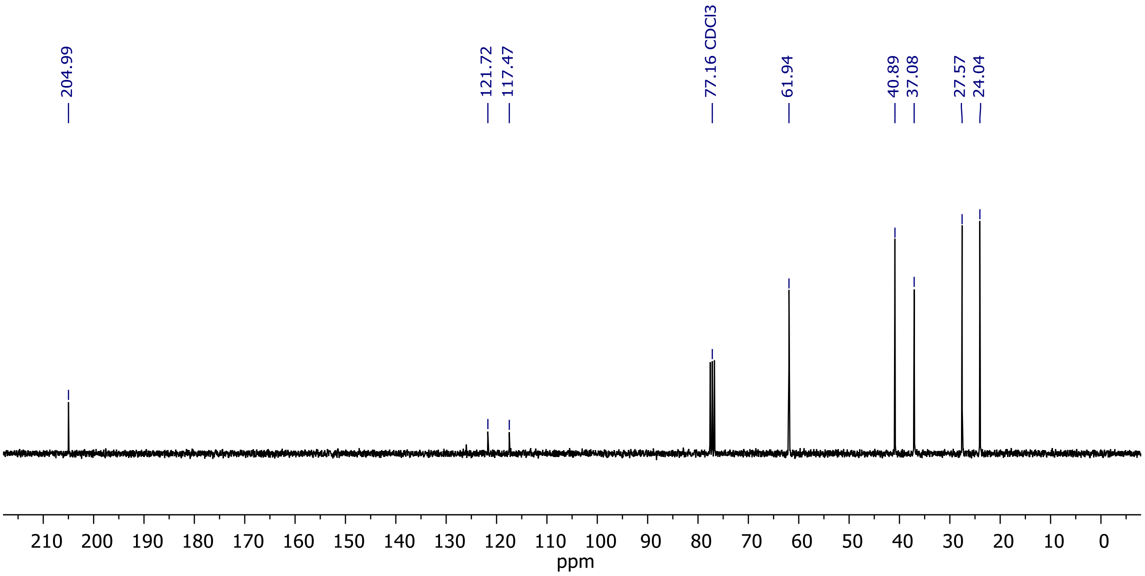

^1H NMR (300 MHz, $CDCl_3$)

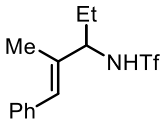

rac-**1a**

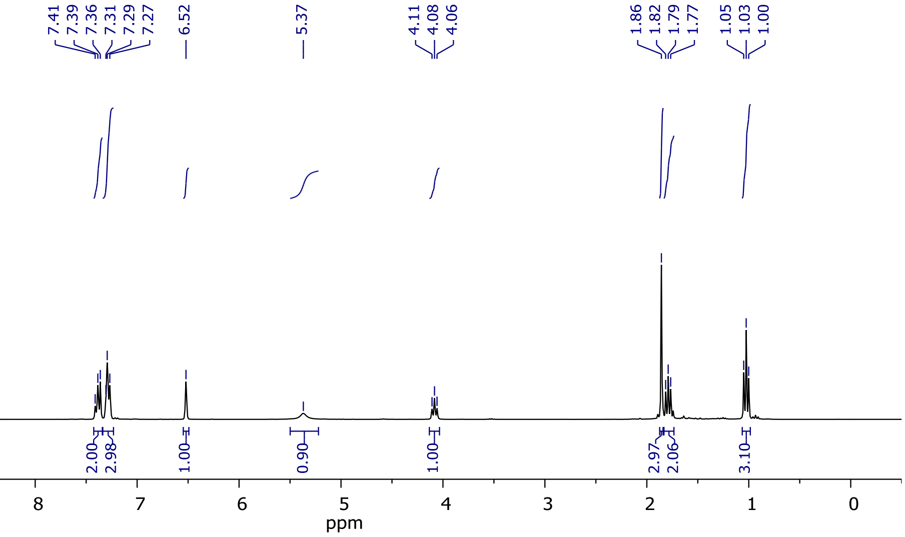

DEPT-135

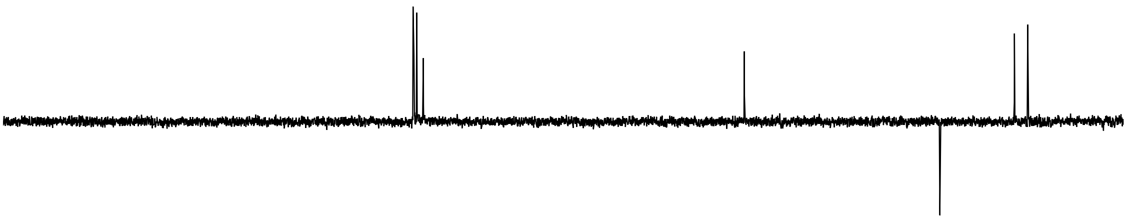

^{13}C NMR (75 MHz, $CDCl_3$)

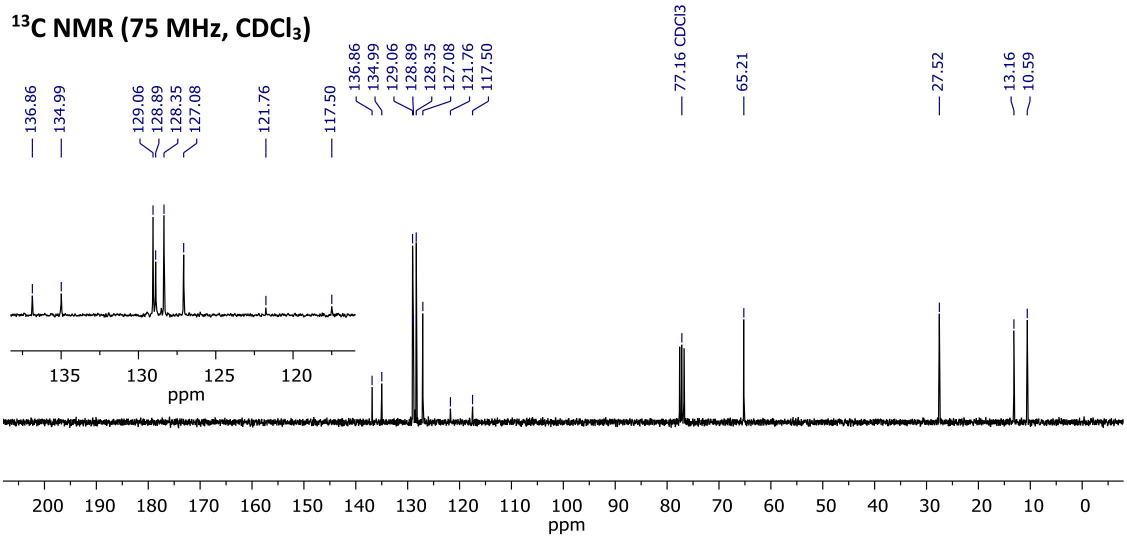

^{1}H NMR (300 MHz, CDCl$_3$)

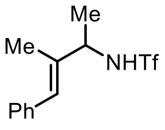

rac-**1b**

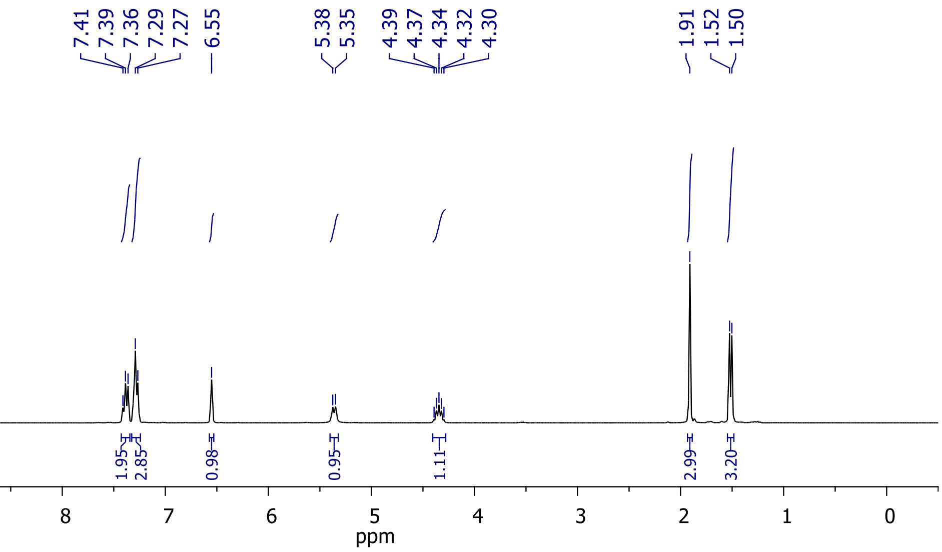

DEPT-135

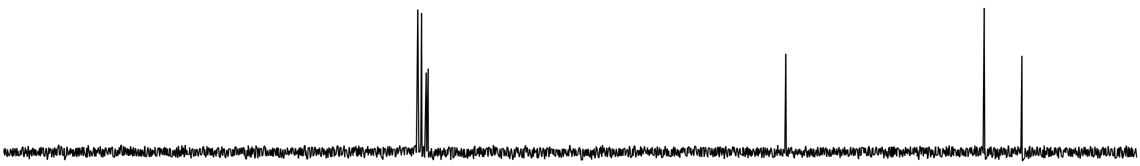

^{13}C NMR (75 MHz, CDCl$_3$)

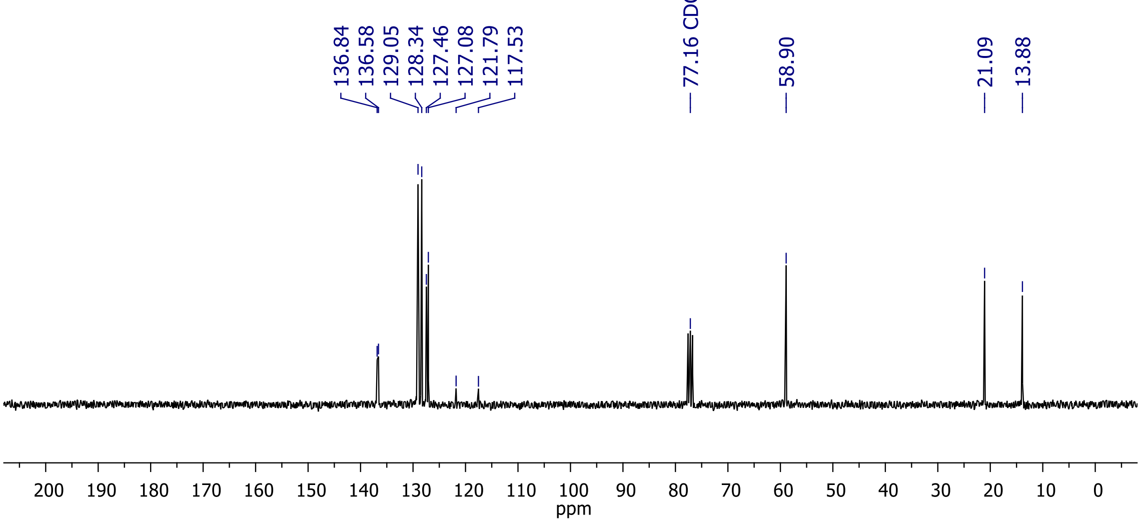

^{1}H NMR (300 MHz, CDCl3)

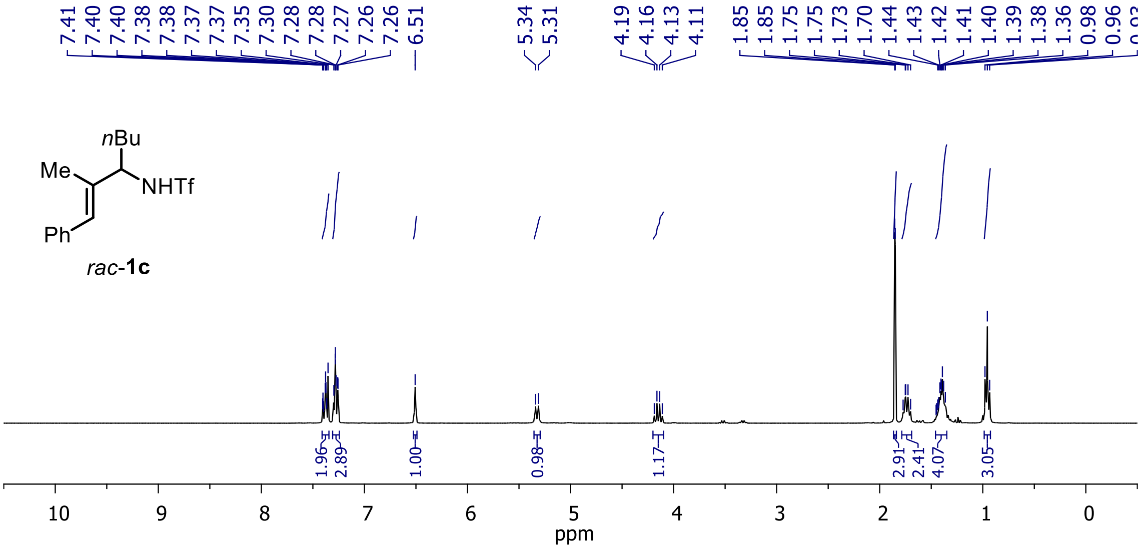

DEPT-135

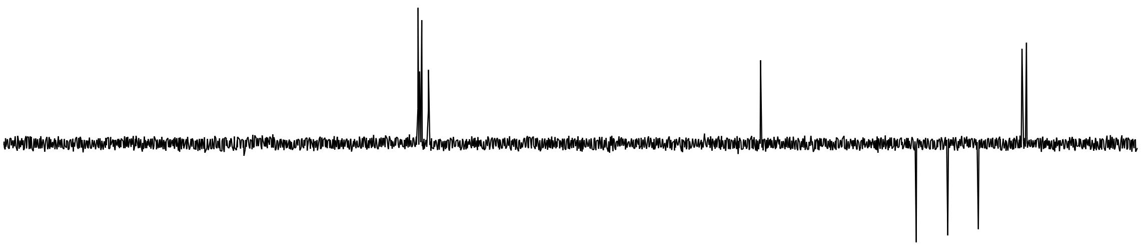

^{13}C NMR (75 MHz, CDCl3)

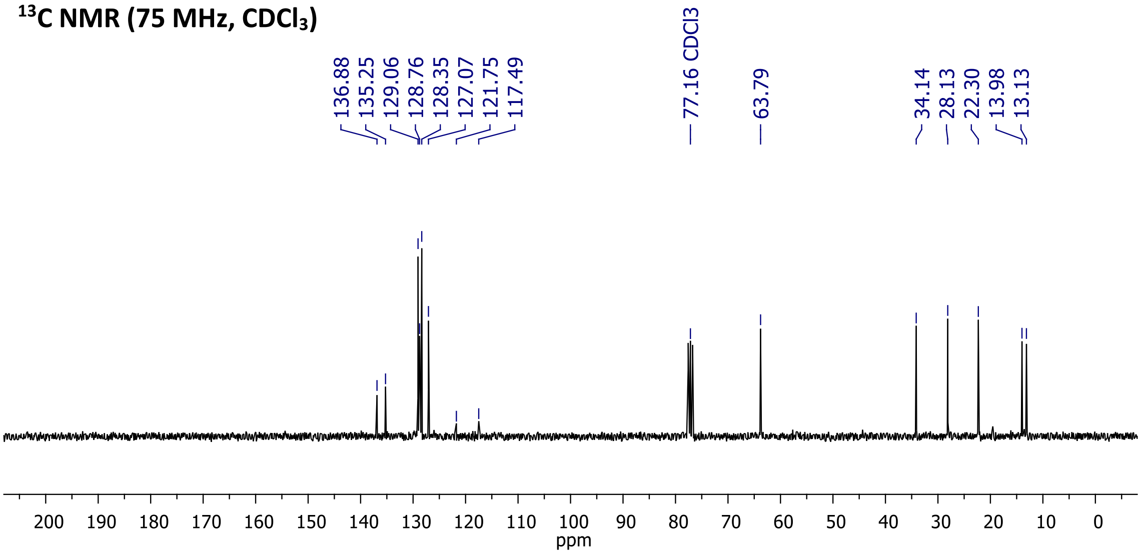

^{1}H NMR (300 MHz, CDCl$_3$)

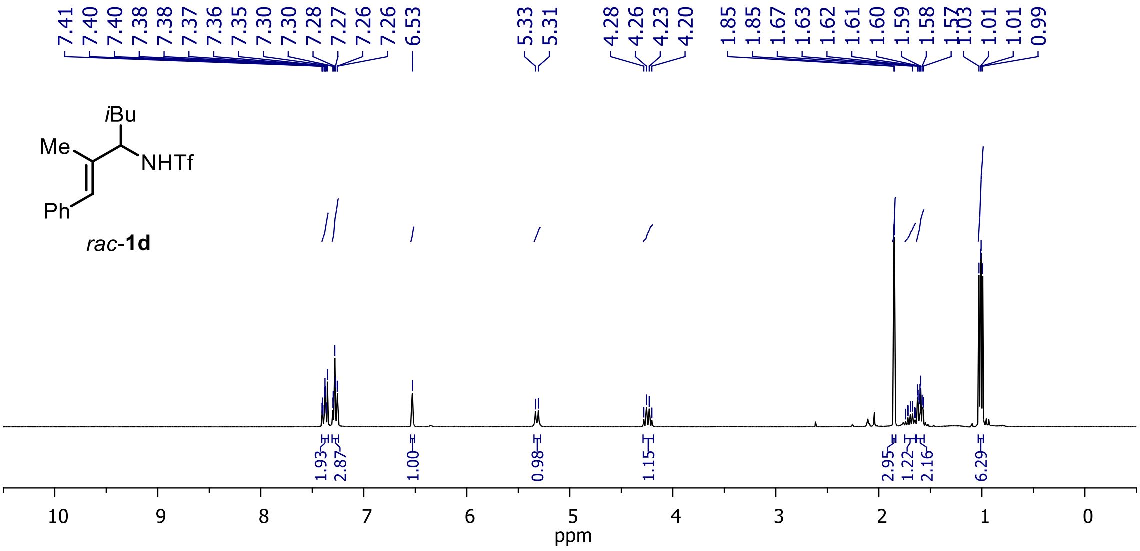

DEPT-135

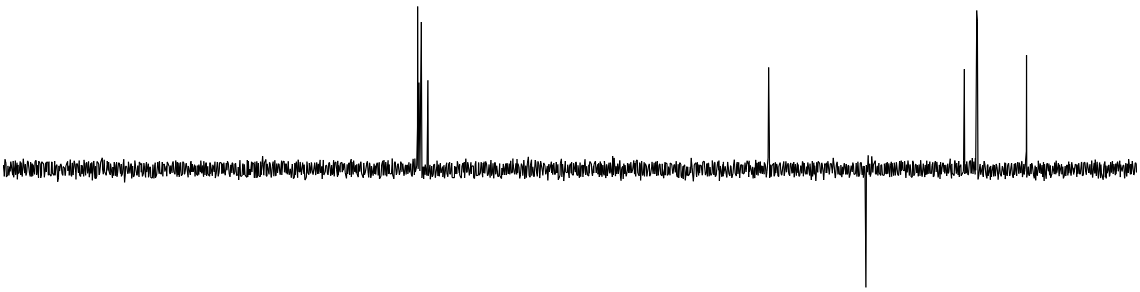

^{13}C NMR (75 MHz, CDCl$_3$)

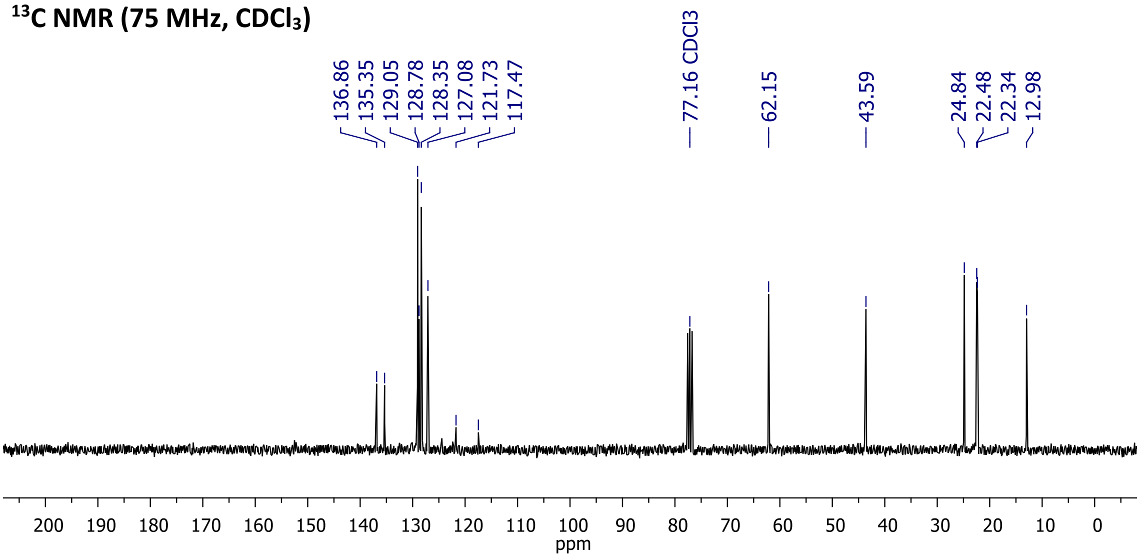

^{1}H NMR (300 MHz, CDCl$_3$)

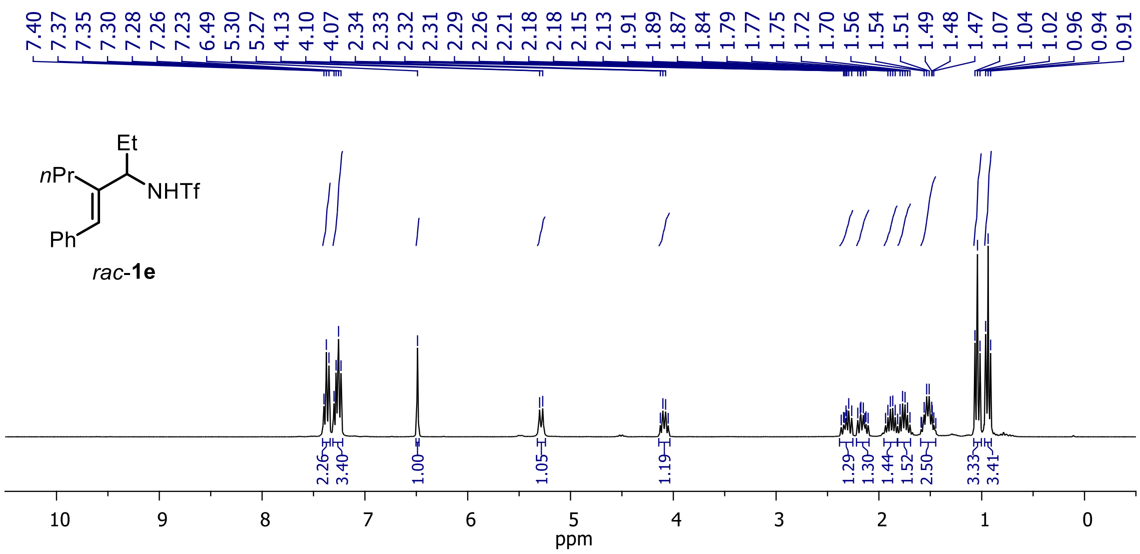

DEPT-135

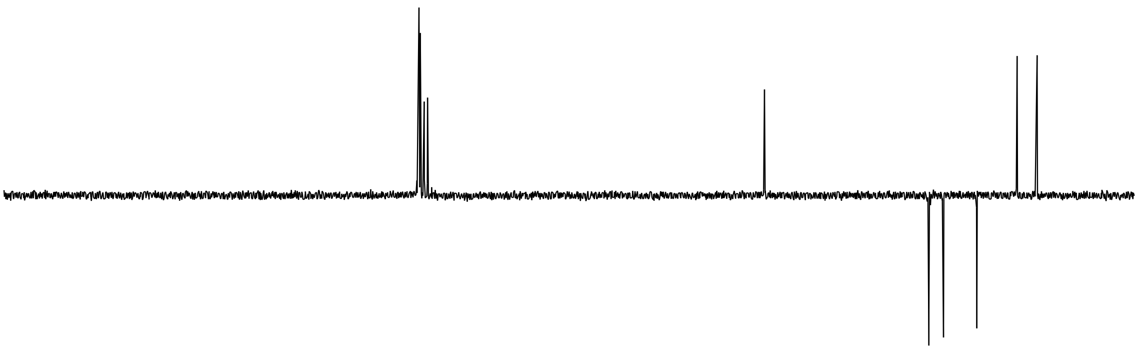

^{13}C NMR (75 MHz, CDCl$_3$)

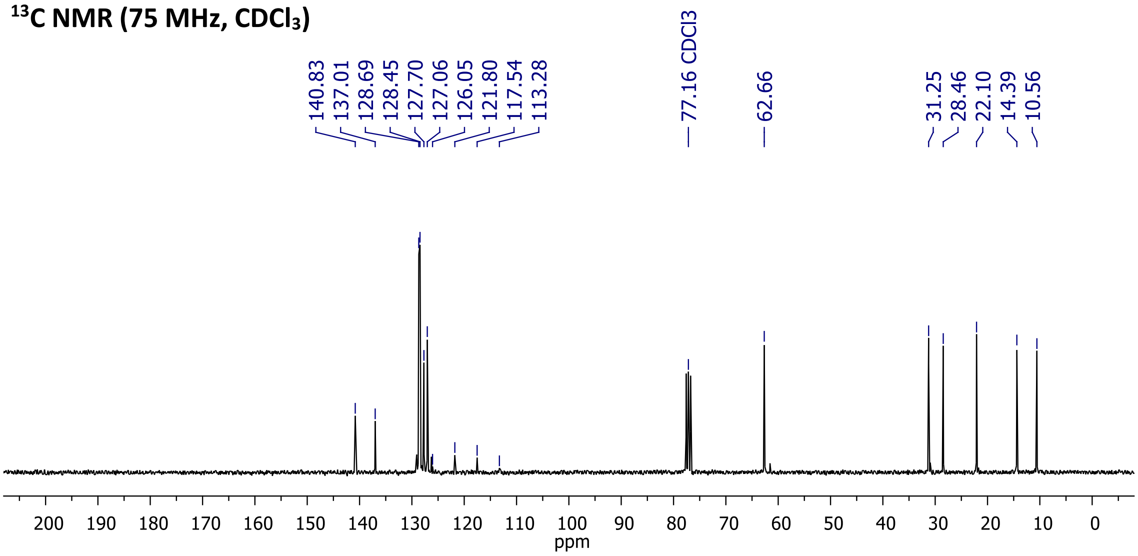

^{1}H NMR (300 MHz, $CDCl_3$)

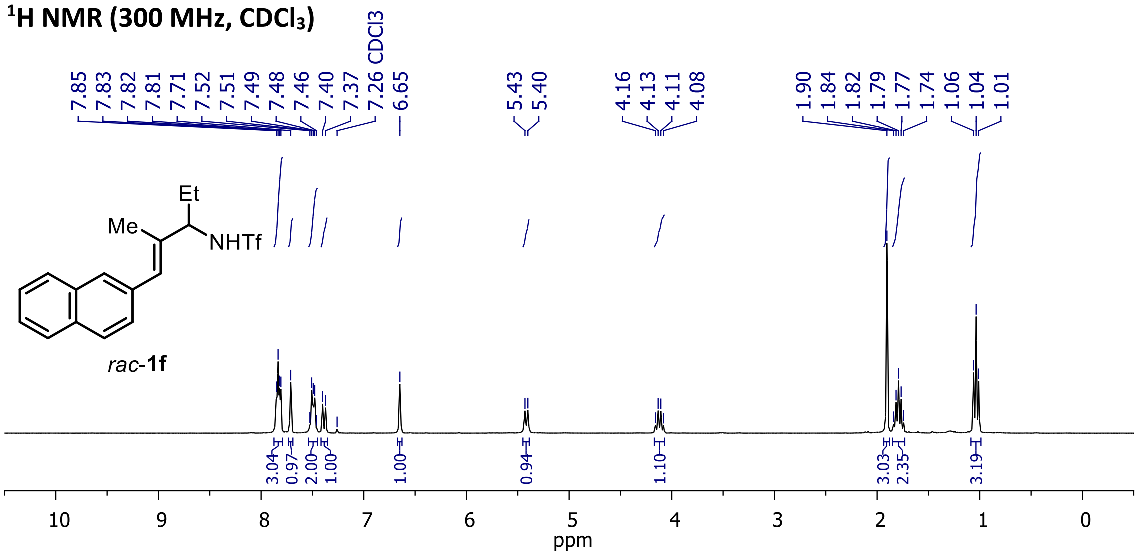

DEPT-135

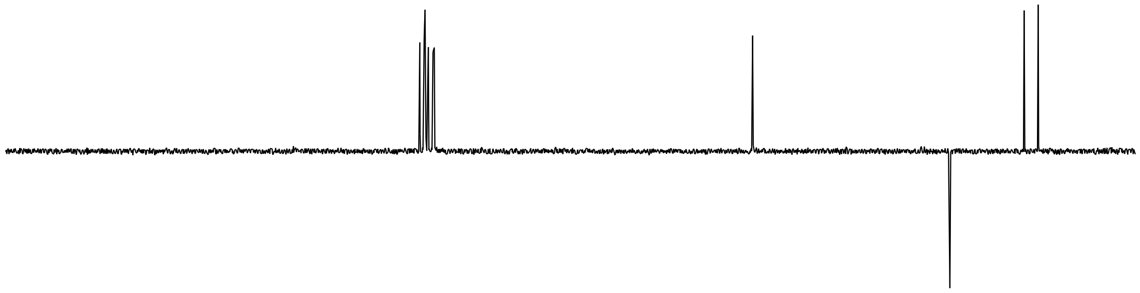

^{13}C NMR (75 MHz, $CDCl_3$)

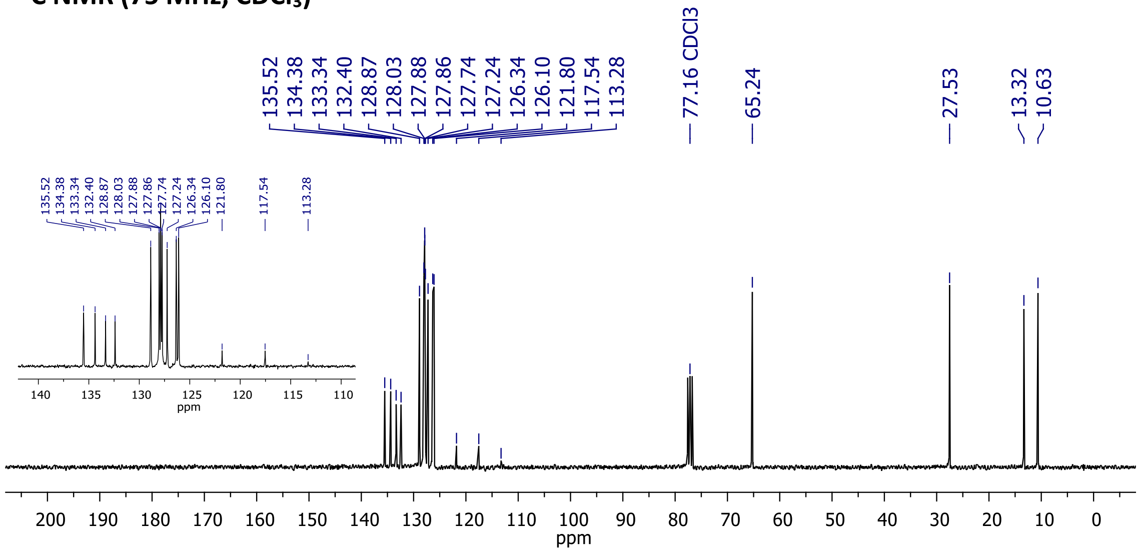

^{1}H NMR (300 MHz, CDCl$_3$)

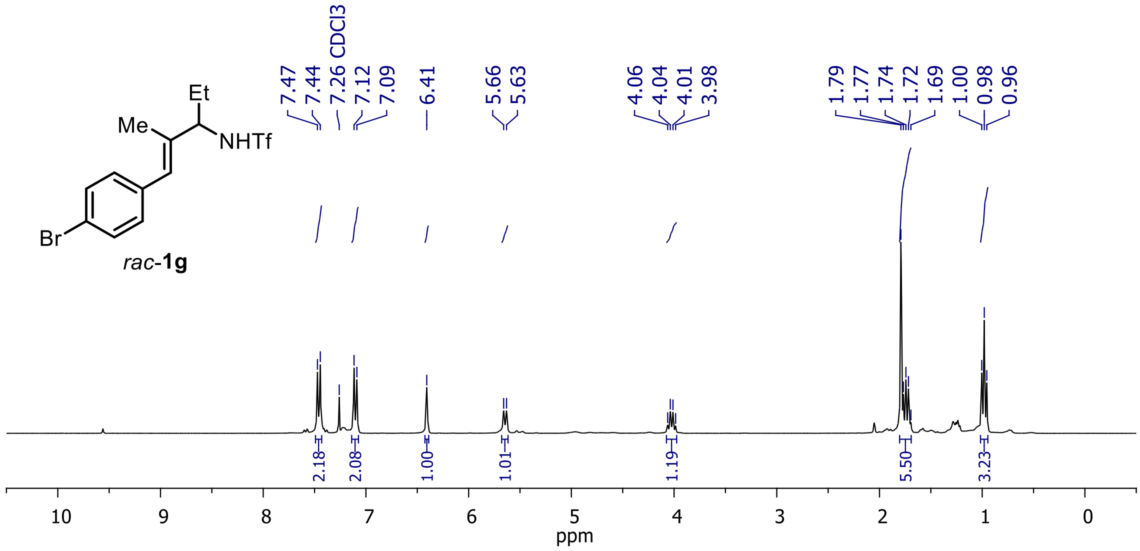

DEPT-135

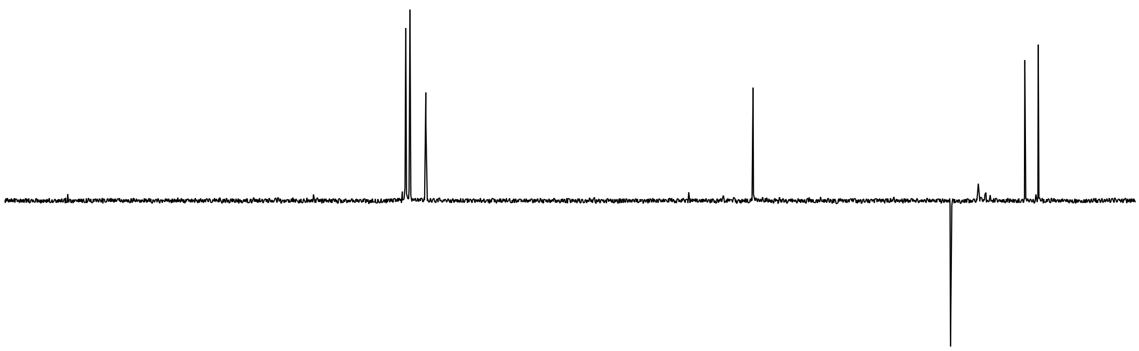

^{13}C NMR (75 MHz, CDCl$_3$)

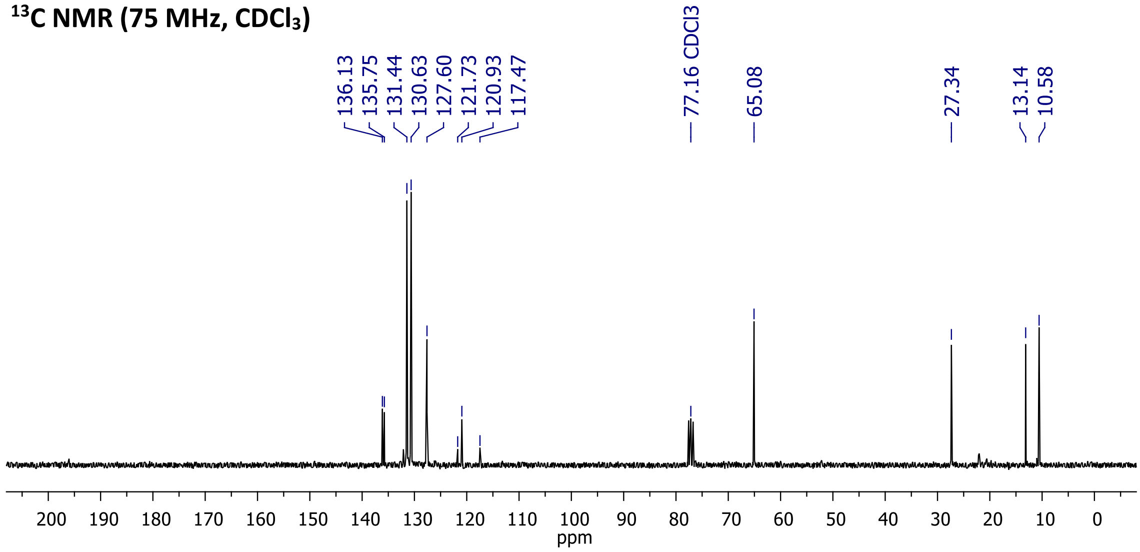

^{1}H NMR (300 MHz, CDCl$_3$)

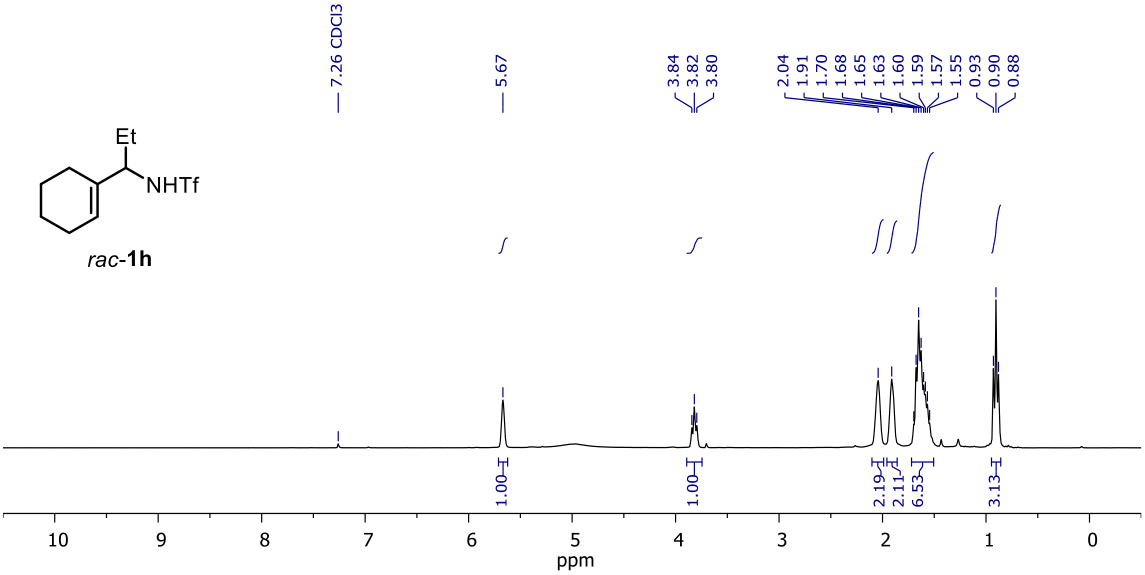

DEPT-135

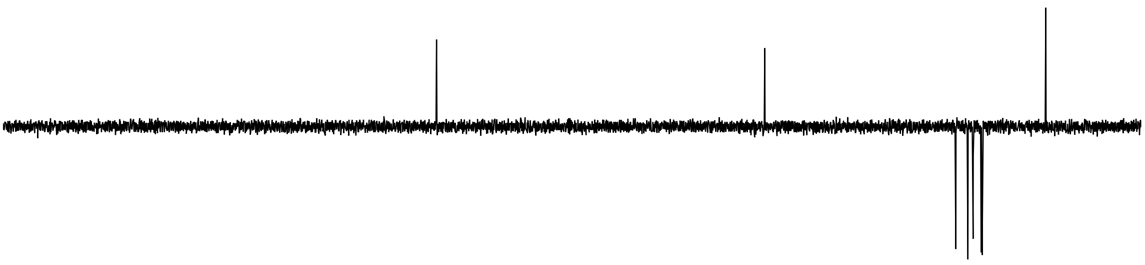

^{13}C NMR (75 MHz, CDCl$_3$)

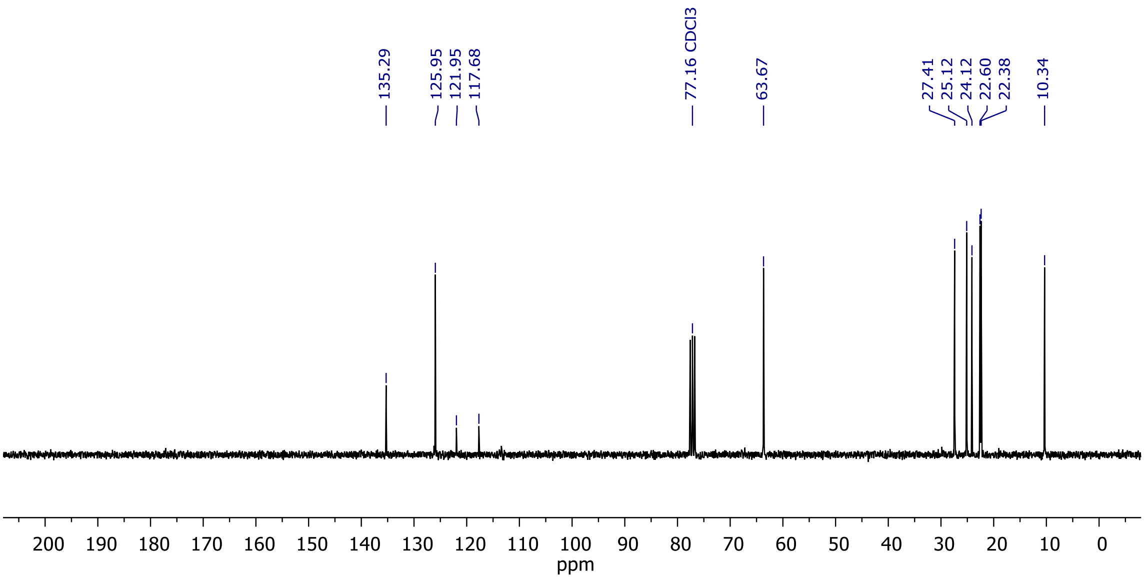

^1H NMR (300 MHz, $CDCl_3$)

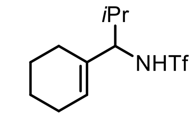

rac-**1i**

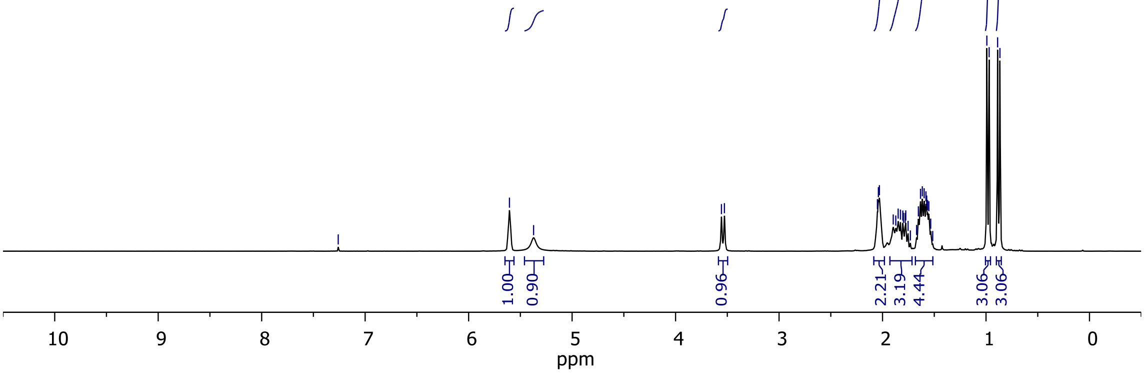

DEPT-135

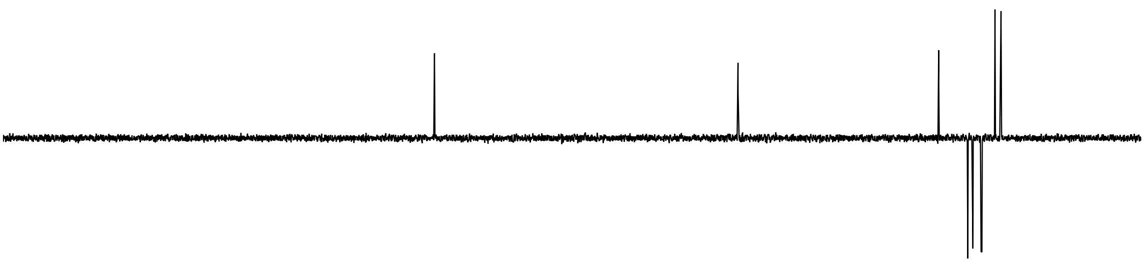

^{13}C NMR (75 MHz, $CDCl_3$)

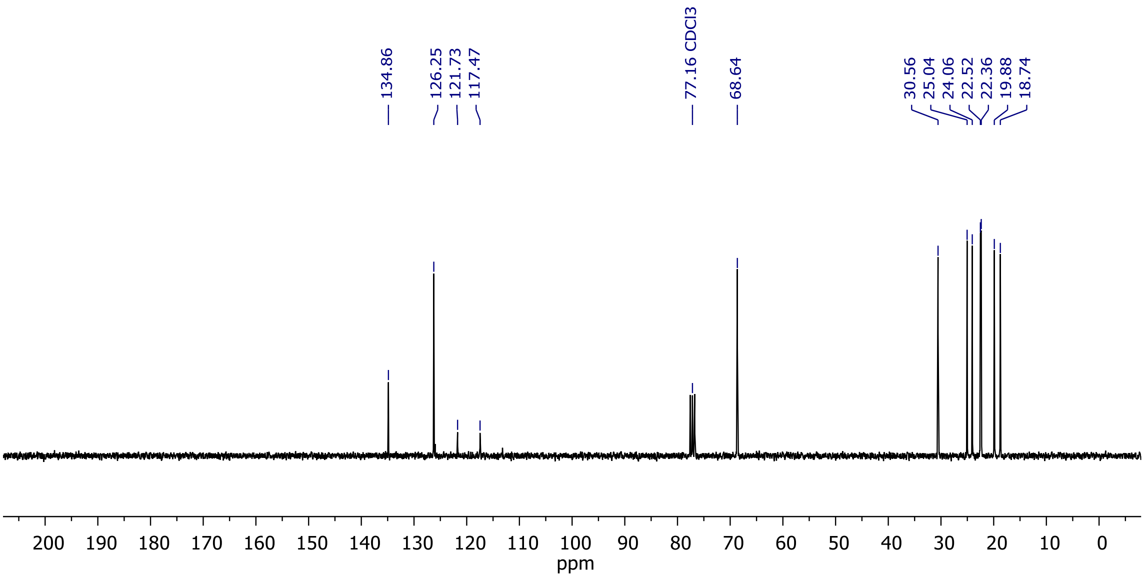

^{1}H NMR (300 MHz, $CDCl_3$)

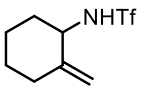

rac-**1j**

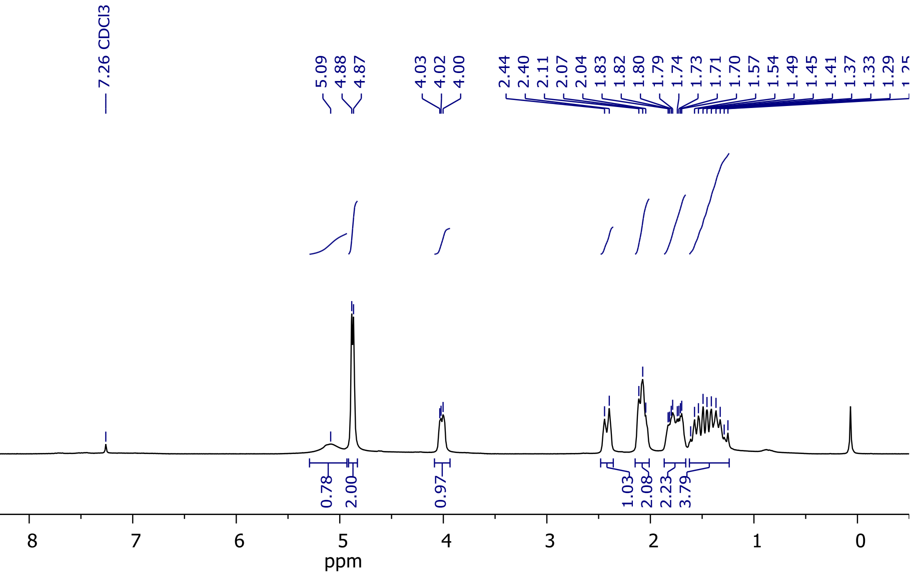

DEPT-135

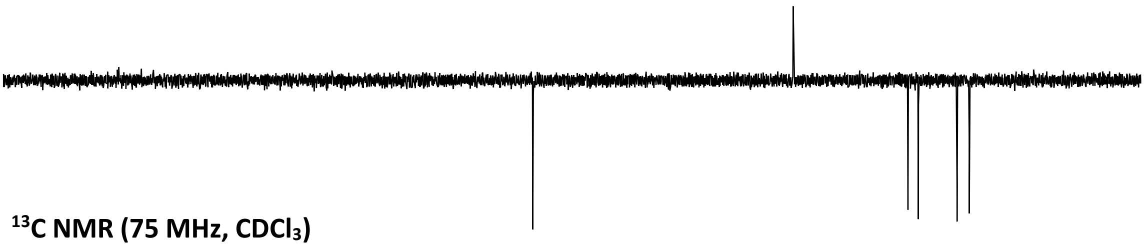

^{13}C NMR (75 MHz, $CDCl_3$)

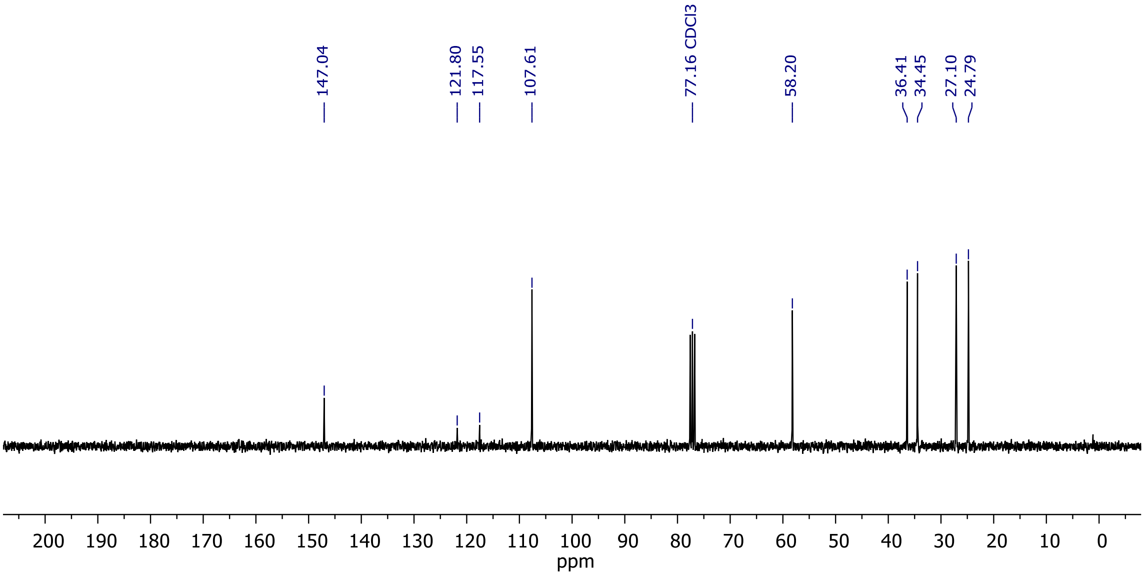

^{1}H NMR (300 MHz, CDCl$_3$)

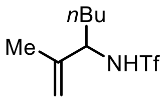

rac-**1k**

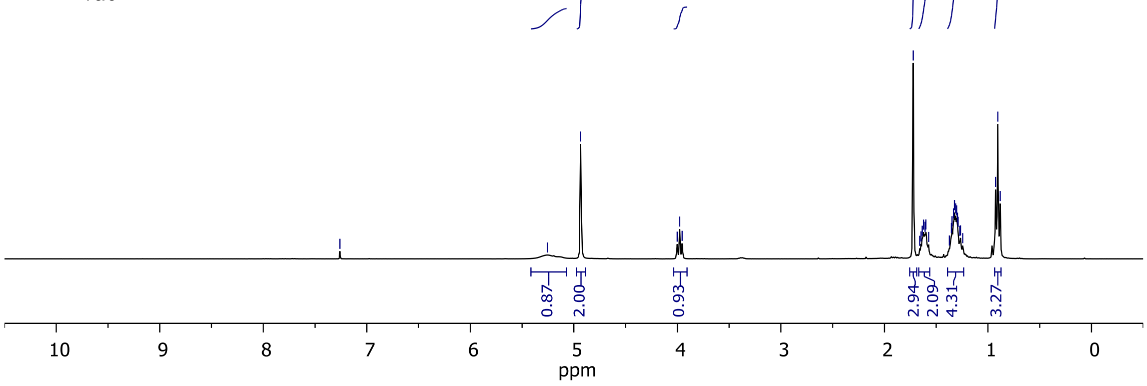

DEPT-135

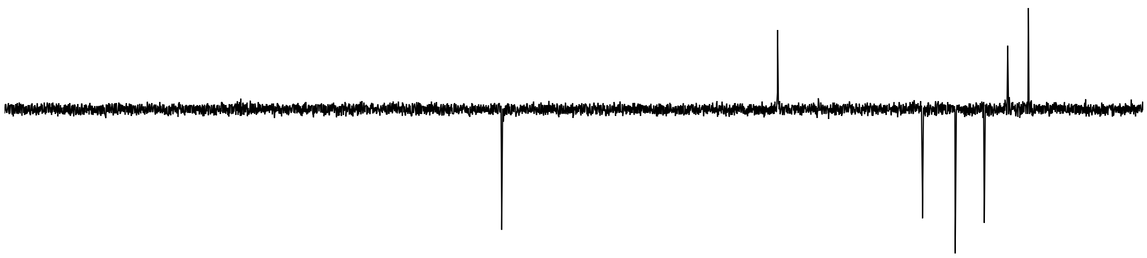

^{13}C NMR (75 MHz, CDCl$_3$)

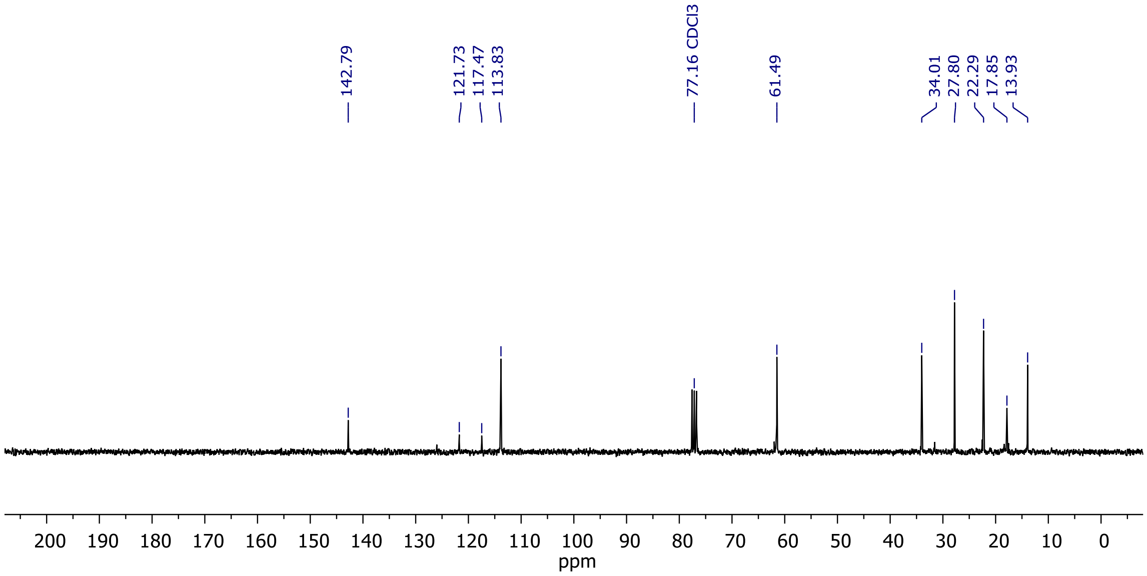

^{1}H NMR (300 MHz, CDCl$_3$)

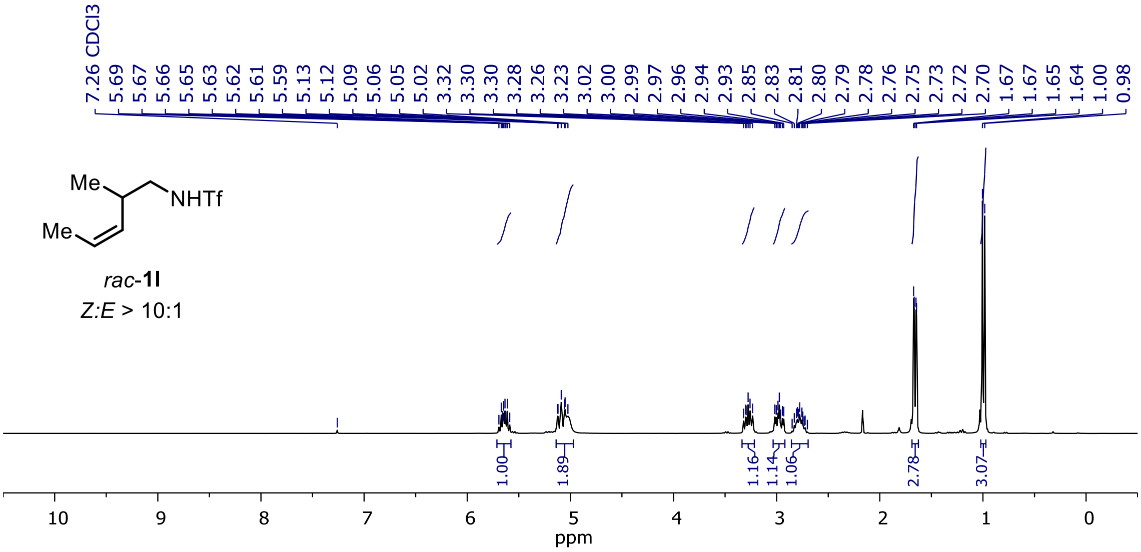

DEPT-135

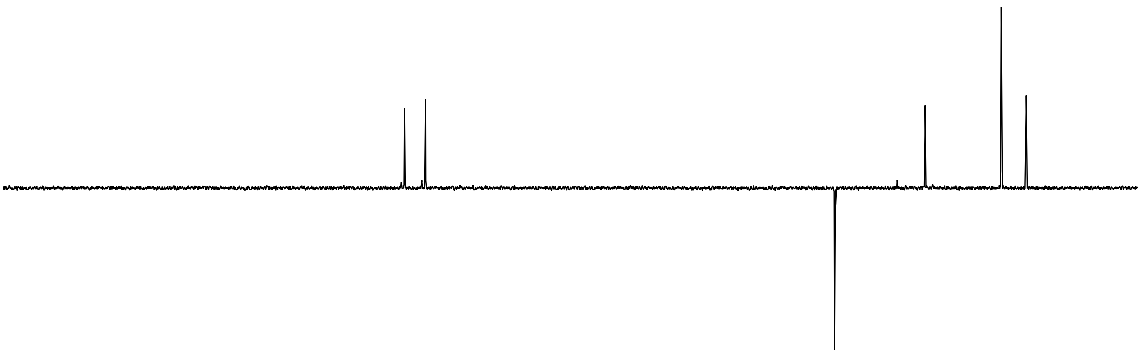

^{13}C NMR (75 MHz, CDCl$_3$)

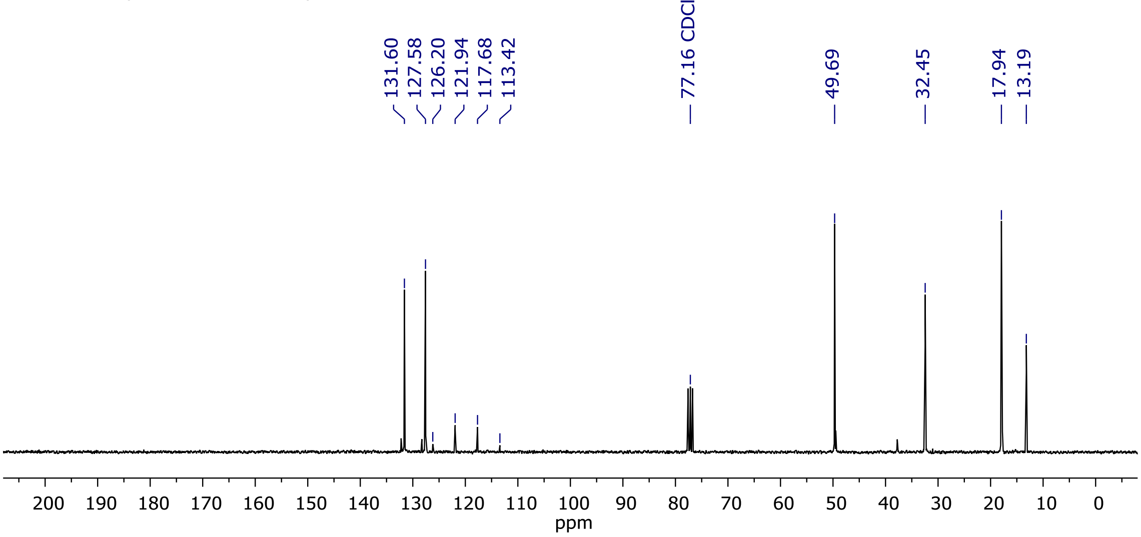

^{1}H NMR (300 MHz, $CDCl_3$)

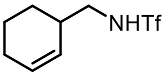

rac-**1m**

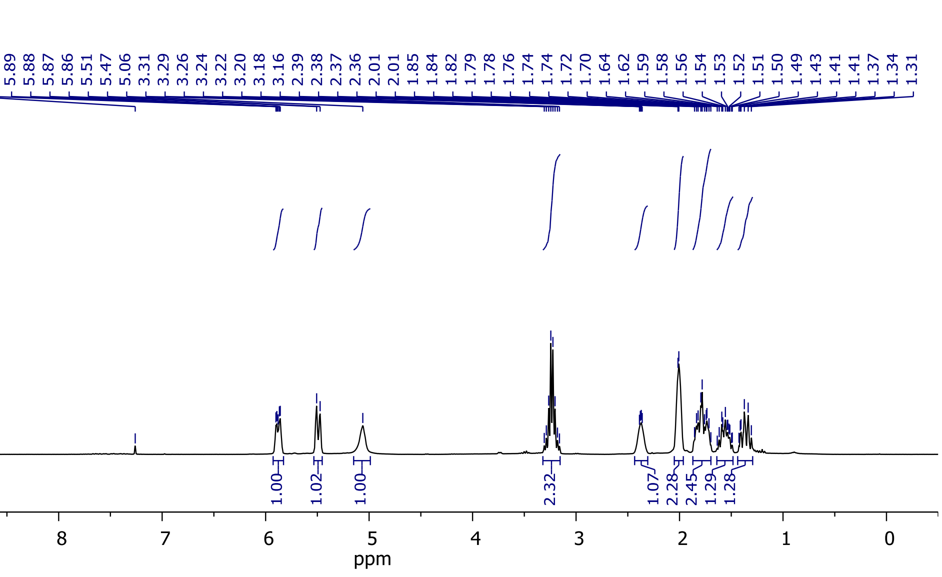

DEPT-135

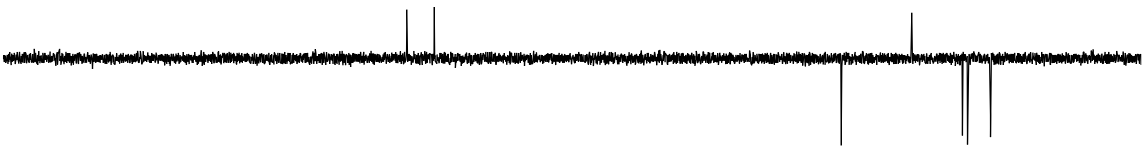

^{13}C NMR (75 MHz, $CDCl_3$)

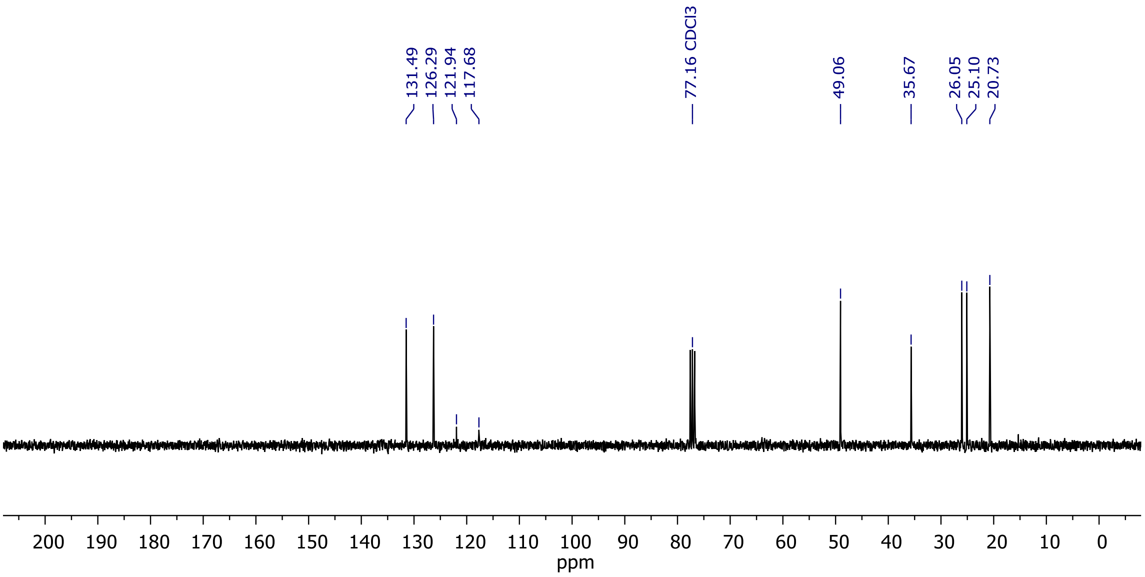

^{1}H NMR (300 MHz, CDCl$_3$)

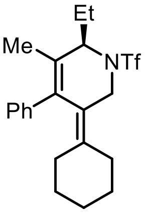

3aa

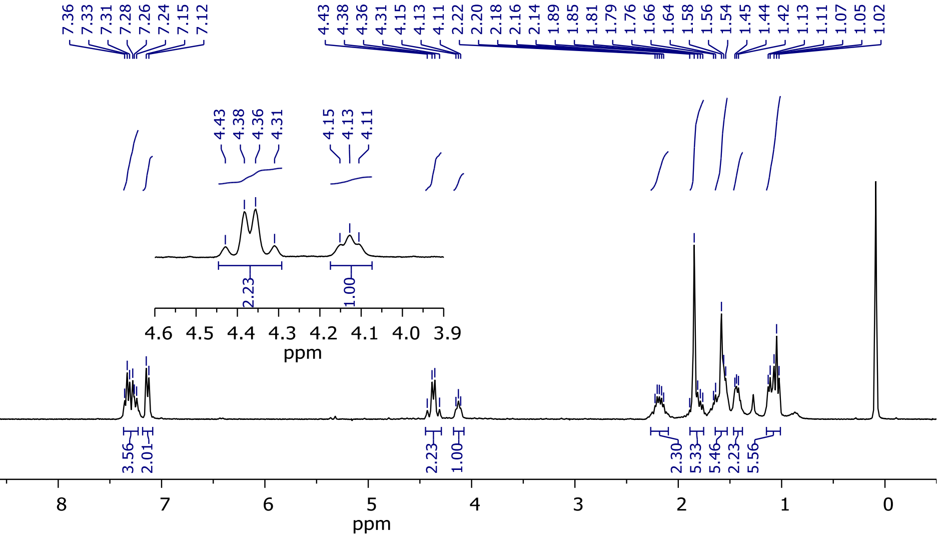

DEPT-135

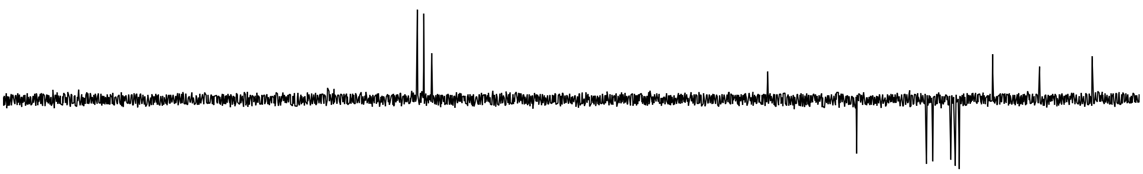

^{13}C NMR (75 MHz, CDCl$_3$)

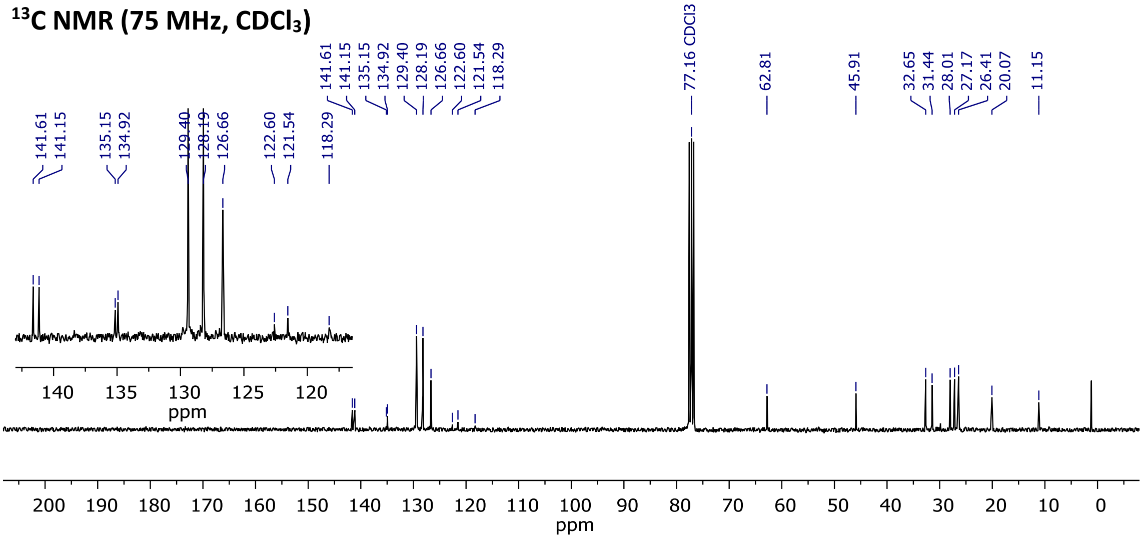

^{1}H NMR (300 MHz, $CDCl_3$)

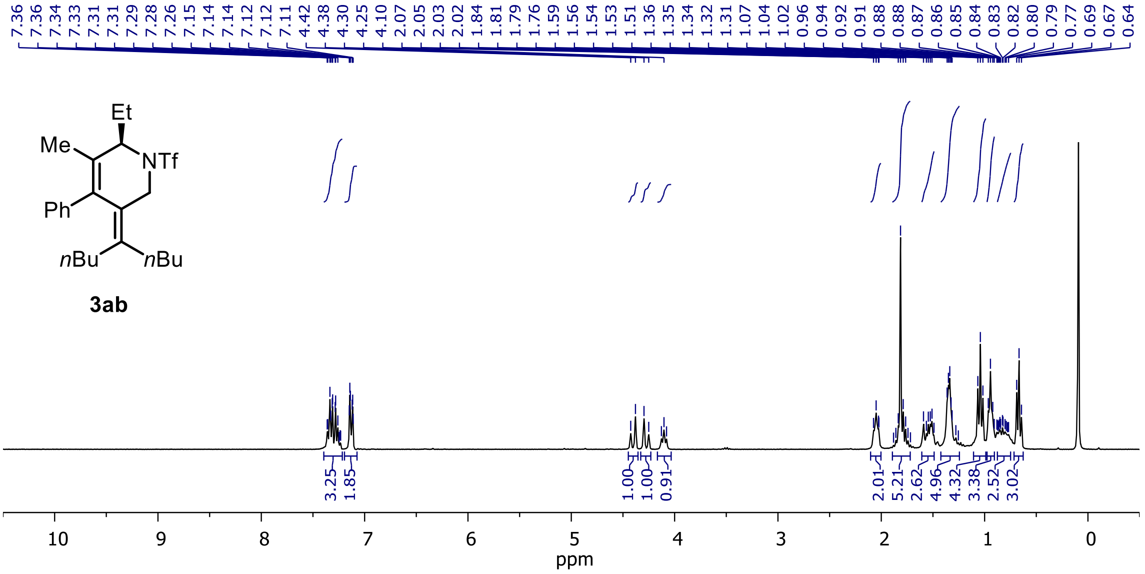

DEPT-135

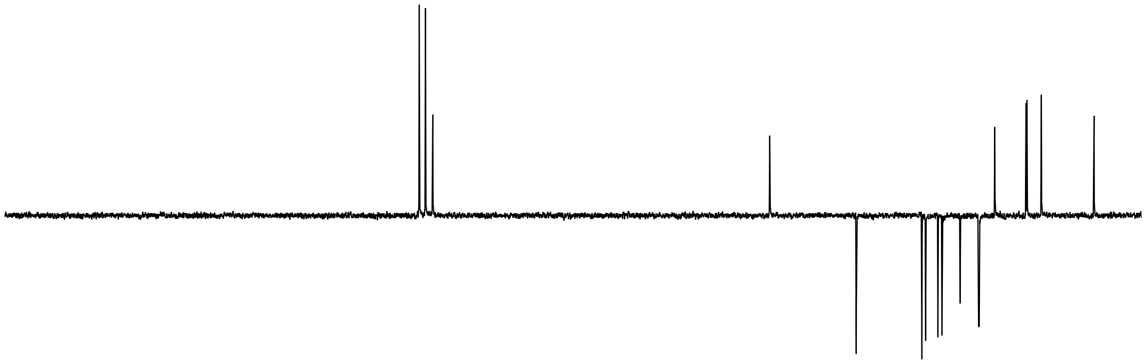

^{13}C NMR (75 MHz, $CDCl_3$)

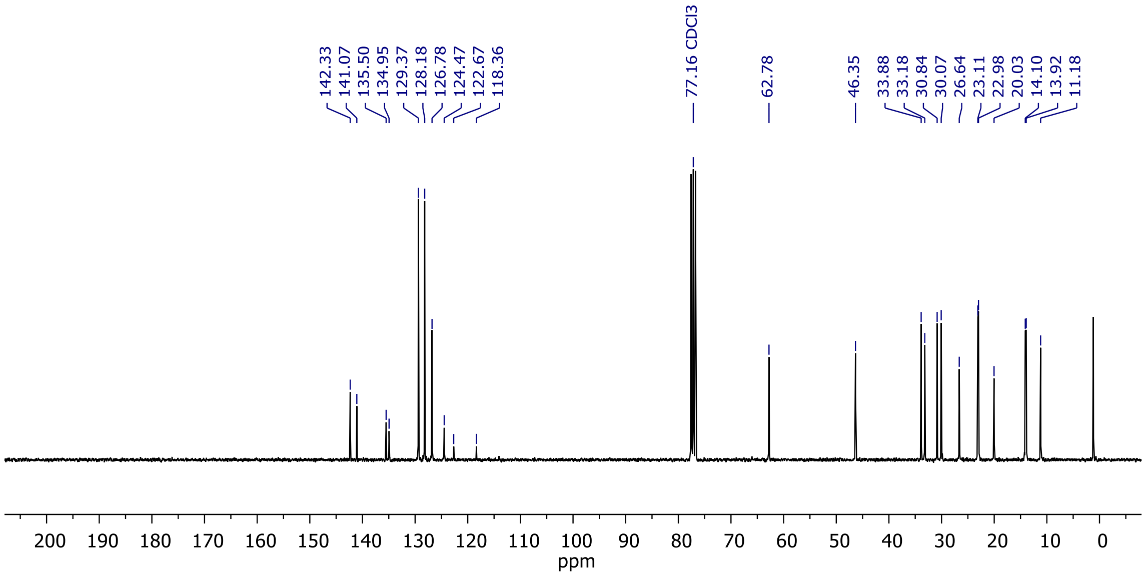

^{1}H NMR (300 MHz, $CDCl_3$)

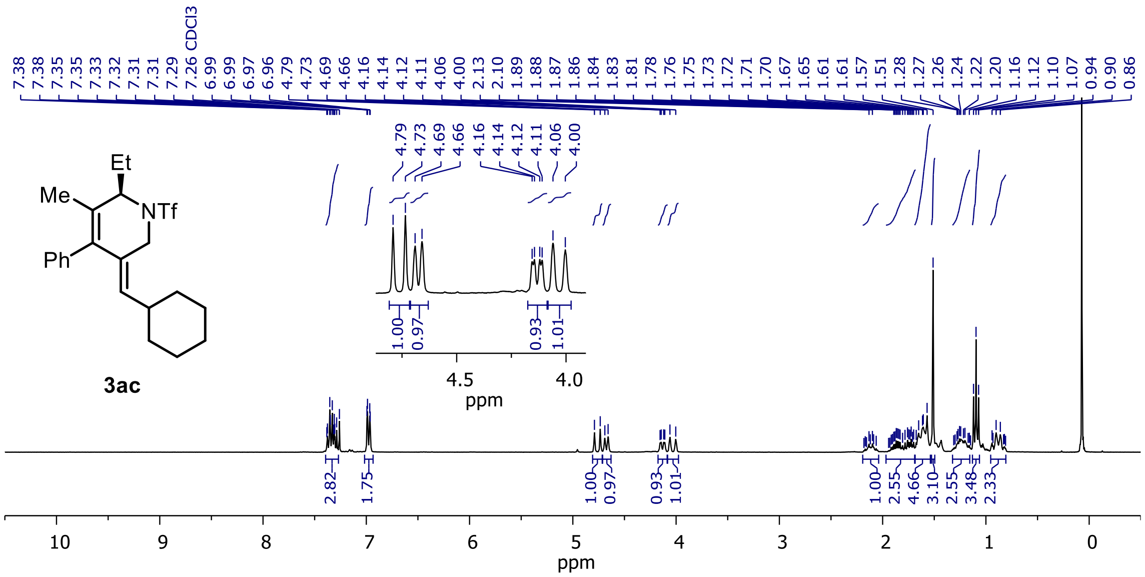

DEPT-135

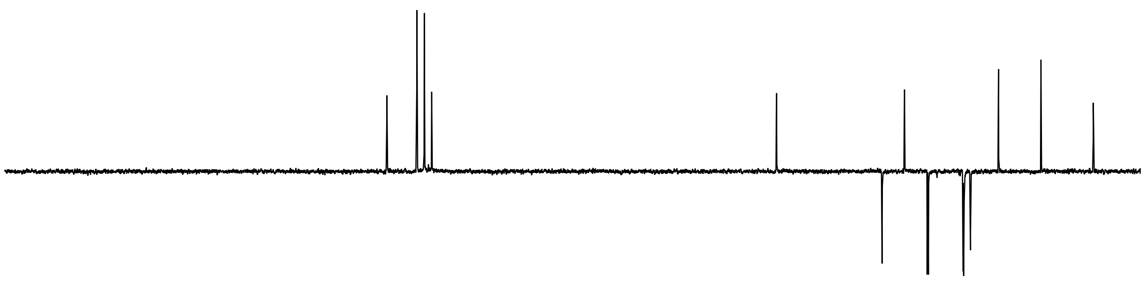

^{13}C NMR (75 MHz, $CDCl_3$)

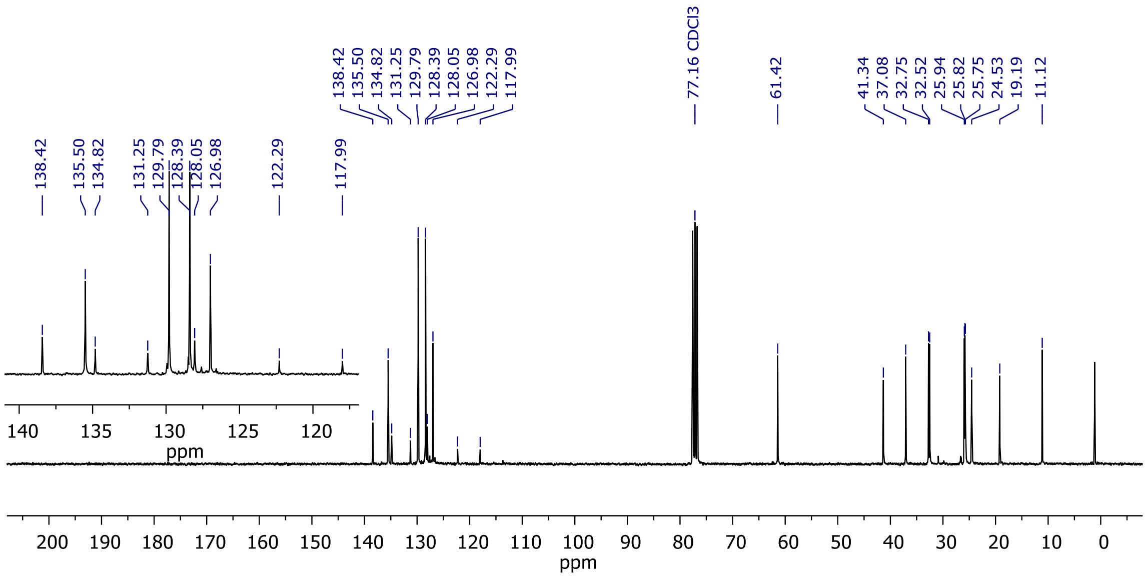

^{1}H NMR (500 MHz, $CDCl_3$)

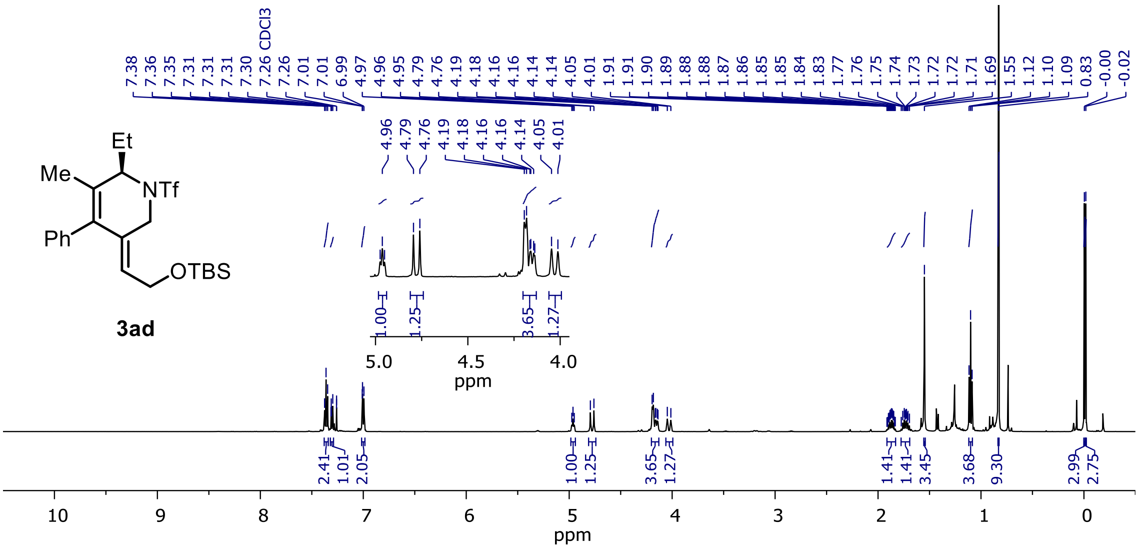

DEPT-135

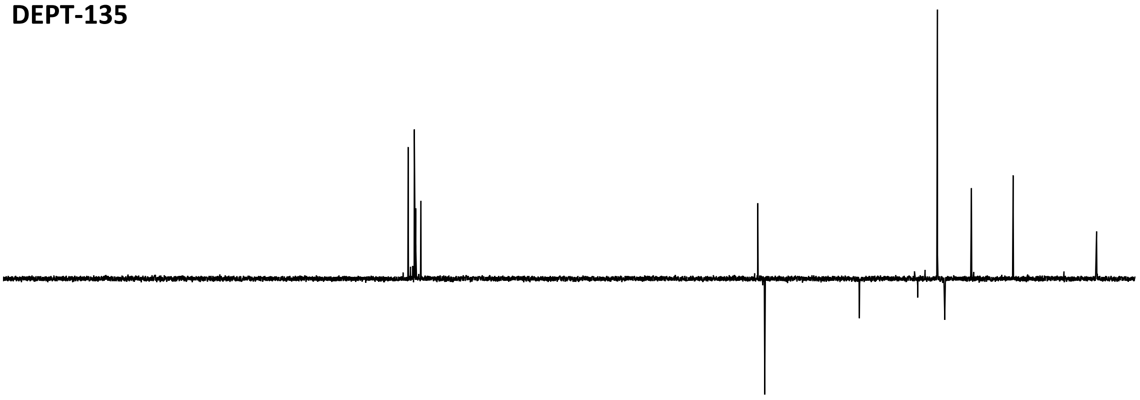

^{13}C NMR (126 MHz, $CDCl_3$)

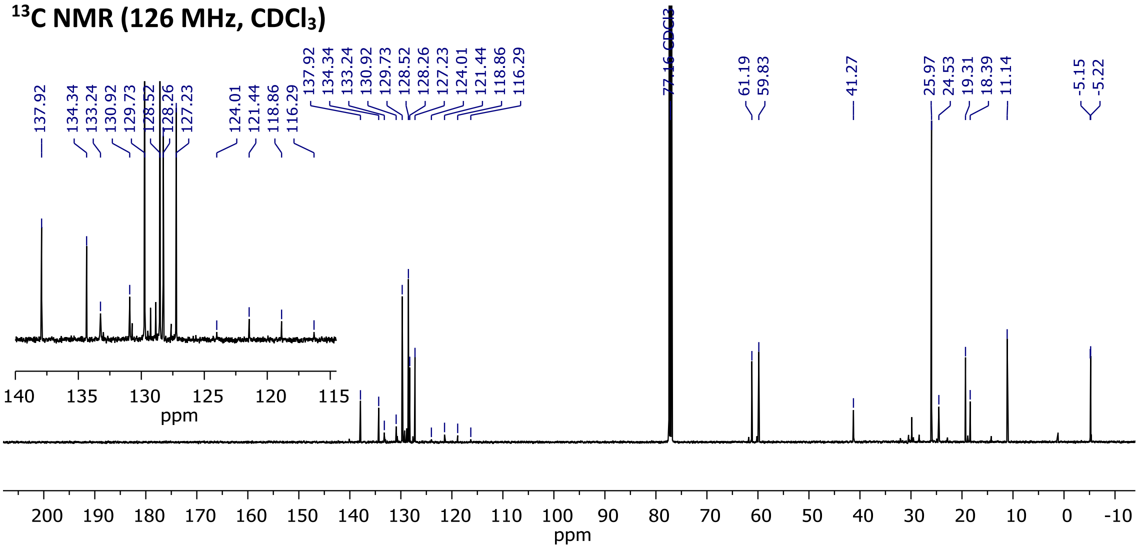

^{1}H NMR (500 MHz, $CDCl_3$)

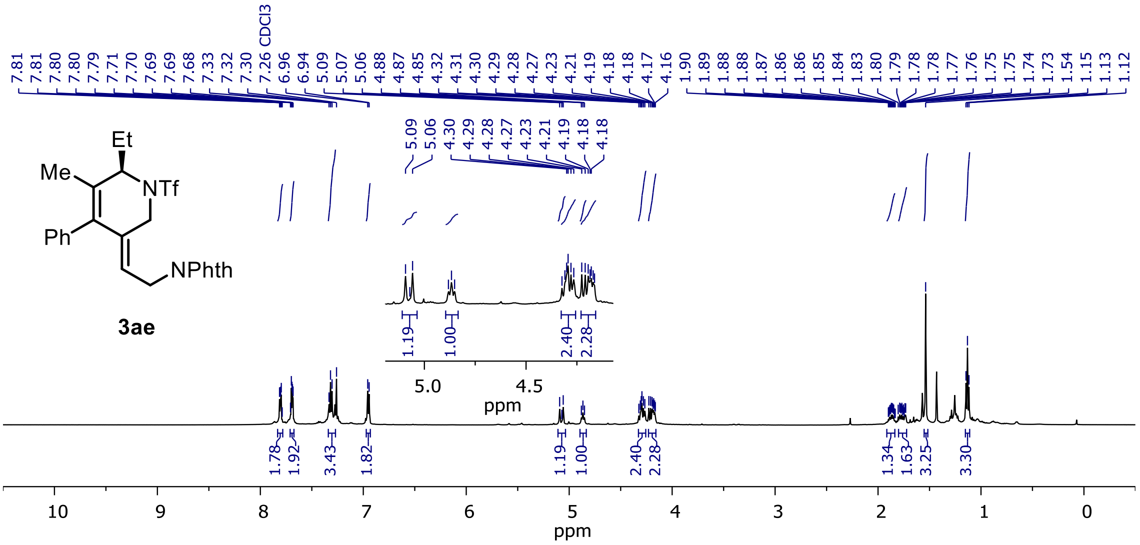

3ae

DEPT-135

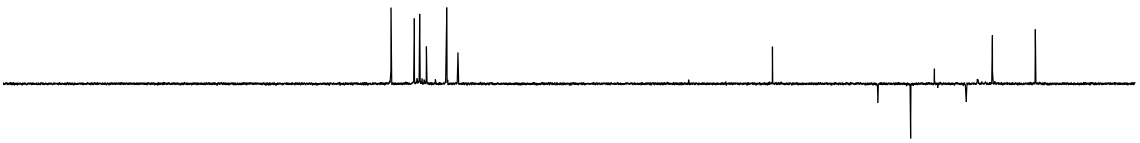

^{13}C NMR (126 MHz, $CDCl_3$)

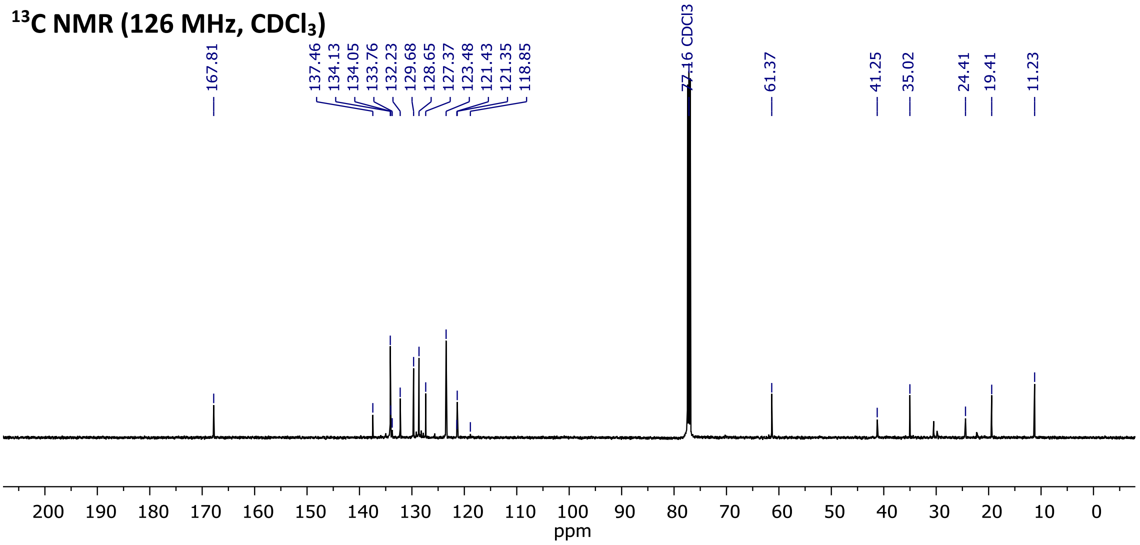

^{1}H NMR (300 MHz, CDCl$_3$)

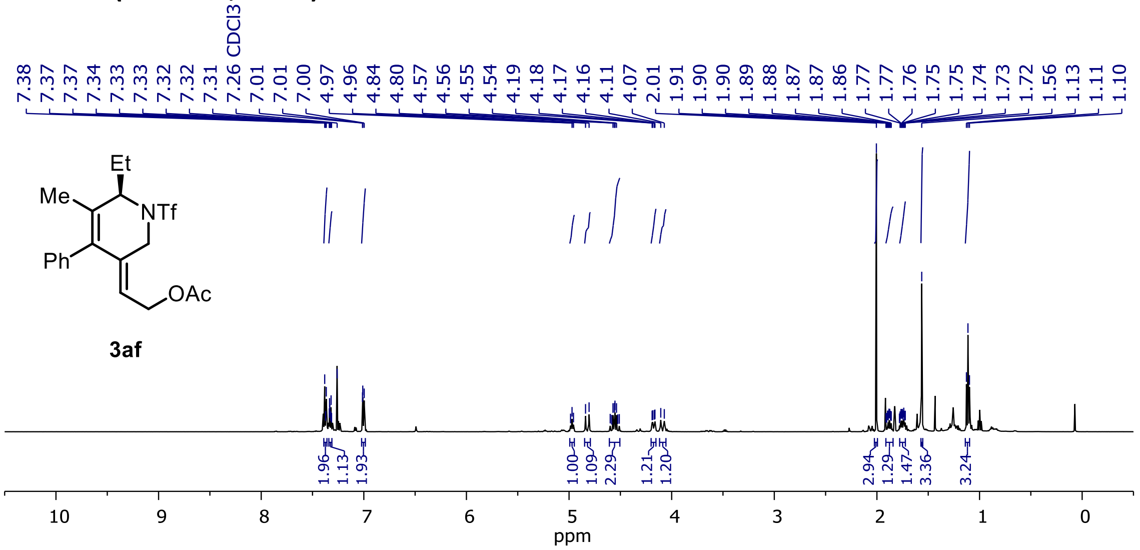

3af

DEPT-135

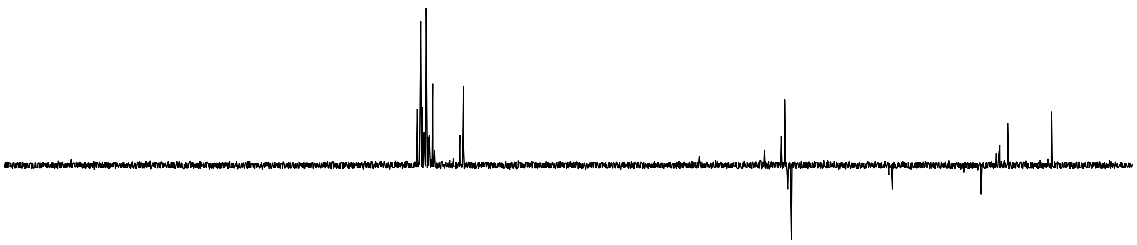

^{13}C NMR (75 MHz, CDCl$_3$)

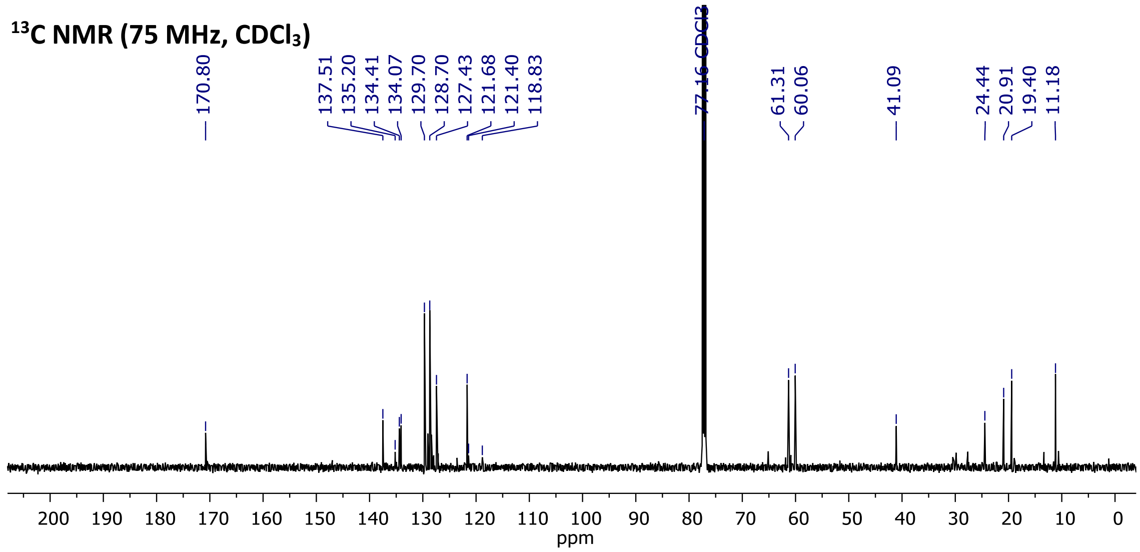

^{1}H NMR (300 MHz, $CDCl_3$)

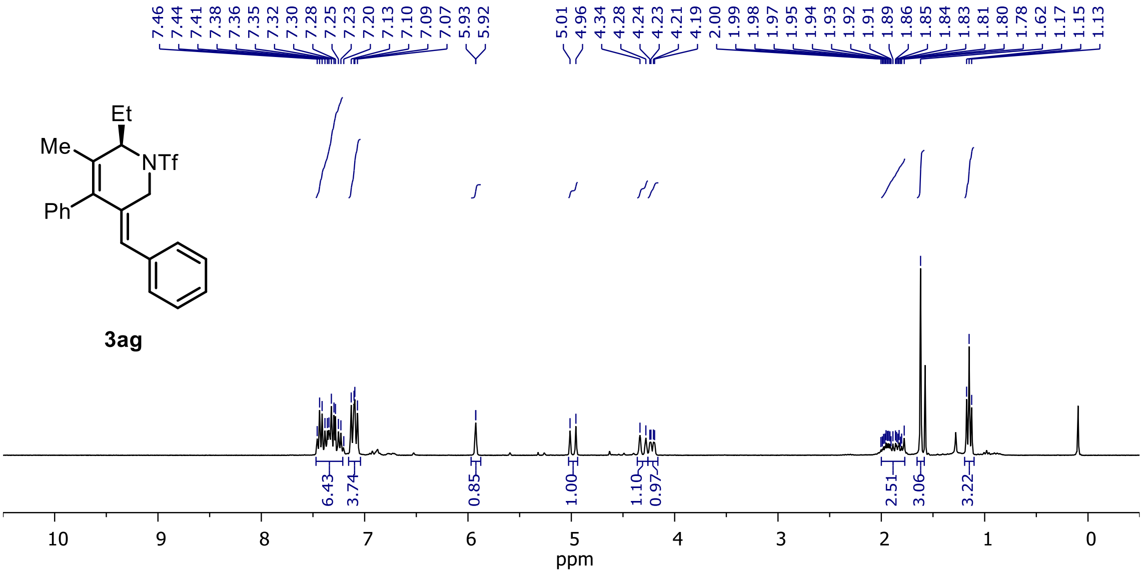

DEPT-135

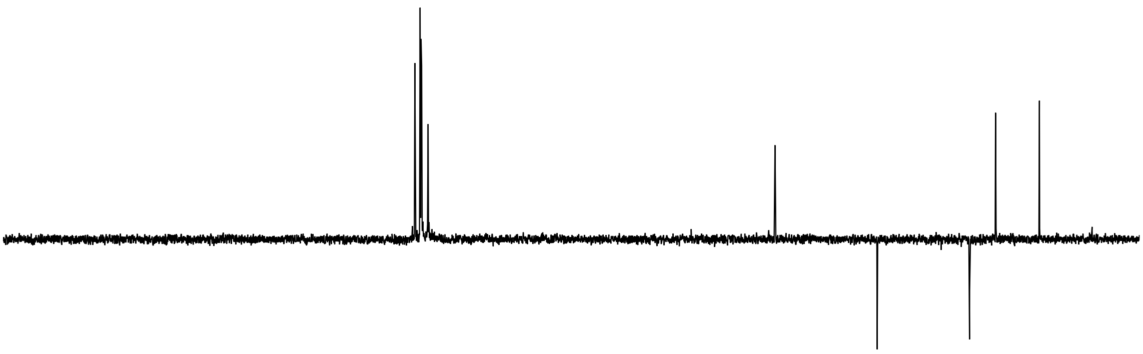

^{13}C NMR (75 MHz, $CDCl_3$)

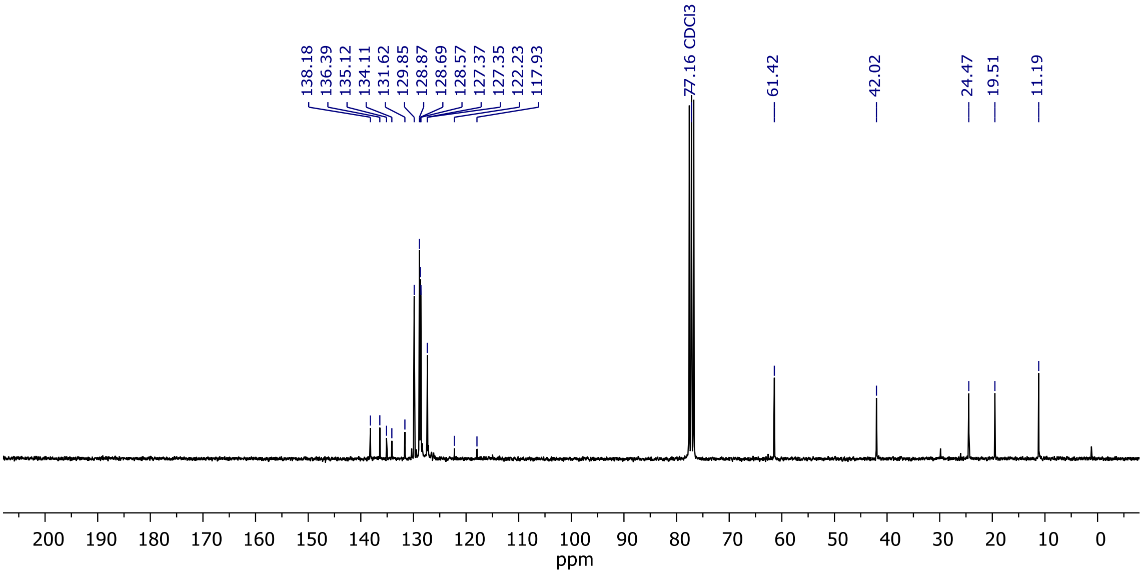

^{1}H NMR (300 MHz, CDCl$_3$)

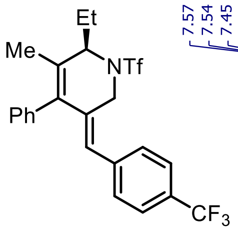

3ah

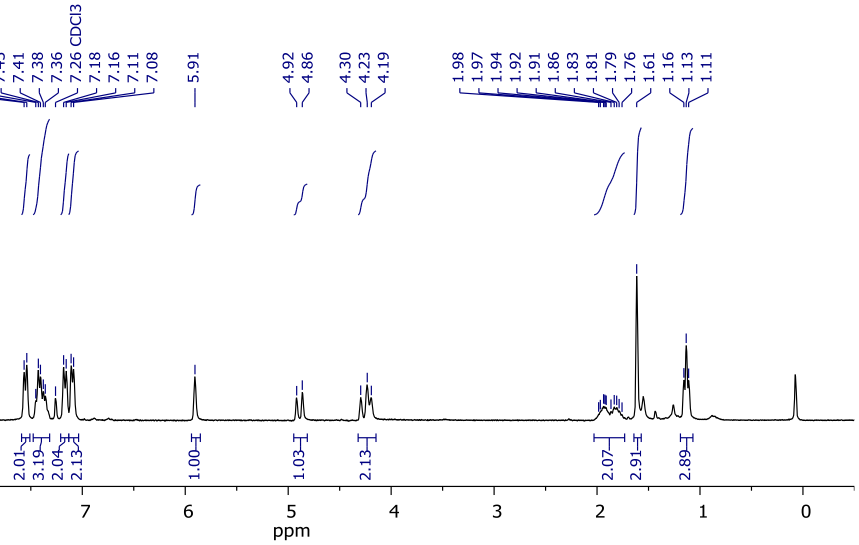

DEPT-135

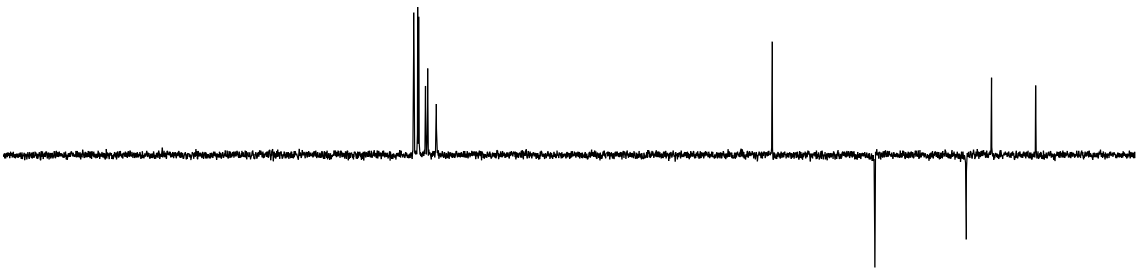

^{13}C NMR (75 MHz, CDCl$_3$)

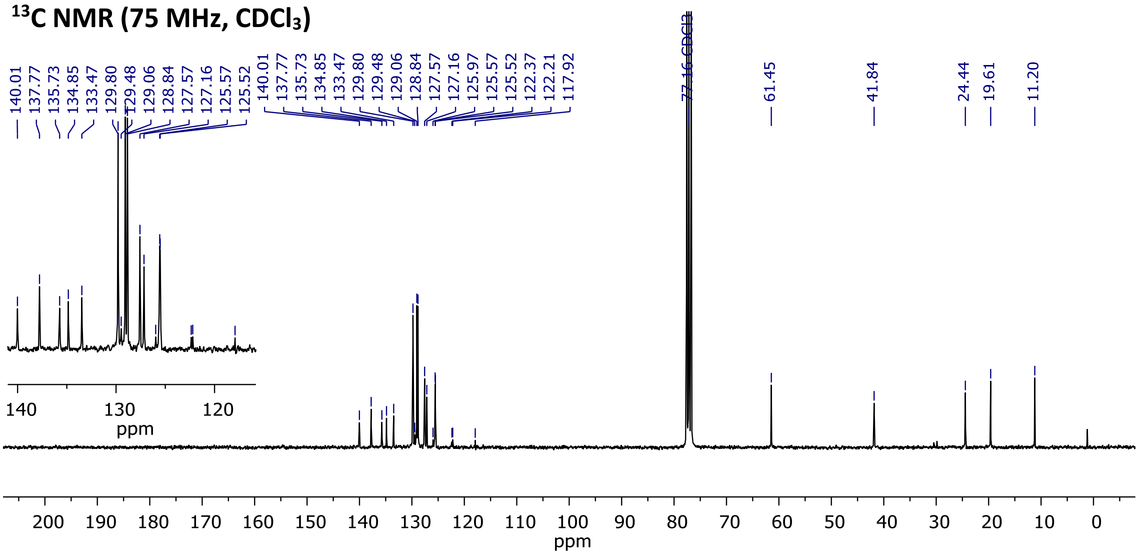

^{1}H NMR (500 MHz, CDCl$_3$)

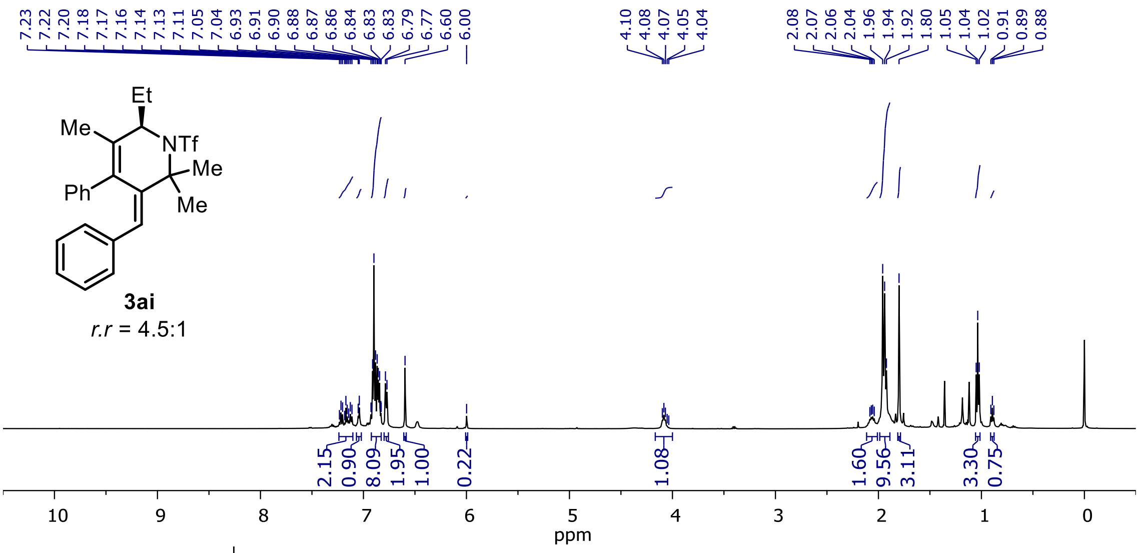

DEPT-135

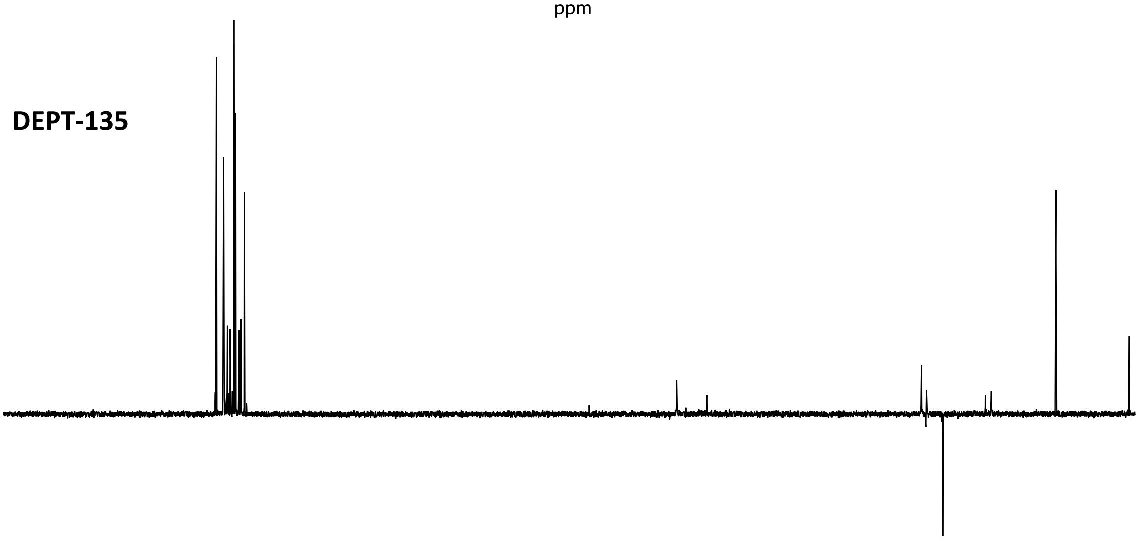

^{13}C NMR (189 MHz, CDCl$_3$)

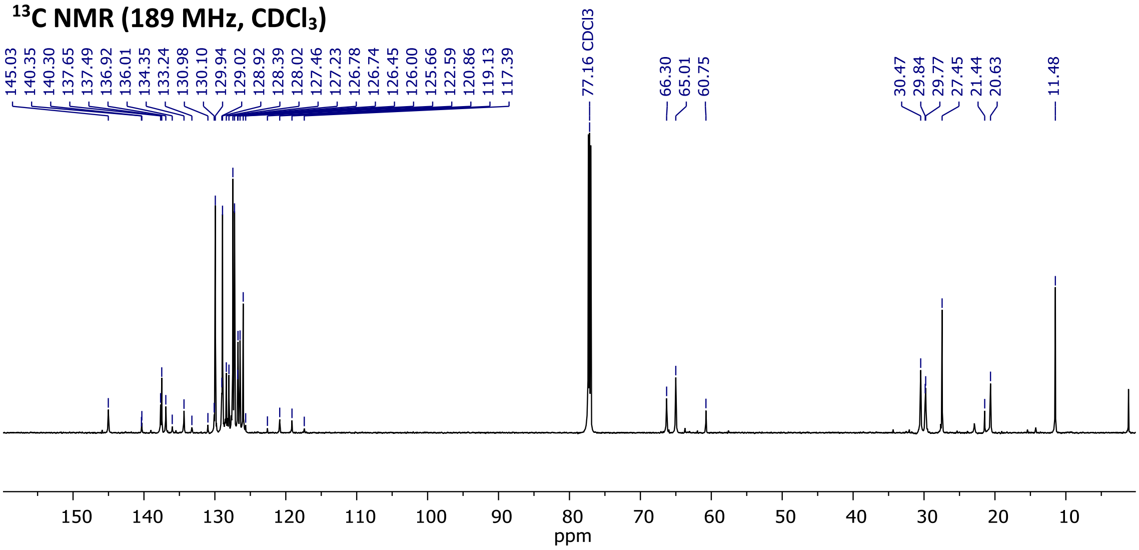

HSQC spectrum ($CDCl_3$)

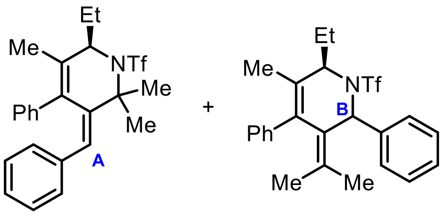

3ai 4.5 : 1 **3ai'**

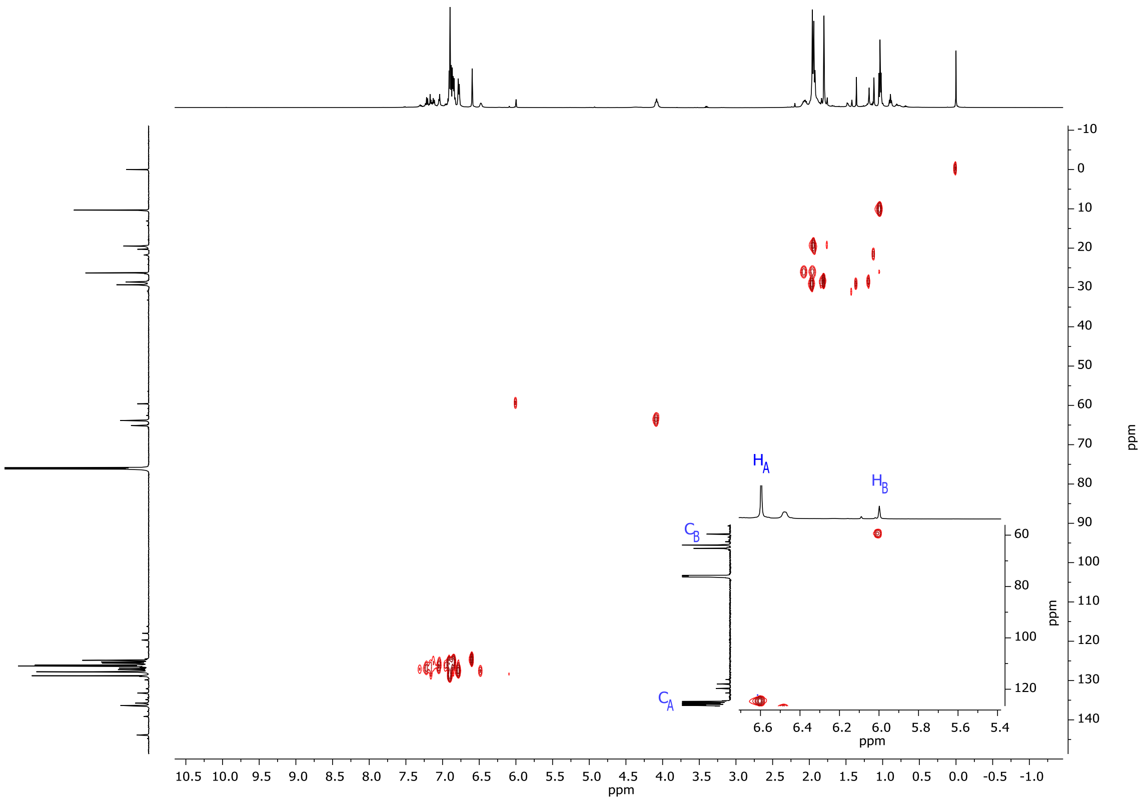

^{1}H NMR (300 MHz, CDCl$_3$)

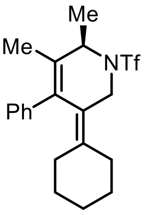

3ba

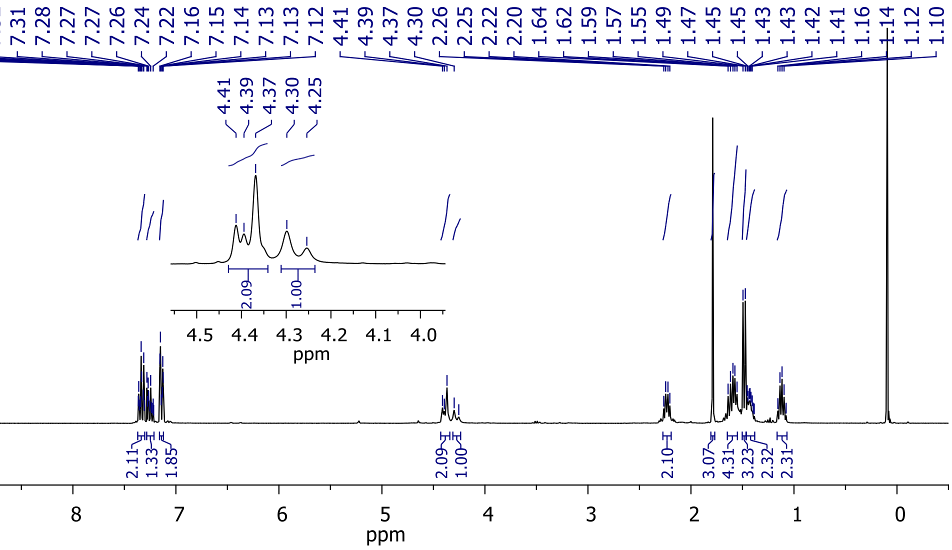

DEPT-135

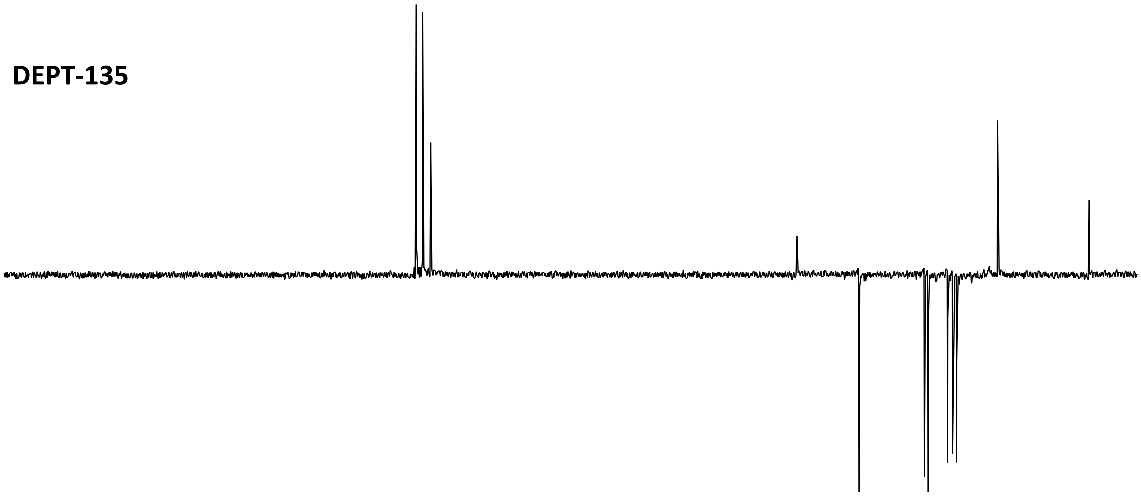

^{13}C NMR (75 MHz, CDCl$_3$)

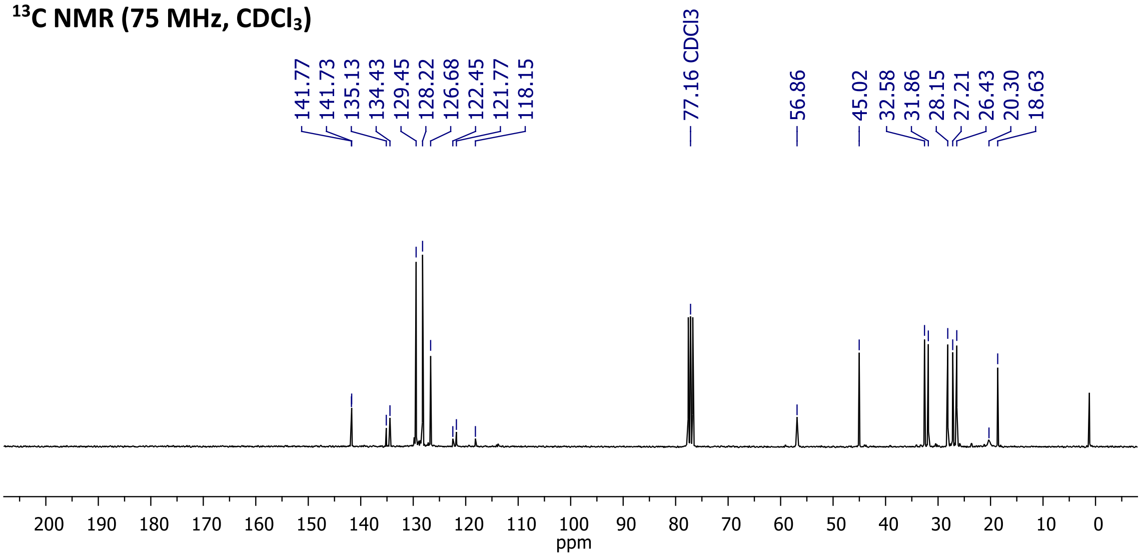

^{1}H NMR (300 MHz, $CDCl_3$)

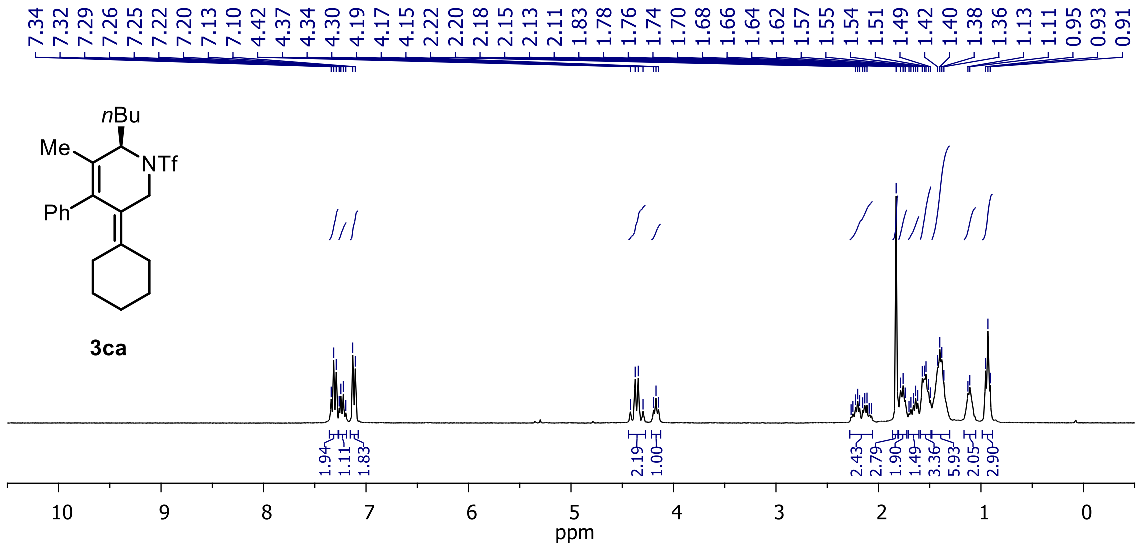

3ca

DEPT-135

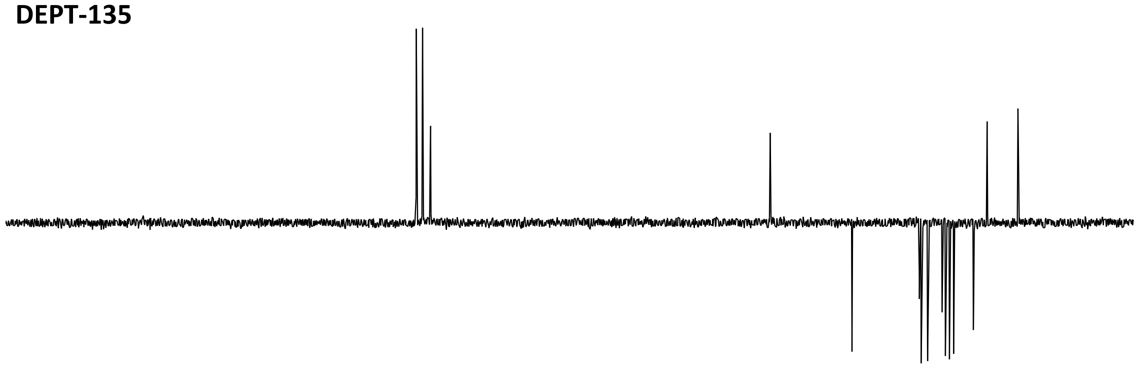

^{13}C NMR (75 MHz, $CDCl_3$)

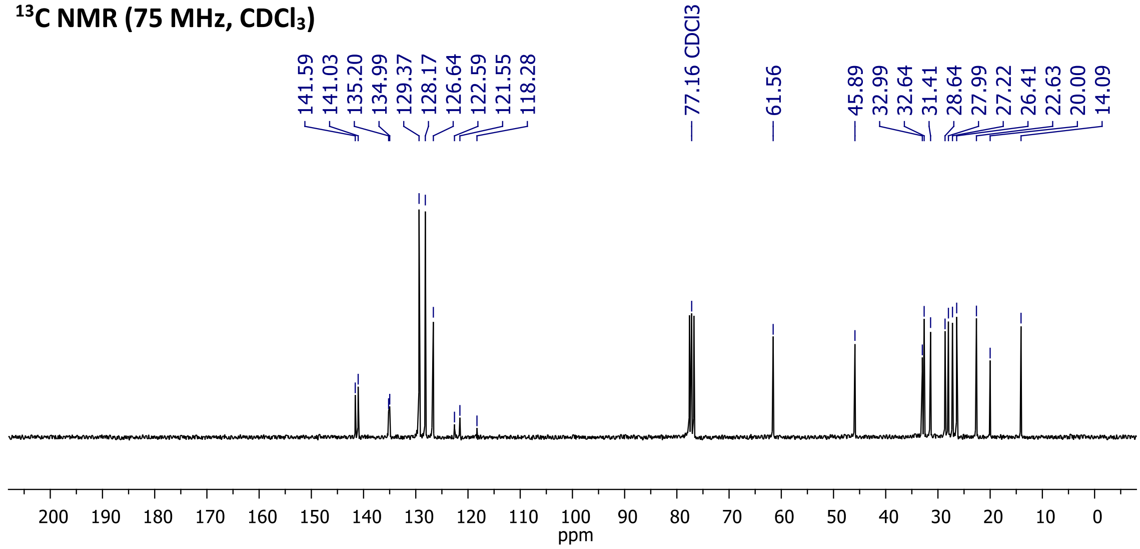

^{1}H NMR (300 MHz, $CDCl_3$)

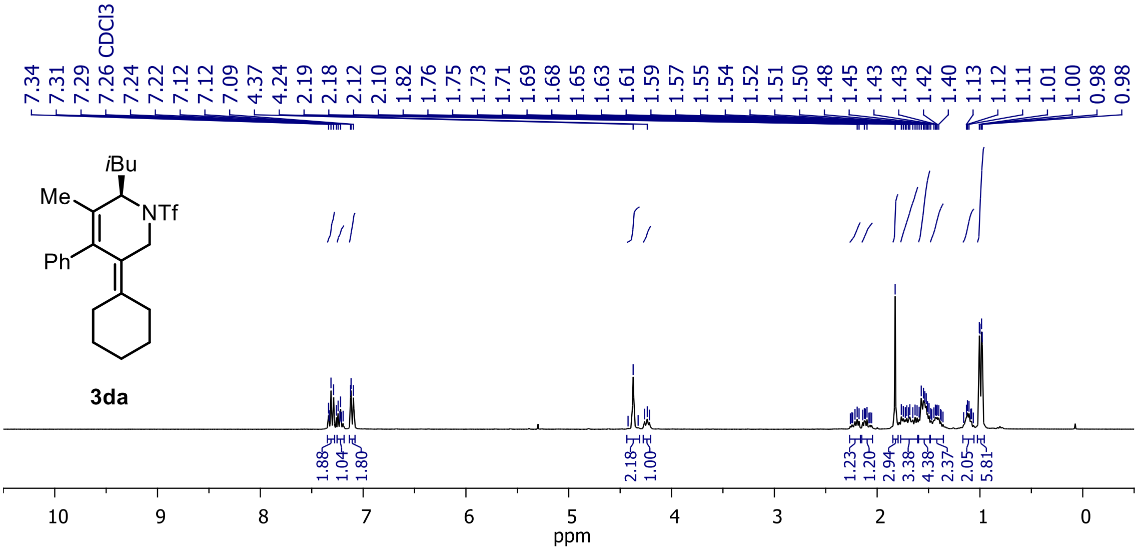

DEPT-135

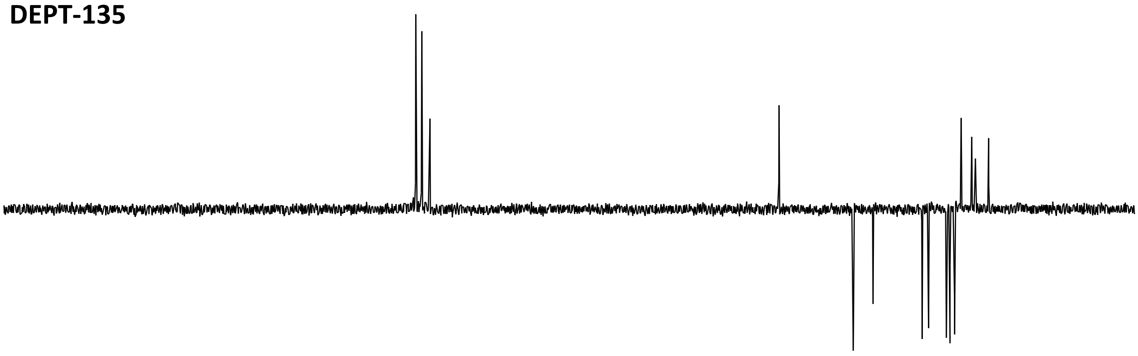

^{13}C NMR (75 MHz, $CDCl_3$)

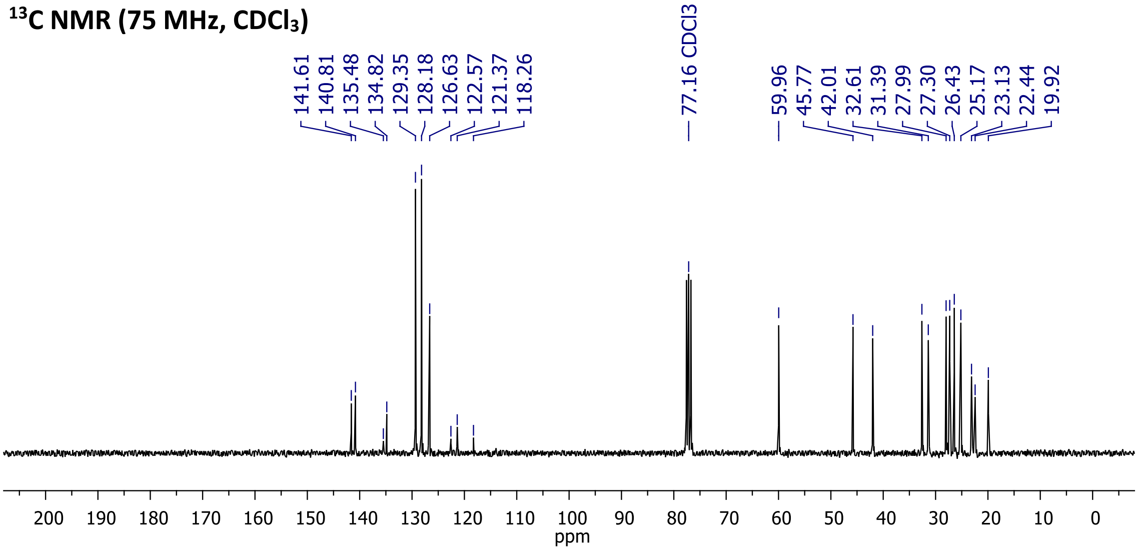

^{1}H NMR (300 MHz, CDCl$_3$)

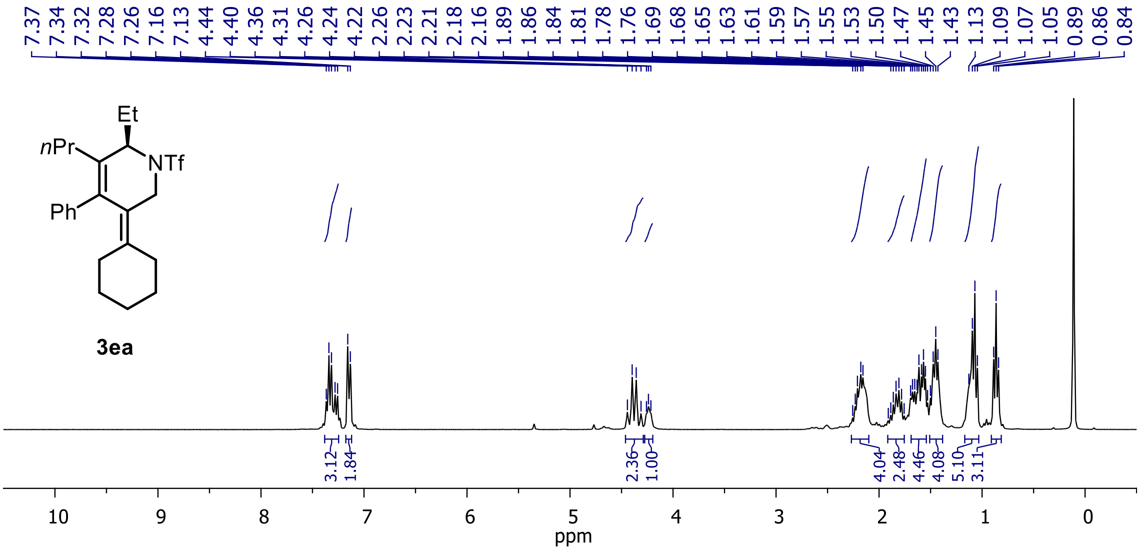

DEPT-135

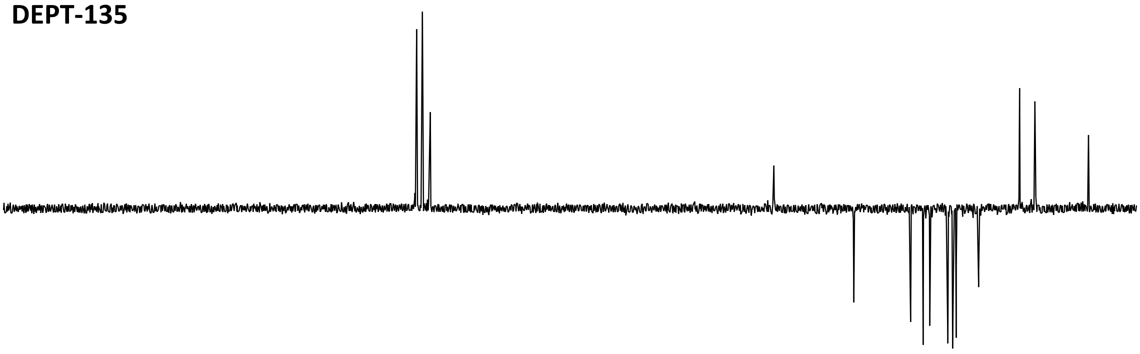

^{13}C NMR (75 MHz, CDCl$_3$)

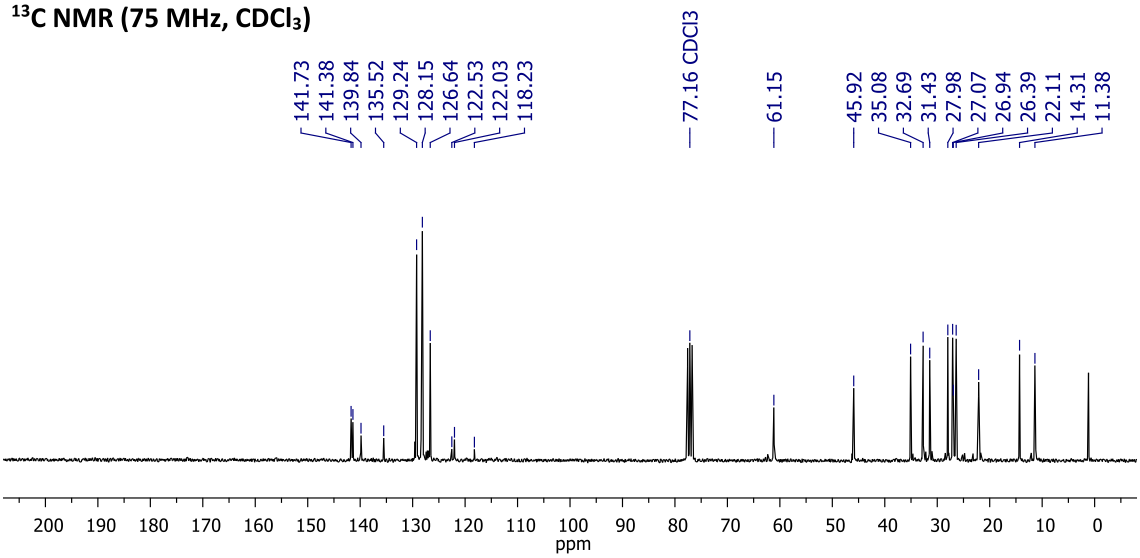

^{1}H NMR (300 MHz, $CDCl_3$)

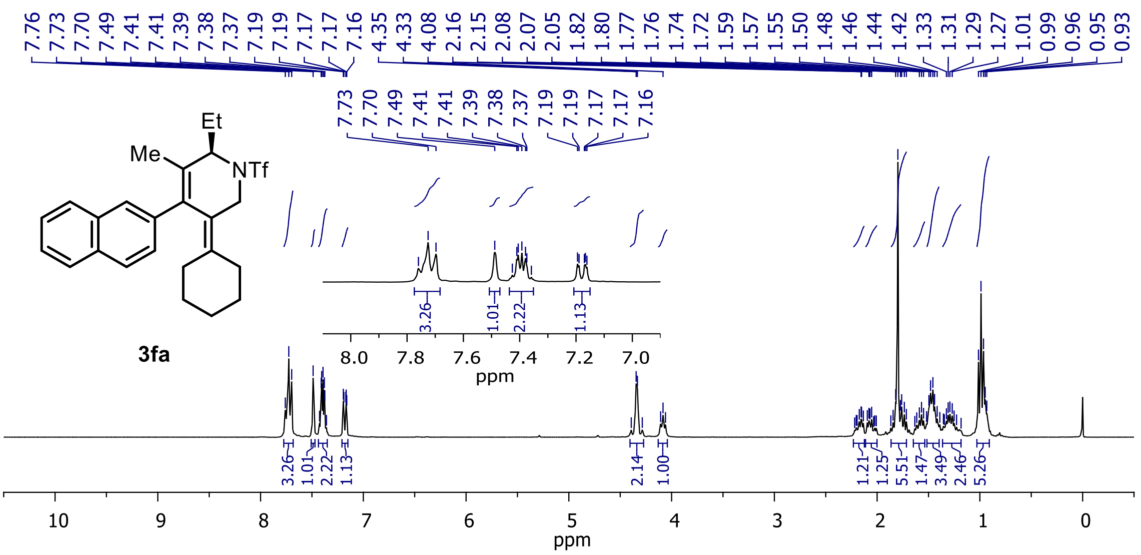

DEPT-135

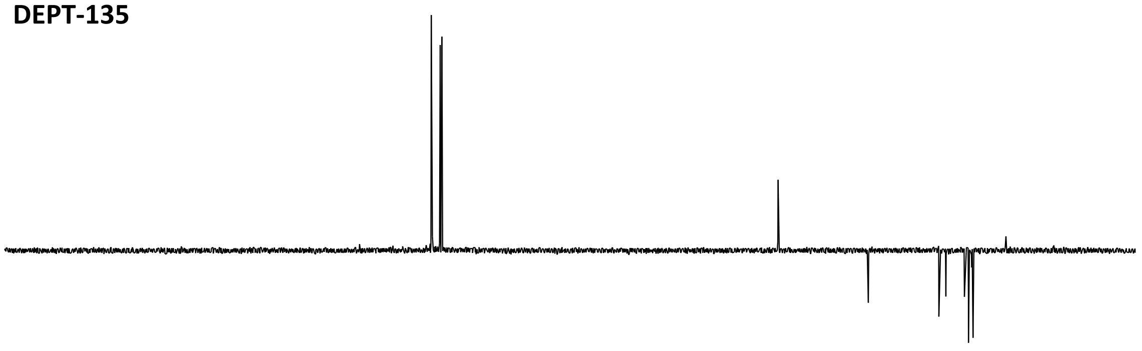

^{13}C NMR (75 MHz, $CDCl_3$)

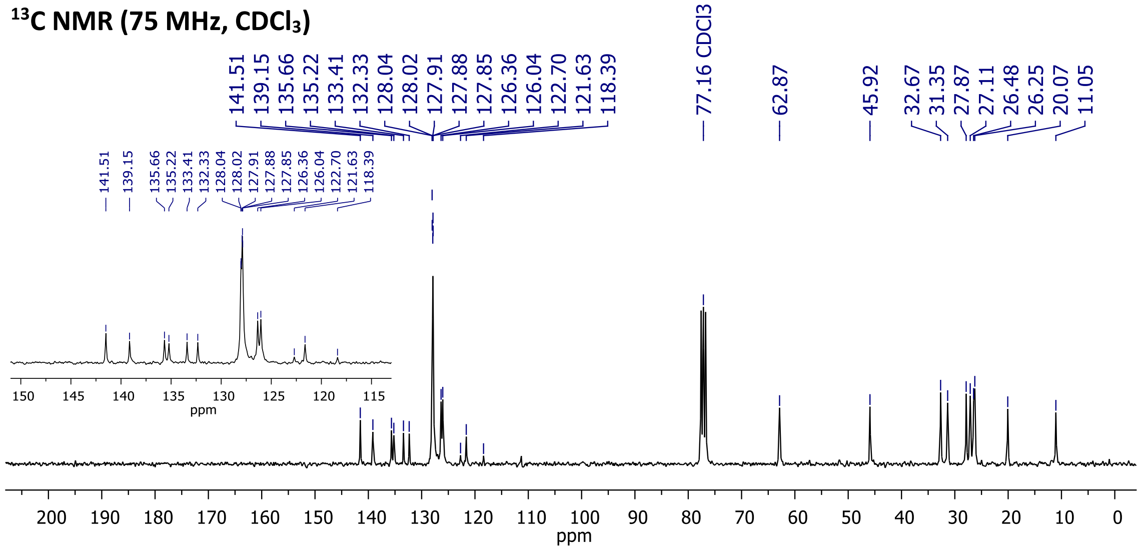

^{1}H NMR (300 MHz, $CDCl_3$)

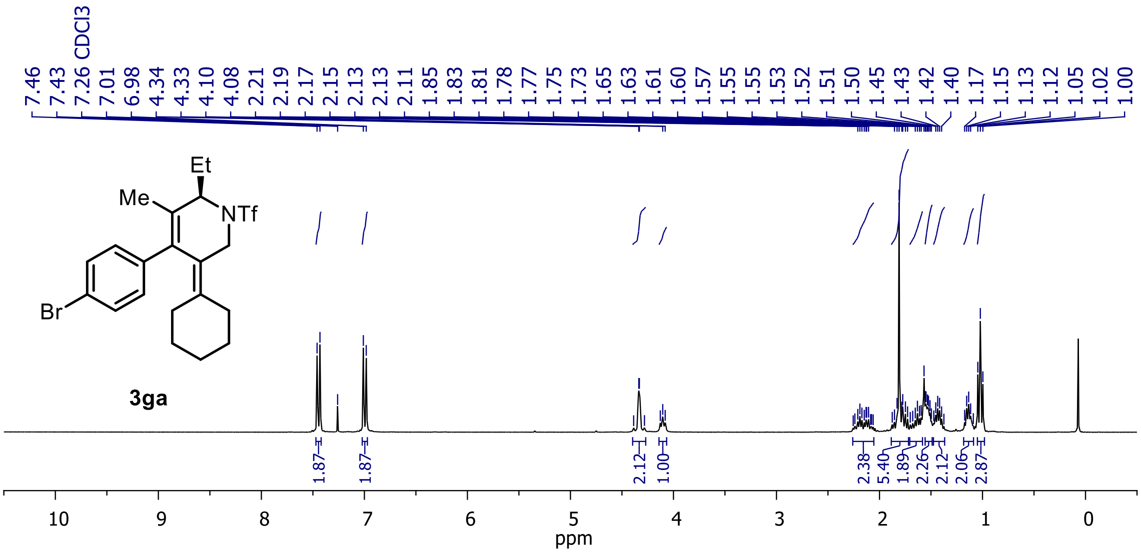

DEPT-135

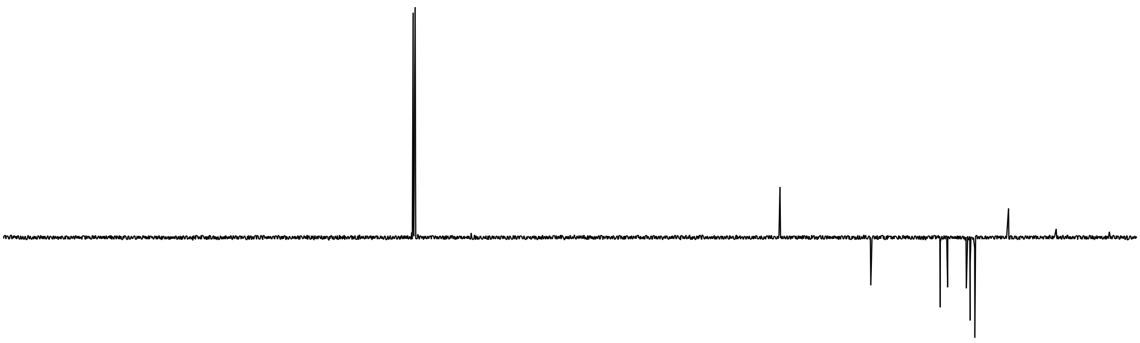

^{13}C NMR (75 MHz, $CDCl_3$)

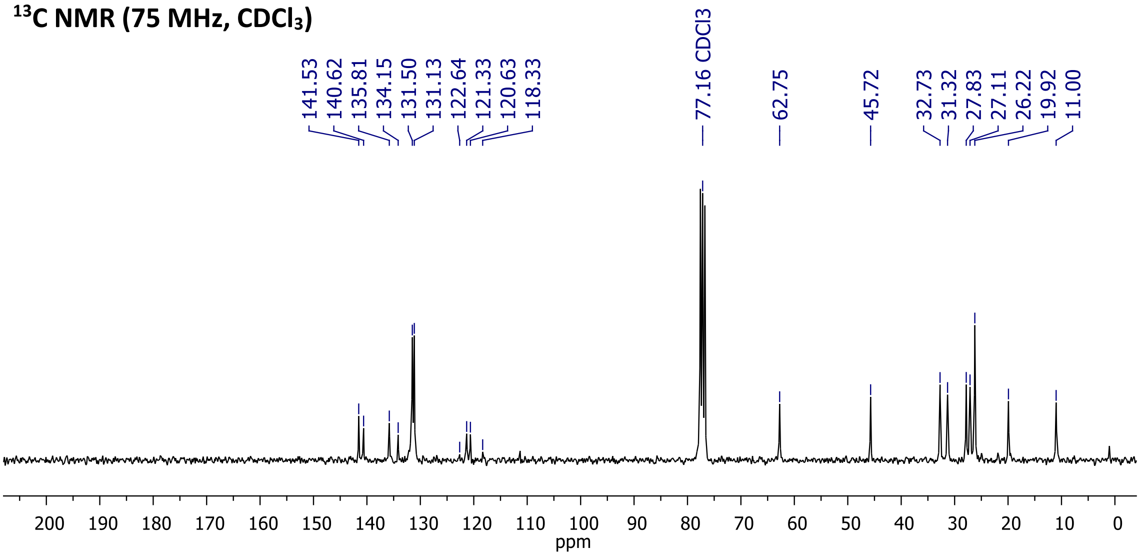

^{1}H NMR (300 MHz, CDCl$_3$)

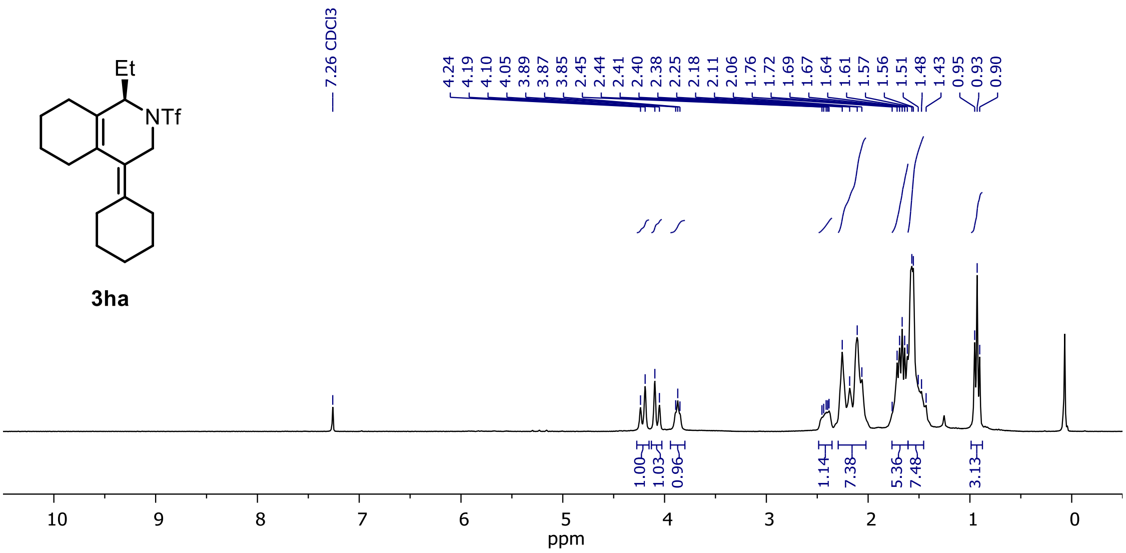

3ha

DEPT-135

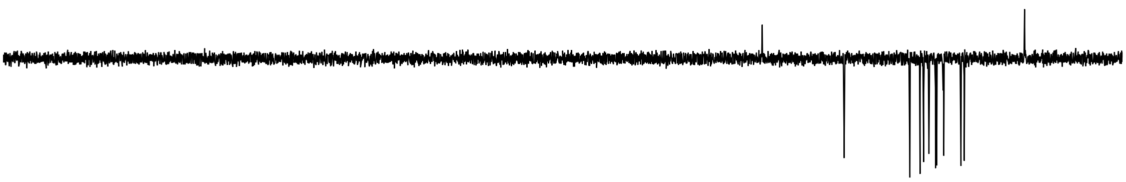

^{13}C NMR (75 MHz, CDCl$_3$)

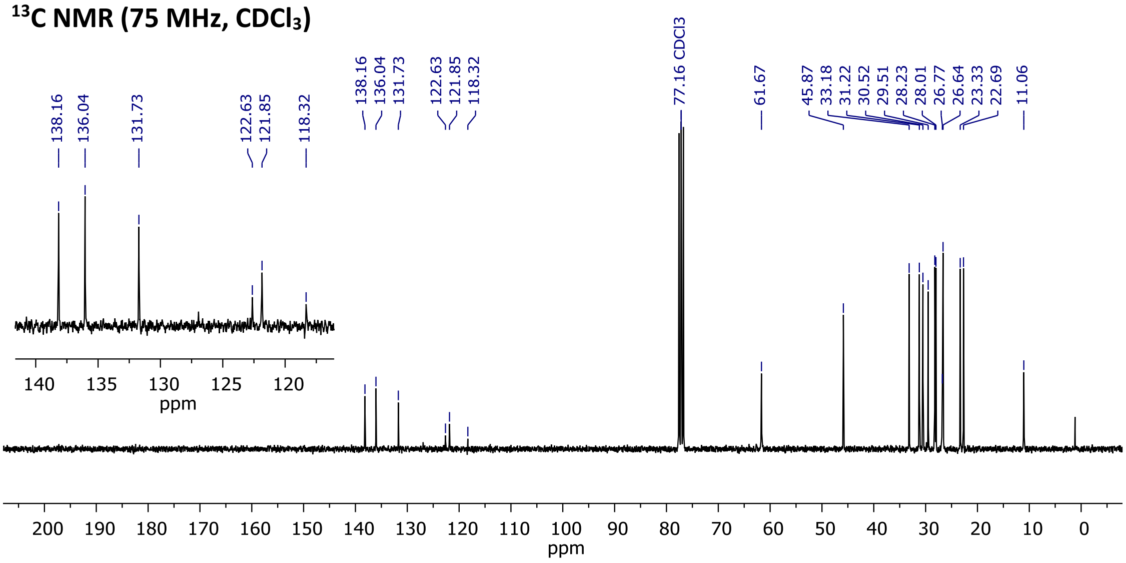

^{1}H NMR (300 MHz, $CDCl_3$)

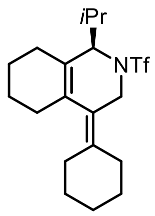

3ia

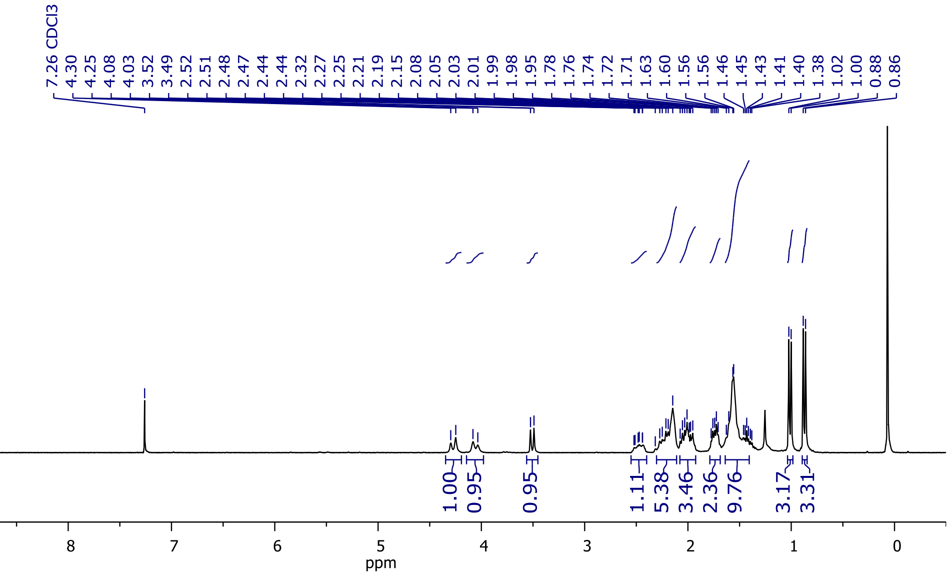

DEPT-135

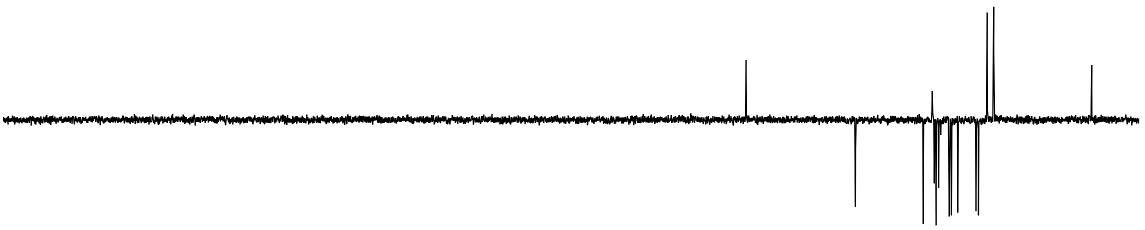

^{13}C NMR (75 MHz, $CDCl_3$)

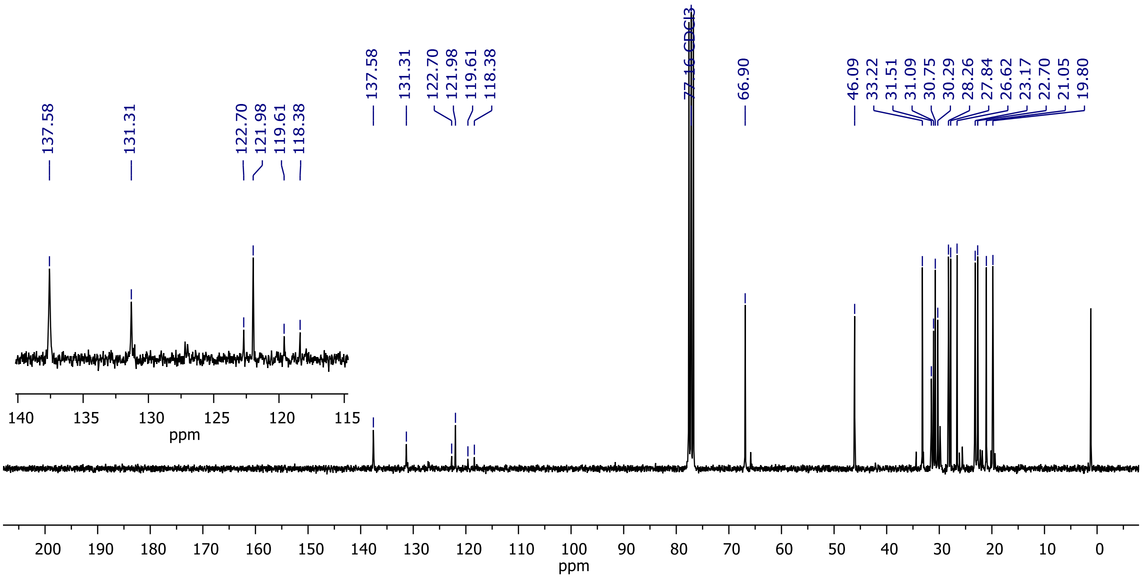

^{1}H NMR (300 MHz, $CDCl_3$)

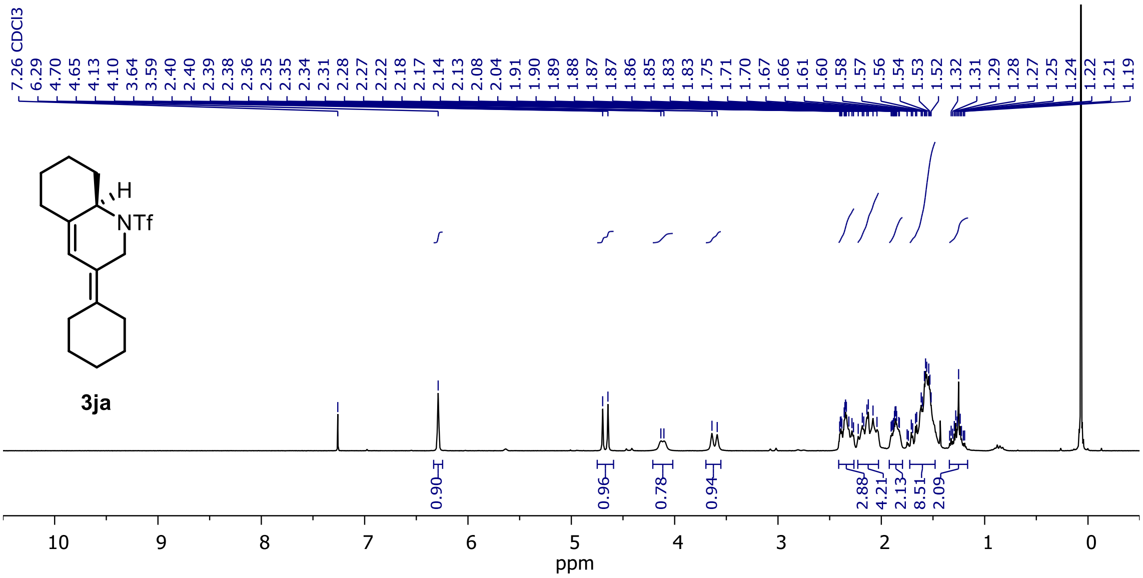

DEPT-135

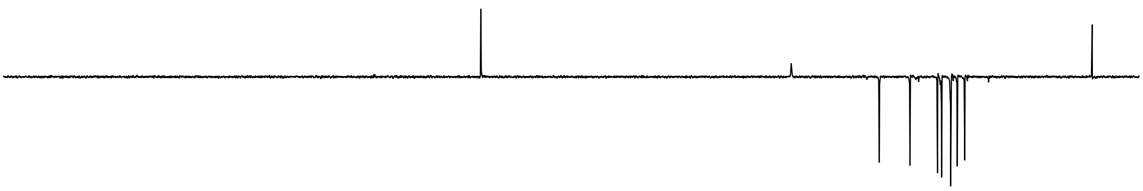

^{13}C NMR (75 MHz, $CDCl_3$)

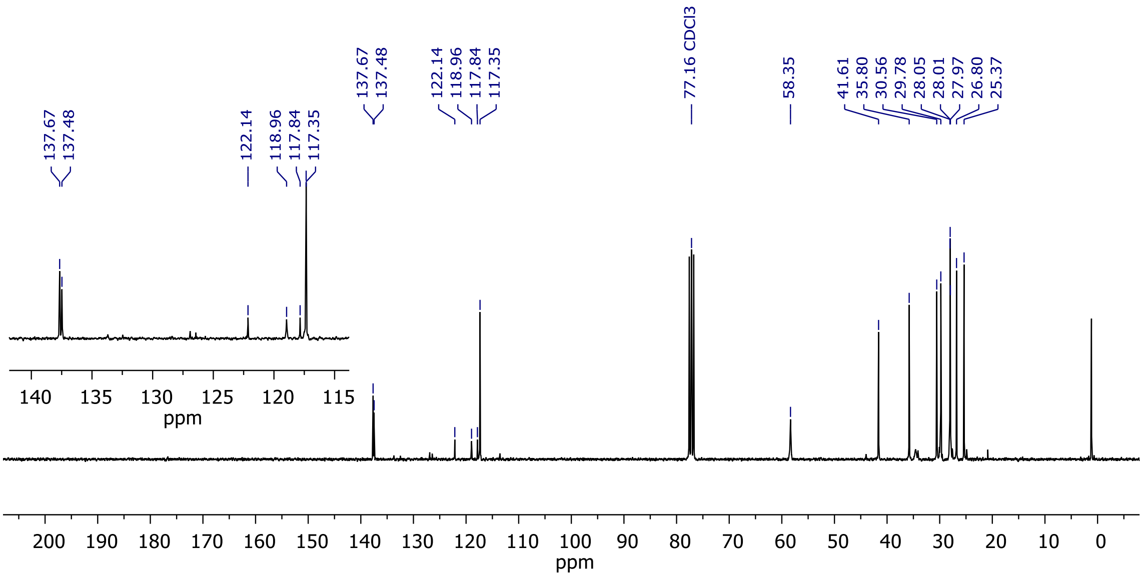

^{1}H NMR (300 MHz, $CDCl_3$)

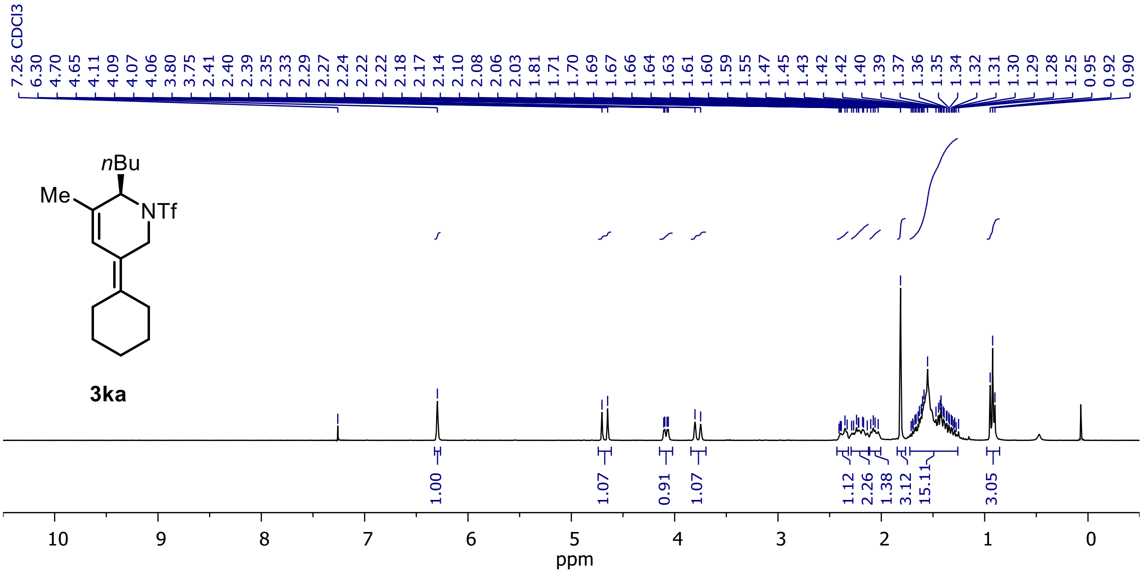

3ka

DEPT-135

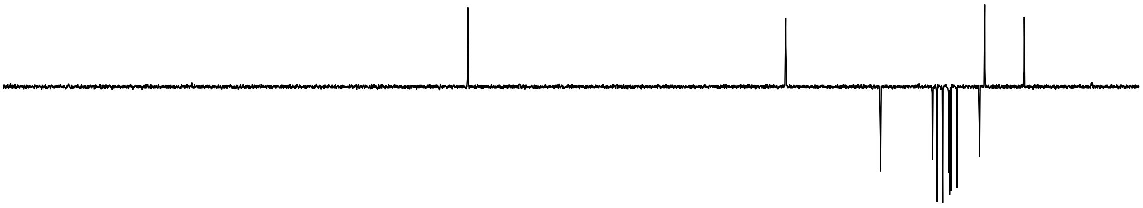

^{13}C NMR (75 MHz, $CDCl_3$)

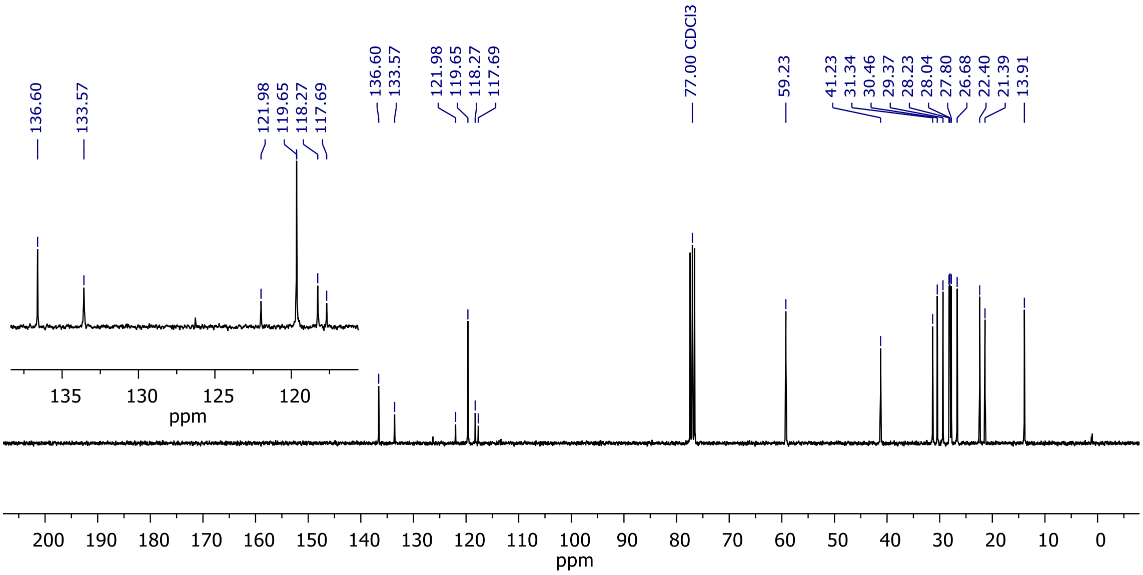

^{1}H NMR (300 MHz, $CDCl_3$)

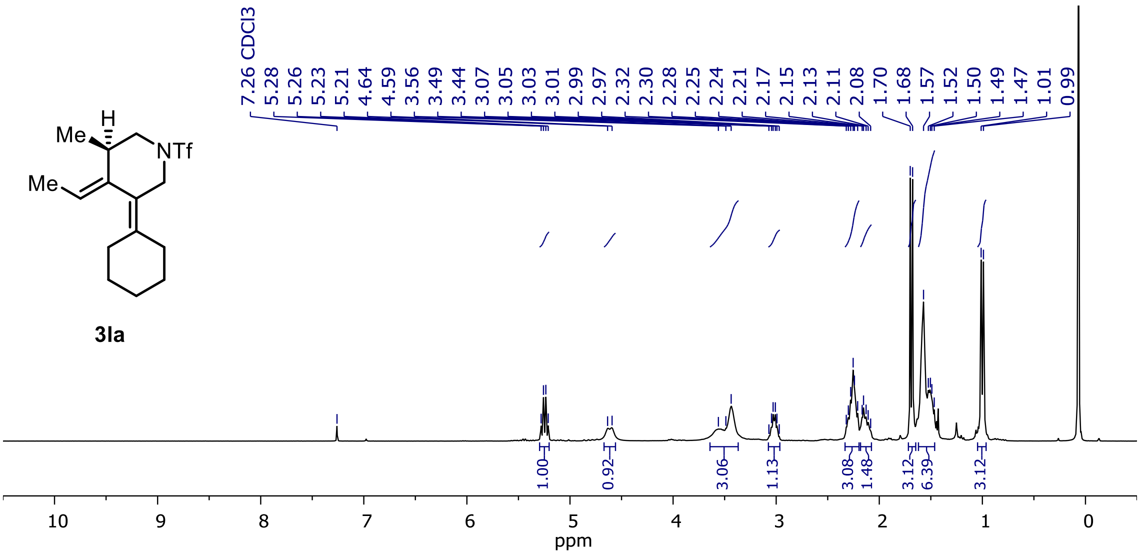

DEPT-135

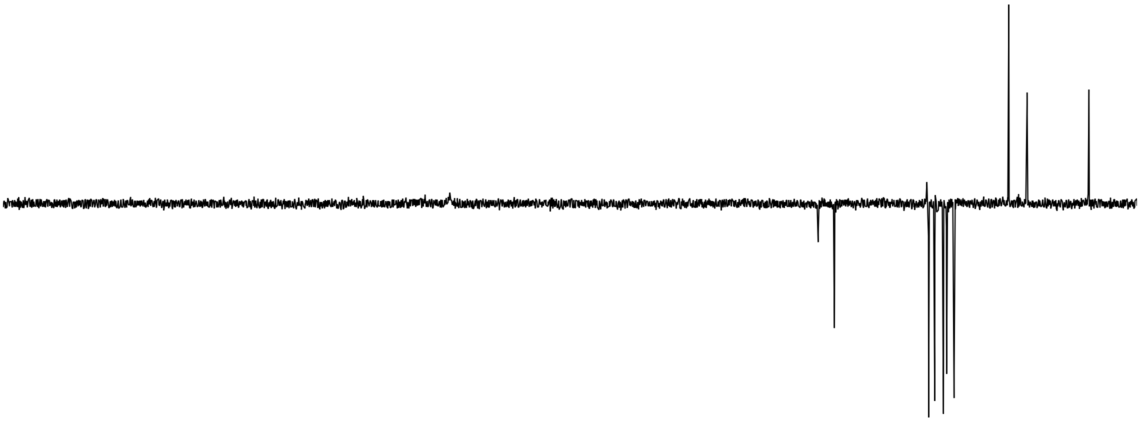

^{13}C NMR (75 MHz, $CDCl_3$)

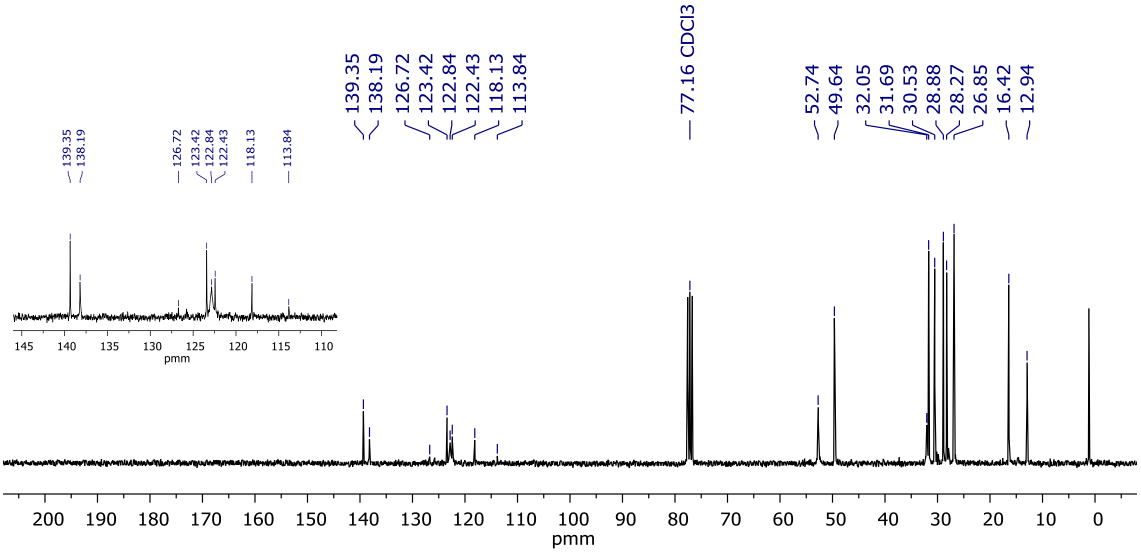

^{1}H NMR (300 MHz, $CDCl_3$)

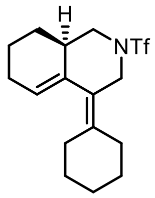

3ma

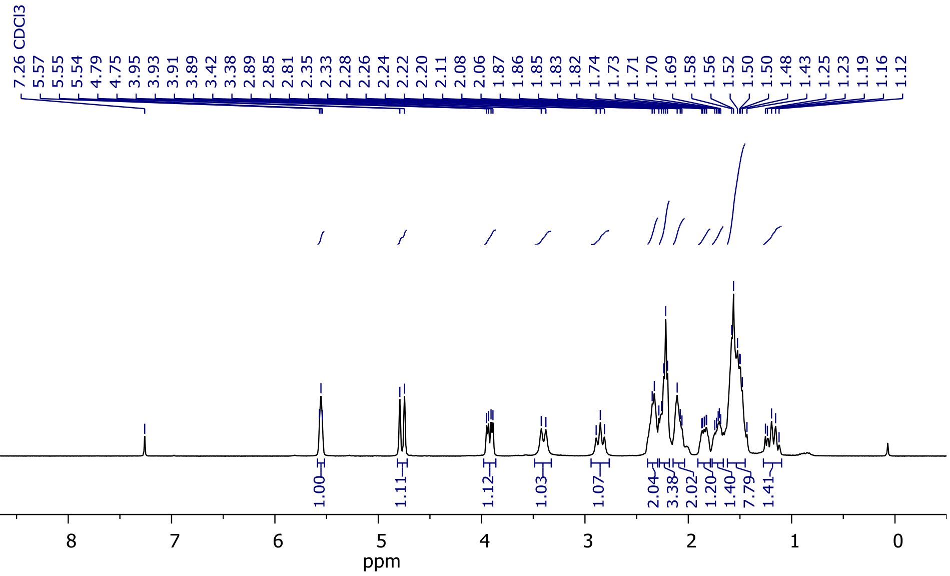

DEPT-135

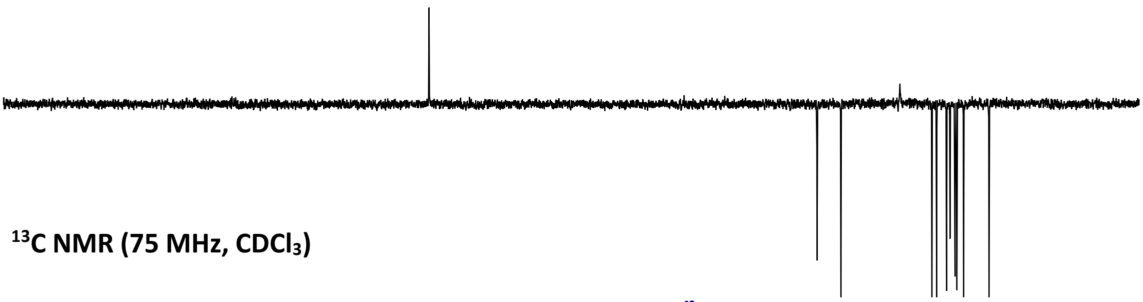

^{13}C NMR (75 MHz, $CDCl_3$)

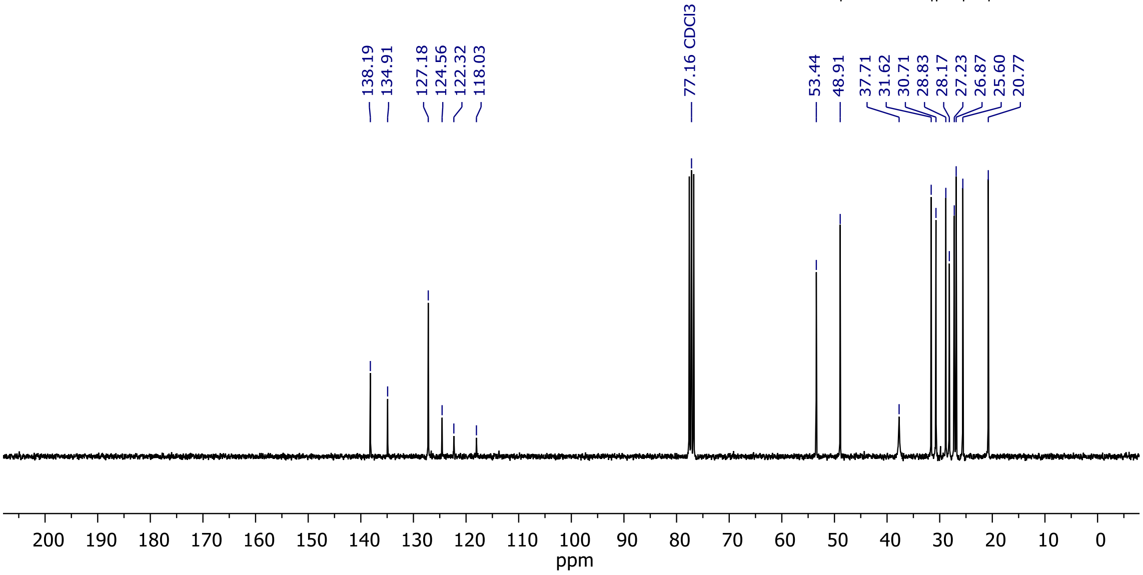

^{1}H NMR (300 MHz, $CDCl_3$)

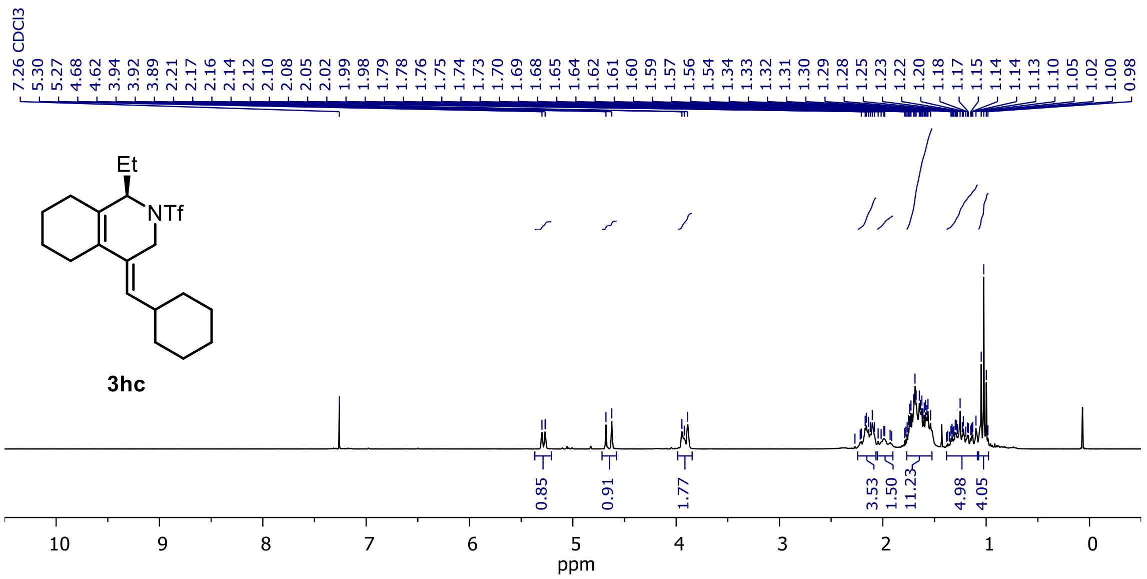

DEPT-135

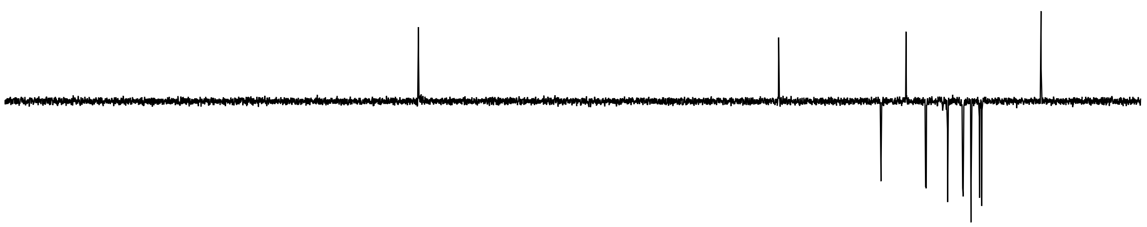

^{13}C NMR (75 MHz, $CDCl_3$)

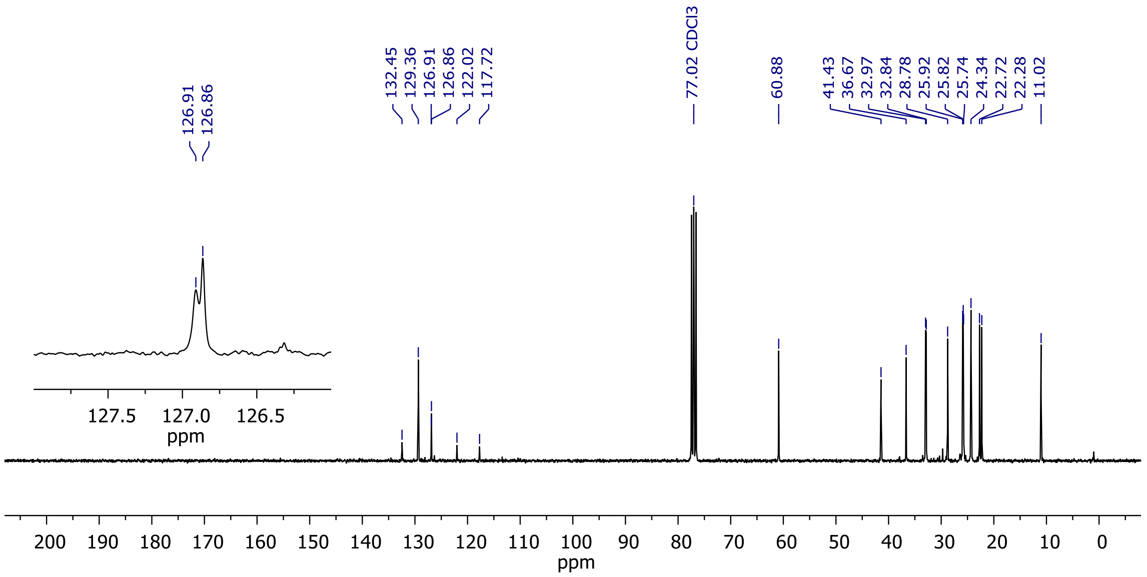

^{1}H NMR (300 MHz, CDCl$_3$)

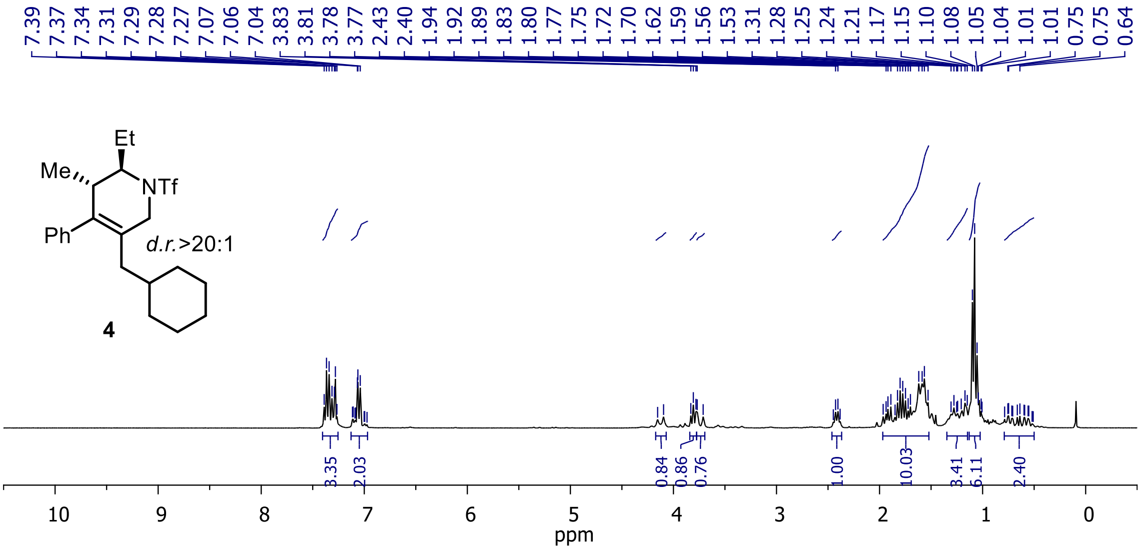

DEPT-135

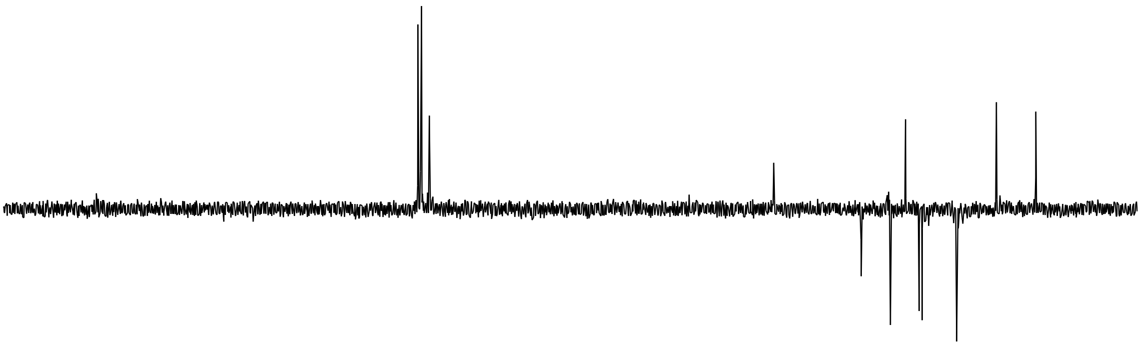

^{13}C NMR (75 MHz, CDCl$_3$)

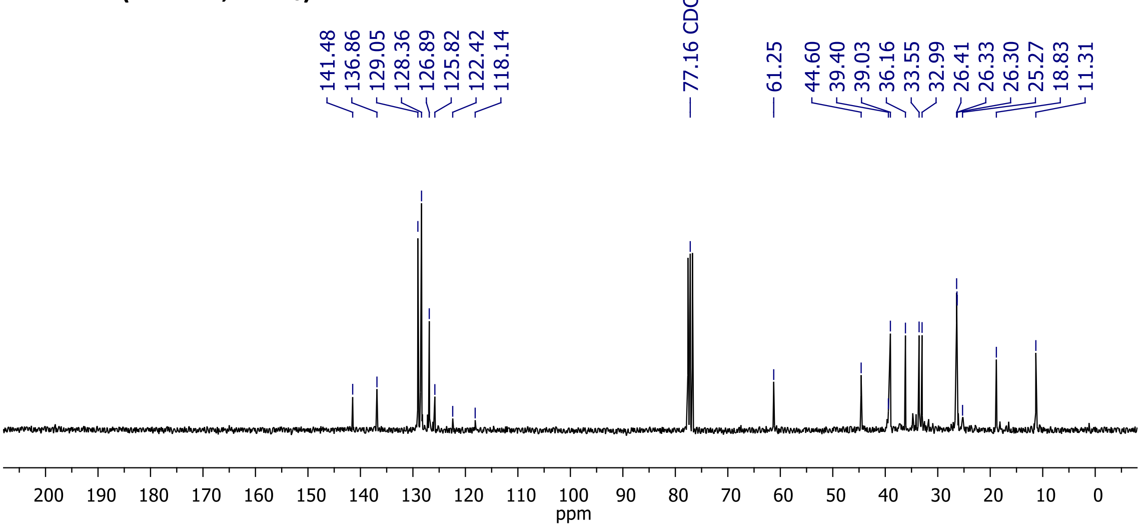

^{1}H NMR (300 MHz, CDCl$_3$)

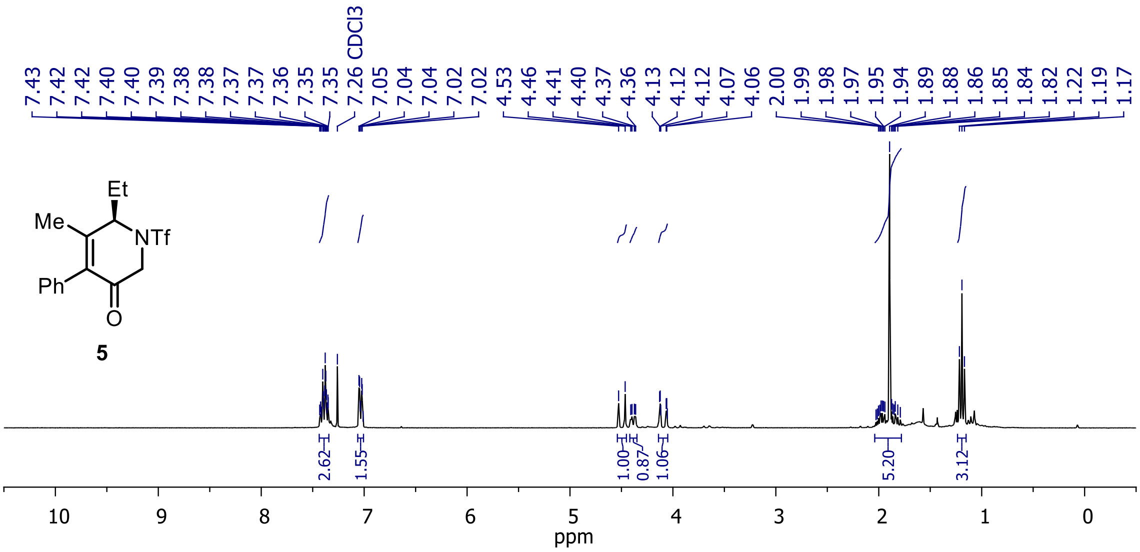

DEPT-135

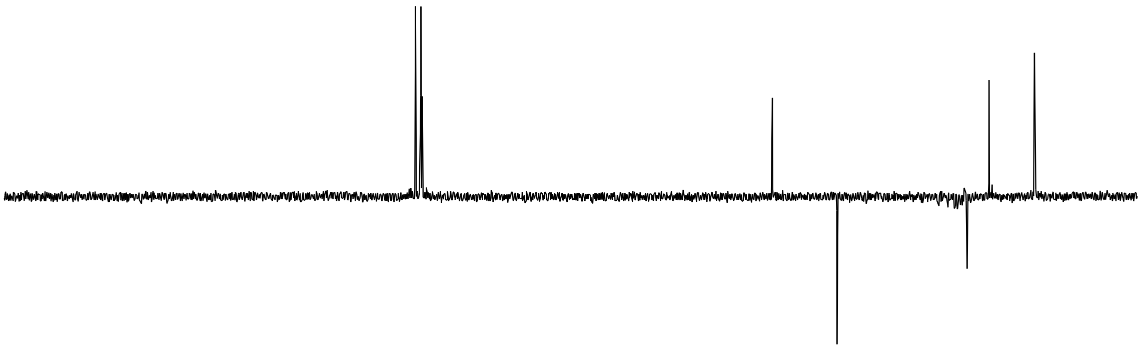

^{13}C NMR (75 MHz, CDCl$_3$)

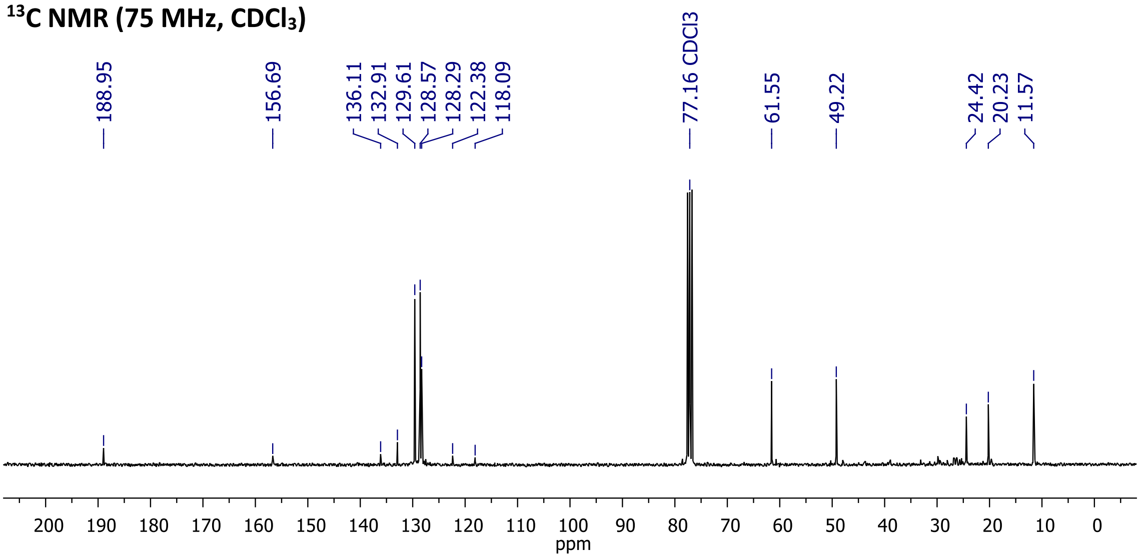

^{1}H NMR (300 MHz, CDCl$_3$)

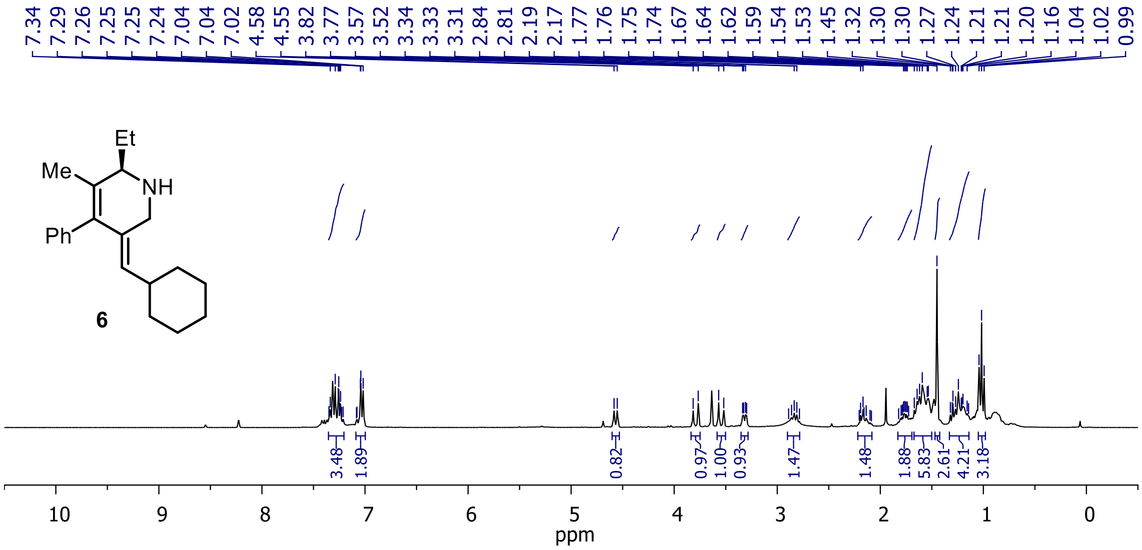

DEPT-135

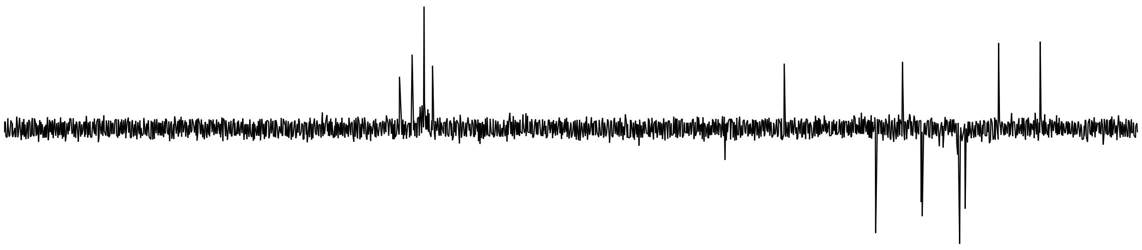

^{13}C NMR (75 MHz, CDCl$_3$)

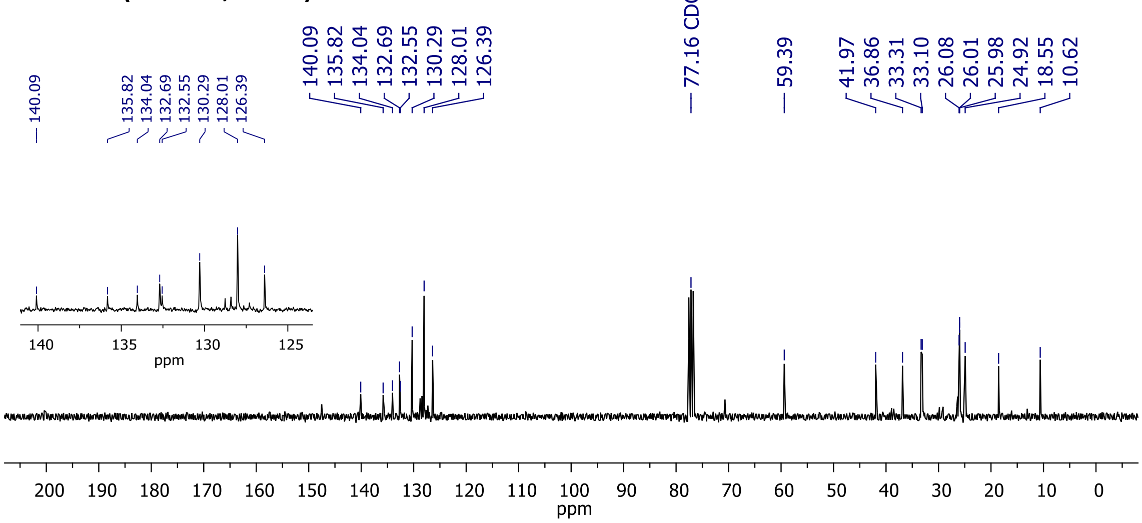

^{1}H NMR (300 MHz, $CDCl_3$)

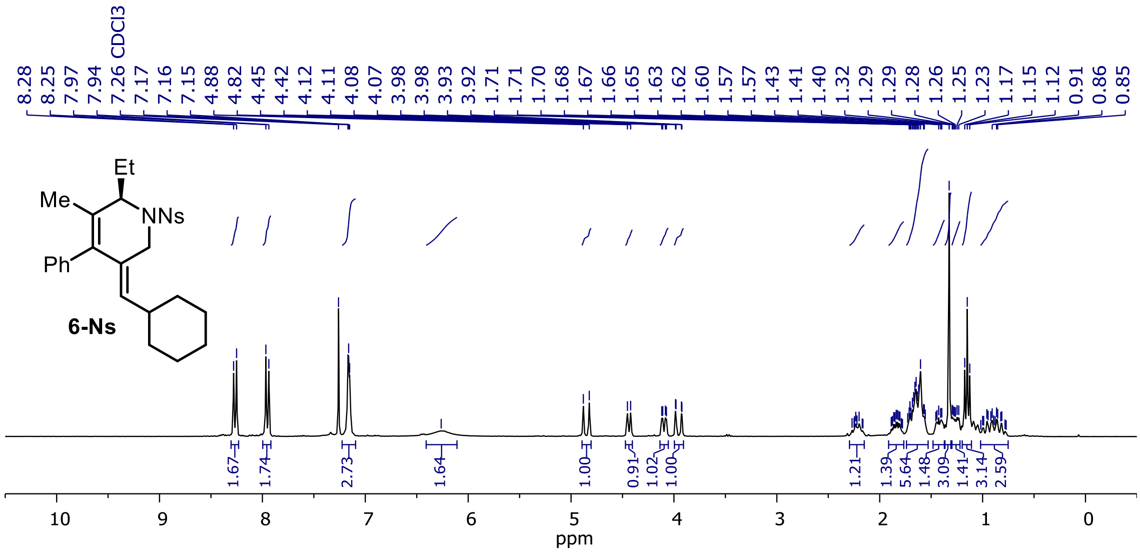

DEPT-135

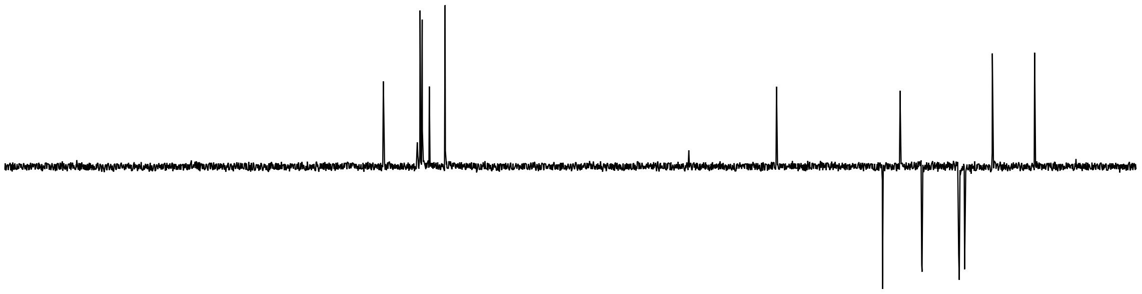

^{13}C NMR (75 MHz, $CDCl_3$)

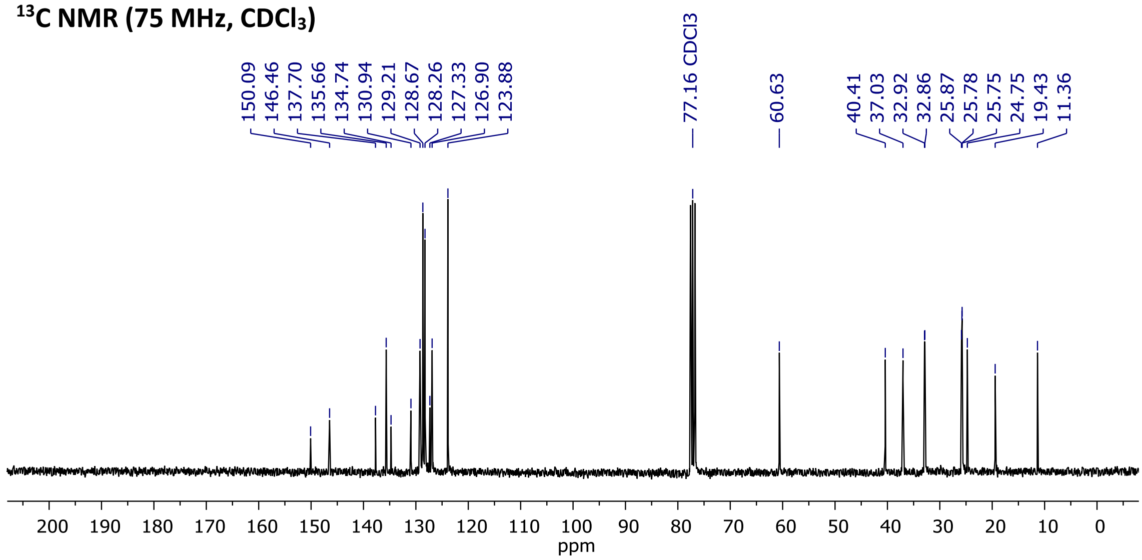

^{1}H NMR (300 MHz, $CDCl_3$)

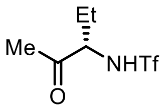

7

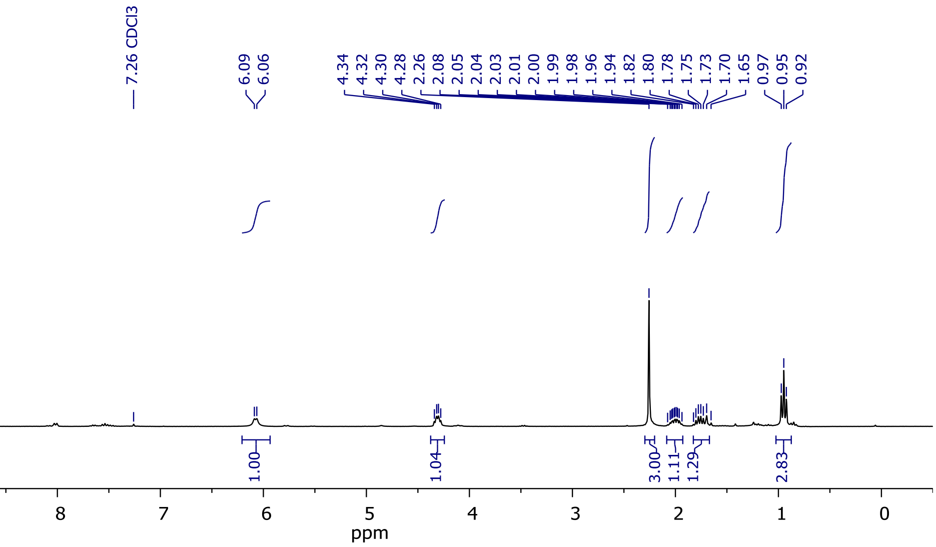

DEPT-135

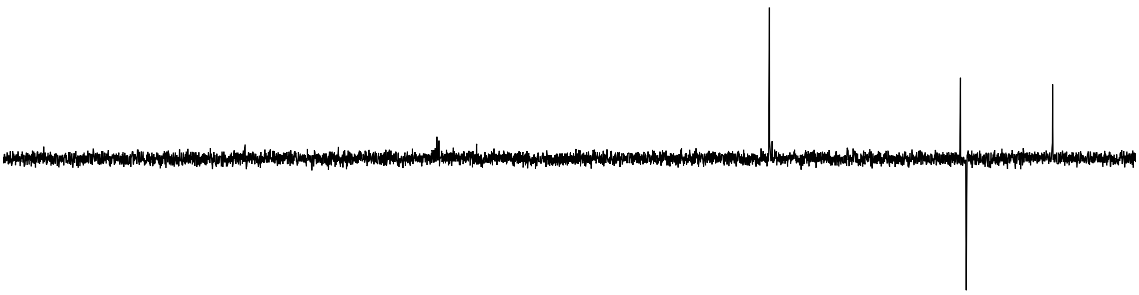

^{13}C NMR (75 MHz, $CDCl_3$)

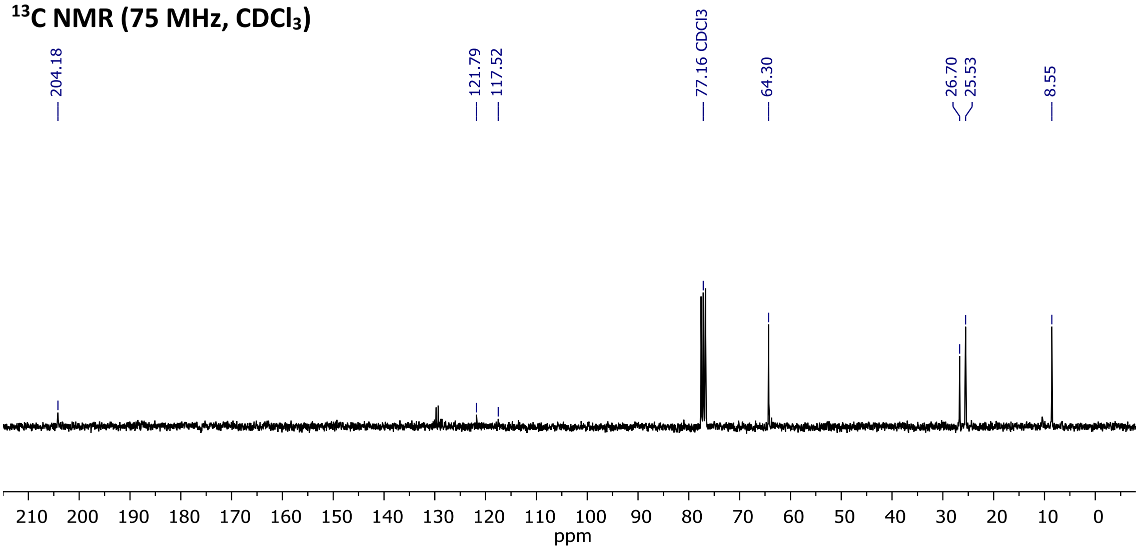

^{1}H NMR (300 MHz, CDCl$_3$)

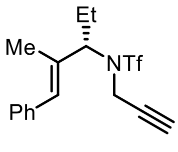

8

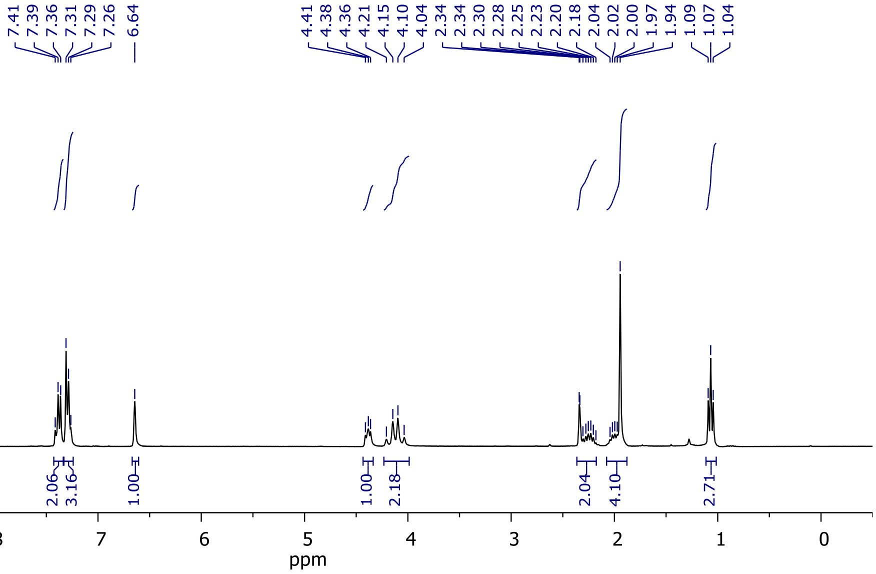

DEPT-135

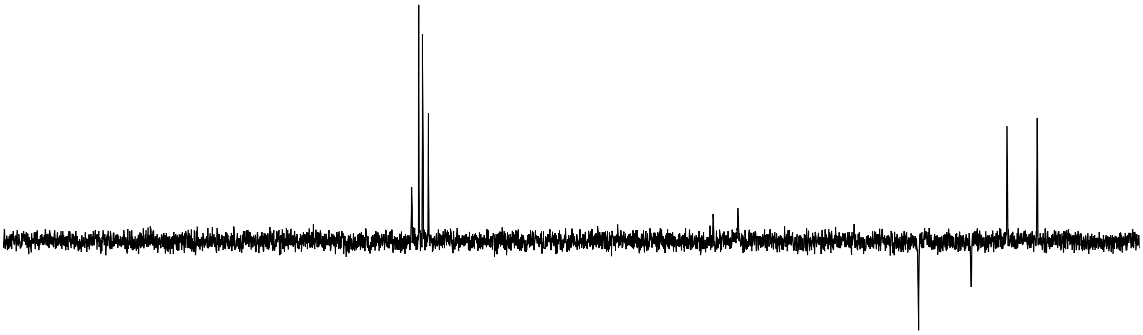

^{13}C NMR (75 MHz, CDCl$_3$)

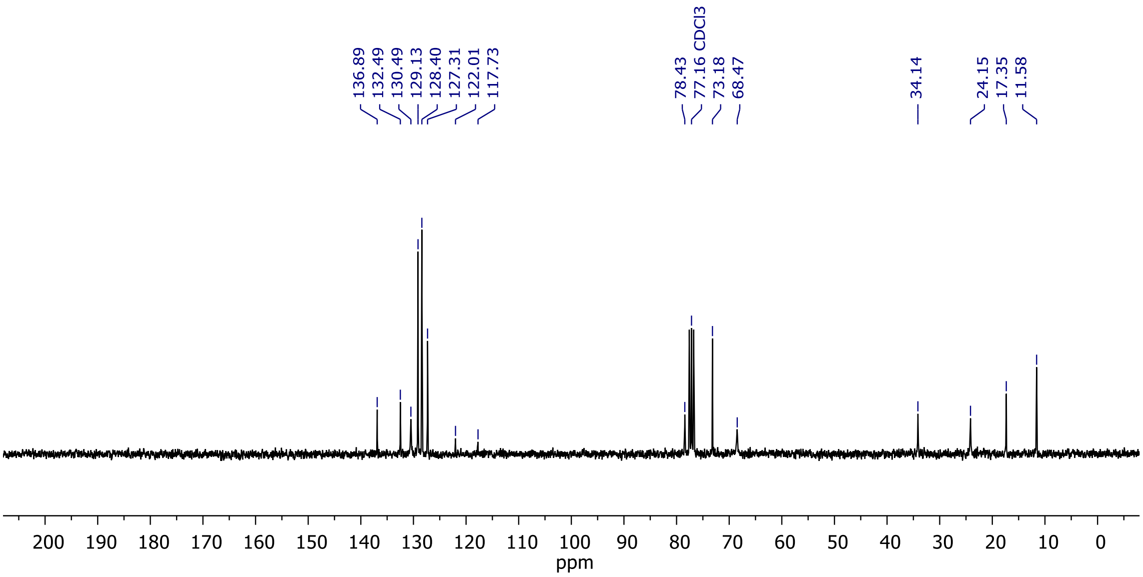

^{1}H NMR (300 MHz, $CDCl_3$)

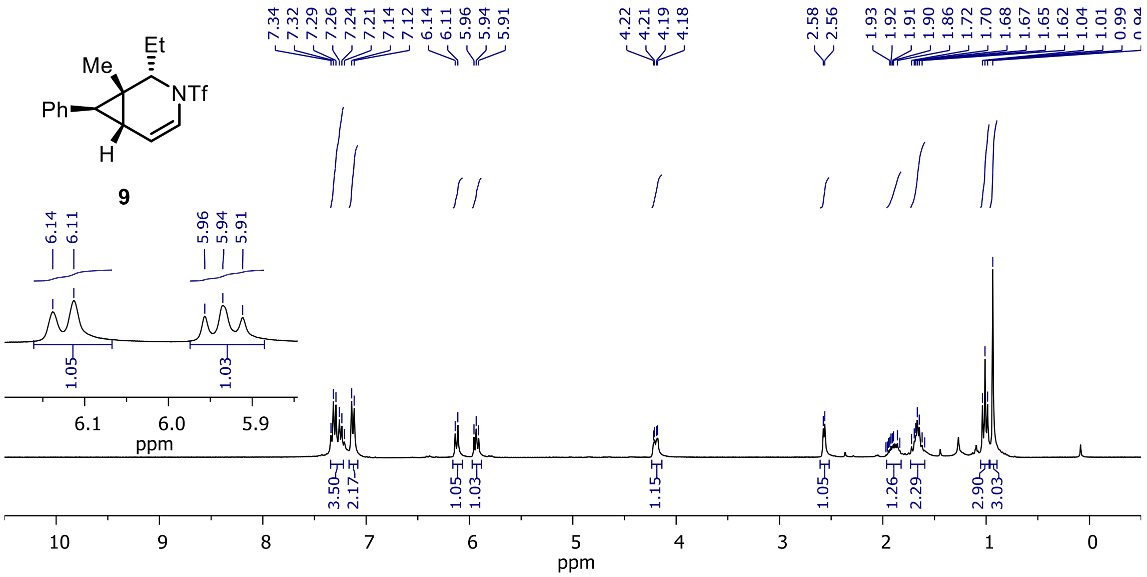

DEPT-135

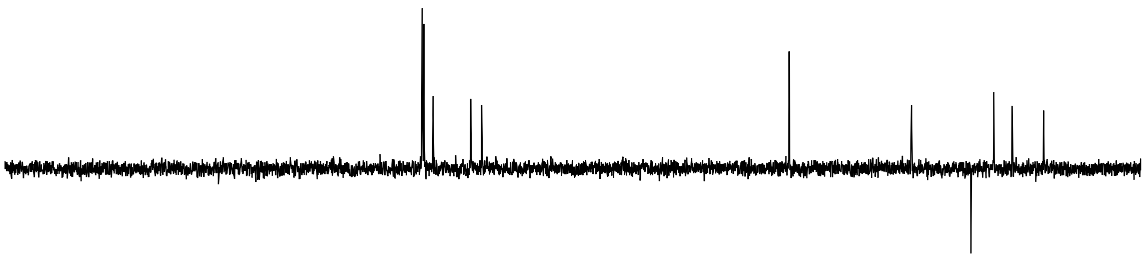

^{13}C NMR (75 MHz, $CDCl_3$)

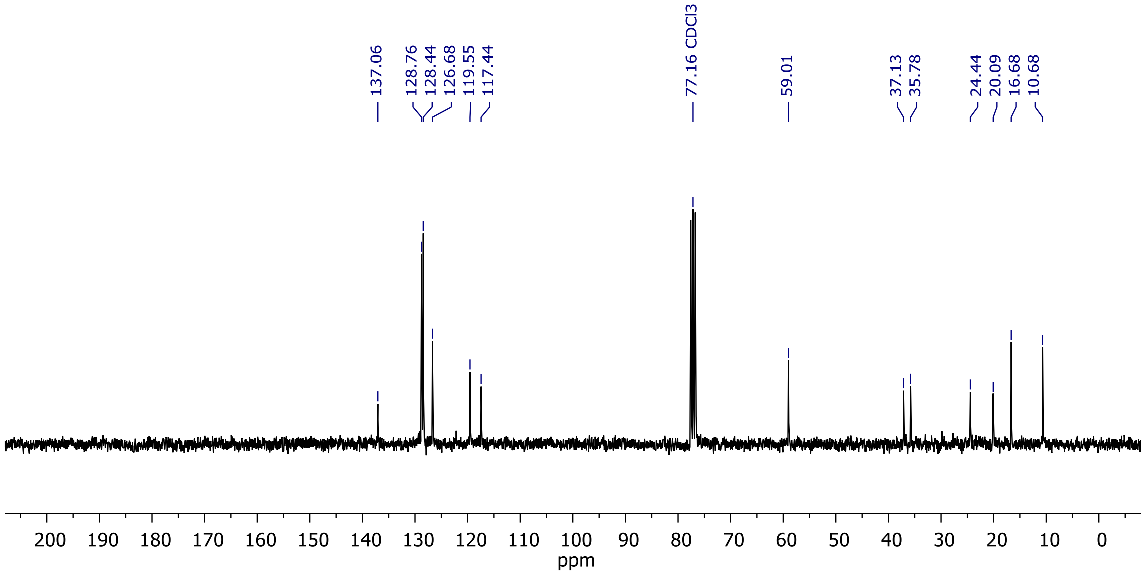

Supplement: Supplementary file 1 — ja1c01929_si_001.pdf [file ja1c01929_si_001.pdf]
